# dyphAI dynamic pharmacophore modeling with AI: a tool for efficient screening of new acetylcholinesterase inhibitors

**DOI:** 10.3389/fchem.2025.1479763

**Published:** 2025-02-04

**Authors:** Yasser Hayek-Orduz, Dorian Armando Acevedo-Castro, Juan Sebastián Saldarriaga Escobar, Brandon Eli Ortiz-Domínguez, María Francisca Villegas-Torres, Paola A. Caicedo, Álvaro Barrera-Ocampo, Natalie Cortes, Edison H. Osorio, Andrés Fernando González Barrios

**Affiliations:** ^1^ Grupo de Diseño de Productos y Procesos (GDPP), Department of Chemical and Food Engineering, Universidad de los Andes, Bogotá, Colombia; ^2^ Computational Bio-Organic Chemistry (COBO), Department of Chemistry, Universidad de los Andes, Bogotá, Colombia; ^3^ Grupo Natura, Facultad de Ingenieria, Diseño y Ciencias Aplicadas, Departamento de Ciencias Biológicas, Bioprocesos y Biotecnología, Universidad ICESI, Cali, Colombia; ^4^ Centro de Investigaciones Microbiológicas (CIMIC), Department of Biological Sciences, Universidad de los Andes, Bogotá, Colombia; ^5^ Grupo Natura, Facultad de Ingenieria, Diseño y Ciencias Aplicadas, Departamento de Ciencias Farmacéuticas y Químicas, Universidad ICESI, Cali, Colombia; ^6^ Grupo de Investigación en Química Bioorgánica y Sistemas Moleculares (QBOSMO), Faculty of Natural Sciences and Mathematics, Universidad de Ibagué, Ibagué, Colombia

**Keywords:** docking, MD simulations, pharmacophore, acetylcholinesterase, machine learning

## Abstract

Therapeutic strategies for Alzheimer’s disease (AD) often involve inhibiting acetylcholinesterase (AChE), underscoring the need for novel inhibitors with high selectivity and minimal side effects. A detailed analysis of the protein-ligand pharmacophore dynamics can facilitate this. In this study, we developed and employed **dyphAI**, an innovative approach integrating machine learning models, ligand-based pharmacophore models, and complex-based pharmacophore models into a pharmacophore model ensemble. This ensemble captures key protein-ligand interactions, including π-cation interactions with Trp-86 and several π-π interactions with residues Tyr-341, Tyr-337, Tyr-124, and Tyr-72. The protocol identified 18 novel molecules from the ZINC database with binding energy values ranging from −62 to −115 kJ/mol, suggesting their strong potential as AChE inhibitors. To further validate the predictions, nine molecules were acquired and tested for their inhibitory activity against human AChE. Experimental results revealed that molecules, 4 (P-1894047), with its complex multi-ring structure and numerous hydrogen bond acceptors, and 7 (P-2652815), characterized by a flexible, polar framework with ten hydrogen bond donors and acceptors, exhibited IC₅₀ values lower than or equal to that of the control (galantamine), indicating potent inhibitory activity. Similarly, molecules 5 (P-1205609), 6 (P-1206762), 8 (P-2026435), and 9 (P-533735) also demonstrated strong inhibition. In contrast, molecule 3 (P-617769798) showed a higher IC_50_ value, and molecules 1 (P-14421887) and 2 (P-25746649) yielded inconsistent results, likely due to solubility issues in the experimental setup. These findings underscore the value of integrating computational predictions with experimental validation, enhancing the reliability of virtual screening in the discovery of potent enzyme inhibitors.

## 1 Introduction

The global average life expectancy reached 73.4 years in 2019, with healthy life expectancy increasing by 8% due to reduced mortality rates (Mortality and global health estimates; Li et al., 2023). However, the length of time spent on health issues remained constant. This raises challenges to global health systems due to the prevalence of age-related diseases like Alzheimer’s disease, which is the seventh leading cause of death globally and impacts over 416 million individuals (Prince et al., 2016; Anonymous, 2021a; Anonymous, 2021b; Gustavsson et al., 2023). Factors such as environmental conditions, genetics, age (Silva et al., 2019; Stępkowski et al., 2020), and others contribute to Alzheimer’s disease (Breijyeh and Karaman, 2020; Scheltens et al., 2021), necessitating thorough research and efficient management to improve the wellbeing of the elderly population (Crestini et al., 2022; Whitfield et al., 2023; Delacourte, 2006; Jomova et al., 2023; Tolar et al., 2024; Vojtechova et al., 2022; Korczyn and Grinberg, 2024). Researchers have proposed three fundamental hypotheses to explain the origins of Alzheimer’s disease (Leng and Edison, 2020; Wang et al., 2023), including the amyloid hypothesis, copper toxicity (Ejaz et al., 2020), and cholinergic dysfunction (Wilkinson et al., 2004; Camps and Munoz-Torrero, 2005; Giacobini et al., 2022). The final hypothesis suggests reduced levels of the neurotransmitter acetylcholine in brain areas linked to cognition contribute to Alzheimer’s disease progression (Amenta et al., 2001; Carotenuto et al., 2022). Consequently, pharmacological interventions aimed at elevating acetylcholine (ACh) concentration represent a promising avenue for potential palliative treatment.

**FIGURE 1:**
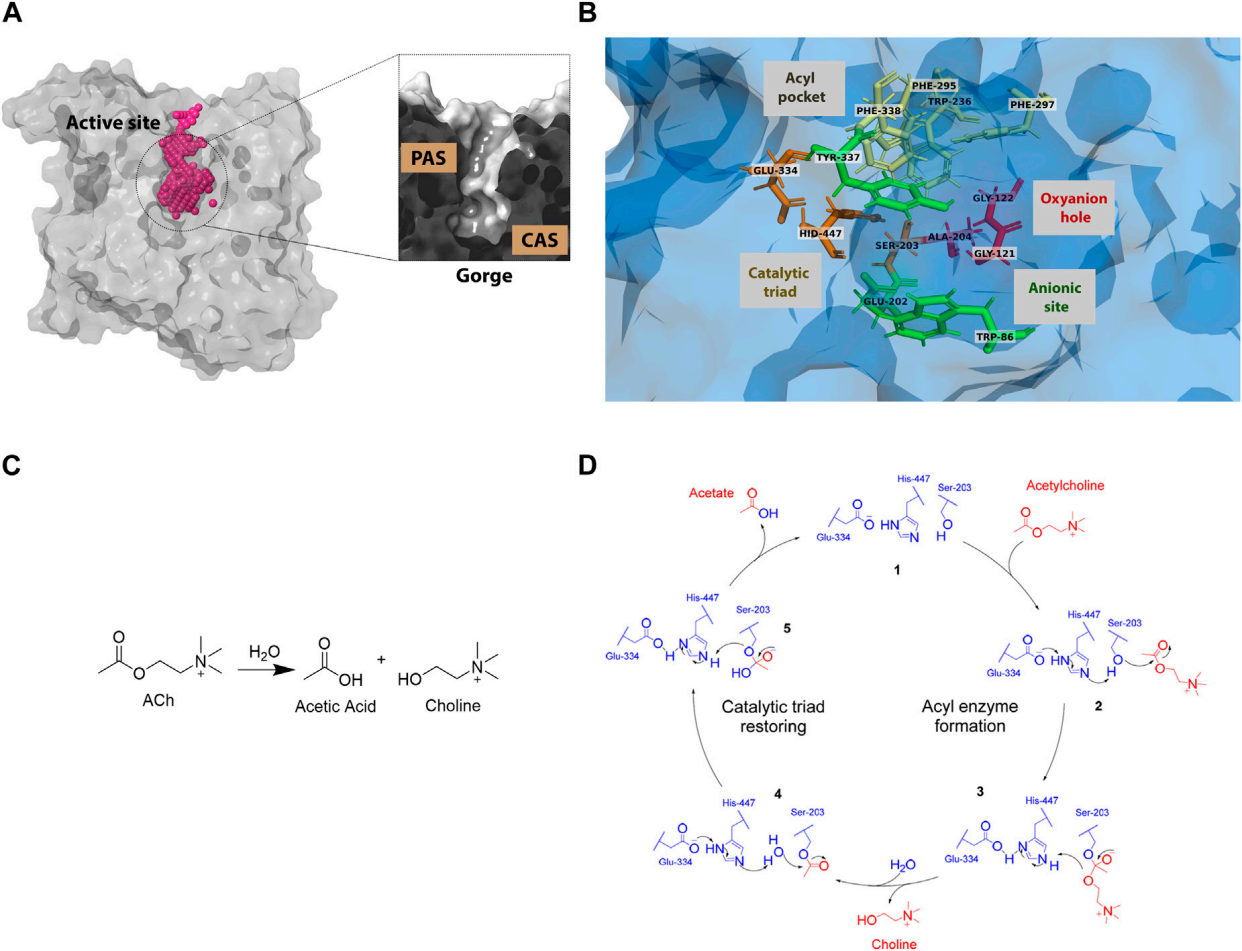
Human acetylcholinesterase’s active site: **(A)** structure and surface **(B)** active site residues with subsites highlighted. (PDB entry: 4EY6) **(C)** Acetylcholine hydrolysis reaction **(D)** Catalytic cycle of the acetylcholinesterase enzyme.

Acetylcholinesterase (AChE) is a hydrolase enzyme that breaks down ACh (Walczak-Nowicka and Herbet, 2021) through hydrolysis. Figure 1C illustrates this process (Komersová et al., 2005; Aroniadou-Anderjaska et al., 2023). Inhibiting AChE can maintain elevated levels of ACh by obstructing its degradation. Several compounds have demonstrated the ability to elevate ACh levels through AChE inhibition (Carotenuto et al., 2022; Akhoon et al., 2020; Marucci et al., 2021), indicating a potential avenue for enhancing Alzheimer’s disease treatment. However, these compounds often cause side effects (Akhoon et al., 2020). Finding more selective and powerful inhibitors is crucial to reduce these side effects, which are likely caused by their non-specific interactions with other enzymes.

The human acetylcholinesterase (huAChE) protein consists of five domains: S1, S2, S3, S4, and the Ω-loop (Cheng et al., 2017; Cheung et al., 2012; Dvir et al., 2010). The active site of huAChE exhibits a gorge-like structure (Figure 1A), with approximate dimensions of 20 Å in height, 5 Å in width, and 5 Å in length (Moghadam et al., 2021). The active site has two subsites: the peripheral anionic site (PAS) and the catalytic anionic site (CAS) (Figure 1B). The CAS has four areas: the acyl pocket, the catalytic triad, the oxyanion hole, and the anionic site (Wlodek et al., 1997). The PAS acts as a bridge for ligands to enter and leave the CAS. These areas are crucial for the acetylcholine hydrolysis reaction (Figures 1C, D) (Silman and Sussman, 2017).

Research on the steric and electronic properties of AChE inhibitors has been extensive (Wu et al., 2020; Zhou et al., 2017; Jang et al., 2018), but there is a lack (Sanson et al., 2011) of comprehensive study across all AChE inhibitor families (Akhoon et al., 2020; Gao et al., 2022; Pérez-Sánchez et al., 2021). While some studies have examined the conformational plasticity of protein-ligand interactions using advanced simulation methods (Lazim et al., 2020; Heilmann et al., 2020; Kaynak et al., 2022; Yasuda et al., 2020; Célerse et al., 2024), it is not common to use energetically unfavorable conformations to find protein inhibitors. Pharmacophore models (Langer et al., 2006) are widely used in drug discovery techniques (Qing et al., 2014; van Drie, 2012; Khedkar et al., 2007), but ensemble pharmacophore modeling can further harness their potential by combining multiple complex-based models. Artificial intelligence and machine learning models have gained significance in drug discovery (Carracedo-Reboredo et al., 2021; Dara et al., 2022; Li et al., 2024; Geng et al., 2024; Gupta et al., 2021), potentially playing a crucial role in virtual screening procedures.

**FIGURE 2:**
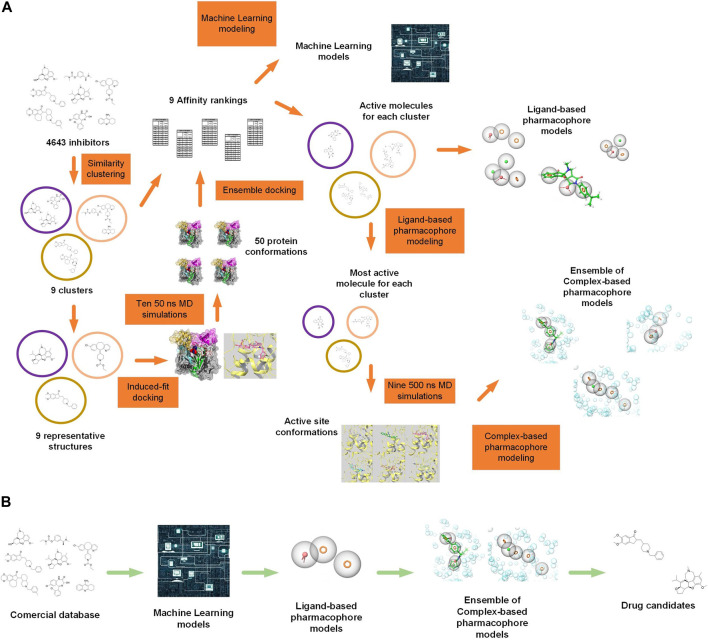
**(A)** Simplified representation of the methodology with illustrations. 4,643 known acetylcholinesterase (AChE) inhibitors were downloaded and categorized into 70 clusters or families based on their molecular structure. From these families, nine were selected for further analysis. The representative ligands from each of the nine families were docked into the AChE receptor employing induced-fit docking. Nine 50 ns MD simulations were performed based on the docked poses, along with an additional simulation of the AChE-galantamine complex. From these simulations, the protein’s conformations were taken out and used in an ensemble docking method, where compounds from each of the nine families were docked separately. The docking scores and experimental IC50 values were used to make an affinity ranking that showed which compounds from each family were the most active. These active compounds were used to generate machine learning models and ligand-based pharmacophore models. For the receptor-based method, the most active compound from each family was used to get the active site’s conformations from 500 ns of MD simulations. These active site conformations were used to generate complex-based pharmacophore models, and the pharmacophore models were combined to create a pharmacophore model ensemble. **(B)** Simplified representation of the virtual screening methodology with illustrations. A commercial molecule database, ZINC22 (Tingle et al., 2023) was utilized, and it was filtered sequentially using machine learning models, ligand-based pharmacophore models, and ensemble pharmacophore models based on ligand-receptor complexes. Obtained molecules are candidates to be inhibitors.

Here, we proposed a workflow to find new acetylcholinesterase inhibitors by identifying key molecular features needed to target the huAChE enzyme specifically. This approach involved an extensive *in silico* protocol (illustrated in Figures 2A, B, Supplementary Figures S1–S6), that included database management, ligand clustering, RMSD calculations, induced-fit docking, molecular dynamics simulations, TRAPP physicochemical analyses (Kokh et al., 2013), ensemble docking, pharmacophore modeling, and machine learning techniques. The method involved looking for different structures in new inhibitors, exploring the space of receptor conformations, combining computer and lab results, using machine learning models to find new inhibitors, and choosing which inhibitors to test in experiments.

We identified nine distinct inhibitor families, each associated with a predictive machine learning model, a ligand-based pharmacophore model, and an ensemble pharmacophore model derived from ligand-receptor complexes. The models enabled virtual screening of the ZINC_22_ database (Tingle et al., 2023), leading to the identification of 18 molecules with significant inhibitory potential against AChE.

The developed methodology and models, along with the insights gained, provide valuable contributions to the field and serve as a robust foundation for future research aimed at designing novel therapeutics for Alzheimer’s disease and related conditions.

## 2 Materials and methods

### 2.1 Yasser number one (YN1) affinity ranking

The affinity ranking of YN1 relies on the dynamic characteristics of the biological system, requiring the integration of computational and experimental data. A comprehensive workflow diagram illustrating this process is provided in Supplementary Figure S3.

#### 2.1.1 Similarity clustering of inhibitor structures

Inhibitors with an IC50 1 ≤ 99,000 nM against the huAChE were extracted from the Binding Database (BD) (https://www.bindingdb.org/) in isomeric SMILES format. These inhibitors were entered into the LigPrep tool of the Schrodinger 2023.1 suite (Schrodinger, LCC) to generate 3D structures using a pH of 7.4 ± 0.2 (Wessler et al., 2015).

We conducted structural similarity clustering of the molecules from the BD using the Canvas module within the Schrödinger 2023.1 suite (Schrödinger, LCC), employing specific settings including 64-bit precision, radial fingerprint type, Tanimoto similarity criterion, and the average linkage method. Subsequently, utilizing the Canvas results and the Kelley penalty value, we determined the optimal number of clusters, crucially balancing between over-clustering and under-clustering to ensure the resulting clusters meaningfulness.

A total of 70 ligands clusters were obtained from the BD. For each cluster, we computed statistical metrics including the average IC50, standard deviation of IC50, variation coefficient of IC50, and cluster size. These statistical parameters, alongside other selection criteria, were used to select 9 representative clusters for further analysis.

#### 2.1.2 Induced-fit docking of representative molecules from inhibitor clusters

To generate the Glide grid for induced-fit docking box, RMSD paired calculations were employed (*for more information, refer to the* Supplementary Material *on RMSD paired calculations*). The PDB files corresponding to the UNIPROT code P22303 of huAChE were downloaded, resulting in a total of 61 structures. Structures that were not wild-type, lacked protein-ligand complexes with covalent ligands, or had a resolution higher than 3.2 Å were excluded. This filtering process left a total of 18 structures with the following PDB codes: 4BDT, 4EY5, 4EY6, 4EY7, 4M0E, 4M0F, 5FOQ, 6CQU, 6F25, 6NEA, 6O4W, 6O4X, 6O5R, 6O5V, 6O50, 6O52, 6U3P, and 6U34. Employing RMSD pocket residues, paired calculations facilitated the identification of the key residues comprising the active site (key pocket), including Tyr-72, Asp-74, Trp-86, Gly-120, Gly-121, Tyr-124, Tyr-133, Glu-202, Ser-203, Trp-286, Phe-295, Phe-297, Tyr-337, Phe-338, Tyr-341, His-447, and Gly-448. The box was manually positioned at the center of the active site using a centroid formed by these residues.

Induced-fit docking validation was carried out using calculations for the huAChE protein-ligand system involving acetylcholine and galantamine molecules. We utilized the protein crystal structure with PDB code 4EY6 and addressed any missing residues using the Prime module within the Schrödinger 2023.1 suite (Schrödinger, LCC). Protonation states were assigned using PROPKA at a pH of 7.4 (Wessler et al., 2015). Energetic minimization of the crystal structures was carried out, restricting the maximum RMSD of heavy atoms to 0.18 Å. Standard protocols were applied with specific settings: redocking within 30 kcal/mol and 20 structures overall, employing XP precision, utilizing the OPLS3e force field, setting receptor van der Waals scaling to 0.5, ligand van der Waals scaling to 0.5, refining residues within 5 Å of ligand poses, enabling optimization of side chains, setting the maximum number of poses to 20, and sampling ring conformations within an energy window of 2.5 kcal/mol.

Following selection of representative molecules, based on molecular structure (cluster centroid), from the nine cluster families (refer to Section 2.1.1) were extracted. Subsequent induced-fit docking calculations were conducted on these molecules, employing the huAChE key pocket residues and adhering to the previously outlined docking settings.

#### 2.1.3 50 ns MD simulation of protein-ligand complexes of representative molecules from clusters of human acetylcholinesterase inhibitors and conformational sampling

The poses corresponding to the 9 representative molecular structures associated with families C31, C4, C19, C50, C35, C20, C36, C42, and C23 were extracted from the induced-fit calculations. In addition, the experimental pose of the galantamine ligand from crystal 4EY6 was also extracted. These 10 poses were used to carry out MD simulations with a production phase duration of 50 ns. The complete molecular dynamics procedure is reported in Supplementary Material.

The 10 trajectories obtained from the 50 ns molecular dynamics simulations were used to extract poses of the active site. The backbone RMSD value was monitored for the key pocket (refer to Section 2.1.2), and Trp-236, Glu-334, Ala-204, Gly-122 throughout the entire trajectory.

The RMSD values of: average ${\bar{x}}_{RMSD}$ , average plus, and minus twice the standard deviation ${\bar{x}}_{RMSD}\pm 2\delta$ were computed. Additionally, both the two smallest $l_{RMSD}$ , ${l+1}_{RMSD}$ and the two largest $h_{RMSD}$ , ${h-1}_{RMSD}$ values were found. This process yielded a total of 7 conformations per trajectory.

The seven conformations were analyzed using paired RMSD (for more information, refer to the Supplementary Material on RMSD paired calculations). Based on the RMSD, five conformations were selected from the original seven conformations for each trajectory.

#### 2.1.4 Ensemble docking and affinity ranking

The 50 key conformations were used as input for an ensemble docking procedure, where all compounds belonging to the key clusters 31, 4, 19, 50, 35, 20, 36, 42, and 23 were docked. The box was located manually in the active site center using a centroid composed of key pocket (refer to Section 2.1.2). Sequential docking of Glide HTVS, Glide SP, and Glide XP was applied. for docking the Epik module to state penalties was utilized.

The XP GScore values obtained from ensemble docking and the experimental inhibition values (IC50) of the compounds in key clusters were used to design an affinity score to rank the compounds in each cluster according to their experimental and computer-predicted inhibition capacities. Hence, the following affinity score, called Yasser’s number one (YN1), was employed:

$$LE_{i}=-\frac{XP\;GScore_{i}}{MW_{i}}\;i=1,2,\ldots .,N$$

$$YN1_{i}=\frac{LE_{i}-LE_{\min}}{LE_{\max}-LE_{\min}}+\left(1-\frac{{\log_{10}IC50}_{i}-{\log_{10}IC50}_{\min}}{{\log_{10}IC50}_{\max}-{\log_{10}IC50}_{\min}}\right)$$

where $LE_{i}$ is ligand efficiency, $MW_{i}$ is molecular weight, ${\log_{10}IC50}_{i}$ is the log base 10 of IC50, max is the maximum value inside the cluster, min is the minimum value inside the cluster, and N is the number of molecules of the cluster.

### 2.2 Machine learning modeling to first accurate and fast screening

A schematic of this procedure is presented in Supplementary Figure S4.

#### 2.2.1 Data splitting for training and testing

All molecules with an XP GScore value from ensemble docking were considered active molecules in this stage. Thus, active molecules were extracted from clusters 31, 4, 19, 50, 35, 20, 36, 42, and 23. These active molecules were randomly divided into two groups: 20% for testing and 80% for training. The training and testing sets of active molecules were used separately for decoy calculation, using the DUDE (Database of Useful Decoys: Enhanced) package, generating approximately 50 decoys per active molecule (Mysinger et al., 2012). Consequently, two libraries were obtained, one for testing and another for training, each containing actives and approximately 50 decoys for every active molecule.

#### 2.2.2 Clustering of molecular descriptors for decoys from the training library

A calculation of molecular descriptors was performed using RDKit for the decoys from the training library (RDKit: Open-source cheminformatics) (Landrum, 1997). A correlation analysis of the molecular descriptors was conducted, in which the correlation matrix was calculated, and the absolute value of each Pearson correlation coefficient was computed. Next, a feature scaling of the molecular descriptors was carried out, and variances of the scaled variables were calculated. It was considered that two variables are correlated if they have an absolute value of Pearson correlation coefficient greater than or equal to 0.9. To address correlated variables, the variable with the highest variance was chosen, and the rest were discarded. As a result, molecular descriptors that are not correlated were obtained.

The uncorrelated molecular descriptors were used to perform a k-means clustering, where the desired number of clusters was set equal to the number of actives in the training library. Later, the decoy corresponding to the centroid of each cluster was extracted, so that the centroids were used to generate a balanced training dataset.

#### 2.2.3 Training machine learning models

The actives and decoys from the balanced training dataset were taken, molecular descriptors were calculated using RDKit (RDKit: Open-source cheminformatics), and a correlation analysis was conducted. Then, a 5-fold cross-validation was performed for hyperparameter optimization using the following binary classification algorithms: logistic regression, support vector machine, decision trees, and random forests. Optuna was employed for optimization, aiming to find hyperparameter configurations that maximize the ROC-AUC. Once the optimal hyperparameters were determined, the four algorithms were trained using the complete balanced dataset.

#### 2.2.4 Testing machine learning models

The actives and decoys from the unbalanced test dataset were taken, and molecular descriptors were calculated using RDKit. Correlated variables found during training were eliminated. The four classification algorithms were then tested with the test molecules.

### 2.3 Ligand-based pharmacophore modeling

In the generation of a ligand-based pharmacophore model, both active molecules and decoys are essential components. Active molecules, identified by a YN1 score equal to or exceeding 1, were selected. Decoys corresponding to the identified active molecules were generated using the DUDE approach, resulting in the production of 50 decoys per active compound (Mysinger et al., 2012).

Ligand-based pharmacophore modeling was configured to identify the best alignment and the most common features. A target of 50 conformers was set, while the number of features in the hypothesis ranged from 3 to 6. The threshold percentage of actives required to match a hypothesis was established at 50%. The scoring function employed was the Phase Hypo Score. All calculations were conducted using the Phase module within the Schrödinger 2023.1 suite.

A schematic of this procedure is presented in Supplementary Figure S5.

### 2.4 Ensemble complex-based pharmacophore modeling

For each of the nine molecular dynamics (MD) trajectories, encompassing 500 nanoseconds each, both stable and unstable conformations of the active site were identified. These conformations were subsequently utilized to construct complex-based pharmacophore models by employing the ‘Develop Pharmacophore Model’ tool within the Schrodinger Phase module. Whilst default settings were initially applied, adjustments were made to the maximum number of features and mandatory features to refine and optimize the receiver operating characteristic-area under the curve (ROC-AUC) values.

A schematic of this procedure is presented in Supplementary Figure S6.

#### 2.4.1 Conformational clustering of ligand poses from ensemble docking

The compounds with the highest YN1 score were selected for each family, totaling 9 compounds. The poses of these selected compounds, acquired through ensemble docking, were employed in conformational clustering using Schrödinger 2023.1’s Conformer Cluster tool. The clustering used the average linkage method, atomic RMSD, and specified comparison regions involving Heavy atoms + OH, SH. We identified the most frequently observed pose among the various poses obtained from ensemble docking for each of the 9 ligands. These 9 poses were used to carry out MD simulations with a production phase duration of 500 ns, using the simulation procedure reported in Supplementary Material.

#### 2.4.2 TRAPP calculations and extraction of active site conformations from 500 ns MD simulations

The RMSF values for heavy atoms (backbone and side chain) were calculated for active site residues, key pocket (refer to Section 2.1.2). These RMSF values were sorted from highest to lowest. From each simulation, the 6 residues with highest RMSF values were selected for analysis.

The heavy atoms RMSD for the individual 6 active site residues, backbone RMSD for the whole protein, backbone RMSD for the active site residues, and RMSD for ligand heavy atoms were calculated for all MD simulations. An exponential moving average (EMA) with a period of 5,000 was computed for the 9 RMSD data sets, each consisting of 50,000 frames. A 5000-period was chosen for the EMA to maintain a 1:10 ratio with the total number of frames. To normalize the EMA values to a range between 0 and 1, the following process was applied: the smallest value in the dataset was identified and subtracted from each value, shifting the range so that the minimum became zero. Then, each adjusted value was divided by the range of the dataset, calculated as the difference between the maximum and minimum values. The normalized moving average was used to compute the numerical derivative. This was done by estimating the absolute value of the derivative at a given point as the difference between the function values at that point and at a slightly larger point, divided by the distance between the two points. Heatmaps were then generated using both the normalized moving average and the numerical derivative.

Moreover, graphs were generated utilizing the RMSD normalized moving average and numerical derivative to emphasize regions exhibiting a moving average and numerical derivative exceeding 0.6. The concurrent utilization of the moving average and numerical derivative facilitated the identification of time intervals where both stable and unstable conformations were observed. These graphs were crafted using MATLAB software, and the accompanying scripts are detailed in Supplementary Data 2.

The TRAPP-Analysis clustering tool was used to extract frames from time ranges of stable conformations (Yuan et al., 2020). On the other hand, the TRAPP-Pocket tool was used to extract frames from unstable conformations (Kokh et al., 2013). For TRAPP-Analysis the parameter “cluster” was set to 3 Å, “kmeans” was set to 3 Å, “zmax” was set to 8 Å, and “bb” was set of “on”. For TRAPP-Pocket the parameter “radius” was set to 5 Å, the active site residues were entered manually using the 17 residues previously identified, and the other settings were retained the same as the AR-TRAPP example found in the TRAPP example folders. The TRAPP-pocket python scripts were edited to enable the computing of 50,000 frames.

The frames obtained from clustering with TRAPP-Analysis of stable time ranges were called stable conformations. On the other hand, graphs of the descriptors of the physicochemical properties of the active site were made from the results of TRAPP-pocket, and frames showing atypical descriptor values were extracted. These frames were called unstable conformations.

#### 2.4.3 Complex-based pharmacophore modeling

##### 2.4.3.1 TRAPP calculations and extraction of active site conformations from 500 ns MD simulations

For each of the 9 molecular dynamics (MD) trajectories of 500 ns, the frames of stable and unstable conformations from the active site were employed to construct complex-based pharmacophore models using the Develop Pharmacophore Model tool in the Schrodinger 2023.1 suite Phase module. Default settings were utilized, and variations were made to the maximum number of features and mandatory features within the ranges of 6 to 5 and 3 to 2, respectively. The pharmacophoric hypotheses were combined, forming what we refer to as a hypothesis ensemble or pharmacophore model ensemble.

##### 2.4.3.2 Validation of hypothesis ensembles without YN2

A molecule is considered active if it can match at least one pharmacophore model from the ensemble. Conversely, a molecule is deemed inactive if it cannot match any pharmacophore model from the ensemble. We define the term “ensemble phase score” as the highest phase score obtained by a molecule among all the phase scores obtained for each pharmacophore model in the ensemble. Naturally, if a molecule does not match any model, it will have a score of zero. The accompanying scripts are detailed in Supplementary Data 2.

To validate the 9 ensembles, actives and decoys from each cluster were extracted. Compounds with a YN1 score greater than or equal to 1 were considered active for this stage. Decoys were obtained from the DUDE package (Mysinger et al., 2012), generating 50 decoys per active. ROC curves, BACC, recall, and specificity values were generated for each validation using the ensemble phase score and the enrichment calculator tool of Schrodinger 2023.1 suite. We selected the configuration of maximum number of features and mandatory features that exhibited the highest ROC-AUC value for the ensemble.

##### 2.4.3.3 Validation of hypothesis ensembles with YN2

To further refine the results and behavior of the ensembles, we introduced a metric called Yasser’s number two (YN2), which combines the values of ensemble phase score, the number of matching stable conformations, and the number of matching unstable conformations. This way, molecules with few matches to the pharmacophore models are penalized.

$${\hat{C}}_{unstable_{i}}=\frac{C_{unstable_{i}}}{C_{unstable\;total\;}}\;{\hat{C}}_{stable_{i}}=\frac{C_{stable_{i}}}{C_{stable\;total\;}}\;i=1,2,\ldots .,N$$

$$if\;Ph_{score\_i}\geq Ph_{score\;\min}\;then\;{\hat{Ph}}_{score}=\frac{Ph_{score_{i}}-Ph_{score\;\min}}{Ph_{score\;\max}-Ph_{score\;\min}}\;and\;YN2_{i}=\frac{\left({\hat{C}}_{unstable}\right)+\left({\hat{C}}_{stable}\right)+\left({\hat{Ph}}_{score}\right)}{3}$$

$$if\;Ph_{score\_i}<\;Ph_{score\;\min}\;then\;{\hat{Ph}}_{score}=0\;and\;YN2_{i}=0$$

where N is the number of molecules in the validation or virtual screening procedure, $C_{unstable_{i}}$ is the number of pharmacophore models derived from unstable conformations that fit with molecule i , $C_{unstable\;total\;}$ is the total number of pharmacophore models from unstable conformations, $C_{stable_{i}}$ is the number of pharmacophore models derived from stable conformations that fit with molecule i , $C_{stable\;total\;}$ is the total number of pharmacophore models from stable conformations, $Ph_{score\_i}$ is the ensemble phase score value for molecule i , $Ph_{score\;\min}$ is the minimum ensemble phase score value among known active molecules, and $Ph_{score\;\max}$ is the maximum ensemble phase score value among known active molecules.

We utilized YN2 for another round of validation. Initially, YN2 calculation was performed for all molecules. For a molecule to be considered active, it must have a value greater than or equal to a YN2 threshold; conversely, for a molecule to be considered inactive, it must have a value lower than a YN2 threshold. To determine the YN2 threshold, we generated an objective function that is the sum of recall and specificity. We optimized the objective function, obtaining the YN2 value that yielded the maximum value for the objective function.

Thus, from each ensemble validation, we extract the YN2 threshold, the value of $Ph_{score\;\max}$ , and $Ph_{score\;\min}$ . These three values, along with the number of stable and unstable conformations, serve as parameters for a subsequent virtual screening run. The accompanying scripts are detailed in Supplementary Data 2.

### 2.5 Database virtual screening

From the ZINC database, molecules with a molecular weight equal to or less than 500 Da and were in stock were downloaded, resulting in the retrieval of 11,012,710 molecules. The molecules underwent a virtual screening protocol consisting of three stages: machine learning models, ligand-based pharmacophore models, and ensemble of complex-based pharmacophore models. Initially, the ZINC molecules underwent screening using 9 machine learning models, each designed for families 31, 4, 19, 50, 35, 20, 36, 42, and 23. Subsequently, the identified actives from each model were combined, and duplicates were removed. The resulting library then underwent screening using ligand-based pharmacophore models designed for key clusters (refer to Section 2.3). The identified actives then proceeded to filtering with the ensemble models of pharmacophores corresponding to their respective families. Finally, we selected the top 500 ligands with the highest YN2 values from each cluster and subjected them to an ensemble docking procedure, incorporating HTVS, SP, XP, and MMGBSA filters. A schematic of this procedure is presented in Supplementary Figure S2.

### 2.6 Human acetylcholinesterase activity inhibition assay

The enzymatic activity of human acetylcholinesterase (hAChE) was quantified using a modified version of Ellman’s method. The assay was carried out in a 96-well plate format, with all fresh prepared components prior to use. The assay buffers included Buffer A (BA): 50 mM Tris-HCl, pH 8.0; Buffer B (BB): 50 mM Tris-HCl, pH 8.0, with 0.1% bovine serum albumin (BSA); Buffer C (BC): 50 mM Tris-HCl, pH 8.0, 0.1 M NaCl, and 20 mM MgCl₂·6H₂O; Acetylthiocholine (ATCI): 15 mM; Ellman’s reagent (DTNB): 3 mM in BC and Acetylcholinesterase (AChE): 0.22 U/mL in BA.

The assay was performed by first preparing a stock solution of each test compound at a concentration of 500 μg/mL. These compounds were then serially diluted from 1.4 μg/mL to 450 μg/mL in 50% DMSO. Galantamine hydrobromide, prepared at 306 μg/mL, served as the positive control. A total of nine test molecules derived from each cluster were included in the assay, and their concentrations in nM are summarized in Table 1.

**TABLE 1:** Identification of the molecules used in the inhibition assay against hAChE.

Molecule ID	Concentration (nM)^a^	MW (g/mol)	URL
1: P-14421887/ZINC10274013	1,070,400	420,42	https://mcule.com/P-14421887/
2: P-25746649/ZINC17245175	1,197,000	375,93	https://mcule.com/P-25746649/
3: P-617769798/ZINC16363937	903,700	497,97	https://mcule.com/P-617769798/
4: P-1894047/ZINC2256546	936,500	480,52	https://mcule.com/P-1894047/
5: P-1205609/ZINC18196844	1,295,000	347,41	https://mcule.com/P-1205609/
6: P-1206762/ZINC18197226	1,238,000	363,45	https://mcule.com/P-1206762/
7: P-2652815/ZINC100759000	942,400	477,51	https://mcule.com/P-2652815/
8: P-2026435/ZINC13691092	981,400	458,51	https://mcule.com/P-2026435/
9: P-533735/ZINC852356	1,129,400	398,45	https://mcule.com/P-533735/
Control: Galantamine hydrobromide	829,180	368,3	https://pubchem.ncbi.nlm.nih.gov/compound/Galantamine-Hydrobromide

^a^ The concentrations expressed in nM correspond to the initial concentration of 450 μg/mL.

Each assay well contained a final volume of 250 μL, consisting of:25 μL of ATCI, 125 μL of DTNB and 50 μL of BB for all plates.

For the blank control, 50 μL of 50% DMSO in BA was added. The negative control consisted of 25 μL of AChE and 25 μL of BA. Each test molecule was added as 25 μL of a 1:1 serial dilution in BA, combined with 25 μL of AChE.

The assay plates were prepared using an OpenTron OT-2 liquid handler, ensuring precise and reproducible liquid handling. The reactions were monitored kinetically at 407 nm using a Biotek Synergy H1 Multi-mode reader, set to 37°C. Absorbance was recorded every 45 s over a 15-min period.

The initial velocity of each reaction was determined from the early linear portion of the absorbance curve, and the percentage of inhibition was calculated using the following formula:

$$\%Inhibition=1-\frac{V_{0}\left(Sample\right)}{V_{0}\left(Control\right)}*10$$

where V0 (Sample) corresponds to the initial velocity of the reaction with the test sample, and V0 (Control) is the initial velocity in the absence of the test sample (control). IC₅₀ values were determined by plotting inhibitor concentration against percentage inhibition and calculating the point at which 50% inhibition was observed.

## 3 Results and discussion

### 3.1 Affinity ranking based on the dynamism of the biological system and experimental data

Here, we retrieved 4,643 huAChE inhibitors from the BD. To scrutinize the chemical composition of these inhibitors and uncover possible molecular patterns, we conducted a clustering analysis of their molecular structures. This analysis enabled us to categorize the inhibitors into separate clusters based on their molecular structure. The cluster analysis indicated that the optimal number of clusters was 383, accompanied by a Kelley penalty of 1,372 (as illustrated in Supplementary Figure S9). We also computed Kelley penalty values for cluster sizes of 50, 70, and 100, yielding 1,780, 1,570, and 1,540 Kelley penalty, respectively. Striking a balance between computational efficiency and reliability, we chose to proceed with 70 clusters. Consequently, the 4,643 inhibitors were grouped into 70 distinct clusters, synonymous of 70 compound families, based on their molecular structure.

Then, we decreased the number of clusters from 70 to reduce the computational workload in subsequent stages of the research, and to focus on families with a higher concentration of molecules. We leveraged the availability of IC50 values for each molecule in the BD, which reflect their respective inhibitory capacities by evaluating the average, standard deviation and coefficient of variation of IC50 and cluster size for each family. Additionally, we assigned a cluster ID to facilitate identification for each family.

Subsequently, we identify clusters from the initial set of 70 that demonstrated the following characteristics: the lowest average IC50 values (indicating high inhibitory potential), minimal IC50 standard deviation (reflecting low variability within the cluster), a moderate coefficient of variation (indicating consistent and uniform data), and a large population size (providing a broader pool of compounds for analysis). To achieve this goal, we established a threshold based on the mean values of IC50 average, IC50 standard deviation, variation coefficient, and population calculated across all families. The determined cutoff values were IC50 average of 16,268 nM, an average IC50 deviation of 14,901 nM, an average variation coefficient of 1.05, and an average population of 66 compounds.

Then, we employed these thresholds as a basis for creating cluster filters, refining them manually to maximize the population of clusters. The established criteria were: (i) average IC50 below 10,000 nM, (ii) IC50 standard deviation under 14,900 nM, (iii) population exceeding 58 molecules, and (iv) variation coefficient less than 1.7. A total of 17 clusters (Supplementary Table S2) satisfied at least 3 conditions (e.g., clusters 41, 1, 25, 51, 22, 56, 60, 46, 36, 31, 4, 19, 50, 35, 20, 42, 23), and among them, 3 clusters fulfilled all conditions (clusters 4, 19, 50). We opted to retain these 3 clusters that met all 4 conditions, along with the 6 clusters that satisfied 3 conditions and had the largest populations. The resultant clusters, namely, 31, 4, 19, 50, 35, 20, 36, 42, and 23, were utilized in subsequent stages. Moreover, we retrieved the compounds associated with the centroid of each cluster (Supplementary Table S3), which theoretically should represent an average molecular structure for each respective family.

We required the initial poses of the 9 centroid molecules on the AChE active site employing induced-fit docking. To validate the efficacy of the induced-fit docking, we performed induced-fit docking calculations for the huAChE protein-ligand system (PDB 4EY6) using acetylcholine and galantamine molecules. The induced-fit docking successfully reproduced the authentic pose of galantamine (Supplementary Figure S10), yielding an RMSD value of 0.93 between the predicted and actual poses. In the case of acetylcholine, induced-fit docking accurately replicated the documented pose from the literature (Supplementary Figure S11) (Raves et al., 1997). After validation, the centroid structures were successfully subjected to induced-fit docking, and the resulting ligand poses were used to perform 9 MD simulations of 50 ns. In addition, a 50 ns MD simulation was performed for the 4EY6 crystal, which contains the co-crystallized galantamine ligand. Energy, volume, temperature, and pressure were obtained during the MD production phase to confirm proper functioning of the 10 simulated systems. The kinetic and total energy demonstrated fluctuation around a stable value, while the volume graphs exhibited constant values. The temperature and pressure oscillated near 310 K and 1 bar, respectively (Supplementary Data S1).

The overall backbone RMSD value was monitored for 21 active site residues throughout the entire trajectory (Supplementary Figure S12). These included 17 residues (key pocket) identified in the RMSD paired calculations and 4 residues chosen for their crucial role in the subsite behavior based on literature (Trp-236, Glu-334, Ala-204, and Gly-122) (Wlodek et al., 1997) and these values were utilized to extract various conformations of the active site of the AChE (Supplementary Figure S12).

An statistical analysis of the RMSD data was conducted by calculating ${\bar{x}}_{RMSD}$ , ${\bar{x}}_{RMSD}\pm 2\delta$ , $l_{RMSD}$ , ${l+1}_{RMSD}$ , $h_{RMSD}$ , and ${h-1}_{RMSD}$ . This process yielded a total of 7 conformations extracted from each MD simulation, which was then repeated for all 10 MD, resulting in a total of 70 conformations (Supplementary Table S6). The 7 conformations obtained were treated as if they were different protein crystals and were subjected to the RMSD paired calculations (for further details, refer to the Supplementary Material on RMSD paired calculations). RMSD paired calculations were carried out using the all-atoms of 21 residues.

Five out of the 21 residues that showed the highest average RMSD from paired RMSD (Supplementary Table S4) were selected (Supplementary Table S5). RMSD heatmaps for these 5 residues were used to extract 5 conformations from the initial 7 conformations to achieve the highest conformational diversity. (All heatmaps can be found in Supplementary Data S1). Upon comparing the results from Supplementary Table S4, it was observed that the residues Asp-74, Glu-334, Tyr-341, His-447, Phe-295, and Phe-338 (as shown in Supplementary Figure S13) displayed significant movement in common among the 7 conformations, revealing changes in both the backbone and the side chain. Finally, 50 conformations were obtained and implemented in the ensemble docking procedure (Supplementary Data S1).

The ensemble docking process utilized 50 active site conformations obtained from 50 ns MD simulations as input, along with all compounds from families (key clusters). The outcome was an affinity ranking based on Glide XP GScore docking scores, encompassing all conformations that matched each ligand and identifying the conformation that best suited each ligand. Subsequently, a metric named Yasser’s Number 1 (YN1) was developed to rank compounds based on both their docking scores and experimental inhibition capacity (IC50). YN1 is comprised of two terms: the first term represents the normalized value of ligand efficiency, while the second term involves the inverted normalized logIC50. The inverted logIC50 value is calculated by subtracting the normalized value from 1 to reverse the scale. YN1 score spans from 0 to 2, where a score nearing 2 signifies greater computational and experimental inhibitory potential.

All ligands from each family were ranked using YN1 as an affinity score, obaitning one ranking for each family. Additionally, we evaluated the correlation of logIC50 and YN1; and XP Gscore and YN1 (Supplementary Figures S14, 15).

Table 2 shows the Recovery percentage of recovered molecules from ensemble docking (%), which represents the percentage of molecules that were selected from the ensemble docking procedure calculated over the number of molecules that entered, due to the fact that some molecules have had a poor XP Gscore so the ensemble docking was not reported by Glide, as they may or may not even have been able to fit into the active site of the receptors.

**TABLE 2:** Results of ensemble docking and YN1 implementation.

Cluster ID	Cluster initial size	Recovered molecules from ensemble docking	Recovery percentage of recovered molecules from ensemble docking (%)	Molecules with YN1 ≥1	Recovery percentage of molecules with YN1 (%)
4	59	58	98	25	43
19	64	59	92	30	51
20	261	156	60	66	42
23	1,455	291	20	95	33
31	48	41	85	11	27
35	68	62	91	44	71
36	47	47	100	20	43
42	1,038	241	23	139	58
50	68	46	68	16	35

Cluster initial size: The number of molecules belonging to the family before ensemble docking. Recovered molecules from ensemble docking: The number of molecules that Glide reports with an XP Gscore value. Recovery percentage of recovered molecules from ensemble docking (%): The percentage of molecules reported by Glide with an XP Gscore value relative to the initial number of molecules. Molecules with YN1 ≥ 1: The number of molecules with a YN1 value greater than or equal to 1. Recovery percentage of molecules with YN1 (%): The percentage of molecules with a YN1 value greater than or equal to 1 over the number of recovered molecules from ensemble docking.

The ensemble docking process and subsequent YN1 scoring enabled us to identify the most promising huAChE inhibitors. We set a YN1 score threshold above 1 to ensure a balanced analysis so it allows us to identify compounds that either demonstrated average performance in both experimental and computational assessments, exhibited poor experimental performance but strong computational performance, or showed strong experimental performance but weak computational.

In our research, the use of IC50 values is particularly valuable as it complements molecular docking rankings with empirical evidence. IC50 values are derived from *in vitro* biological assays (Cer et al., 2009), mimicking the protein’s physiological environment, hence directly measuring a compound’s ability to inhibit the protein. This direct measure offers a stronger correlation with *in vivo* biological activity. Molecular docking, however, is an *in silico* technique predicting compound-protein binding through computational simulations, providing insights into molecular interactions and potential binding sites but not guaranteeing biological activity.

Finally, the ligand with the highest YN1 was obtained from each ranking, resulting in 9 ligands presenting the best equilibrium between docking score and IC50 values (Supplementary Table S7). Among the selected compounds, inhibitors with IC50 values lower than 420 nM and XP GScore lower than −9.4 kcal/mol were obtained, demonstrating the interesting inhibitory capacity of the selected ligands.

### 3.2 Machine learning modeling

For this stage, we took the molecules obtained from the output of each of the 9 ensemble dockings to generate 9 ML models capable of classifying molecules as inactive or active with respect to each identified family of inhibitors.

The molecules recovered from ensemble docking were divided into libraries with a 20% portion allocated for testing and an 80% portion for training. In addition, approximately 50 decoys were generated for each active compound, providing both positive and negative instances for training and testing (Table 3). Consequently, two unbalanced libraries were formed, comprising positive and negative instances, and allocated for training and testing purposes separately. To ensure balanced training, we implemented a protocol involving clustering based solely on the molecular descriptors of the negative instances. This protocol enabled the extraction of the most diverse decoys, resulting in negative instances with a wide range of molecular descriptor values, thereby facilitating optimal model training. Then, we proceeded to train four classification algorithms: logistic regression, support vector machine, decision trees, and random forests. Cross-validation and hyperparameter optimization techniques were carried out to maximize the performance of each algorithm using the available data. Subsequently, average values for accuracy, recall, specificity, and ROC AUC were evaluated over multiple iterations of cross-validation.

**TABLE 3:** Data splitting for training and testing.

	Training		Testing	
Family	Number of actives	Number of decoys	Number of actives	Number of decoys
**C4**	47	2,450	11	637
**C19**	48	2,351	11	539
**C20**	125	6135	31	1,519
**C23**	233	13,261	58	3,649
**C31**	33	1,617	8	392
**C35**	50	3,220	12	931
**C36**	38	3,234	9	833
**C42**	193	11,502	48	2,695
**C50**	37	1813	9	441

Performance evaluation included assessing the models’ capacity to identify decoys excluded during the generation of the balanced training library as inactive, the models’ ability to classify the initial 4,643 molecules from the binding database as active or inactive, the capacity to classify all molecules belonging to the cluster/family, and the ability to classify molecules from the binding database that do not belong to the cluster/family. The outcomes of both training and testing the models for the nine families of inhibitors are detailed in Supplementary Tables S8–16.

In order to select the appropriate model, we employed an objective function comprised of specificity values, the proportion of true negatives identified within decoys excluded from training, the percentage of active compounds identified from the family, and the percentage of compounds from the binding database not associated with the family, then we established an ML model for each family, as delineated in Table 4. These ML models are to be applied in the subsequent sections to execute a virtual screening procedure. The scripts utilized for training the ML models and the .pkl files of the models are provided in Supplementary Data S2.

**TABLE 4:** Metrics of the best machine learning algorithms for each of the families. Random forest, RF; Support vector, SV.

Family	ML algorithm	Average metrics cross validation				Test metrics			
		Accuracy	Recall	Specificity	ROC AUC	Accuracy	Recall	Specificity	ROC AUC
**C4**	RF	0.990	0.980	1.000	0.990	0.995	1.000	0.995	0.998
**C19**	RF	1.000	1.000	1.000	1.000	0.998	1.000	0.998	0.999
**C20**	RF	0.988	0.976	1.000	0.988	0.992	0.871	0.994	0.933
**C23**	RF	0.970	0.961	0.979	0.970	0.988	0.948	0.989	0.969
**C31**	SV	0.969	0.967	0.967	0.967	0.995	0.875	0.997	0.936
**C35**	RF	1.000	1.000	1.000	1.000	1.000	1.000	1.000	1.000
**C36**	RF	1.000	1.000	1.000	1.000	1.000	1.000	1.000	1.000
**C42**	RF	0.995	0.990	1.000	0.995	0.998	0.979	0.999	0.989
**C50**	RF	0.946	0.918	0.975	0.946	0.982	1.000	0.982	0.991

### 3.3 Ligand-based pharmacophore modeling

Pharmacophore models were generated using the Phase module of Schrodinger 2023.1 from the molecules derived from the 9 ensemble dockings. We employed the DUDE package (Mysinger et al., 2012) to generate decoys at a ratio of approximately 50 decoys per active compound to validate the pharmacophore models. Thus, we generated 9 libraries comprising molecules with a YN1 score greater than or equal to 1 along with their respective decoys. The Phase calculation resulted in the creation of multiple ligand-based pharmacophore models for each of the nine clusters. However, only models with the highest area under the ROC curve (ROC AUC) were utilized.

**FIGURE 3:**
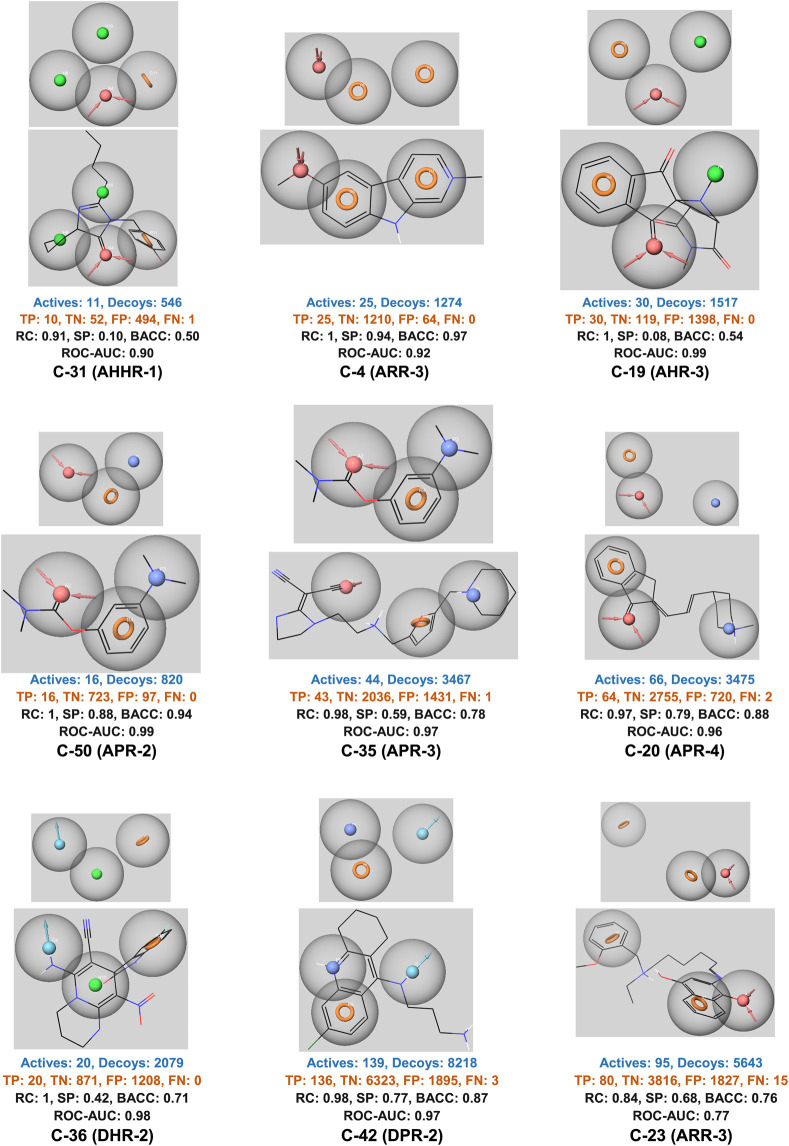
The best ligand-based pharmacophore models. H-bond acceptor (A) in red, H-bond donor (D) in light blue, positive point (P) in dark blue, aromatic (R) in orange, and hydrophobic (H) in green. TP, true positive; TN, true negative; FP, false positive; FN, false negative; RC, recall; SP, specificity; BACC, balanced accuracy; ROC-AUC, area under ROC curve.

Performance metrics, particularly the ROC AUC values, indicates that most models exhibit high predictive power (Figure 3, Supplementary Figure S16). For example, models such as C-31 (AHHR-1) and C-4 (ARR-3) achieve ROC AUC values of 0.90 and 0.92, respectively, which are indicative of strong discrimination capabilities. These high AUC values are complemented by other performance metrics like recall (RC) and specificity (SP), with some models achieving perfect recall (e.g., C-4 with an RC of 1), highlighting their ability to correctly identify all active compounds. However, the specificity values vary widely, suggesting that while some models are excellent at identifying actives, they might struggle with negatives, as seen in models with lower SP values. Models like C-4 and C-50 (APR-2) demonstrate high BACC values, underscoring their balanced approach in handling both true positives and true negatives effectively.

Overall, the ligand-based pharmacophore models present strength in terms of high recall and ROC AUC values, suggesting their potential utility in identifying novel active compounds. However, the variability in specificity and balanced accuracy across different models also points to areas for further refinement, particularly in reducing false positive rates to improve the models’ practical applicability in diverse drug discovery scenarios.

### 3.4 Ensemble complex-based pharmacophore modeling

The nine ligands with the highest YN1 from the nine families were employed to perform nine 500 ns MD simulations (Supplementary Table S7). To accomplish this, the poses of the ligands were extracted from the ensemble docking. A ligand geometry clustering was performed, where the poses associated with the 50 receptor conformations were clustered to extract the most frequently occurring pose (Supplementary Figure S17). The optimal number of clusters was determined by finding the global minimum in the Kelley penalty graphs (Supplementary Figures S18, 19). The pose with the highest frequency was defined as the centroid of the most populated cluster and was extracted as the initial pose for MD simulations.

The root-mean-square-deviation (RMSD) over backbone atoms of whole protein, active site, and ligand heavy atoms are reported in Supplementary Figures S20–22. Per-residue-root-mean-square-fluctuation (RMSF) over backbone atoms for the MD production stage of all systems and all heavy atoms (backbone and chain) were evaluated (Supplementary Figures S23–26 **)**. MD simulations showed kinetic and total energy fluctuation around a stable value, while the volume remainded constant. Temperature and pressure oscillated near 310 K and 1 bar, respectively (Supplementary Data S1).

Regarding protein flexibility (Supplementary Figures S27, 28 **)** RMSF backbone values for the 9 MD simulations of the 4EY6 complex showed the S2 domain (228–303) and S3 (331–407) with high fluctuations, suggesting flexibility. On the other hand, the S4 domain (435–457) and S1 domain (117–153) show lower fluctuations, indicating regions with rigidity. The Ω-loop domain (69–96) also shows moderate fluctuations, which could influence its function or interaction with inhibitors, as it is located directly in the active site. Furthermore, the RMSF for sidechain and backbone showed elevated values due to the higher flexibility of the sidechains. Regions with high flexibility, such as the S2 domain and Ω-loop, stand out even more when all heavy atoms of the residue are considered. Previous studies suggest that the omega-loop is involved in induced fit phenomena in the active site (Cheng et al., 2017), which is consistent with our findings of flexibility in the omega-loop.

Concerning ligand’s behavior in terms of its interaction with the protein we evaluated hydrogen bond occupancy (Supplementary Figures S30, 31), protein-ligand contact for all residues (Supplementary Figures S32–40), and protein-ligand contact for residues that were within 5 Å of the ligand at some point during the MD simulation (Supplementary Figures S41–49) to finally obtain the percentage frequency of interaction occurrences (Supplementary Figures S29).

The following residues obtained a percentage equal to or greater than 80% in terms of hydrophobic contacts for several MD simulations, with the number of total appearances in parentheses: Trp-86 (9), Tyr-341 (9), Tyr-124 (8), Tyr-337 (8), Gly-121 (6), Trp-286 (6), Tyr-72 (6), Asp-74 (4), Gly-448 (4), Hid-447 (4), Met-85 (4), Phe-297 (4), Phe-338 (4), Thr-75 (4), Thr-83 (4), Glu-292 (3), Ser-125 (3), Val-73 (3), Asn-87 (2), Leu-76 (2), Phe-80 (2), Ser-293 (2), Tyr-449 (2), Tyr-77 (2), Val-294 (2), Ala-343 (1), Arg-296 (1), Gln-71 (1), Gly-122 (1), Gly-342 (1), Gly-362 (1), Gly-366 (1), Gly-82 (1), Ile-451 (1), Leu-339 (1), Leu-398 (1), Lys-348 (1), Phe-295 (1), Pro-344 (1), Ser-438 (1), Trp-439 (1), Tyr-133 (1), Val-132 (1), Val-365 (1). Among these, the residues that appeared in 4 or more MD simulations were: Trp-86, Tyr-341, Tyr-124, Tyr-337, Gly-121, Trp-286, Tyr-72, Asp-74, Gly-448, Hid-447, Met-85, Phe-297, Phe-338, Thr-75, and Thr-83.

Nine MD simulations were employed to extract protein conformations for generating complex-based pharmacophore models. To do this, RMSF calculated for heavy atoms (backbone and side chain) were employed. The RMSF for Tyr-72, Asp-74, Trp-86, Gly-120, Gly-121, Tyr-124, Tyr-133, Glu-202, Ser-203, Trp-286, Phe-295, Phe-297, Tyr-337, Phe-338, Tyr-341, His-447, and Gly-448 were selected and ranked, resulting in 6 residues being chosen from each of the 9 simulations (Supplementary Table S17). These residues exhibited the greatest conformational variability throughout the entire simulation.

Individual heavy atoms RMSD for the 6 active site residues, backbone RMSD for the whole protein, backbone RMSD for the 17 active site residues, and RMSD for ligand heavy atoms were calculated for all MD simulations. To smooth the RMSD data, a moving average was constructed for the 9 data sets (Supplementary Figures S50–58). The moving average data was utilized to normalize data. Heatmaps were obtained with the normalized average to compare the behavior of the RMSD for the 9 data sets (Supplementary Figures S59–67).

In addition, the average was utilized to calculate the numerical derivative (Supplementary Figures S59–67). The derivative was used to identify regions of variation in the RMSD. Finally, we highlighted the moments where the moving average and numerical derivative were both greater than 0.6 (Supplementary Figures S68–76).

The parallel implementation of the moving average and numerical derivative enables the identification of stable and unstable conformations. If the RMSD normalized moving average does not show large variations over a time range, it suggests that the active site adopts stable conformations. Conversely, if the RMSD normalized moving average displays large variations, then the active site adopts unstable conformations. In order for the active site geometry to transition between stable conformations, it has to pass through an unstable conformation that enables the change. Therefore, high values of the numerical derivative can be used to identify time intervals where unstable conformations are more likely to occur, as these represent transition zones between stable conformations.

The zones of stable and unstable conformations can be identified in heatmap (Supplementary Figures S68–76) (A stable zone is identified when the graph remains in a green or blue color over a large time interval while an unstable zone is identified when the graph changes from green to blue and red squares are present in between). For example, the MD trajectory of Cluster 4, displayed three stable zones corresponding to 0–150, 180–430, and 460–500 ns (Supplementary Figures S59, S68) and two unstable zones for 150–180 and 430–460 ns. These regions were used to extract specific frames (stable or unstable conformations) for the generation of complex-based pharmacophore models (Table 5).

**TABLE 5:** Time intervals of stable and unstable conformations during 500 ns MD simulations. Number of extracted conformations in parentheses.

Cluster	Stable	Unstable
**C4**	(0–150**(3)**), (180–430**(2)**), (460–500**(3)**)	(150–180**(12)**), (430–460**(13)**)
**C19**	(0–60**(3)**), (100–200**(1)**), (220–330**(3)**), (350–500**(2)**)	(60–100**(8)**), (200–220**(12)**), (330–350**(8)**)
**C20**	(0–350**(3)**), (410–500**(1)**)	(350–410**(15)**)
**C23**	(0–110**(2)**), (140–410**(3)**), (450–500**(2)**)	(110–140**(12)**), (410–450**(12)**)
**C31**	(0–90**(1)**), (130–380**(4)**), (410–500**(2)**)	(90–130**(13)**), (380–410**(12)**)
**C35**	(0–50**(3)**), (60–210**(1)**), (250–500**(4)**)	(50–60**(15)**), (210–250**(15)**)
**C36**	(0–60**(3)**), (100–500**(4)**)	(60–100**(12)**)
**C42**	(0–60**(2)**), (130–500**(2)**)	(60–130**(17)**)
**C50**	(0–50**(2)**), (60–270**(5)**), (280–500**(2)**)	(50–60**(15)**), (270–280**(9)**)

The TRAPP-Analysis clustering tool was used to extract frames from stable zones and the TRAPP-Pocket tool was utilized to extract frames from unstable zones (Kokh et al., 2013; Yuan et al., 2020; Kokh et al., 2016). TRAPP-Analysis uses a clustering algorithm that serves to categorize the active site conformations into similar groups (Kokh et al., 2013; Yuan et al., 2020; Kokh et al., 2016), in this manner the conformations obtained correspond to the highest populated (energetically most favorable) conformations in the time range.

In the case of TRAPP-pocket, numerical descriptors related to the physicochemical properties of the active site were calculated for each frame of the trajectory (Figure 4) in order to identify energetically high conformations, so these conformations were assumed to be energetically unfavorable (Supplementary Figures S77–92; Table 5) and viceversa.

**FIGURE 4:**
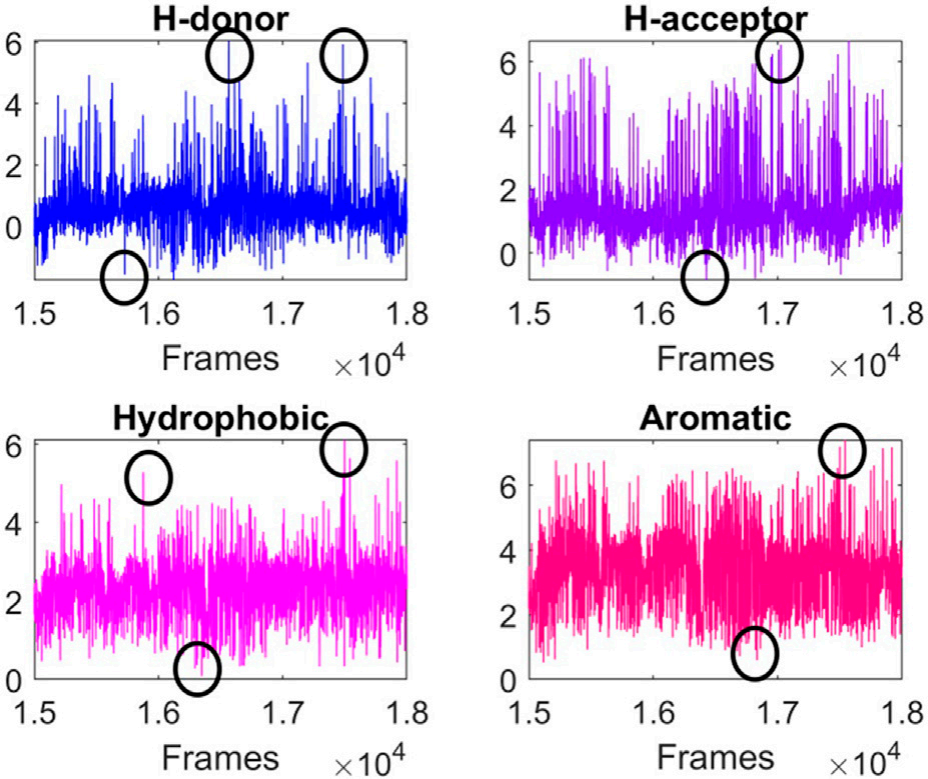
TRAPP-pocket results for the unstable zone of 150–180 ns. The plots belong to cluster 4.

Stable and unstable conformations were utilized to construct complex-based pharmacophore models using the Schrödinger 2023-1 phase module. These models were combined into an ensemble. Within this framework, (Supplementary Figures S93), molecules undergo virtual screening through all pharmacophore models derived from both stable and unstable conformations. Pharmacophore models are linked in the ensemble based on the logical OR operator. This means that for a molecule to be active, it is sufficient for just one individual model to classify it as active, and for a molecule to be inactive, it should not be classified as active by any model. Lastly, we define the ensemble Phase score as the highest Phase score attained by a molecule among all Phase scores obtained for each pharmacophore model in the ensemble.

The pharmacophore model ensembles were subjected to validation using the same actives and decoys employed in the ligand-based model validation. Subsequently, these validation results were introduced into the Schrodinger Enrichment Calculator tool, and ROC curves were generated for each ensemble (Supplementary Figures S94). Additionally, recall, specificity, ACC, and BACC values were obtained (Supplementary Table S18).

To improve the performance of the ensemble, we introduced a metric called Yasser’s number two (YN2), which combines the values of ensemble phase score, the number of matching stable conformations, and the number of matching unstable conformations. This metric averages the normalized values of ensemble phase score, the number of matching stable conformations, and the number of matching unstable conformations. A high YN2 value indicates that the molecule in question has an excellent ensemble phase score and fits with the majority of stable and unstable conformations.

For a molecule to be considered active employing YN2, it must have a value greater than or equal to a YN2 threshold and viceversa. To perform virtual screening using pharmacophore model ensembles coupled with YN2, 5 parameters were required: the number of stable and unstable conformations, the minimum ensemble phase score value among known active molecules ( $\left.Ph_{score\;\min}\right)$ , the maximum ensemble phase score value among known active molecules $\left(Ph_{score\;\max}\right)$ , and the YN2 threshold. The number of conformations were fixed while the other parameters were obtained in the ensemble validation process.

YN2 threshold was defined based on an objective function summing recall and specificity. After evaluating YN2 for all molecules (based on Supplementary Table S18) we carried out an optimization of an objective function corresponding to the sum of specificity and recall by varying the value of YN2. This way, we obtained the YN2 value that yielded the maximum value for the objective function (Supplementary Tables S19–S27; Supplementary Table S28).

The validation of the improved ensembles with the inclusion of YN2 was conducted (Supplementary Table S29). Furthermore, these findings were integrated into the Schrödinger Enrichment Calculator tool, and ROC curves were generated for each ensemble (Supplementary Figures S95). Supplementary Table S30 presents the percentage improvements in ensemble performance wht and without the evaluation of YN2, indicating a slight decline in recall values and a significant increase in specificity values in some cases, up to approximately 2000%. The ensembles displayed good metrics, with ROC AUC values greater than 0.8 in most cases.

**FIGURE 5:**
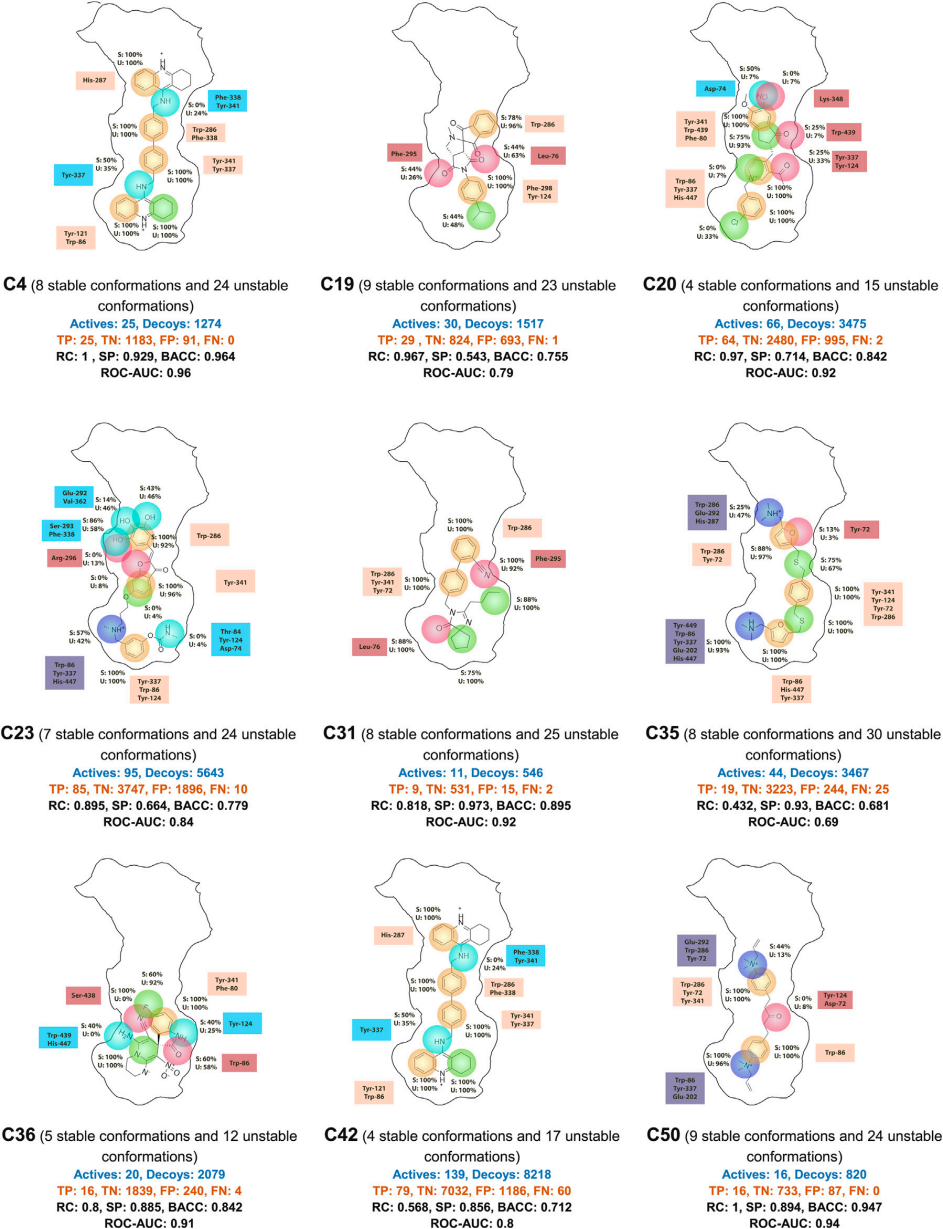
Summary of pharmacophore model ensembles for each cluster. Hydrophobic contacts are shown in green, H-bond donors in light blue, H-bond acceptors in red, aromatic interactions in beige, and positively charged groups in dark blue. The crucial residues for each feature are displayed next to the corresponding feature. S indicates stable conformation and U indicates unstable conformation. The percentages shown correspond to the number of times a feature appears in the total stable and unstable conformations. TP, true positive; TN, true negative; FP, false positive; FN, false negative; PC, precision; RC, recall; SP, specificity; BACC, balanced accuracy; ROC-AUC, area under ROC curve.

The most common features found among the ensembles correspond to aromatic interactions, including those caused by the residues Trp-86, Tyr-341, Tyr-337, Tyr-124, Tyr-72, and Trp-286 (Figure 5). Among these, Trp-86 and Trp-286 showed a capacity to generate π-π interactions between aromatic rings and also cation-π interactions with positive groups of the ligands. The role of these residues concerning the ligand entry is reported in other studies (Branduardi et al., 2005), indicating that these residues interact with the positive charges of the natural substrate acetylcholine and drive it into the active site. The fact of finding this dual capacity of these tryptophan residues in this research confirms their ability to interact with positive charges. On the other hand, the residues Tyr-341, Tyr-337, Tyr-124, and Tyr-72 showed the ability only to generate aromatic π-π interactions. But in some conformations, these tyrosine residues presented hydrogen bond interactions.

Protein dynamics are crucial for understanding how proteins interact with other molecules. Two key models for explaining protein dynamics are the induced-fit model and conformational selection (Galburt and Tomko, 2017). The induced-fit model suggests that when a ligand binds, it triggers changes in the protein’s shape to accommodate the ligand. Conversely, the conformational selection model proposes that the protein exists in various conformations, and ligand binding selects a specific one. Both models underscore the significance of protein dynamics in ligand binding. In our research, we considered the protein’s dynamic behavior in various computational calculations. Protein dynamics, including induced-fit and conformational selection, can significantly impact ligand binding. For instance, a molecule might not fit into a specific protein conformation, appearing inactive, yet it could effectively bind to other conformations. By employing ensemble pharmacophore models and other techniques, we gained valuable insights into the diverse protein conformations that a ligand could bind to, enhancing the accuracy of inhibitory activity predictions.

It was pivotal to link pharmacophore models corresponding to various active site conformations using the logical OR and YN2 on identified actives from each model individually. This approach allows for active site flexibility, contrasting with the use of a single pharmacophore model. Besides, we emphasize the importance of exclusion volumes in complex-based models. These volumes mimic the steric effects of the active site, effectively preventing recognition of ligands with unfavorable geometries as active. Exclusion volumes primarily aim to boost specificity and selectivity of pharmacophore models by narrowing the search space, thereby minimizing false positives resulting from improper ligand positioning.

Regarding seeking selective drugs, we consider crucial to individually address energetically unstable conformations. For instance, targeting specific protein conformations can yield more effective drugs with fewer side effects by reducing the likelihood of interference with the normal functioning of other enzymes. In molecular dynamics research, clustering techniques are commonly employed to extract representative frames of protein conformations observed during simulations. Traditional clustering groups frames based on their similarity in conformation, prioritizing the most frequently occurring ones in the simulation. However, this can lead to the loss of important information from less populated conformations, which may be equally relevant for understanding the binding’s behavior. This is the reason why the combined use of stable and unstable conformations is important.

### 3.5 Database virtual screening

**FIGURE 6:**
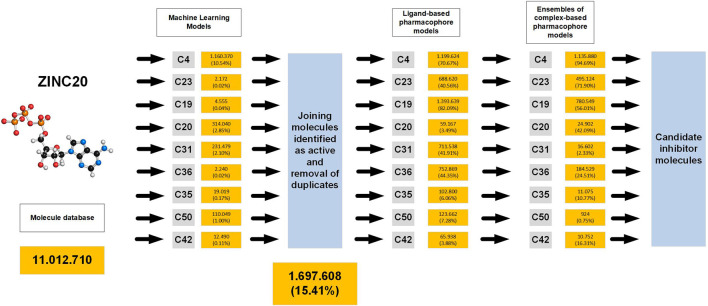
Scheme of the procedure used for virtual screening of databases and results obtained.

We aimed to implement the predictive models developed in this research to identify molecules that could function as novel inhibitors for AChE. To achieve this goal, we designed the virtual screening scheme depicted in Supplementary Figures S2. The scheme consists of a sequential use of machine learning models, ligand-based pharmacophore models, and complex-based pharmacophore model ensembles (Figure 6).

**FIGURE 7:**
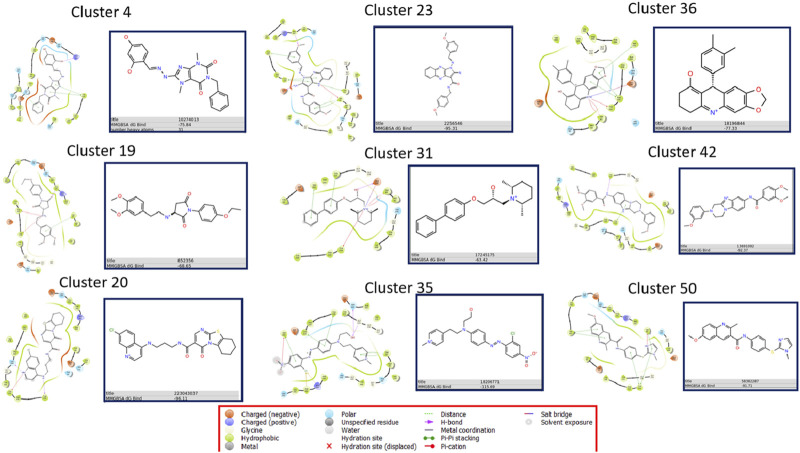
Summary of results of ensemble docking calculations the molecules with the best MMGBSA value for each family.

In order to find the inhibitors we extracted 11,012,710 molecules from the ZINC database. These molecules underwent virtual screening with the 9 ML models, and the active molecules identified by each model were aggregated into a combined library, resulting in a total of 1,697,608 retrieved molecules, which represents 15.41% of the original ZINC library. Subsequently, these molecules were subjected to screening with 9 ligand-based pharmacophore models and 9 ensembles of complex-based pharmacophore models. 500 molecules with the highest YN2 scores were selected from each final list, and subjected to ensemble docking calculations and MMGBSA free energy calculations to obtain those with the most interesting interactions (Supplementary Figures S96, 97; Figure 7).

Supplementary Figures S98–106 display the 2D interaction diagrams of the poses in the protein-ligand complex for these compounds. Free energy value varies from ZINC22983357 with −62.77 kJ/mol, which comes from the C31 family to ZINC18206771 from the C35 family, with −115.69 kJ/mol.

The virtual screening results underscore the effectiveness of an integrated approach that combines computational and experimental data through the development and application of machine learning and pharmacophore modeling. The order of filter application was specifically designed to progress from less to more precision in molecule searching. Initially, the ML models were trained to identify molecules from each family, unlike the ligand-based and complex-based pharmacophore models, which were trained with the molecules exhibiting the highest activity by YN1 from each family. Additionally, since the ML models were located at the first phases, we focused on seeking models with more specificity. This way, we filtered out inactive molecules initially, expecting the ligand-based and complex-based pharmacophore models to identify active molecules. The ML models proved very useful as they required lower computational resources compared to directly using pharmacophore models. This enabled successful management of the initial ZINC library of 11,012,710 molecules, reducing it by up to 15%.

Afterwards, the molecules underwent ligand-based pharmacophore modeling and complex-based pharmacophore ensemble modeling. Pharmacophore modeling is a powerful tool for drug discovery, but its effectiveness heavily relies on the availability of both active molecules and the biological target (Qing et al., 2014). However, when only the biological target is known, this approach encounters several challenges, making it difficult to identify promising drug candidates. It is assumed that pharmacophoric points represent regions where favorable protein-ligand contacts occur (Ertl et al., 2011). This holds particularly true for complex-based pharmacophores, which utilize the receptor’s structure in complex with the ligand for model creation. However, in the case of ligand-based or receptor-based pharmacophore models, the hypothesis might fail to capture locations where favorable binding contacts occur. This is why we positioned ligand-based models in second place, as complex-based models provide more precise information for identifying active molecules. This is evident when comparing the superior metrics of the complex-based pharmacophore ensemble models with those of ligand-based models. Additionally, another reason for prioritizing ligand-based models before complex-based ones is computational cost, as pharmacophore ensembles require more resources. Thus, our aim was to minimize the number of molecules reaching the complex models to optimize computational time and resources.

### 3.6 Experimental validation

After applying the virtual screening protocol (Supplementary Figures S2), we identified 18 candidate molecules based on their docking scores and MMGBSA free energy calculations. These molecules were prioritized for their predicted binding affinity and potential inhibitory activity against acetylcholinesterase (AChE).

To ensure a diverse representation across molecular families, we aimed to include at least one molecule from each family identified in the virtual screening results. The final selection was also constrained by commercial availability. Following an extensive search among several suppliers, we acquired nine molecules that were readily available for purchase. The decision to proceed with commercially available compounds allowed us to maintain a balance between molecular diversity and feasibility for experimental validation.

These nine molecules, spanning different families, were subjected to *in vitro* AChE inhibition assays to experimentally validate their predicted activity. This approach ensured that our experimental testing covered a range of chemical scaffolds, providing insights into structure-activity relationships and enhancing the robustness of our validation process.

The IC₅₀ values for each tested molecule are presented in Table 6. Molecules 4, 5, 6, 7, 8, and 9 exhibited IC₅₀ values equal to or lower than the control, indicating potent inhibition of acetylcholinesterase activity. In contrast, molecule 3 displayed a higher IC₅₀ value, suggesting a weaker inhibitory effect. Molecules 1 and 2, however, did not yield consistent IC₅₀ values across replicates, which could be attributed to several experimental factors. Possible reasons for this inconsistency include poor solubility of the compounds in the assay buffer, inadequate interaction between the molecules and the enzyme, or potential interference from DMSO in the assay environment. These experimental variables may have impacted the reproducibility and accuracy of the observed inhibition, leading to unreliable IC₅₀ values for these molecules.

**TABLE 6:** IC₅₀ values of tested molecules.

Molecule	IC_50_ (µM)
1	2.08E-197 ± 0.022
2	3.71E+16 ± 0.021
3	58.41 ± 0.033
4	29.22 ± 0.016
5	40.59 ± 0.015
6	41.60 ± 0.017
7	26.13 ± 0.014
8	24.85 ± 0.019
9	9.98 ± 0.021
Control	41.60 ± 0.011

Data are expressed as the means ± standard deviation (SD) of at least three independent experiments.

## 4 Conclusion

The intricate process of identifying potent human acetylcholinesterase enzyme inhibitors relies on the seamless integration of computational and experimental methodologies. A key initial step was the clustering analysis of 4,643 inhibitors from the binding database, which played a crucial role in organizing and managing the extensive dataset. This analysis categorized the inhibitors into 70 distinct clusters based on their molecular structures, significantly reducing computational complexity. Refining these clusters further to focus on those with higher concentrations of molecules and lower IC₅₀ values proved essential. This refinement, which resulted in nine significant clusters, underscored the importance of balancing computational efficiency with result reliability, ensuring that the most promising inhibitors were prioritized for further analysis.

Furthermore, combining the ensemble docking process with the innovative YN1 metric provided a sophisticated means to rank inhibitors by integrating both docking scores and experimental IC₅₀ values. This approach addressed the limitations of relying solely on computational predictions, which can sometimes overlook critical biological nuances. The YN1 score, ranging from 0 to 2, effectively balanced ligand efficiency and IC₅₀ values, offering a comprehensive assessment of a compound’s inhibitory potential. The observed correlation between lower log (IC₅₀) values and higher YN1 scores validated this metric’s utility. This dual-faceted ranking method successfully identified compounds with both high computational and experimental inhibitory activity, demonstrating the enhanced reliability of integrating diverse data sources.

Moreover, developing and validating ML models further strengthened the screening process. These models were trained on the refined set of active compounds and incorporated extensive decoy generation to ensure high specificity in distinguishing active from inactive compounds. The selection of the best-performing algorithm for each inhibitor family based on a comprehensive objective function ensured that the models were robust and precise. This integration of ML models into the screening pipeline not only enhanced efficiency but also improved the optimization of computational costs for the identification of viable huAChE inhibitors.

Additionally, the use of pharmacophore model ensembles provided a good metrics, showcasing their high capability to identify active molecules. The consideration of stable and unstable conformations to account for the flexibility of the protein’s active site resulted in a improved performance compared to ligand-based models. We believe that the application of model ensembles could emerge as a powerful technique for virtual screening processes, especially when dealing with large numbers of molecules.

The experimental validation further strengthened these findings. Seven of the nine molecules tested showed strong inhibitory activity against huAChE, with molecules 4, 5, 6, 7, 8, and 9 demonstrating IC₅₀ values superior to the control, galantamine. It is noteworthy that the observed inhibitory capacities may be underestimated due to partial solubility of these molecules, suggesting that their effective concentrations could be even lower, potentially enhancing their IC₅₀ values. In contrast, molecules 1 and 2 exhibited inconsistent results, likely due to solubility issues in DMSO, which could be resolved by exploring alternative solvents in future studies.

Finally, the successful identification of promising inhibitors through the integrated approach of virtual screening, experimental validation, and advanced ML algorithms represents a significant achievement. This comprehensive methodology not only accelerates the identification of viable huAChE inhibitors but also establishes a reliable and efficient framework for drug discovery. The findings from this research open the door to designing new molecules with high affinity for huAChE, potentially paving the way for novel treatments for Alzheimer’s disease. We believe that this study sets a precedent for future virtual screening efforts, combining computational, experimental, and machine learning approaches to discover potent enzyme inhibitors.

## Data Availability

The data that support the findings of this study are openly available in ZENODO: Supplementary Data 1: https://zenodo.org/doi/10.5281/zenodo.11402870; Supplementary Data 2: https://zenodo.org/doi/10.5281/zenodo.11402907.
