# Supplementary material for: dyphAI dynamic pharmacophore modeling with AI: a tool for efficient screening of new acetylcholinesterase inhibitors
Source: Front Chem. 2025 Feb 4;13:1479763. doi: 10.3389/fchem.2025.1479763 (PMC11865752; doi:10.3389/fchem.2025.1479763)
Supplement: Supplementary file 1 [file DataSheet1.pdf]

Supplementary information

dyphAI dynamic pharmacophore modeling with AI: a tool for efficient screening of new acetylcholinesterase inhibitors

Yasser Hayek-Orduz[1*†], Dorian Armando Acevedo-Castro[1,2*†], Juan Sebastián Saldarriaga Escobar[3], Brandon Eli Ortiz-Domínguez[3], María Francisca Villegas-Torres[4], Paola A. Caicedo[3], Álvaro Barrera-Ocampo[5], Natalie Cortes[6], Edison H. Osorio[6] and Andrés Fernando González Barrios[1*]

[1]Grupo de Diseño de Productos y Procesos (GDPP), Department of Chemical and Food Engineering, Universidad de los Andes, Bogotá, Colombia
[2]Computational Bio-Organic Chemistry (COBO), Department of Chemistry, Universidad de los Andes, Bogotá, Colombia
[3]Grupo Natura, Facultad de Ingenieria, Diseño y Ciencias Aplicadas, Departamento de Ciencias Biológicas, Bioprocesos y Biotecnología, Universidad ICESI, Cali, Colombia
[4]Centro de Investigaciones Microbiológicas (CIMIC), Department of Biological Sciences, Universidad de los Andes, Bogotá, Colombia
[5]Grupo Natura, Facultad de Ingeniería, Diseño y Ciencias Aplicadas, Departamento de Ciencias Farmacéuticas y Químicas, Universidad ICESI, Cali, Colombia
[6]Grupo de Investigación en Química Bioorgánica y Sistemas Moleculares (QBOSMO), Faculty of Natural Sciences and Mathematics, Universidad de Ibagué, Ibagué, Colombia
[†]These authors contributed equally

***Correspondence:**

Andrés Fernando González Barrios, andgonza@uniandes.edu.co

Yasser Hayek-Orduz, y.hayek10@uniandes.edu.co

Dorian Armando Acevedo-Castro, da.acevedo10@uniandes.edu.co

Table of contents

		Page
**Figure S1.**	Summary of the methods to generate ML and pharmacophore models for AChE inhibitors.	1
**Figure S2.**	Scheme of the procedure used for virtual screening of databases.	**2**
**Figure S3.**	Detailed workflow diagram for the generation of an affinity ranking based on the dynamism of the biological system and experimental data.	**3**
**Figure S4.**	Detailed workflow diagram for the generation of machine learning models to encounter active molecules.	**4**
**Figure S5.**	Detailed workflow diagram for the generation of ligand-based pharmacophore models to encounter active molecules.	**5**
**Figure S6.**	Detailed workflow diagram for the generation of ensembles of complex-based pharmacophore models to encounter active molecules.	**6**
**Figure S7.**	Heatmap indicating the all-atom RMSD values for Tyr-341 of 153 paired combinations originated from the 18 protein crystals.	**8**
**Figure S8.**	Detailed workflow diagram for RMSD paired calculations.	**9**
**Table S1.**	Residues located 5 Å distal to the center of mass of the ligand for 18 protein crystals.	**10**
**Figure S9.**	Kelley penalty vs the number of clusters for the similarity clustering of 4643 inhibitors with an IC50 between 1 and 99000 nM against the huAChE enzyme from Binding Database.	**11**
**Table S2.**	Size, average IC50, standard deviation IC50, and variation coefficient for clusters that satisfied at least 3 conditions. Properties that surpassed the threshold are highlighted in blue. Selected clusters are highlighted in red.	**11**
**Table S3.**	Representative compounds (centroids) of the 9 selected families.	**12**
**Figure S10.**	Comparison of the best poses produced by the induced-fit docking of galantamine and the experimental pose of galantamine.	**13**
**Figure S11.**	2D Interaction diagram and pose produced by the induced-fit docking of acetylcholine.	**13**
**Figure S12.**	RMSD graphs of backbone atoms of 50 ns MD simulations for active site residues.	**14**
**Table S4.**	RMSD Average of 21 residues involved in RMSD paired calculations of 7 conformations for each cluster.	**15**
**Table S5.**	Residues that presented the highest conformational diversity after RMSD paired calculations for the 7 conformations extracted from the 50 ns MD simulations.	**16**
**Figure S13.**	Conformational changes of some active site residues through the course of molecular dynamics for C4 and C20 representative ligands.	**16**

**Table S6.**	Simulation time (nanoseconds) of the frames belonging to the average RMSD, average RMSD plus twice the RMSD standard deviation, average RMSD minus twice the RMSD standard deviation, the two smallest RMSD values, and the two largest RMSD values.	**17**
**Figure S14.**	logIC50 vs Yasser's Number 1 (YN1) score of the ensemble docking calculations for 31, 4, 19, 50, 35, 20, 36, 42, and 23 clusters.	**18**
**Figure S15.**	XP GScore vs Yasser's Number 1 (YN1) score of the ensemble docking calculations for 31, 4, 19, 50, 35, 20, 36, 42, and 23 clusters.	**19**
**Table S7.**	Ligands that displayed best experimental inhibition and docking scores from the ensemble docking procedure for 31, 4, 19, 50, 35, 20, 36, 42, and 23 families.	**20**
**Table S8.**	Training and testing results of C4 Machine Learning models.	**21**
**Table S9.**	Training and testing results of C19 Machine Learning models.	**22**
**Table S10.**	Training and testing results of C20 Machine Learning models.	**23**
**Table S11.**	Training and testing results of C23 Machine Learning models.	**24**
**Table S12.**	Training and testing results of C31 Machine Learning models.	**25**
**Table S13.**	Training and testing results of C35 Machine Learning models.	**26**
**Table S14.**	Training and testing results of C36 Machine Learning models.	**27**
**Table S15.**	Training and testing results of C42 Machine Learning models.	**28**
**Table S16.**	Training and testing results of C50 Machine Learning models.	**29**
**Figure S16.**	ROC curve for best ligand-based pharmacophore models.	**30**
**Figure S17.**	Example of geometry clustering	**31**
**Figure S18.**	Kelley penalty graphs for pose clustering from the ensemble docking calculations for 4, 19, 20, 23, 31, and 35 compounds.	**32**
**Figure S19.**	Kelley penalty graphs for pose clustering from the ensemble docking calculations for 36, 42, and 50 compounds.	**33**
**Figure S20.**	RMSD graphs of backbone atoms of 500 ns MD simulations for the whole protein.	**34**
**Figure S21.**	RMSD graphs of backbone atoms of 500 ns MD simulations for the active site residues.	**35**
**Figure S22.**	RMSD graphs of 500 ns MD simulations for the ligand heavy atoms.	**36**
**Figure S23.**	RMSF values for backbone of 500 ns MD simulations for C4, C19, C20, C23, C31 and C35.	**37**
**Figure S24.**	RMSF values for backbone of 500 ns MD simulations for C36, C42 and C50.	**38**
**Figure S25.**	RMSF values for all heavy atoms (backbone and sidechain) of 500 ns MD simulations for C4, C19, C20, C23, C31 and C35.	**39**
**Figure S26.**	RMSF values for all heavy atoms (backbone and sidechain) of 500 ns MD simulations for C36, C42 and C50.	**40**
**Figure S27.**	RMSF backbone values for 500 ns MD simulation applied on huAChE ribbons representation for C4, C19, C20, C23, C31, C35, C36, and C42 simulations.	**41**
**Figure S28.**	RMSF backbone values for 500 ns MD simulation applied on huAChE ribbons representation for C50 simulation.	**42**
**Figure S29.**	Protein-ligand interaction diagrams with the percentage frequency of interaction occurrences.	**43**
**Figure S30.**	Ligand Hydrogen bond occupancy (%) heatmap for 500 ns MD simulations of C4, C19, C20, C23, and C31.	**44**
**Figure S31.**	Ligand Hydrogen bond occupancy (%) heatmap for 500 ns MD simulations of C35, C36, C42, and C50.	**45**
**Figure S32.**	Protein-ligand contacts heatmap of all residues for 500 ns MD simulation of C4.	**46**
**Figure S33.**	Protein-ligand contacts heatmap of all residues for 500 ns MD simulation of C19.	**47**
**Figure S34.**	Protein-ligand contacts heatmap of all residues for 500 ns MD simulation of C20.	**48**
**Figure S35.**	Protein-ligand contacts heatmap of all residues for 500 ns MD simulation of C23.	**49**
**Figure S36.**	Protein-ligand contacts heatmap of all residues for 500 ns MD simulation of C31.	**50**
**Figure S37.**	Protein-ligand contacts heatmap of all residues for 500 ns MD simulation of C35.	**51**
**Figure S38.**	Protein-ligand contacts heatmap of all residues for 500 ns MD simulation of C36.	**52**
**Figure S39.**	Protein-ligand contacts heatmap of all residues for 500 ns MD simulation of C42.	**53**
**Figure S40.**	Protein-ligand contacts heatmap of all residues for 500 ns MD simulation of C50.	**54**
**Figure S41.**	Protein-ligand contacts heatmap for residues that were within 5 angstroms of the ligand at some point during the simulation of 500 ns MD simulation of C4.	**55**
**Figure S42.**	Protein-ligand contacts heatmap for residues that were within 5 angstroms of the ligand at some point during the simulation of 500 ns MD simulation of C19.	**56**
**Figure S43.**	Protein-ligand contacts heatmap for residues that were within 5 angstroms of the ligand at some point during the simulation of 500 ns MD simulation of C20.	**57**
**Figure S44.**	Protein-ligand contacts heatmap for residues that were within 5 angstroms of the ligand at some point during the simulation of 500 ns MD simulation of C23.	**58**
**Figure S45.**	Protein-ligand contacts heatmap for residues that were within 5 angstroms of the ligand at some point during the simulation of 500 ns MD simulation of C31.	**59**
**Figure S46.**	Protein-ligand contacts heatmap for residues that were within 5 angstroms of the ligand at some point during the simulation of 500 ns MD simulation of C35.	**60**

**Figure S47.**	Protein-ligand contacts heatmap for residues that were within 5 angstroms of the ligand at some point during the simulation of 500 ns MD simulation of C36.	**61**
**Figure S48.**	Protein-ligand contacts heatmap for residues that were within 5 angstroms of the ligand at some point during the simulation of 500 ns MD simulation of C42.	**62**
**Figure S49.**	Protein-ligand contacts heatmap for residues that were within 5 angstroms of the ligand at some point during the simulation of 500 ns MD simulation of C50.	**63**
**Table S17.**	Heavy atoms RMSF values of the 6 active site residues with highest flexibility.	**64**
**Figure S50.**	Heavy atoms RMSD for the 6 active site residues, backbone RMSD for the whole protein, backbone RMSD for the 17 active site residues and RMSD of ligand heavy atoms for C4.	**65**
**Figure S51.**	Heavy atoms RMSD for the 6 active site residues, backbone RMSD for the whole protein, backbone RMSD for the 17 active site residues and RMSD of ligand heavy atoms for C19.	**66**
**Figure S52.**	Heavy atoms RMSD for the 6 active site residues, backbone RMSD for the whole protein, backbone RMSD for the 17 active site residues and RMSD of ligand heavy atoms for C20.	**67**
**Figure S53.**	Heavy atoms RMSD for the 6 active site residues, backbone RMSD for the whole protein, backbone RMSD for the 17 active site residues and RMSD of ligand heavy atoms for C23.	**68**
**Figure S54.**	Heavy atoms RMSD for the 6 active site residues, backbone RMSD for the whole protein, backbone RMSD for the 17 active site residues and RMSD of ligand heavy atoms for C31.	**69**
**Figure S55.**	Heavy atoms RMSD for the 6 active site residues, backbone RMSD for the whole protein, backbone RMSD for the 17 active site residues and RMSD of ligand heavy atoms for C35.	**70**
**Figure S56.**	Heavy atoms RMSD for the 6 active site residues, backbone RMSD for the whole protein, backbone RMSD for the 17 active site residues and RMSD of ligand heavy atoms for C36.	**71**
**Figure S57.**	Heavy atoms RMSD for the 6 active site residues, backbone RMSD for the whole protein, backbone RMSD for the 17 active site residues and RMSD of ligand heavy atoms for C42.	**72**
**Figure S58.**	Heavy atoms RMSD for the 6 active site residues, backbone RMSD for the whole protein, backbone RMSD for the 17 active site residues and RMSD of ligand heavy atoms for C50.	**73**
**Figure S59.**	(A) RMSD Normalized moving average for the 9 data sets (Heavy atoms RMSD for the 6 active site residues, backbone RMSD for the whole protein, backbone RMSD for the 17 active site residues, and RMSD of ligand heavy atoms. (B) Mean of RMSD Normalized moving average for the 9 data sets. (C) Numerical derivative of RMSD Normalized moving average. The plots belong to C4.	**74**
**Figure S60.**	(A) RMSD Normalized moving average for the 9 data sets (Heavy atoms RMSD for the 6 active site residues, backbone RMSD for the whole protein, backbone RMSD for the 17 active site residues, and RMSD of ligand heavy atoms. (B) Mean of RMSD Normalized moving average for the 9 data sets. (C) Numerical derivative of RMSD Normalized moving average. The plots belong to C19.	**75**
**Figure S61.**	(A) RMSD Normalized moving average for the 9 data sets (Heavy atoms RMSD for the 6 active site residues, backbone RMSD for the whole protein, backbone RMSD for the 17 active site residues, and RMSD of ligand heavy atoms. (B) Mean of RMSD Normalized moving average for the 9 data sets. (C) Numerical derivative of RMSD Normalized moving average. The plots belong to C20.	**76**
**Figure S62.**	(A) RMSD Normalized moving average for the 9 data sets (Heavy atoms RMSD for the 6 active site residues, backbone RMSD for the whole protein, backbone RMSD for the 17 active site residues, and RMSD of ligand heavy atoms. (B) Mean of RMSD Normalized moving average for the 9 data sets. (C) Numerical derivative of RMSD Normalized moving average. The plots belong to C23.	**77**
**Figure S63.**	(A) RMSD Normalized moving average for the 9 data sets (Heavy atoms RMSD for the 6 active site residues, backbone RMSD for the whole protein, backbone RMSD for the 17 active site residues, and RMSD of ligand heavy atoms. (B) Mean of RMSD Normalized moving average for the 9 data sets. (C) Numerical derivative of RMSD Normalized moving average. The plots belong to C31.	**78**
**Figure S64.**	(A) RMSD Normalized moving average for the 9 data sets (Heavy atoms RMSD for the 6 active site residues, backbone RMSD for the whole protein, backbone RMSD for the 17 active site residues, and RMSD of ligand heavy atoms. (B) Mean of RMSD Normalized moving average for the 9 data sets. (C) Numerical derivative of RMSD Normalized moving average. The plots belong to C35.	**79**
**Figure S65.**	(A) RMSD Normalized moving average for the 9 data sets (Heavy atoms RMSD for the 6 active site residues, backbone RMSD for the whole protein, backbone RMSD for the 17 active site residues, and RMSD of ligand heavy atoms. (B) Mean of RMSD Normalized moving average for the 9 data sets. (C) Numerical derivative of RMSD Normalized moving average. The plots belong to C36.	**80**
**Figure S66.**	(A) RMSD Normalized moving average for the 9 data sets (Heavy atoms RMSD for the 6 active site residues, backbone RMSD for the whole protein, backbone RMSD for the 17 active site residues, and RMSD of ligand heavy atoms. (B) Mean of RMSD Normalized moving average for the 9 data sets. (C) Numerical derivative of RMSD Normalized moving average. The plots belong to C42.	**81**
**Figure S67.**	(A) RMSD Normalized moving average for the 9 data sets (Heavy atoms RMSD for the 6 active site residues, backbone RMSD for the whole protein, backbone RMSD for the 17 active site residues, and RMSD of ligand heavy atoms. (B) Mean of RMSD Normalized moving average for the 9 data sets. (C) Numerical derivative of RMSD Normalized moving average. The plots belong to C50.	**82**
**Figure S68.**	(A) Simulation times where the value of the numerical derivative is higher than 0.6. (B) Simulation times where the RMSD Normalized moving average is higher than 0.6. (C) Simulation times where the RMSD Normalized moving average and the numerical derivative are higher than 0.6. The plots belong to C4.	**83**
**Figure S69.**	(A) Simulation times where the value of the numerical derivative is higher than 0.6. (B) Simulation times where the RMSD Normalized moving average is higher than 0.6. (C) Simulation times where the RMSD Normalized moving average and the numerical derivative are higher than 0.6. The plots belong to C19.	**84**
**Figure S70.**	(A) Simulation times where the value of the numerical derivative is higher than 0.6. (B) Simulation times where the RMSD Normalized moving average is higher than 0.6. (C) Simulation times where the RMSD Normalized moving average and the numerical derivative are higher than 0.6. The plots belong to C20.	**85**
**Figure S71.**	(A) Simulation times where the value of the numerical derivative is higher than 0.6. (B) Simulation times where the RMSD Normalized moving average is higher than 0.6. (C) Simulation times where the RMSD Normalized moving average and the numerical derivative are higher than 0.6. The plots belong to C23.	**86**
**Figure S72.**	(A) Simulation times where the value of the numerical derivative is higher than 0.6. (B) Simulation times where the RMSD Normalized moving average is higher than 0.6. (C) Simulation times where the RMSD Normalized moving average and the numerical derivative are higher than 0.6. The plots belong to C31.	**87**

**Figure S73.**	(A) Simulation times where the value of the numerical derivative is higher than 0.6. (B) Simulation times where the RMSD Normalized moving average is higher than 0.6. (C) Simulation times where the RMSD Normalized moving average and the numerical derivative are higher than 0.6. The plots belong to C35.	**88**
**Figure S74.**	(A) Simulation times where the value of the numerical derivative is higher than 0.6. (B) Simulation times where the RMSD Normalized moving average is higher than 0.6. (C) Simulation times where the RMSD Normalized moving average and the numerical derivative are higher than 0.6. The plots belong to C36.	**89**
**Figure S75.**	(A) Simulation times where the value of the numerical derivative is higher than 0.6. (B) Simulation times where the RMSD Normalized moving average is higher than 0.6. (C) Simulation times where the RMSD Normalized moving average and the numerical derivative are higher than 0.6. The plots belong to C42.	**90**
**Figure S76.**	(A) Simulation times where the value of the numerical derivative is higher than 0.6. (B) Simulation times where the RMSD Normalized moving average is higher than 0.6. (C) Simulation times where the RMSD Normalized moving average and the numerical derivative are higher than 0.6. The plots belong to C50.	**91**
**Figure S77.**	TRAPP-pocket results for the unstable zone of 150-180 ns of C4.	**92**
**Figure S78.**	TRAPP-pocket results for the unstable zone of 430-460 ns for C4.	**92**
**Figure S79.**	TRAPP-pocket results for the unstable zone of 60-100 ns for C19.	**93**
**Figure S80.**	TRAPP-pocket results for the unstable zone of 200-220 ns for C19.	**93**
**Figure S81.**	TRAPP-pocket results for the unstable zone of 330-350 ns for C19.	**94**
**Figure S82.**	TRAPP-pocket results for the unstable zone of 350-410 ns for C20.	**94**
**Figure S83.**	TRAPP-pocket results for the unstable zone of 110-140 ns for C23.	**95**
**Figure S84.**	TRAPP-pocket results for the unstable zone of 410-450 ns for C23.	**95**
**Figure S85.**	TRAPP-pocket results for the unstable zone of 90-130 ns for C31.	**96**
**Figure S86.**	TRAPP-pocket results for the unstable zone of 380-410 ns for C31.	**96**
**Figure S87.**	TRAPP-pocket results for the unstable zone of 50-60 ns for C35.	**97**
**Figure S88.**	TRAPP-pocket results for the unstable zone of 210-250 ns for C35.	**97**
**Figure S89.**	TRAPP-pocket results for the unstable zone of 60-100 ns for C36.	**98**
**Figure S90.**	TRAPP-pocket results for the unstable zone of 60-100 ns for C42.	**98**
**Figure S91.**	TRAPP-pocket results for the unstable zone of 50-60 ns for C50.	**99**
**Figure S92.**	TRAPP-pocket results for the unstable zone of 270-280 ns for C50.	**99**
**Figure S93.**	Scheme of a pharmacophore model ensemble.	**100**
**Figure S94.**	ROC curve for the validation of the ensemble pharmacophore models without YN2 application.	**101**
**Table S18.**	Results of validation of pharmacophore model ensembles without YN2 application.	**102**
**Table S19.**	Results of optimization for YN2 threshold for C4.	**103**
**Table S20.**	Results of optimization for YN2 threshold for C19.	**104**
**Table S21.**	Results of optimization for YN2 threshold for C20.	**105**
**Table S22.**	Results of optimization for YN2 threshold for C23.	**106**
**Table S23.**	Results of optimization for YN2 threshold for C31.	**107**
**Table S24.**	Results of optimization for YN2 threshold for C35.	**108**
**Table S25.**	Results of optimization for YN2 threshold for C36.	**109**
**Table S26.**	Results of optimization for YN2 threshold for C42.	**110**
**Table S27.**	Results of optimization for YN2 threshold for C50.	**111**
**Table S28.**	Parameter values of pharmacophore model ensembles with YN2 application.	**111**
**Table S29.**	Results of validation of pharmacophore model ensembles with YN2 application.	**112**
**Figure S95.**	ROC curve for the validation of the ensemble pharmacophore models with YN2 application.	**113**
**Table S30.**	Percentage improvement of recall and specificity for the ensembles using and not using YN2.	**114**
**Figure S96.**	Molecular structures of the two compounds with the lowest MMGBSA binding energy for C4, C19, C20, C23, C31 families from the virtual screening of the ZINC library.	**115**
**Figure S97.**	Molecular structures of the two compounds with the lowest MMGBSA binding energy for C31, C35, C36, C42, C50 families from the virtual screening of the ZINC library.	**116**
**Figure S98.**	2D Interaction diagrams of the two compounds with the lowest MMGBSA binding energy for C4 family from the virtual screening of the ZINC library.	**117**
**Figure S99.**	2D Interaction diagrams of the two compounds with the lowest MMGBSA binding energy for C19 family from the virtual screening of the ZINC library.	**118**
**Figure S100.**	2D Interaction diagrams of the two compounds with the lowest MMGBSA binding energy for C20 family from the virtual screening of the ZINC library.	**119**
**Figure S101.**	2D Interaction diagrams of the two compounds with the lowest MMGBSA binding energy for C23 family from the virtual screening of the ZINC library.	**120**

**Figure S102.**	2D Interaction diagrams of the two compounds with the lowest MMGBSA binding energy for C31 family from the virtual screening of the ZINC library.	**121**
**Figure S103.**	2D Interaction diagrams of the two compounds with the lowest MMGBSA binding energy for C35 family from the virtual screening of the ZINC library.	**122**
**Figure S104.**	2D Interaction diagrams of the two compounds with the lowest MMGBSA binding energy for C36 family from the virtual screening of the ZINC library.	**123**
**Figure S105.**	2D Interaction diagrams of the two compounds with the lowest MMGBSA binding energy for C42 family from the virtual screening of the ZINC library.	**124**
**Figure S106.**	2D Interaction diagrams of the two compounds with the lowest MMGBSA binding energy for C50 family from the virtual screening of the ZINC library.	**125**

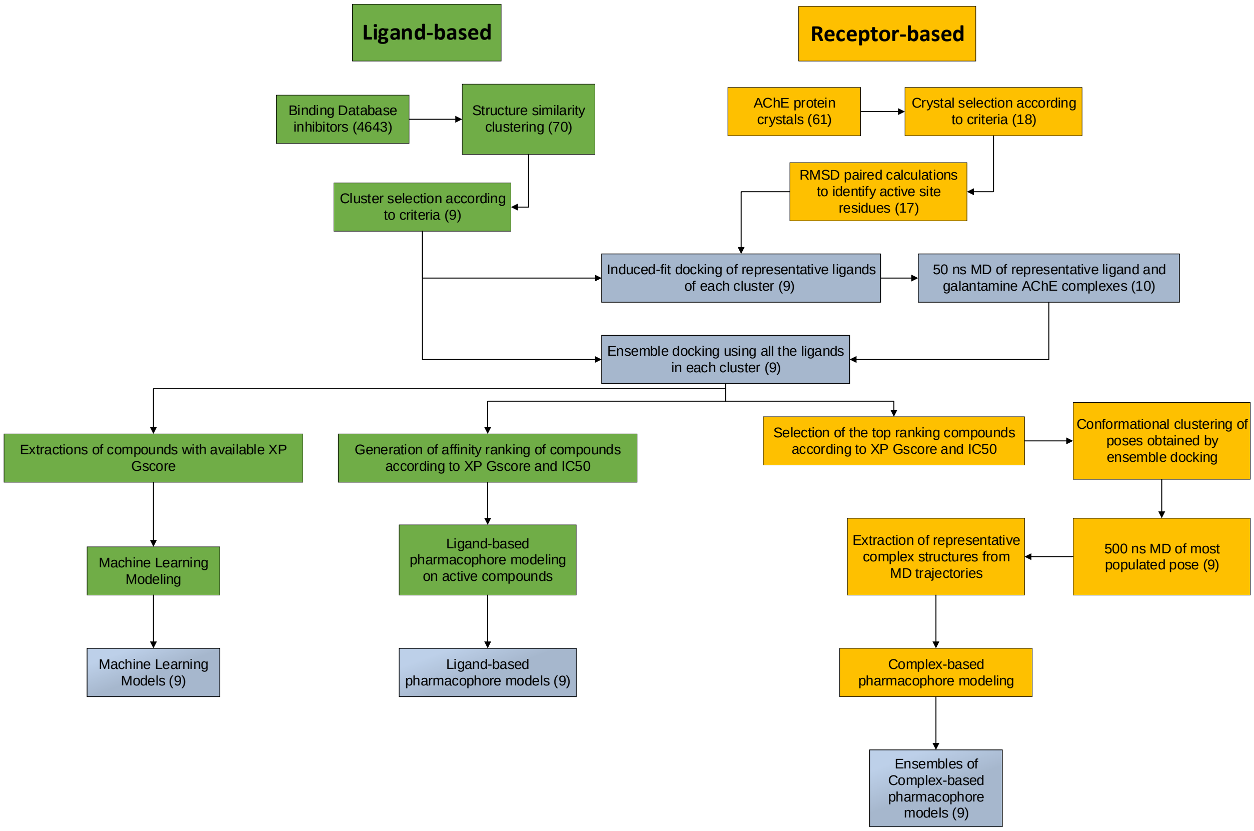

Figure S1. Summary of the methods to generate ML and pharmacophore models for AChE inhibitors. The research protocol was divided into two parts: ligand-based and receptor-based. For the ligand-based approach, 4,643 known AChE inhibitors were downloaded and categorized into 70 clusters or families based on molecular structure. Nine families were selected from these for further analysis. For the receptor-based approach, 61 PDB format crystal structures of huAChE were downloaded and 18 were selected based on certain criteria. A paired combinatorial RMSD study was performed on these 18 structures, identifying 17 flexible residues. The two approaches were then combined using an induced-fit docking approach, in which the representative ligands from each of the nine families were docked into the AChE receptor. Nine 50 ns MD simulations were performed based on the docked poses, along with an additional simulation of the AChE-galantamine complex. Conformations of the protein were extracted from these simulations and used in an ensemble docking approach, in which compounds from each of the nine families were docked separately. The compounds that passed the docking stage through Schrödinger ensemble docking, meaning those that obtained at least a negative XP Gscore, were used for the generation of machine learning models. The ligand-based approach used the resulting docking scores and experimental IC50 values to generate a ranking of affinity, identifying the most active compounds from each family. These compounds were used to generate ligand-based pharmacophore models. For the receptor-based approach, the most active compound from each family was used to extract conformations of the active site through 500 ns MD simulations, RMSD analysis and TRAPP physicochemical calculations. These conformations were used to generate complex-based pharmacophore models and these were employed to create ensembles of pharmacophore models.

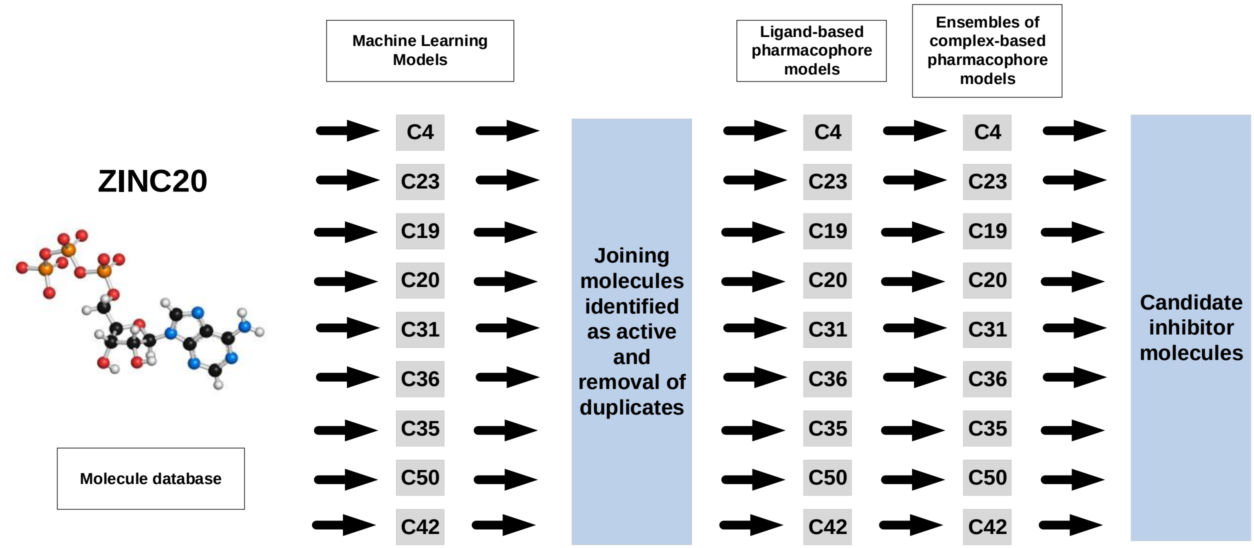

Figure S2. Scheme of the procedure used for virtual screening of databases. The molecules underwent a virtual screening protocol consisting of three stages: machine learning models, ligand-based pharmacophore models, and ensemble of complex-based pharmacophore models. Initially, the database molecules undergo screening using 9 machine learning models, each designed for families 31, 4, 19, 50, 35, 20, 36, 42, and 23. Subsequently, the identified actives from each model are combined, and duplicates are removed. The resulting library then undergoes screening using ligand-based pharmacophore models designed for families 31, 4, 19, 50, 35, 20, 36, 42, and 23. The identified actives then proceed to the ensemble models of pharmacophores corresponding to their respective families.

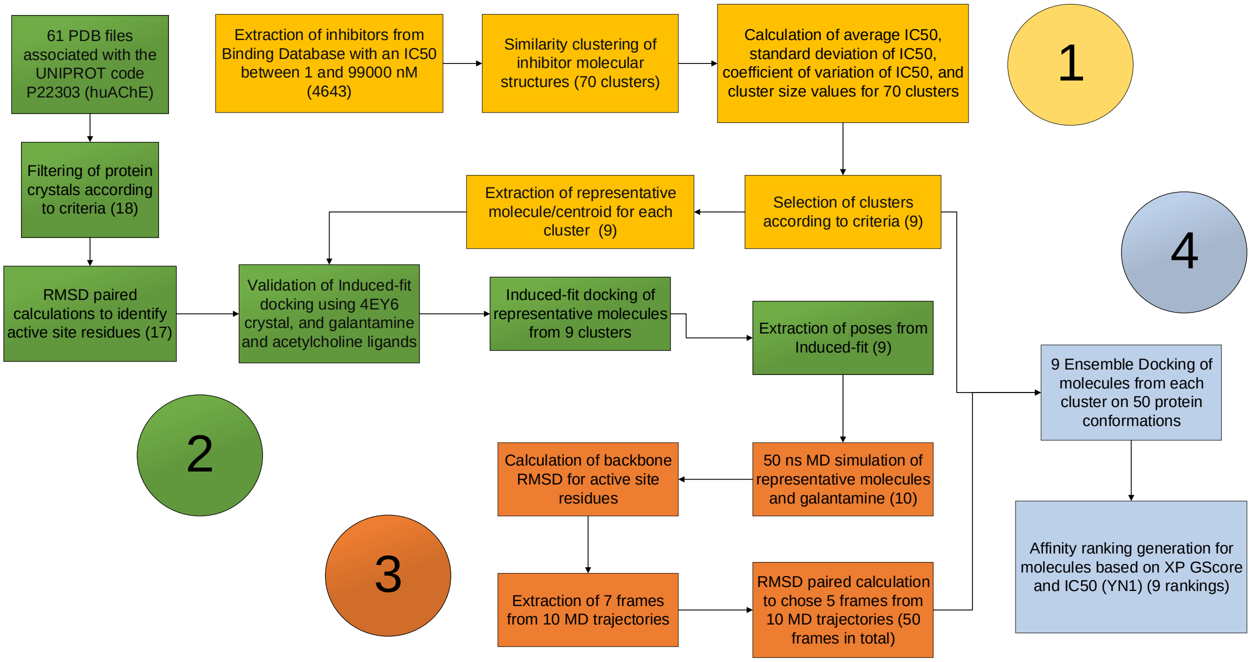

Figure S3. Detailed workflow diagram for the generation of an affinity ranking based on the dynamism of the biological system and experimental data. (1) Similarity clustering of inhibitor structures, (2) Induced-fit docking of representative molecules from inhibitor clusters, (3) 50 ns MD Simulation of protein-ligand complexes of representative molecules from clusters of human acetylcholinesterase inhibitors and conformational sampling, and (4) Ensemble docking and affinity ranking.

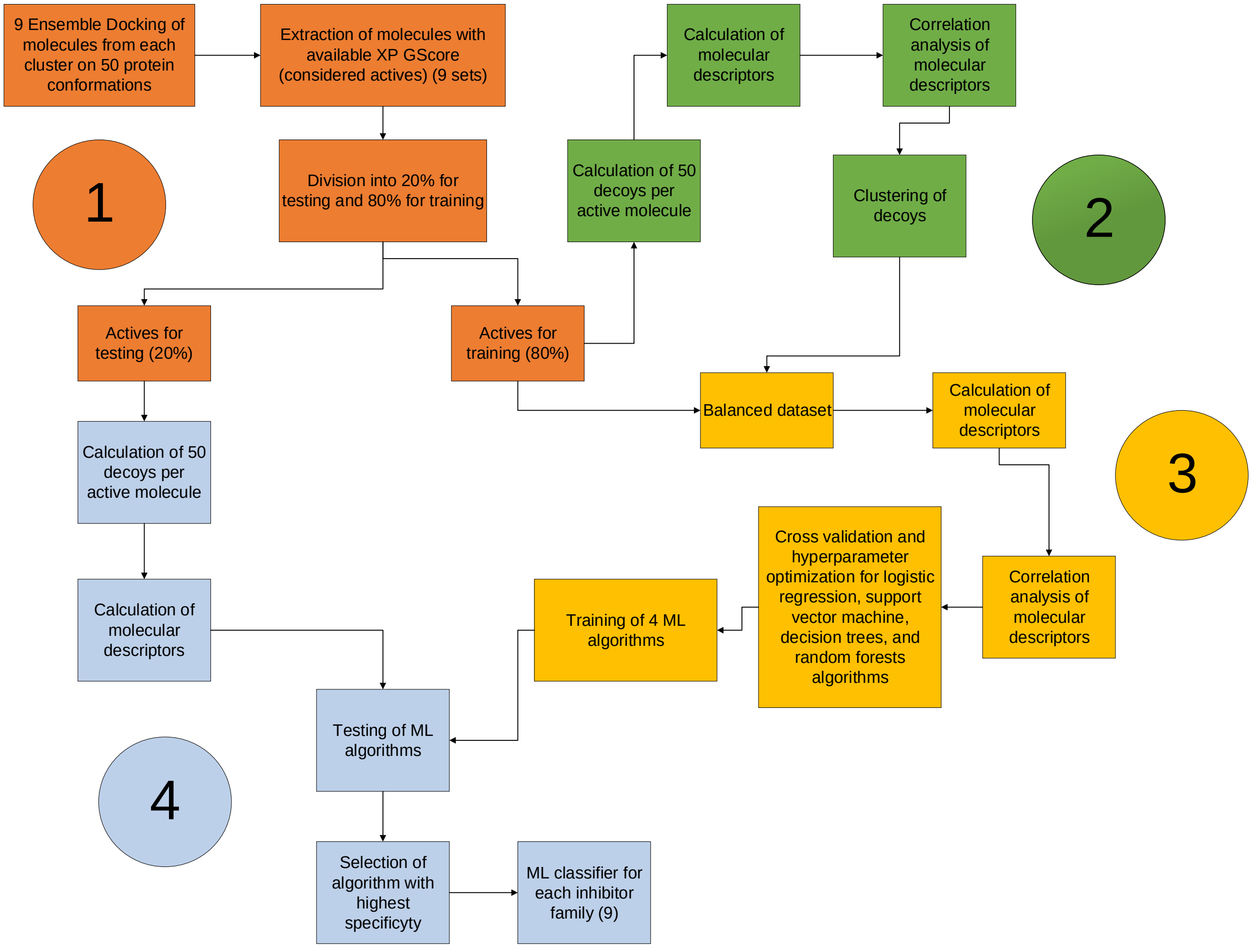

Figure S4. Detailed workflow diagram for the generation of machine learning models to encounter active molecules. (1) Data Splitting for Training and Testing, (2) Clustering of Molecular Descriptors for Decoys from the Training Library, (3) Training Machine Learning Models, and (4) Testing Machine Learning Models.

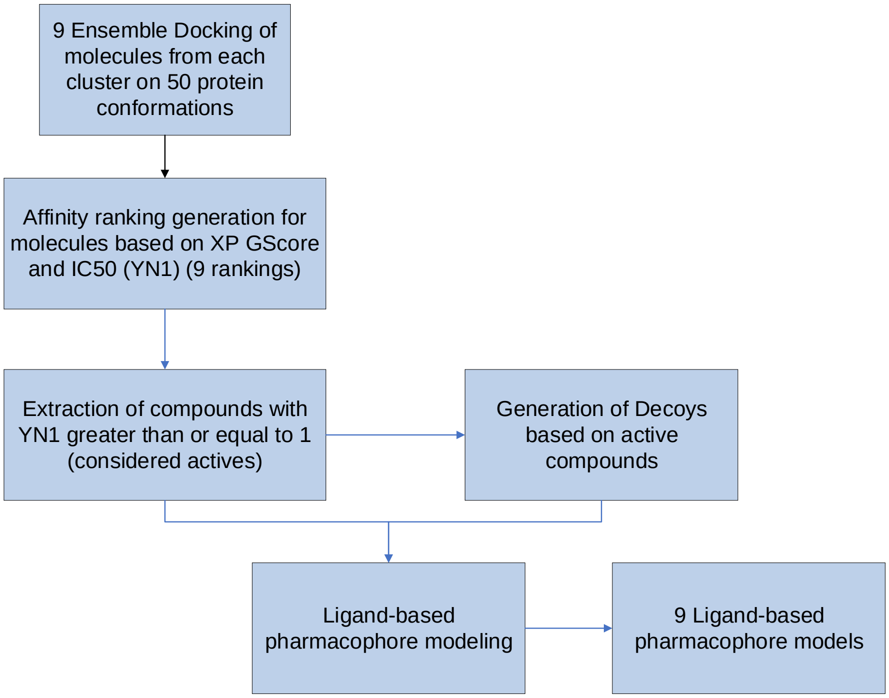

Figure S5. Detailed workflow diagram for the generation of ligand-based pharmacophore models to encounter active molecules.

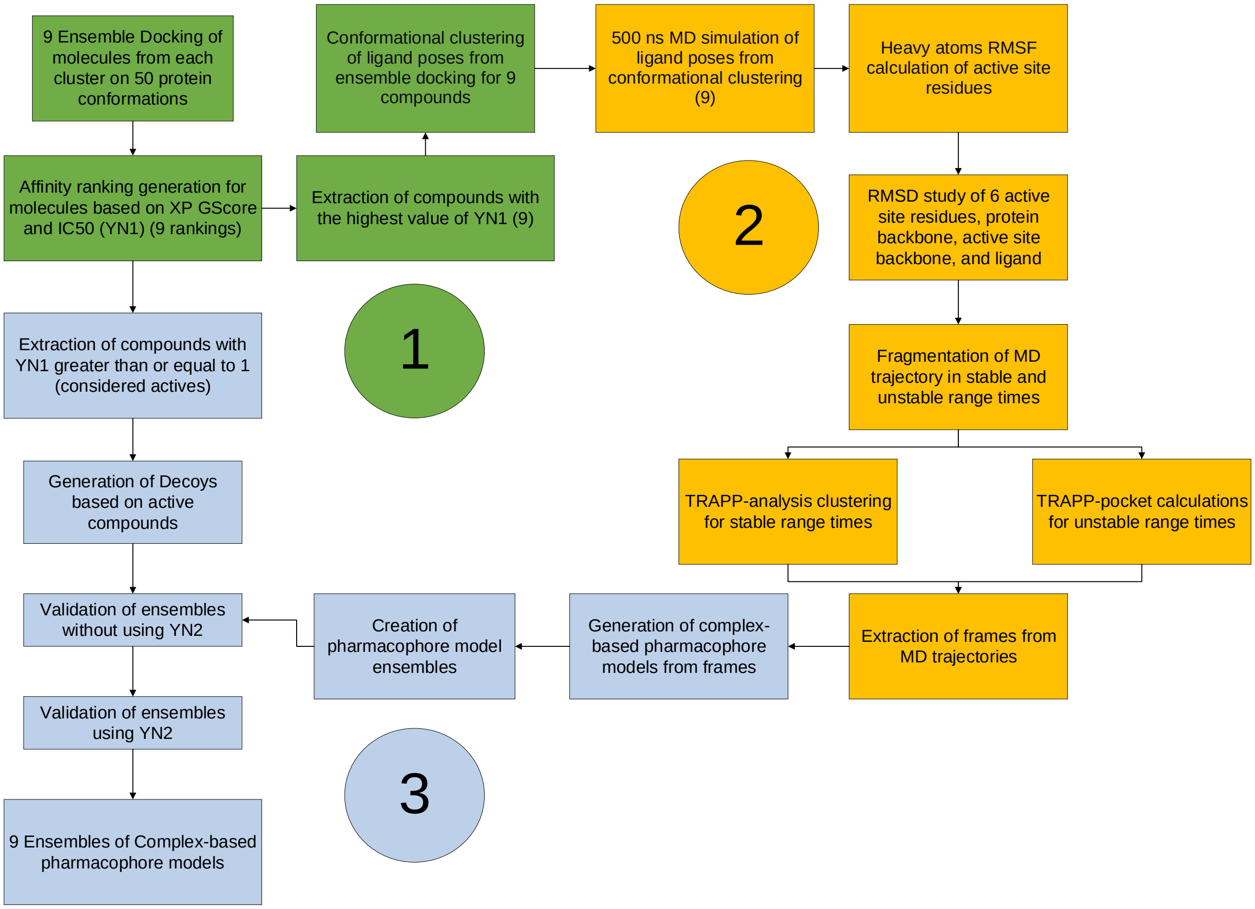

Figure S6. Detailed workflow diagram for the generation of ensembles of complex-based pharmacophore models to encounter active molecules. (1) Conformational clustering of ligand poses from ensemble docking, (2) TRAPP calculations and extraction of active site conformations from 500 ns MD simulations, and (3) complex-based pharmacophore modeling.

General procedure of MD simulations

The following molecular dynamics procedure was consistent throughout this investigation, with only changes made to the production time. The ligand poses contained in the protein-ligand complexes were extracted. These ligand structures were used for electronic energy minimization using the RHF/6-31G(d) chemical model. The minimized geometries were utilized for a restricted electrostatic potential (RESP) calculation using a Merz-Singh-Kollman (MK) scheme. All quantum mechanical calculations were performed in the gas phase, with singlet multiplicity and using Gaussian 16 software. The RESP calculation was used for ligand parameterization employing the GAFF force field via AmberTools22[1]. Protein atoms were parameterized using the ff14SB force field and ligand atoms with the GAFF force field[2]. In addition, the acpype.py script was used to convert the files to Gromacs format[3].

Protein-ligand complexes were solvated using a cubic box with a distance of 1.5 nm. Water molecules were parameterized using the TIP3P model. The net charge of the system was neutralized using sodium and chloride ions with a NaCl concentration of 0.15 M. The integrator algorithm used was leap-frog. The preparation of the system was performed in three main steps: energy minimization, heating, and equilibration. Energy minimization was performed in two stages: i) minimization of solvent atoms, ions, and hydrogen atoms, and ii) minimization of all atoms. For the minimization, the steepest descent algorithm was used with a step of 0.01 nm, and restraint with a force constant of 10 kcal/molÅ^2. The heating was performed in three stages:

i) NVT 0 to 100 K restraining heavy backbone atoms and ligand with a force constant of 10 kcal/molÅ^2. Time step of 1 fs and a simulation time of 63 ps.

ii) NVT 100 to 200 K restraining heavy backbone atoms and ligand with a force constant of 10 kcal/molÅ^2. Time step of 1 fs and a simulation time of 63 ps.

iii) NVT 200 to 310 K restraining heavy backbone atoms and ligand with a force constant of 5 kcal/molÅ^2. Time step of 1 fs and a simulation time of 63 ps.

Initial random atomic velocities were obtained using Maxwell-Boltzmann distribution with a temperature of 310 K. The equilibration of the system was carried out in six steps:

i) NPT at 310 K restraining backbone and ligand with a force constant of 5 kcal/molÅ^2. Time step of 1 fs and a simulation time of 2 ns.

ii) NPT at 310 K restraining backbone with a force constant of 3 kcal/molÅ^2 and ligand with 5 kcal/molÅ^2. Time step of 1 fs and a simulation time of 2 ns.

iii) NPT at 310 K restraining backbone with a force constant of 1 kcal/molÅ^2 and ligand with 5 kcal/molÅ^2. Time step of 1 fs and a simulation time of 2 ns.

iv) NPT at 310K restraining backbone with a force constant of 1 kcal/molÅ^2 and ligand with 3 kcal/molÅ^2. Time step of 1 fs and a simulation time of 2 ns.

v) NPT at 310 K restraining backbone with a force constant of 1 kcal/molÅ^2 and ligand with 1 kcal/molÅ^2. Time step of 1 fs and a simulation time of 2 ns.

vi) NPT at 310 K restraining backbone and ligand with a force constant of 0.1 kcal/molÅ^2. Time step of 2 fs and a simulation time of 20 ns.

Electrostatic interactions were modeled with the Particle Mesh Ewald (PME) method using a Fourier space of 0.125 and a cutoff of 1 nm. Finally, the production run of the systems was performed using the Berendsen-modified thermostat and the Parrinello-Rahman barostat. All simulations were run in the HYPATIA cluster from Universidad de Los Andes and the TITAN workstation.

RMSD paired calculations

We refer to a paired RMSD calculation as a computation that calculates the RMSD corresponding to a residue of interest for various crystal pairs. The result is a heatmap in which crystals are represented on the x-axis and y-axis, and their respective RMSD values are depicted on the heat map. Below is an example.

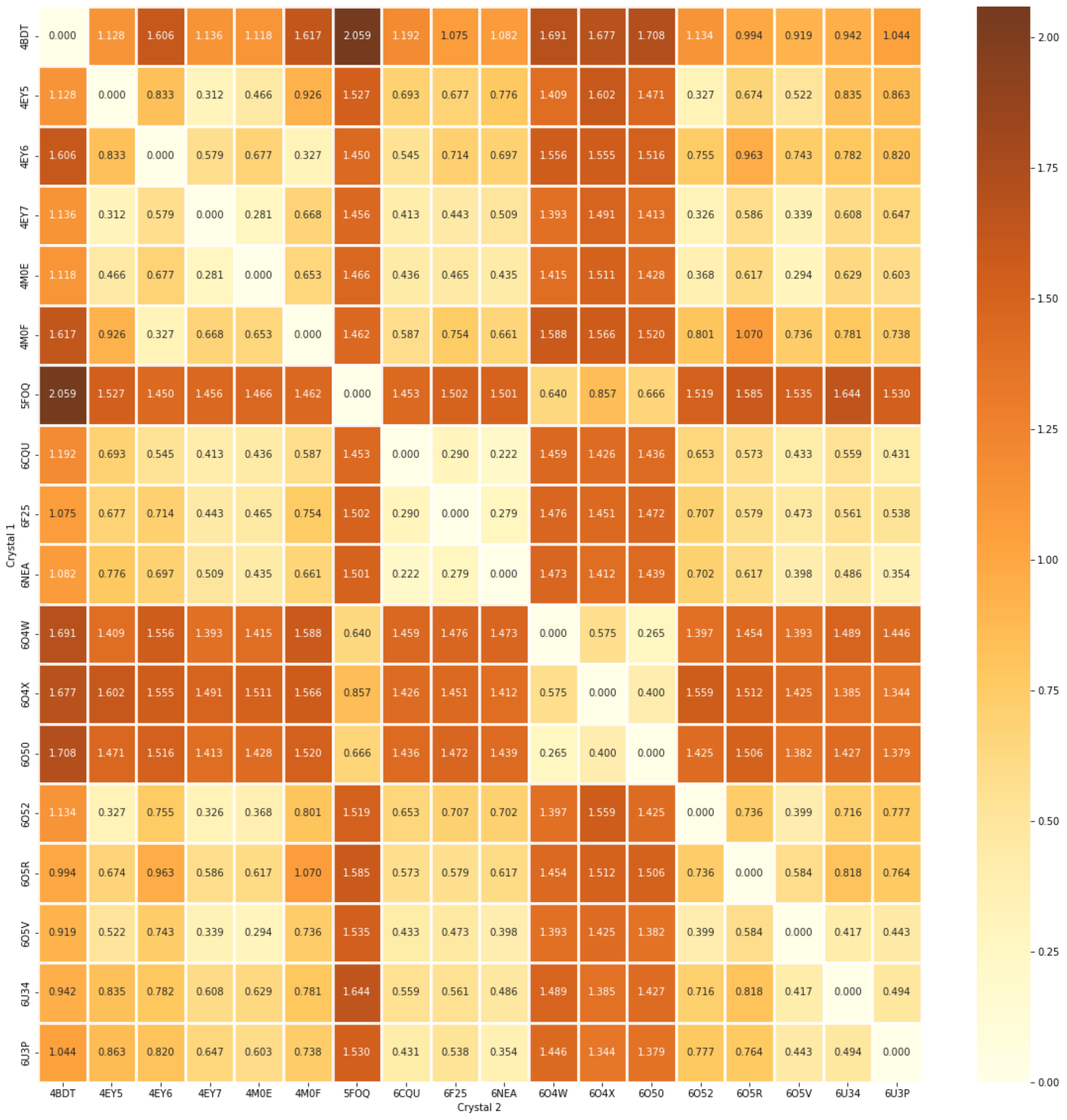

Figure S7. Heatmap indicating the all-atom RMSD values for Tyr-341 of 153 paired combinations originated from the 18 protein crystals.

In order to perform a RMSD paired calculation, we initially sought protein crystals that fulfilled the following criteria: i) resolution of less than 3.2 Å, ii) wild-type enzyme, and iii) contained non-covalent co-crystallized ligands. A total of 18 huAChE protein crystals were selected, specifically identified by their PDB codes: 4BDT, 4EY5, 4EY6, 4EY7, 4M0E, 4M0F, 5FOQ, 6CQU, 6F25, 6NEA, 6O4W, 6O4X, 6O5R, 6O5V, 6O50, 6O52, 6U3P, and 6U34.

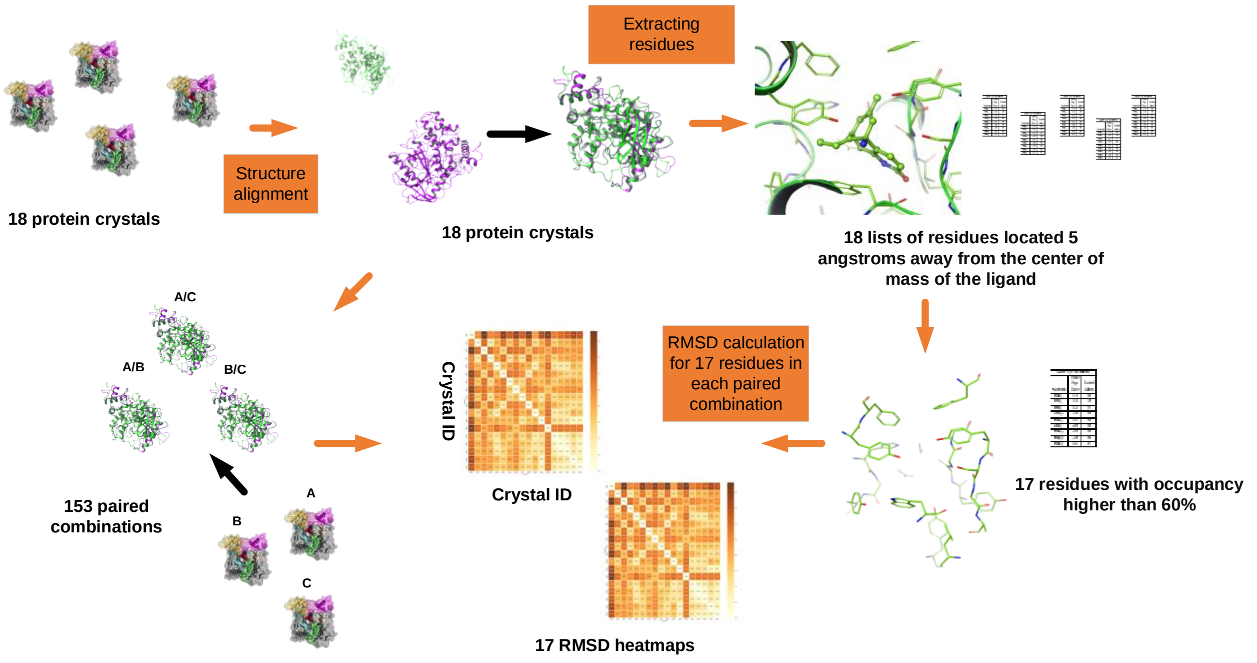

Figure S8. Detailed workflow diagram for RMSD paired calculations.

To perform a paired RMSD calculation, we established three steps: (i) aligning all protein crystals relative to a reference crystal, (ii) extracting residues located 5 Å away from the ligand center of mass for each crystal structure and obtaining residues with an occupancy higher than 60%, and (iii) calculating the RMSD for specific residues (occupancy higher than 60%) found in the second step for each pairwise combination of protein crystals. The number of possible combinations was determined using the formula C(n, 2) = n! / (2! * (n-2)!), where n is the number of crystals. To facilitate this process, we developed four Python scripts (Supplementary Data 1). The Pymol module for Python and the RmsdByResidue Pymol script[4] were employed to conduct the calculations.

Applied to our system, for the first step, the 18 protein crystals were aligned with the PDB 4BDT crystal (selected randomly) as the reference structure. Concerning the second step, to compare residues located 5 Å away from the ligand, we utilized the term "occupancy," which represents the percentage of times a residue is found within 5 Å of the ligand across all the protein crystals. We identified the residues located at 5 Å from the ligands for all 18 crystals (**Table S1**) and then calculated the occupancy for each residue. We found 17 residues with an occupancy of 60% or higher: Tyr-72, Asp-74, Trp-86, Gly-120, Gly-121, Tyr-124, Tyr-133, Glu-202, Ser-203, Trp-286, Phe-295, Phe-297, Tyr-337, Phe-338, Tyr-341, His-447, and Gly-448. Additionally, we identified 4 residues with 100% occupancy: Asp-74, Tyr-124, Tyr-337, and Tyr-341.

We employed the 17 residues identified in the second step to define, in the subsequent stages of the research, which residues constitute the active site. On the other hand, for the third step, we used the same 17 residues to calculate the RMSD heatmaps, which are reported in (Supplementary Data 1).

Table S1. Residues located 5 Å distal to the center of mass of the ligand for 18 protein crystals.

PDB	Residues located 5 Å distal to the center of mass of the ligand
4BDT	82, 86, 120, 121, 122, 124, 125, 133, 202, 203, 204, 295, 297, 337, 338, 341, 439, 443, 446, 447, 448, 449
4EY5	74, 86, 119, 120, 121, 122, 124, 125, 126, 127, 130, 133, 202, 203, 297, 337, 338, 447, 448, 449
4EY6	74, 86, 120, 121, 122, 124, 125, 133, 202, 203, 204, 236, 295, 297, 337, 338, 341, 447, 448, 451
4EY7	72, 74, 86, 120, 121, 124, 133, 202, 203, 286, 289, 293, 294, 295, 296, 297, 337, 338, 341, 447, 448
4M0E	72, 74, 124, 286, 293, 294, 295, 296, 297, 337, 338, 341
4M0F	72, 74, 86, 120, 121, 122, 124, 125, 133, 202, 203, 286, 293, 294, 295, 296, 297, 337, 338, 341, 342, 447, 448
5FOQ	72, 74, 75, 86, 120, 121, 124, 133, 202, 203, 286, 297, 337, 338, 341, 447, 448, 451
6CQU	72, 73, 74, 87, 124, 125, 282, 283, 285, 286, 337, 338, 341
6F25	72, 74, 82, 83, 86, 120, 121, 122, 124, 125, 133, 202, 203, 283, 286, 287, 293, 294, 295, 296, 297, 337, 338, 341, 439, 447
6NEA	72, 73, 74, 87, 124, 125, 282, 283, 285, 286, 337, 338, 341, 447
6O4W	72, 74, 86, 120, 121, 124, 133, 202, 203, 286, 289, 293, 294, 295, 296, 297, 337, 338, 341, 447, 448, 451
6O4X	82, 83, 86, 120, 121, 124, 133, 202, 203, 337, 341, 439, 447, 448, 449, 451
6O50	72, 74, 76, 86, 120, 121, 122, 124, 125, 133, 202, 203, 286, 295, 297, 337, 338, 341, 447, 448, 449, 451
6O52	72, 76, 86, 120, 121, 122, 124, 125, 133, 202, 203, 286, 295, 297, 337, 338, 341, 447, 448, 449
6O5R	72, 74, 124, 286, 293, 294, 295, 296, 297, 337, 338, 341
6O5V	72, 74, 121, 122, 124, 286, 287, 294, 295, 296, 297, 337, 338, 341
6U34	86, 120, 121, 122, 124, 125, 133, 202, 203, 295, 297, 337, 338, 341, 447, 448
6U3P	72, 74, 76, 86, 120, 121, 122, 124, 133, 202, 203, 286, 295, 297, 337, 338, 341, 447, 451

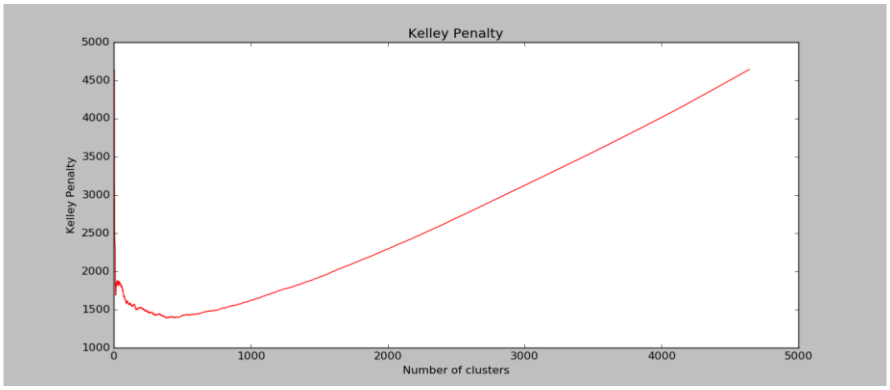

Figure S9. Kelley penalty vs the number of clusters for the similarity clustering of 4643 inhibitors with an IC50 between 1 and 99000 nM against the huAChE enzyme from Binding Database.

Table S2. Size, average IC50, standard deviation IC50, and variation coefficient for clusters that satisfied at least 3 conditions. Properties that surpassed the threshold are highlighted in blue. Selected clusters are highlighted in red.

Cluster ID	Size	Average IC50 (nM)	Standard deviation IC50 (nM)	Variation coefficient
**41**	2	**149.0**	**110.3**	**0.74**
**1**	3	**6750.0**	**444.4**	**0.07**
**25**	3	**9133.3**	**2136.2**	**0.23**
**51**	3	**3303.3**	**4175.8**	**1.26**
**22**	7	**72.3**	**85.3**	**1.18**
**56**	9	**10097.6**	**11234.0**	**1.11**
**60**	10	**102.3**	**43.9**	**0.43**
**46**	24	**9680.3**	**10513.8**	**1.09**
**36**	47	**5655.7**	**4170.6**	**0.74**
**31**	48	**2400.7**	**1245.6**	**0.52**
**4**	**59**	**6032.2**	**7578.1**	**1.26**
**19**	**64**	**2742.2**	**4188.6**	**1.53**
**50**	**68**	**8347.6**	**13442.9**	**1.61**
**35**	**68**	**2819.7**	**8184.2**	2.90
**20**	**261**	**3235.8**	**8610.9**	2.66
**42**	**1038**	**5529.9**	**13652.9**	2.47
**23**	**1455**	**6590.7**	**14219.0**	2.16

Table S3. Representative compounds (centroids) of the 9 selected families.

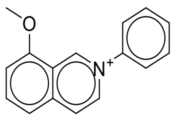	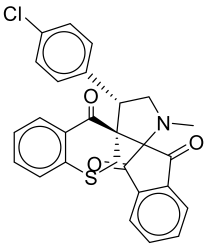	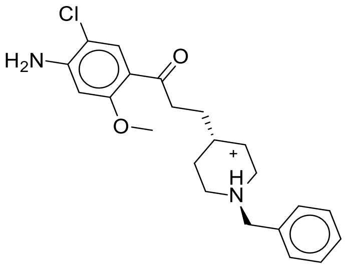
**C-4 (ID 50564847)**	**C-19 (ID 50558519)**	**C-23 (ID 50079370)**
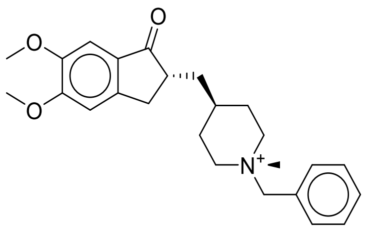	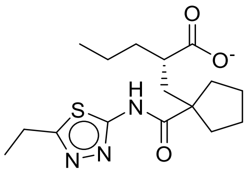	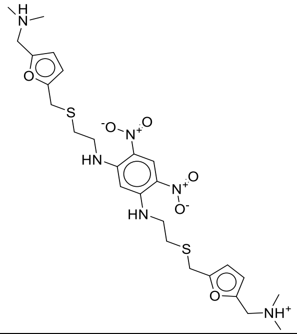
**C-20 (ID 50117583)**	**C-31 (ID 50190736)**	**C-35 (ID 50005500)**
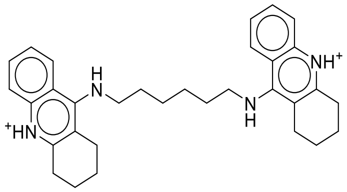	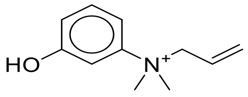	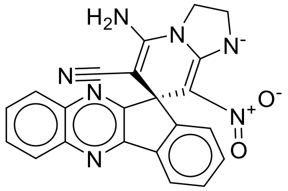
**C-42 (ID 9047)**	**C-50 (ID 50108419)**	**C-36 (ID 50236706)**

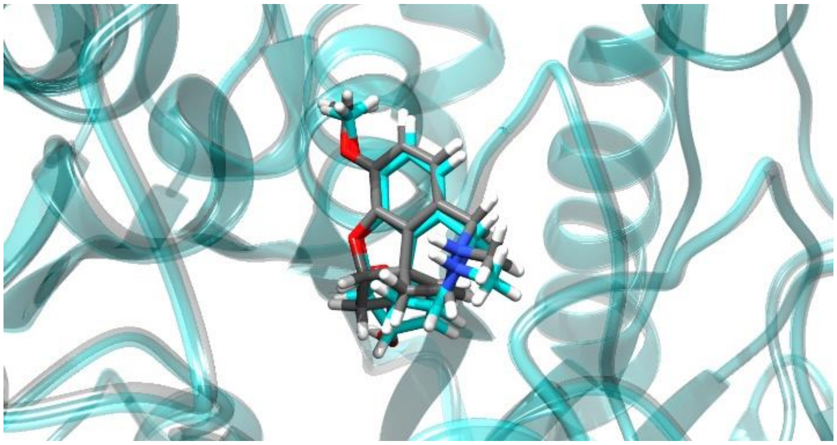

Galantamine (0.93 Å)

Figure S10. Comparison of the best poses produced by the induced-fit docking of galantamine and the experimental pose of galantamine. Gray predicted pose, green actual pose.

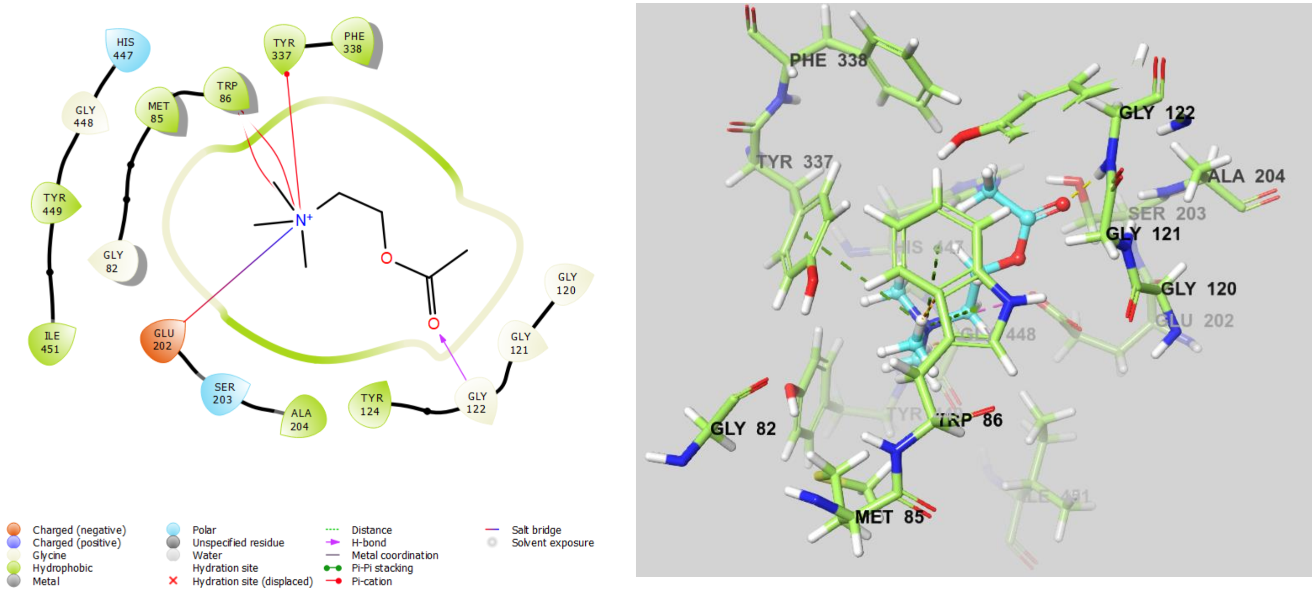

4EY6 (-10.88 kcal/mol)

Figure S11. 2D Interaction diagram and pose produced by the induced-fit docking of acetylcholine.

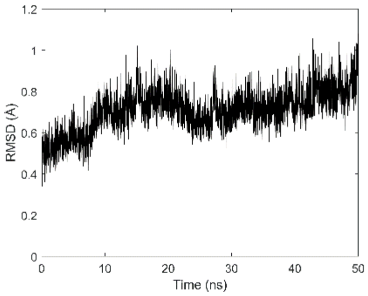	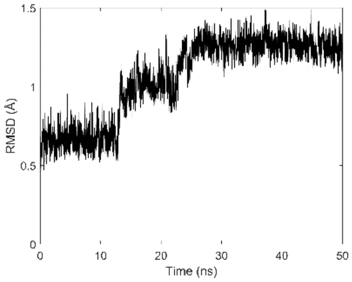	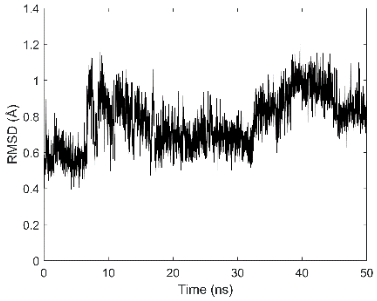
**C4 (ID 50564847)**	**C19 (ID 50558519)**	**C20 (ID 50117583)**
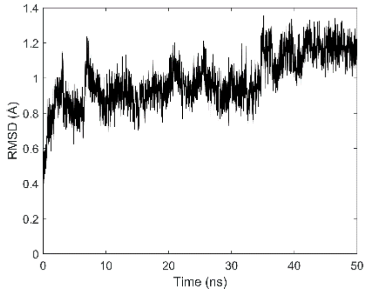	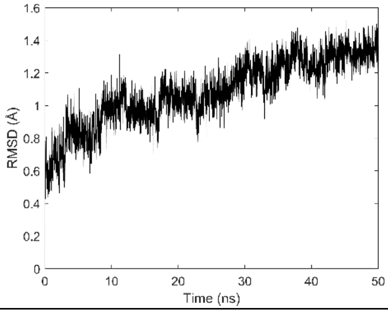	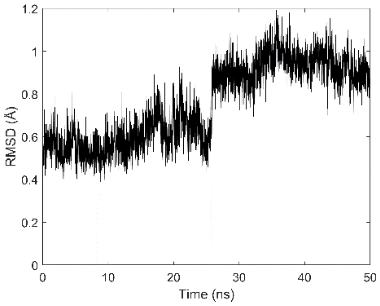
**C23 (ID 50079370)**	**C31 (ID 50190736)**	**C35 (ID 50005500)**
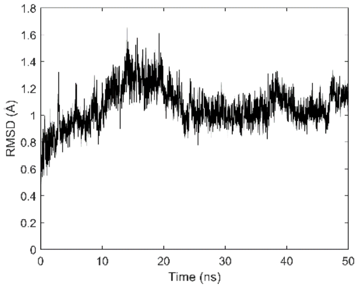	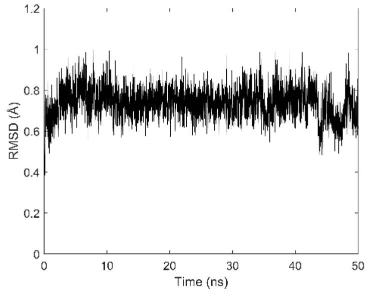	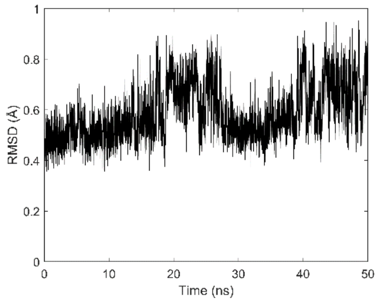
**C36 (ID 9047)**	**C42 (ID 50108419)**	**C50 (50236706)**
	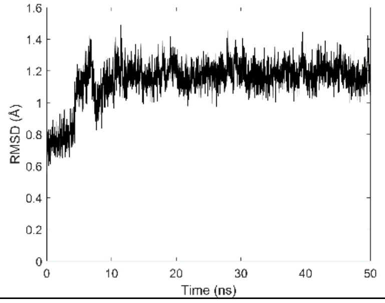	
	**GAL**	

Figure S12. RMSD graphs of backbone atoms of 50 ns MD simulations for the following active site residues: Tyr-72, Asp-74, Trp-86, Gly-120, Gly-121, Tyr-124, Tyr-133, Glu-202, Ser-203, Trp-286, Phe-295, Phe-297, Tyr-337, Phe-338, Tyr-341, His-447, Gly-448, Trp-236, Glu-334, Ala-204, and Gly-122.

Table S4. RMSD Average of 21 residues involved in RMSD paired calculations of 7 conformations for each cluster.

	RMSD Average (Å)									
**Residue**	**C4**	**C19**	**C20**	**C23**	**C31**	**C35**	**C36**	**C42**	**C50**	**GAL**
ALA-204	1.07	1.16	1.04	0.92	0.94	0.97	0.80	0.98	0.86	0.99
ASP-74	1.31	3.57	1.25	2.59	4.08	2.06	2.89	1.70	1.29	2.37
GLU-202	1.12	1.57	0.51	1.76	1.63	0.91	1.10	0.83	0.63	1.21
GLU-334	1.84	0.74	0.83	1.30	0.77	0.78	1.09	0.60	0.59	0.87
GLY-120	1.26	1.29	0.58	0.74	1.42	0.87	1.06	0.93	0.61	1.01
GLY-121	0.59	1.30	0.54	0.66	1.21	0.74	1.06	0.85	0.67	1.01
GLY-122	0.60	1.14	0.59	0.64	0.90	0.79	0.71	0.77	0.55	0.84
GLY-448	1.25	1.79	1.44	2.22	1.10	1.85	1.13	1.14	1.12	1.17
HID-447	1.68	2.36	0.81	1.62	1.45	1.65	1.34	1.16	0.84	1.41
PHE-295	1.45	2.01	1.68	1.78	1.67	0.67	2.08	0.82	0.87	0.80
PHE-297	1.20	1.06	0.93	1.38	0.89	0.81	1.33	0.68	0.96	0.79
PHE-338	1.48	3.24	1.21	1.42	1.22	1.05	1.45	1.09	0.79	1.37
SER-203	0.78	1.02	0.48	0.82	0.98	0.88	0.52	0.79	0.54	0.58
TRP-236	0.72	0.94	0.69	1.21	0.63	0.55	0.92	0.81	0.85	0.65
TRP-286	1.29	1.11	0.69	1.57	1.02	0.66	1.21	1.04	0.87	1.67
TRP-86	0.91	1.60	1.06	1.41	2.39	1.10	1.40	1.49	1.04	3.06
TYR-124	0.80	0.89	0.62	0.86	0.80	0.81	0.97	1.05	0.89	0.80
TYR-133	0.68	1.09	1.35	0.91	0.81	0.72	1.80	0.60	1.02	0.69
TYR-337	0.74	1.24	1.40	2.07	1.01	2.02	1.09	1.78	0.90	1.22
TYR-341	1.24	3.55	1.49	1.97	2.29	1.75	2.59	1.39	1.54	2.50
TYR-72	1.20	1.30	1.39	1.47	1.55	1.15	1.22	1.87	1.35	1.49

Table S5. Residues that presented the highest conformational diversity after RMSD paired calculations for the 7 conformations extracted from the 50 ns MD simulations.

**C4**	Glu-334, His-447, Phe-338, Phe-295, Asp-74
**C19**	Asp-74, Tyr-341, Phe-338, His-447, Phe-295
**C20**	Phe-295, Tyr-341, Gly-448, Tyr-337, Tyr-72
**C23**	Asp-74, Gly-447, Tyr-337, Tyr-341, Phe-295
**C31**	Asp-74, Trp-86, Tyr-341, Phe-295, Glu-202
**C35**	Asp-74, Tyr-337, Gly-448, Tyr-341, His-447
**C36**	Asp-74, Tyr-341, Phe-295, Tyr-133, Phe-338
**C42**	Tyr-72, Tyr-337, Asp-74, Trp-86, Tyr-341
**C50**	Tyr-341, Tyr-72, Asp-74, Gly-447, Trp-86
**GAL**	Trp-86, Tyr-341, Asp-74, Trp-286, Tyr-72

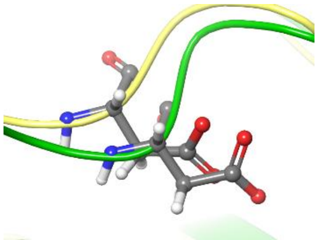

Asp-74 (C4)

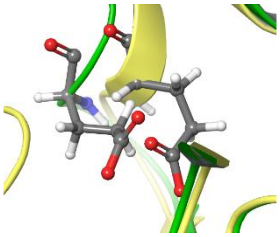

Glu-334 (C4)

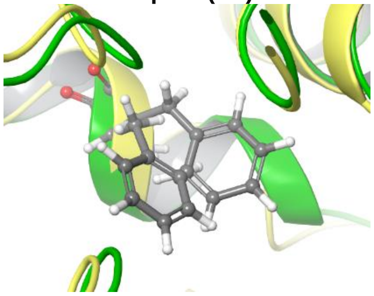

Phe-338 (C4)

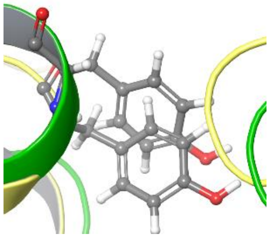

Tyr-341 (C20)

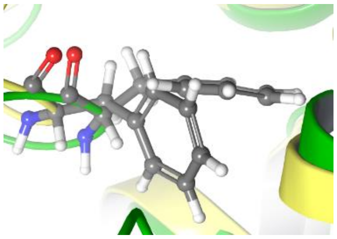

Phe-295 (C20)

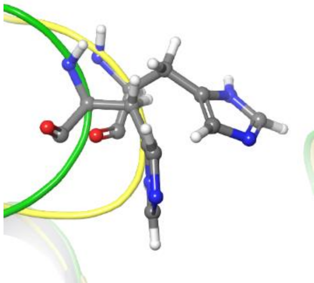

His-447 (C4)

Figure S13. Conformational changes of some active site residues through the course of molecular dynamics for C4 and C20 representative ligands.

Table S6. Simulation time (nanoseconds) of the frames belonging to the average RMSD, average RMSD plus twice the RMSD standard deviation, average RMSD minus twice the RMSD standard deviation, the two smallest RMSD values, and the two largest RMSD values. The values in red correspond to the frames selected according to the RMSD heatmaps.

	C4	C19	C20	C23	C31	C35	C36	C42	C50	GAL
**Average**	17.96	18.27	14.20	26.05	25.85	46.98	35.83	44.35	36.21	9.65
**First Minimum**	0.09	0.14	4.23	0.11	0.08	3.29	0.05	0.14	0.42	0.04
**Second minimum**	1.82	0.25	0.09	0.03	0.50	9.42	0.08	0.04	9.32	2.08
**First maximum**	49.78	44.86	39.66	35.12	45.23	35.65	14.03	48.28	48.57	11.5
**Second maximum**	49.98	37.21	38.72	48.76	49.75	37.67	19.26	36.94	43.56	28.02
**Average + 2 standard deviations**	14.98	37.36	40.57	42.09	49.14	36.60	17.15	5.79	19.37	49.65
**Average - 2 standard deviations**	1.07	2.82	1.32	5.87	0.82	8.87	2.37	25.92	1.88	3.39

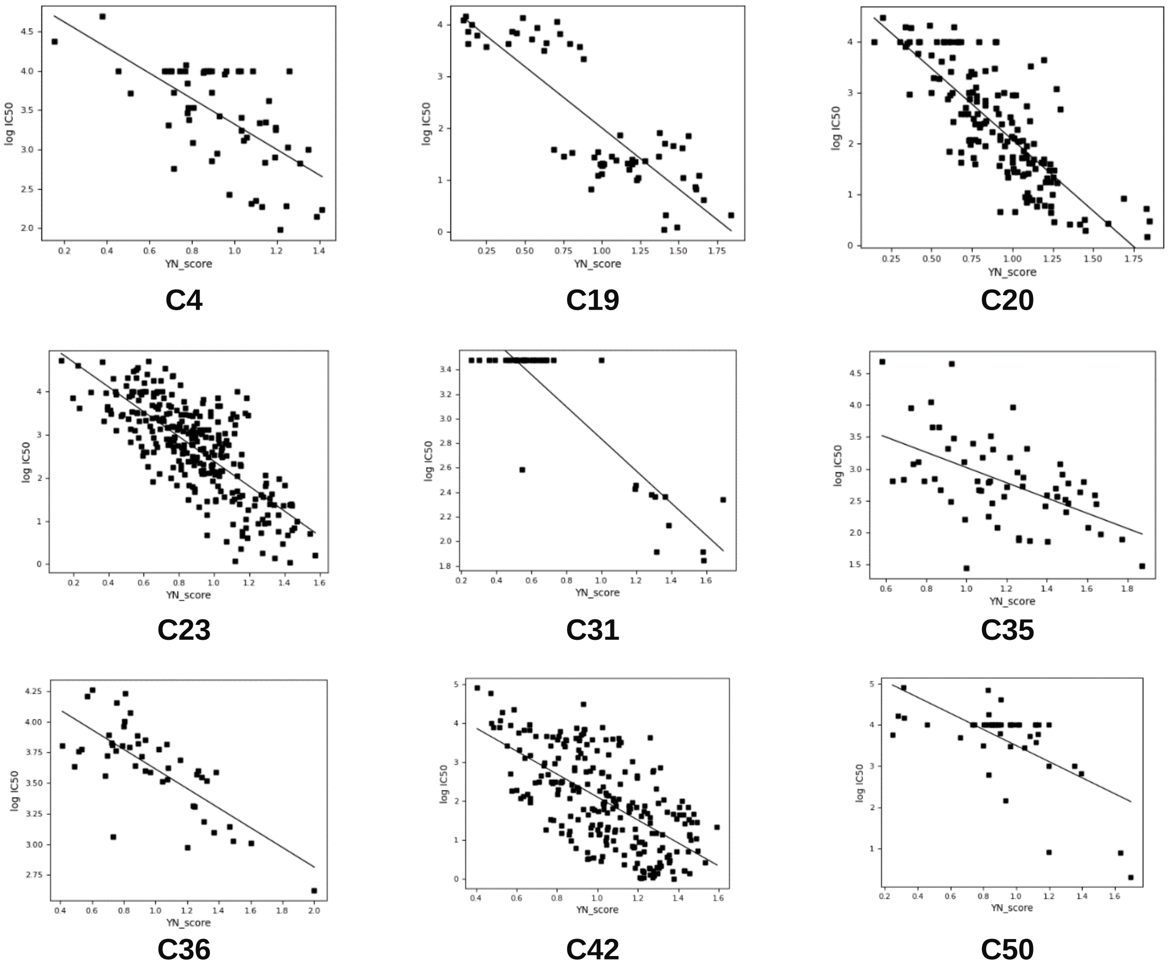

Figure S14. logIC50 vs Yasser's Number 1 (YN1) score of the ensemble docking calculations for 31, 4, 19, 50, 35, 20, 36, 42, and 23 clusters.

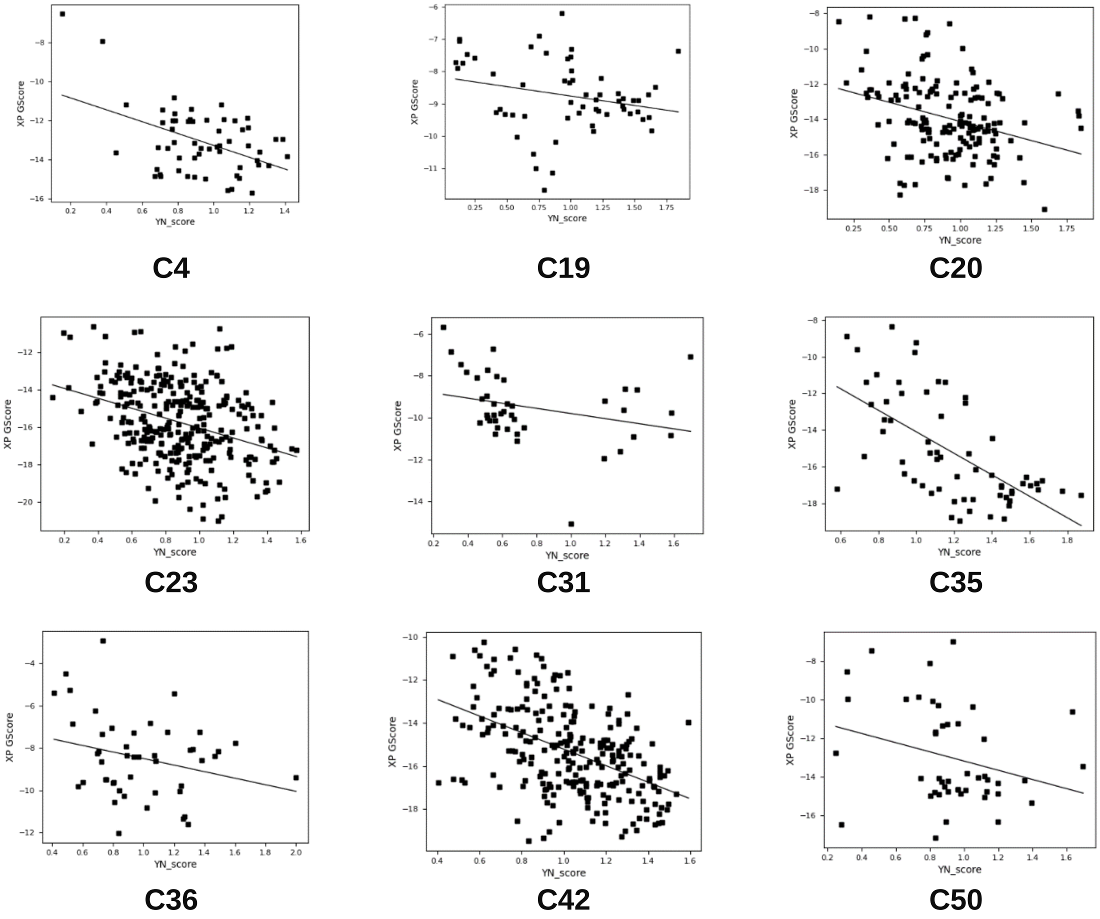

Figure S15. XP GScore vs Yasser's Number 1 (YN1) score of the ensemble docking calculations for 31, 4, 19, 50, 35, 20, 36, 42, and 23 clusters.

Table S7. Ligands that displayed best experimental inhibition and docking scores from the ensemble docking procedure for 31, 4, 19, 50, 35, 20, 36, 42, and 23 families.

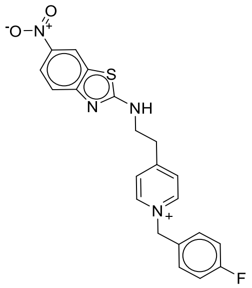

C-4 (ID 205985)
XP GScore (-15.699 kcal/mol)
IC50 96 nM

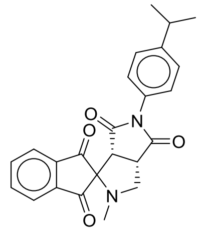

C-19 (ID 50242993)
XP GScore (-9.821 kcal/mol)
IC50 12 nM

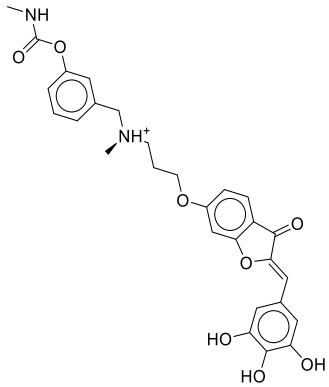

C-23 (ID 50318726)
XP GScore (-19.439 kcal/mol)
IC50 2.99 nM

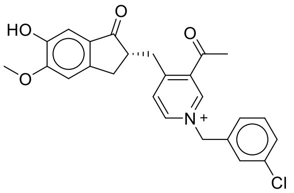

C-20 (ID 50291432)
XP GScore (-14.079 kcal/mol)
IC50 2.7 nM

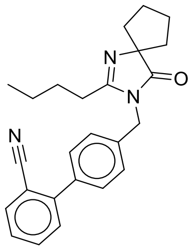

C-31 (ID 50042242)
XP GScore (-10.835 kcal/mol)
IC50 82.5 nM

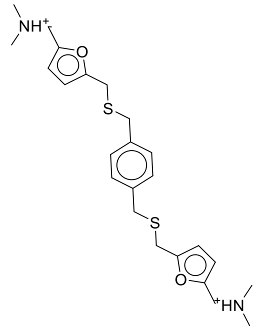

C-35 (ID 50005502)
XP GScore (-18.717 kcal/mol)
IC50 260 nM

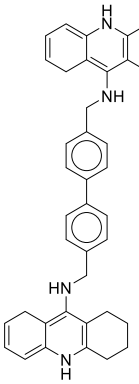

C-42 (ID 50510840)
XP GScore (-18.986 kcal/mol)
IC50 3.6 nM

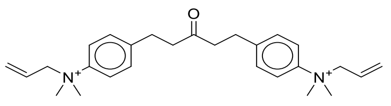

C-50 (ID 10624)
XP GScore (-16.312 kcal/mol)
IC50 8 nM

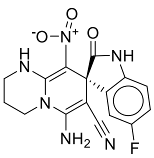

C-36 (ID 50147785)
XP GScore (-9.404 kcal/mol)
IC50 420 nM

Table S8. Training and testing results of C4 Machine Learning models. Logistic Regression=LR, Random Forest=RF, Support vector=SV, Decision Tree=DT. *Average Metrics Cross Validation* corresponds to the average values of the metrics Accuracy, Recall, Specificity, and ROC AUC across different iterations of cross-validation. *Test metrics* correspond to the values of the metrics Accuracy, Recall, Specificity, and ROC AUC for the test set. *Test Excluded Decoys of Training* corresponds to a test using the decoys that were excluded from training and cross-validation after the clustering process. *Test BDB Molecules* corresponds to a test using the 4643 initial molecules from the binding database. *Test Cluster Molecules* corresponds to a test using all molecules from cluster C4. *Test BDB Molecules Excluding Cluster Molecules* corresponds to a test using the 4643 initial molecules from the binding database and excluding molecules from cluster C4.

		**LR**	**SV**	**DT**	**RF**
Average Metrics Cross Validation	**Accuracy**	0.979	0.979	0.947	0.990
	**Recall**	0.980	0.980	0.960	0.980
	**Specificity**	0.978	0.978	0.936	1.000
	**ROC AUC**	0.979	0.979	0.948	0.990
Test Metrics	**Accuracy**	0.997	0.995	0.983	0.995
	**Recall**	1.000	1.000	1.000	1.000
	**Specificity**	0.997	0.995	0.983	0.995
	**ROC AUC**	0.998	0.998	0.991	0.998
Test Excluded decoys of training	**Percentage of True Negatives**	99.3%	99.2%	98.8%	99.5%
Test BDB molecules	**Percentage of active compounds in the library**	54.5%	56.3%	19.2%	19.5%
	**Percentage of inactive compounds in the library**	45.5%	43.7%	80.8%	80.5%
Test family molecules	**Percentage of active compounds in the library**	100.0%	100.0%	96.6%	100.0%
	**Percentage of inactive compounds in the library**	0.0%	0.0%	3.4%	0.0%
Test BDB molecules excluding family molecules	**Percentage of active compounds in the library**	53.9%	55.7%	18.2%	18.5%
	**Percentage of inactive compounds in the library**	46.1%	44.3%	81.8%	81.5%

Table S9. Training and testing results of C19 Machine Learning models. Logistic Regression=LR, Random Forest=RF, Support vector=SV, Decision Tree=DT. *Average Metrics Cross Validation* corresponds to the average values of the metrics Accuracy, Recall, Specificity, and ROC AUC across different iterations of cross-validation. *Test metrics* correspond to the values of the metrics Accuracy, Recall, Specificity, and ROC AUC for the test set. *Test Excluded Decoys of Training* corresponds to a test using the decoys that were excluded from training and cross-validation after the clustering process. *Test BDB Molecules* corresponds to a test using the 4643 initial molecules from the binding database. *Test Cluster Molecules* corresponds to a test using all molecules from cluster C19. *Test BDB Molecules Excluding Cluster Molecules* corresponds to a test using the 4643 initial molecules from the binding database and excluding molecules from cluster C19.

		**LR**	**SV**	**DT**	**RF**
Average Metrics Cross Validation	**Accuracy**	1.000	1.000	1.000	1.000
	**Recall**	1.000	1.000	1.000	1.000
	**Specificity**	1.000	1.000	1.000	1.000
	**ROC AUC**	1.000	1.000	1.000	1.000
Test Metrics	**Accuracy**	0.998	1.000	0.993	0.998
	**Recall**	1.000	1.000	1.000	1.000
	**Specificity**	0.998	1.000	0.993	0.998
	**ROC AUC**	0.999	1.000	0.996	0.999
Test Excluded decoys of training	**Percentage of True Negatives**	100.0%	100.0%	98.4%	100.0%
Test BDB molecules	**Percentage of active compounds in the library**	4.1%	1.4%	3.1%	2.0%
	**Percentage of inactive compounds in the library**	95.9%	98.6%	96.9%	98.0%
Test family molecules	**Percentage of active compounds in the library**	100.0%	98.4%	100.0%	100.0%
	**Percentage of inactive compounds in the library**	0.0%	1.6%	0.0%	0.0%
Test BDB molecules excluding family molecules	**Percentage of active compounds in the library**	2.7%	0.0%	1.7%	0.6%
	**Percentage of inactive compounds in the library**	97.3%	100.0%	98.3%	99.4%

Table S10. Training and testing results of C20 Machine Learning models. Logistic Regression=LR, Random Forest=RF, Support vector=SV, Decision Tree=DT. *Average Metrics Cross Validation* corresponds to the average values of the metrics Accuracy, Recall, Specificity, and ROC AUC across different iterations of cross-validation. *Test metrics* correspond to the values of the metrics Accuracy, Recall, Specificity, and ROC AUC for the test set. *Test Excluded Decoys of Training* corresponds to a test using the decoys that were excluded from training and cross-validation after the clustering process. *Test BDB Molecules* corresponds to a test using the 4643 initial molecules from the binding database. *Test Cluster Molecules* corresponds to a test using all molecules from cluster C20. *Test BDB Molecules Excluding Cluster Molecules* corresponds to a test using the 4643 initial molecules from the binding database and excluding molecules from cluster C20.

		LR	SV	DT	RF
Average Metrics Cross Validation	**Accuracy**	0.988	0.980	0.960	0.988
	**Recall**	0.992	0.960	0.968	0.976
	**Specificity**	0.984	1.000	0.952	1.000
	**ROC AUC**	0.988	0.980	0.960	0.988
Test Metrics	**Accuracy**	0.987	0.982	0.945	0.992
	**Recall**	1.000	1.000	1.000	0.871
	**Specificity**	0.987	0.982	0.943	0.994
	**ROC AUC**	0.993	0.991	0.972	0.933
Test Excluded decoys of training	**Percentage of True Negatives**	99.4%	99.6%	95.9%	99.8%
Test BDB molecules	**Percentage of active compounds in the library**	31.7%	18.2%	22.8%	21.6%
	**Percentage of inactive compounds in the library**	68.3%	81.8%	77.2%	78.4%
Test family molecules	**Percentage of active compounds in the library**	93.5%	87.0%	93.1%	91.2%
	**Percentage of inactive compounds in the library**	6.5%	13.0%	6.9%	8.8%
Test BDB molecules excluding family molecules	**Percentage of active compounds in the library**	28.1%	14.1%	18.6%	17.5%
	**Percentage of inactive compounds in the library**	71.9%	85.9%	81.4%	82.5%

Table S11. Training and testing results of C23 Machine Learning models. Logistic Regression=LR, Random Forest=RF, Support vector=SV, Decision Tree=DT. *Average Metrics Cross Validation* corresponds to the average values of the metrics Accuracy, Recall, Specificity, and ROC AUC across different iterations of cross-validation. *Test metrics* correspond to the values of the metrics Accuracy, Recall, Specificity, and ROC AUC for the test set. *Test Excluded Decoys of Training* corresponds to a test using the decoys that were excluded from training and cross-validation after the clustering process. *Test BDB Molecules* corresponds to a test using the 4643 initial molecules from the binding database. *Test Cluster Molecules* corresponds to a test using all molecules from cluster C23. *Test BDB Molecules Excluding Cluster Molecules* corresponds to a test using the 4643 initial molecules from the binding database and excluding molecules from cluster C23.

		LR	SV	DT	RF
Average Metrics Cross Validation	**Accuracy**	0.949	0.951	0.916	0.970
	**Recall**	0.966	0.932	0.910	0.961
	**Specificity**	0.932	0.970	0.923	0.979
	**ROC AUC**	0.949	0.951	0.916	0.970
Test Metrics	**Accuracy**	0.965	0.984	0.902	0.988
	**Recall**	0.966	0.948	0.914	0.948
	**Specificity**	0.965	0.984	0.902	0.989
	**ROC AUC**	0.965	0.966	0.908	0.969
Test Excluded decoys of training	**Percentage of True Negatives**	96.9%	98.6%	92.6%	99.1%
Test BDB molecules	**Percentage of active compounds in the library**	39.4%	34.5%	29.6%	28.7%
	**Percentage of inactive compounds in the library**	60.6%	65.5%	70.4%	71.3%
Test family molecules	**Percentage of active compounds in the library**	86.5%	81.6%	70.1%	77.4%
	**Percentage of inactive compounds in the library**	13.5%	18.4%	29.9%	22.6%
Test BDB molecules excluding family molecules	**Percentage of active compounds in the library**	17.9%	13.0%	11.1%	6.5%
	**Percentage of inactive compounds in the library**	82.1%	87.0%	88.9%	93.5%

Table S12. Training and testing results of C31 Machine Learning models. Logistic Regression=LR, Random Forest=RF, Support vector=SV, Decision Tree=DT. *Average Metrics Cross Validation* corresponds to the average values of the metrics Accuracy, Recall, Specificity, and ROC AUC across different iterations of cross-validation. *Test metrics* correspond to the values of the metrics Accuracy, Recall, Specificity, and ROC AUC for the test set. *Test Excluded Decoys of Training* corresponds to a test using the decoys that were excluded from training and cross-validation after the clustering process. *Test BDB Molecules* corresponds to a test using the 4643 initial molecules from the binding database. *Test Cluster Molecules* corresponds to a test using all molecules from cluster C31. *Test BDB Molecules Excluding Cluster Molecules* corresponds to a test using the 4643 initial molecules from the binding database and excluding molecules from cluster C31.

		LR	SV	DT	RF
Average Metrics Cross Validation	**Accuracy**	0.954	0.969	0.879	0.955
	**Recall**	0.967	0.967	0.938	0.967
	**Specificity**	0.933	0.967	0.819	0.938
	**ROC AUC**	0.950	0.967	0.879	0.952
Test Metrics	**Accuracy**	0.893	0.995	0.908	0.973
	**Recall**	1.000	0.875	1.000	1.000
	**Specificity**	0.890	0.997	0.906	0.972
	**ROC AUC**	0.945	0.936	0.953	0.986
Test Excluded decoys of training	**Percentage of True Negatives**	96.5%	99.2%	92.6%	97.4%
Test BDB molecules	**Percentage of active compounds in the library**	25.4%	1.2%	46.6%	14.6%
	**Percentage of inactive compounds in the library**	74.7%	98.8%	53.4%	85.4%
Test family molecules	**Percentage of active compounds in the library**	100.0%	97.9%	100.0%	100.0%
	**Percentage of inactive compounds in the library**	0.0%	2.1%	0.0%	0.0%
Test BDB molecules excluding family molecules	**Percentage of active compounds in the library**	24.6%	0.2%	46.0%	13.7%
	**Percentage of inactive compounds in the library**	75.4%	99.8%	54.0%	86.3%

Table S13. Training and testing results of C35 Machine Learning models. Logistic Regression=LR, Random Forest=RF, Support vector=SV, Decision Tree=DT. *Average Metrics Cross Validation* corresponds to the average values of the metrics Accuracy, Recall, Specificity, and ROC AUC across different iterations of cross-validation. *Test metrics* correspond to the values of the metrics Accuracy, Recall, Specificity, and ROC AUC for the test set. *Test Excluded Decoys of Training* corresponds to a test using the decoys that were excluded from training and cross-validation after the clustering process. *Test BDB Molecules* corresponds to a test using the 4643 initial molecules from the binding database. *Test Cluster Molecules* corresponds to a test using all molecules from cluster C35. *Test BDB Molecules Excluding Cluster Molecules* corresponds to a test using the 4643 initial molecules from the binding database and excluding molecules from cluster C35.

		**LR**	**SV**	**DT**	**RF**
Average Metrics Cross Validation	**Accuracy**	0.990	1.000	1.000	1.000
	**Recall**	1.000	1.000	1.000	1.000
	**Specificity**	0.980	1.000	1.000	1.000
	**ROC AUC**	0.990	1.000	1.000	1.000
Test Metrics	**Accuracy**	0.998	1.000	0.989	1.000
	**Recall**	1.000	1.000	1.000	1.000
	**Specificity**	0.998	1.000	0.989	1.000
	**ROC AUC**	0.999	1.000	0.995	1.000
Test Excluded decoys of training	**Percentage of True Negatives**	99.6%	99.9%	98.0%	99.8%
Test BDB molecules	**Percentage of active compounds in the library**	2.1%	1.5%	3.2%	1.6%
	**Percentage of inactive compounds in the library**	97.9%	98.5%	96.8%	98.4%
Test family molecules	**Percentage of active compounds in the library**	100.0%	98.5%	100.0%	100.0%
	**Percentage of inactive compounds in the library**	0.0%	1.5%	0.0%	0.0%
Test BDB molecules excluding family molecules	**Percentage of active compounds in the library**	0.6%	0.0%	1.8%	0.2%
	**Percentage of inactive compounds in the library**	99.4%	100.0%	98.2%	99.8%

Table S14. Training and testing results of C36 Machine Learning models. Logistic Regression=LR, Random Forest=RF, Support vector=SV, Decision Tree=DT. *Average Metrics Cross Validation* corresponds to the average values of the metrics Accuracy, Recall, Specificity, and ROC AUC across different iterations of cross-validation. *Test metrics* correspond to the values of the metrics Accuracy, Recall, Specificity, and ROC AUC for the test set. *Test Excluded Decoys of Training* corresponds to a test using the decoys that were excluded from training and cross-validation after the clustering process. *Test BDB Molecules* corresponds to a test using the 4643 initial molecules from the binding database. *Test Cluster Molecules* corresponds to a test using all molecules from cluster C36. *Test BDB Molecules Excluding Cluster Molecules* corresponds to a test using the 4643 initial molecules from the binding database and excluding molecules from cluster C36.

		LR	SV	DT	RF
Average Metrics Cross Validation	**Accuracy**	1.000	1.000	0.987	1.000
	**Recall**	1.000	1.000	0.971	1.000
	**Specificity**	1.000	1.000	1.000	1.000
	**ROC AUC**	1.000	1.000	0.986	1.000
Test Metrics	**Accuracy**	1.000	1.000	1.000	1.000
	**Recall**	1.000	1.000	1.000	1.000
	**Specificity**	1.000	1.000	1.000	1.000
	**ROC AUC**	1.000	1.000	1.000	1.000
Test Excluded decoys of training	**Percentage of True Negatives**	99.9%	99.9%	99.7%	100.0%
Test BDB molecules	**Percentage of active compounds in the library**	1.9%	1.0%	2.4%	1.0%
	**Percentage of inactive compounds in the library**	98.1%	99.0%	97.6%	99.0%
Test family molecules	**Percentage of active compounds in the library**	100.0%	100.0%	100.0%	100.0%
	**Percentage of inactive compounds in the library**	0.0%	0.0%	0.0%	0.0%
Test BDB molecules excluding family molecules	**Percentage of active compounds in the library**	0.9%	0.0%	1.4%	0.0%
	**Percentage of inactive compounds in the library**	99.1%	100.0%	98.6%	100.0%

Table S15. Training and testing results of C42 Machine Learning models. Logistic Regression=LR, Random Forest=RF, Support vector=SV, Decision Tree=DT. *Average Metrics Cross Validation* corresponds to the average values of the metrics Accuracy, Recall, Specificity, and ROC AUC across different iterations of cross-validation. *Test metrics* correspond to the values of the metrics Accuracy, Recall, Specificity, and ROC AUC for the test set. *Test Excluded Decoys of Training* corresponds to a test using the decoys that were excluded from training and cross-validation after the clustering process. *Test BDB Molecules* corresponds to a test using the 4643 initial molecules from the binding database. *Test Cluster Molecules* corresponds to a test using all molecules from cluster C42. *Test BDB Molecules Excluding Cluster Molecules* corresponds to a test using the 4643 initial molecules from the binding database and excluding molecules from cluster C42.

		**LR**	**SV**	**DT**	**RF**
Average Metrics Cross Validation	**Accuracy**	0.982	0.990	0.930	0.995
	**Recall**	0.990	0.984	0.958	0.990
	**Specificity**	0.974	0.995	0.901	1.000
	**ROC AUC**	0.982	0.990	0.930	0.995
Test Metrics	**Accuracy**	0.981	0.992	0.953	0.998
	**Recall**	0.979	1.000	1.000	0.979
	**Specificity**	0.981	0.992	0.952	0.999
	**ROC AUC**	0.980	0.996	0.976	0.989
Test Excluded decoys of training	**Percentage of True Negatives**	97.8%	99.4%	95.7%	99.8%
Test BDB molecules	**Percentage of active compounds in the library**	25.7%	23.0%	20.1%	16.9%
	**Percentage of inactive compounds in the library**	74.3%	77.0%	79.9%	83.1%
Test family molecules	**Percentage of active compounds in the library**	73.4%	74.6%	73.3%	69.9%
	**Percentage of inactive compounds in the library**	26.6%	25.4%	26.7%	30.1%
Test BDB molecules excluding family molecules	**Percentage of active compounds in the library**	11.9%	8.1%	4.8%	1.7%
	**Percentage of inactive compounds in the library**	88.1%	91.9%	95.2%	98.3%

Table S16. Training and testing results of C50 Machine Learning models. Logistic Regression=LR, Random Forest=RF, Support vector=SV, Decision Tree=DT. *Average Metrics Cross Validation* corresponds to the average values of the metrics Accuracy, Recall, Specificity, and ROC AUC across different iterations of cross-validation. *Test metrics* correspond to the values of the metrics Accuracy, Recall, Specificity, and ROC AUC for the test set. *Test Excluded Decoys of Training* corresponds to a test using the decoys that were excluded from training and cross-validation after the clustering process. *Test BDB Molecules* corresponds to a test using the 4643 initial molecules from the binding database. *Test Cluster Molecules* corresponds to a test using all molecules from cluster C50. *Test BDB Molecules Excluding Cluster Molecules* corresponds to a test using the 4643 initial molecules from the binding database and excluding molecules from cluster C50.

		**LR**	**SV**	**DT**	**RF**
Average Metrics Cross Validation	**Accuracy**	0.987	0.987	0.906	0.946
	**Recall**	0.971	0.971	0.921	0.918
	**Specificity**	1.000	1.000	0.896	0.975
	**ROC AUC**	0.986	0.986	0.909	0.946
Test Metrics	**Accuracy**	0.911	0.922	0.940	0.982
	**Recall**	1.000	1.000	1.000	1.000
	**Specificity**	0.909	0.921	0.939	0.982
	**ROC AUC**	0.955	0.960	0.969	0.991
Test Excluded decoys of training	**Percentage of True Negatives**	95.6%	95.9%	93.4%	98.9%
Test BDB molecules	**Percentage of active compounds in the library**	25.9%	30.0%	33.6%	4.2%
	**Percentage of inactive compounds in the library**	74.1%	70.0%	66.4%	95.8%
Test family molecules	**Percentage of active compounds in the library**	98.5%	98.5%	92.7%	94.1%
	**Percentage of inactive compounds in the library**	1.5%	1.5%	7.4%	5.9%
Test BDB molecules excluding family molecules	**Percentage of active compounds in the library**	24.9%	28.9%	32.7%	2.9%
	**Percentage of inactive compounds in the library**	75.1%	71.1%	67.3%	97.1%

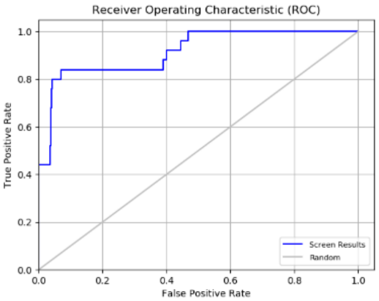

C-4 (ROC-AUC:0.92)

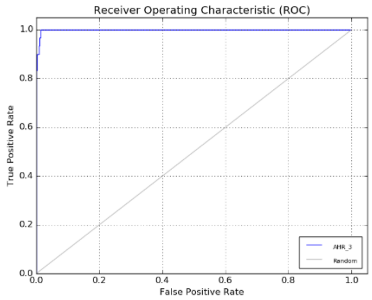

C-19 (ROC-AUC:0.99)

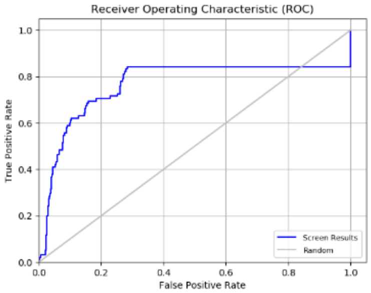

C-23 (ROC-AUC:0.77)

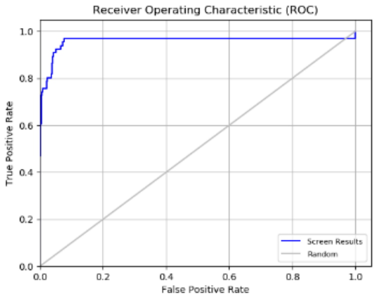

C-20 (ROC-AUC:0.96)

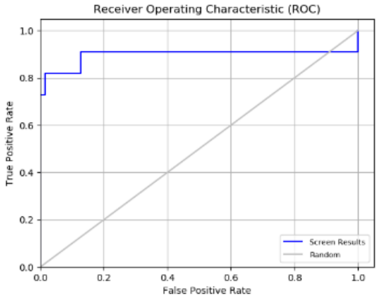

C-31 (ROC-AUC:0.90)

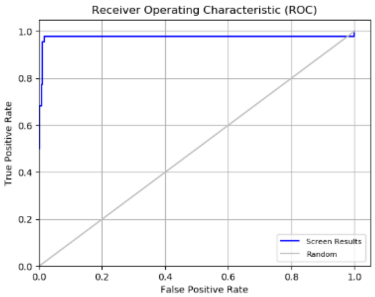

C-35 (ROC-AUC:0.97)

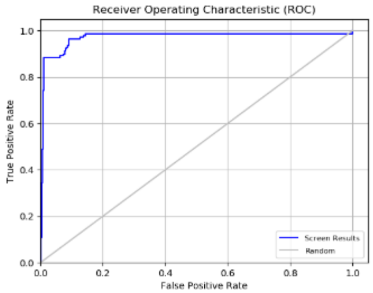

C-42 (ROC-AUC:0.97)

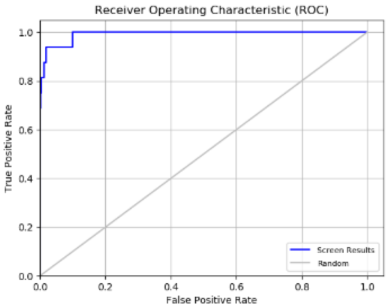

C-50 (ROC-AUC:0.99)

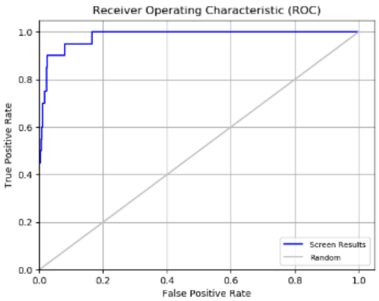

C-36 (ROC-AUC:0.98)

Figure S16. ROC curve for best ligand-based pharmacophore models.

(A) (B)

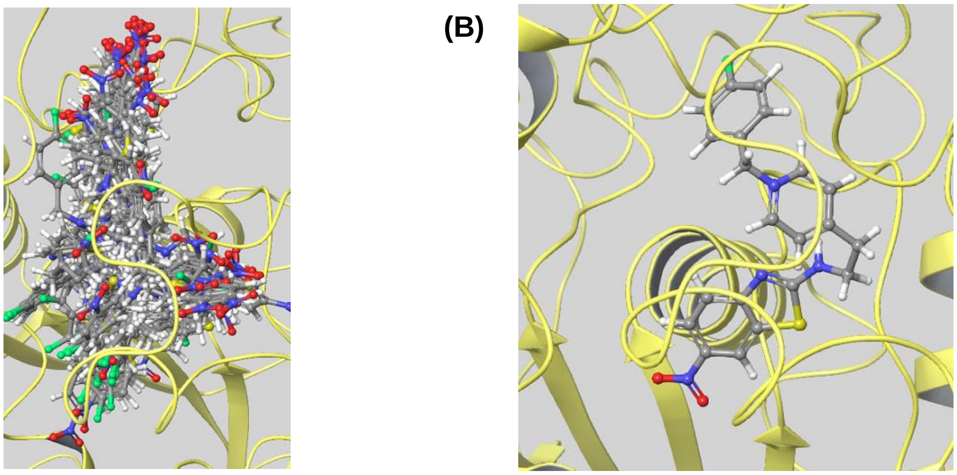

Figure S17. Example of geometry clustering. (A) Ligand poses and **(B)** the most populated pose obtained for the compound of C4.

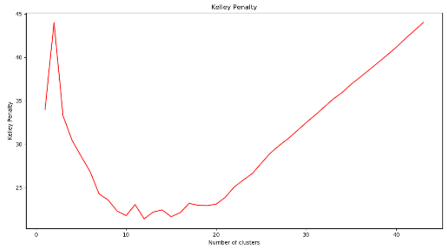

C-4

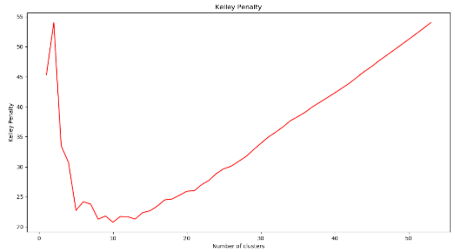

C-19

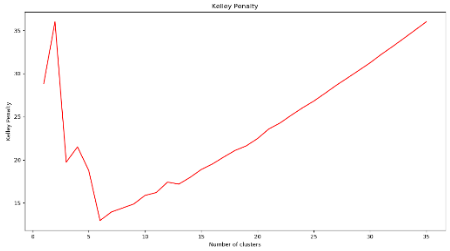

C-20

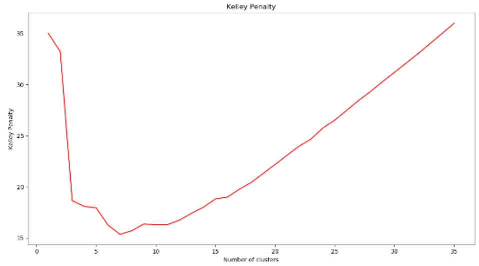

C-23

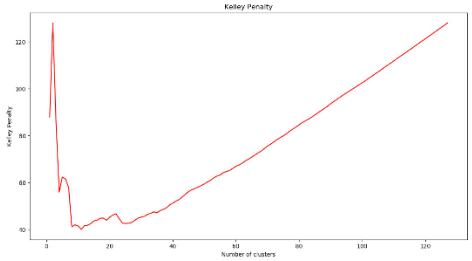

C-31

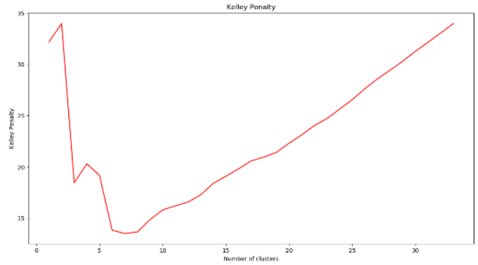

C-35

Figure S18. Kelley penalty graphs for pose clustering from the ensemble docking calculations for 4, 19, 20, 23, 31, and 35 compounds.

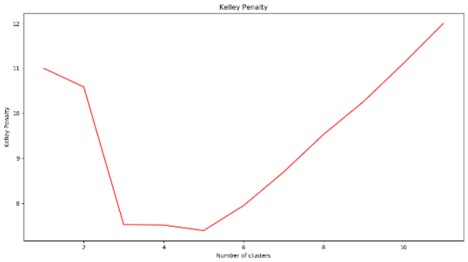

C-36

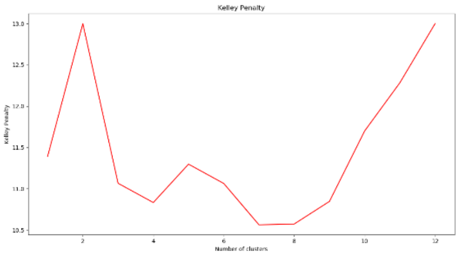

C-42

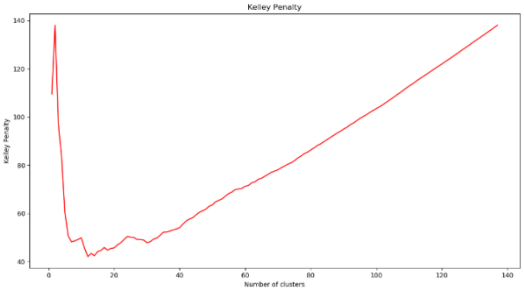

C-50

Figure S19. Kelley penalty graphs for pose clustering from the ensemble docking calculations for 36, 42, and 50 compounds.

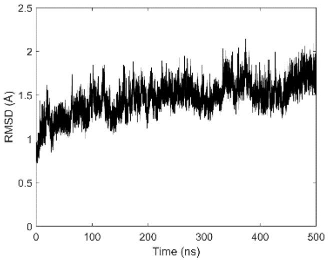

C4

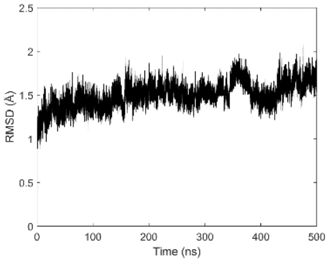

C19

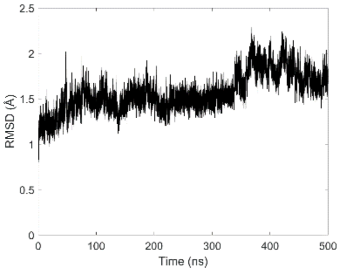

C20

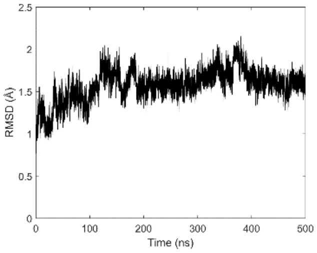

C23

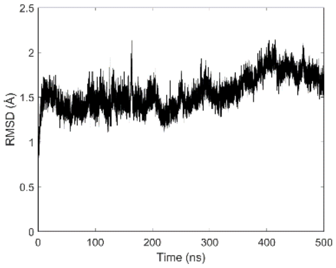

C31

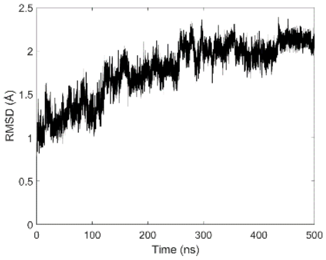

C35

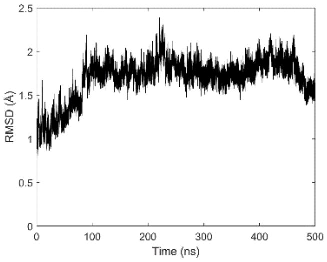

C36

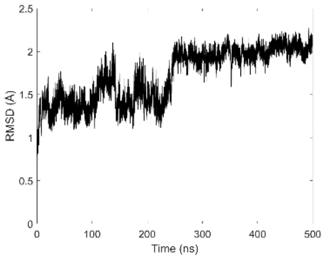

C42

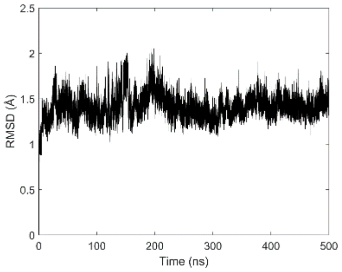

C50

Figure S20. RMSD graphs of backbone atoms of 500 ns MD simulations for the whole protein.

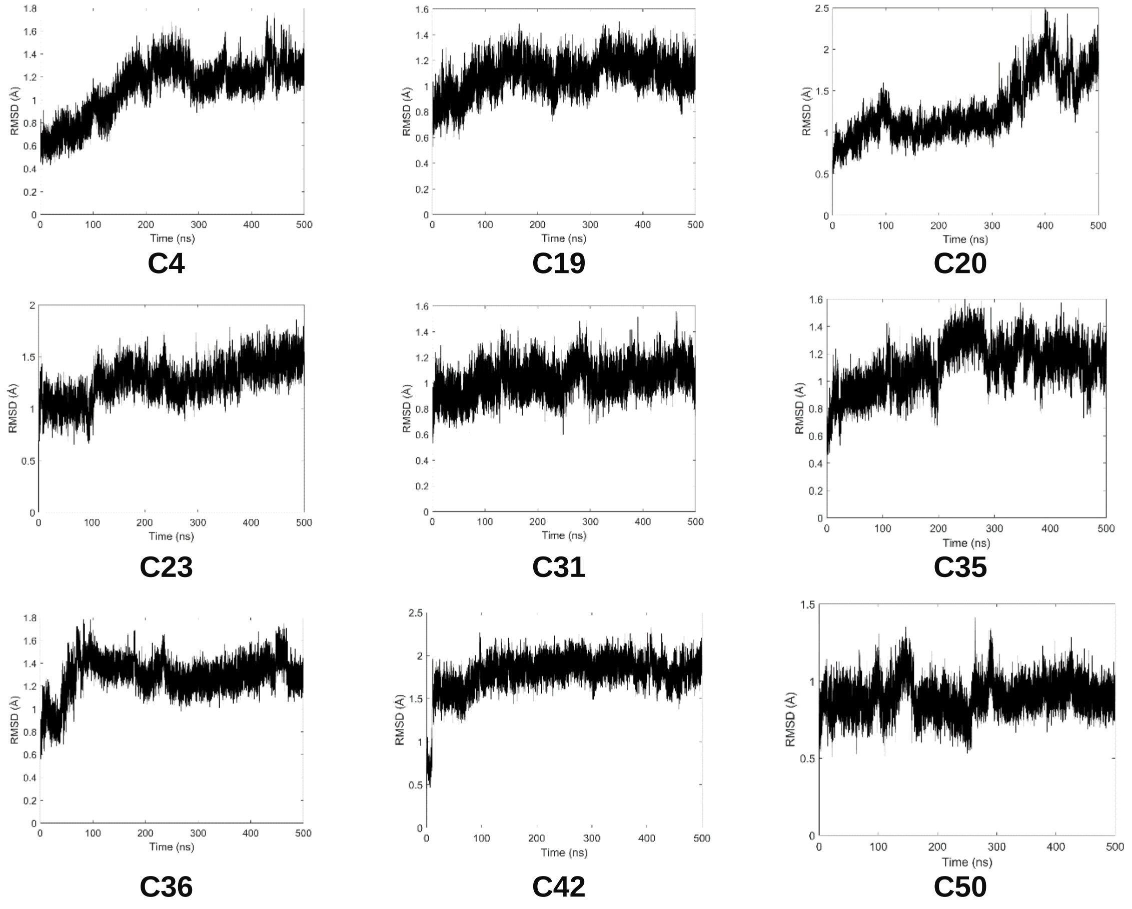

Figure S21. RMSD graphs of backbone atoms of 500 ns MD simulations for the following active site residues: Tyr-72, Asp-74, Trp-86, Gly-120, Gly-121, Tyr-124, Tyr-133, Glu-202, Ser-203, Trp-286, Phe-295, Phe-297, Tyr-337, Phe-338, Tyr-341, His-447, Gly-448, Trp-236, Glu-334, Ala-204, and Gly-122.

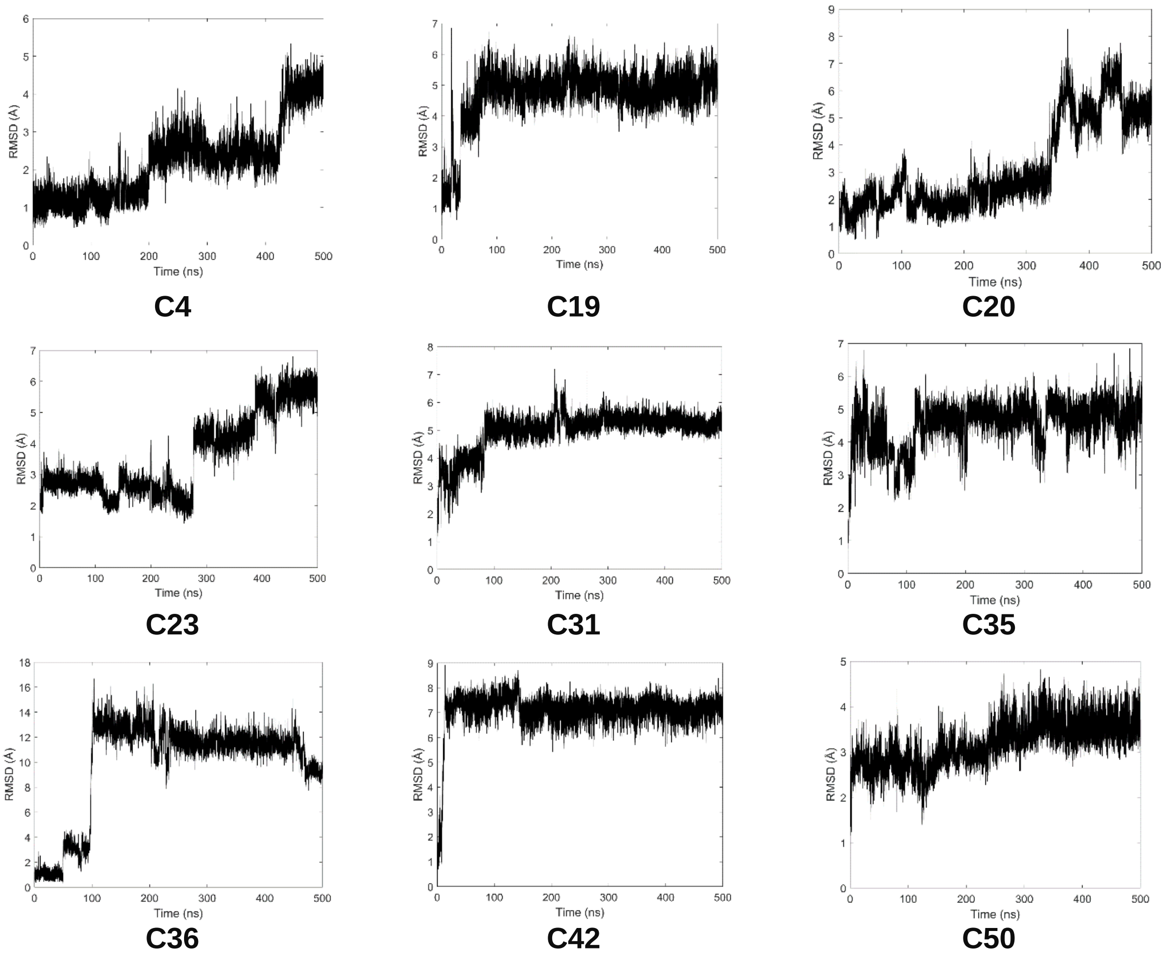

Figure S22. RMSD graphs of 500 ns MD simulations for the ligand heavy atoms.

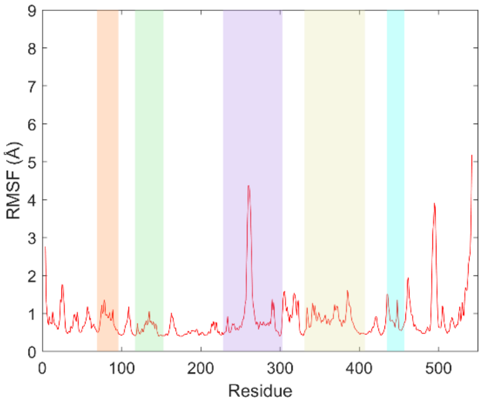

C4

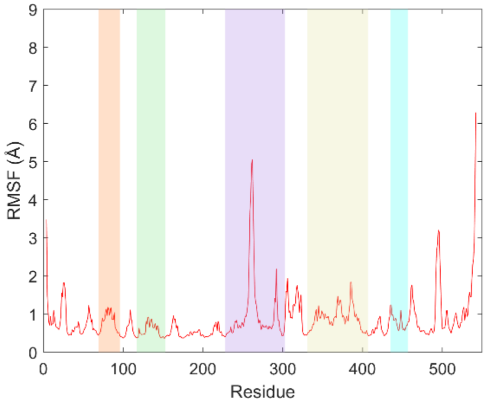

C19

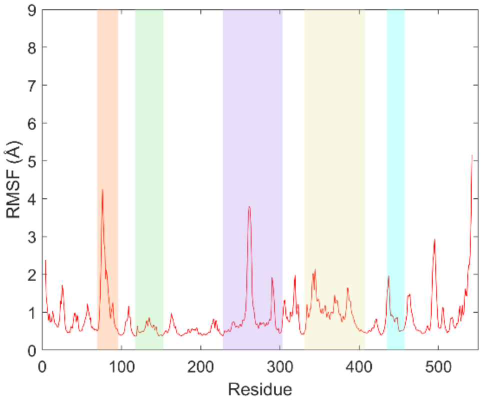

C20

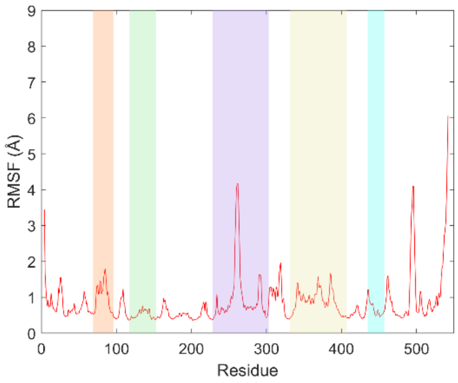

C23

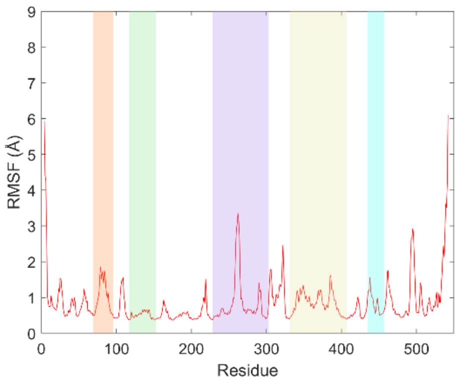

C31

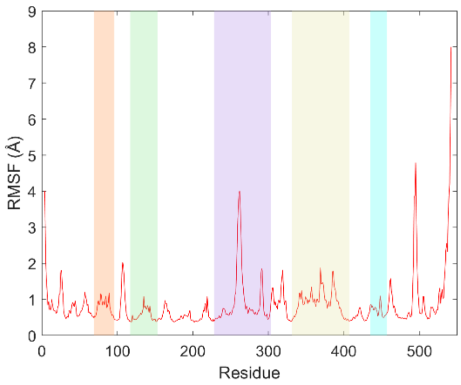

C35

Figure S23. RMSF values for backbone of 500 ns MD simulations for C4, C19, C20, C23, C31 and C35. Green for S1 domain (117-153), purple for S2 domain (228-303), yellow for S3 domain (331-407), cyan for S4 domain (435-457), and red for Ω-loop (69-96).

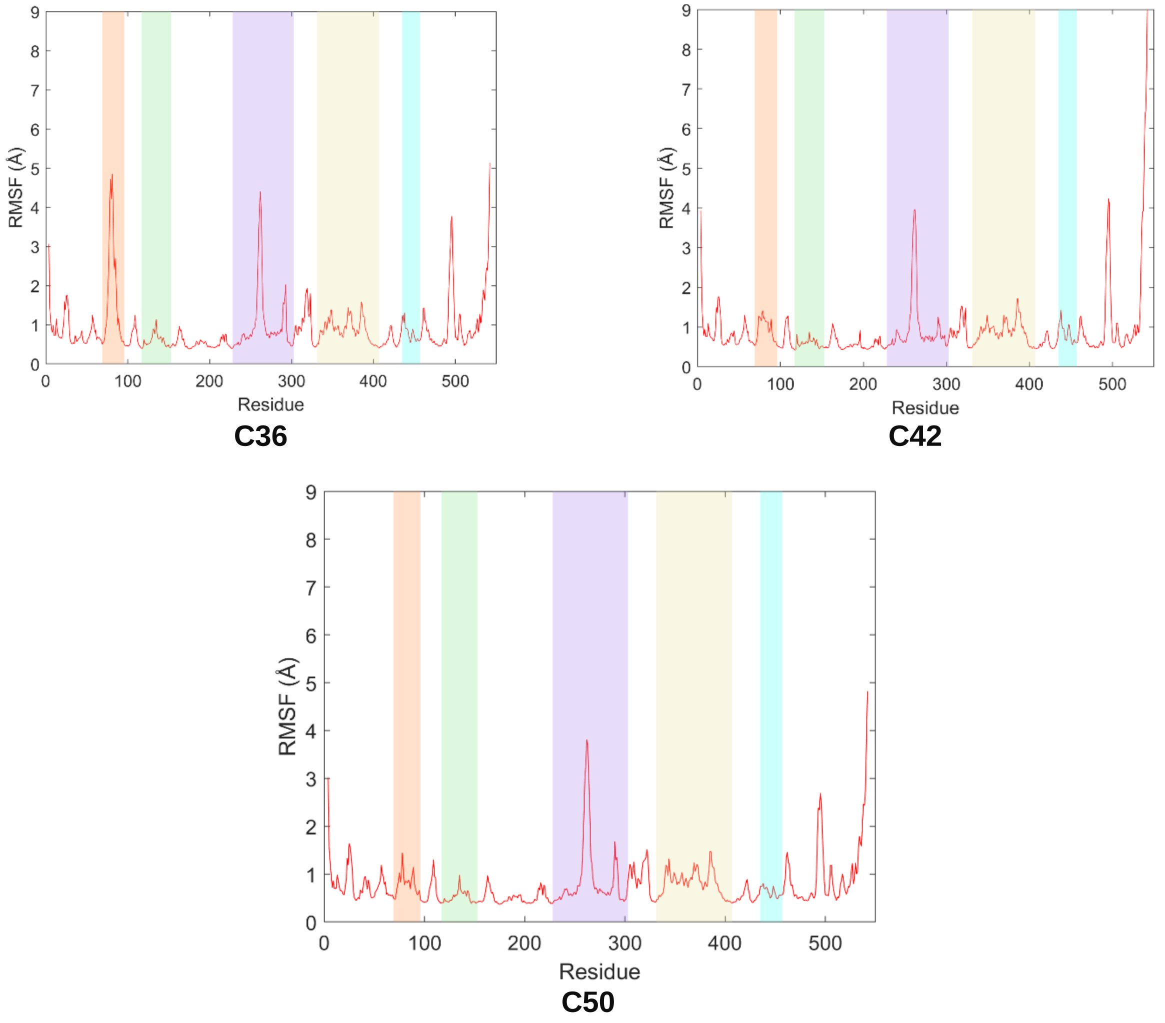

Figure S24. RMSF values for backbone of 500 ns MD simulations for C36, C42 and C50. Green for S1 domain (117-153), purple for S2 domain (228-303), yellow for S3 domain (331-407), cyan for S4 domain (435-457), and red for Ω-loop (69-96).

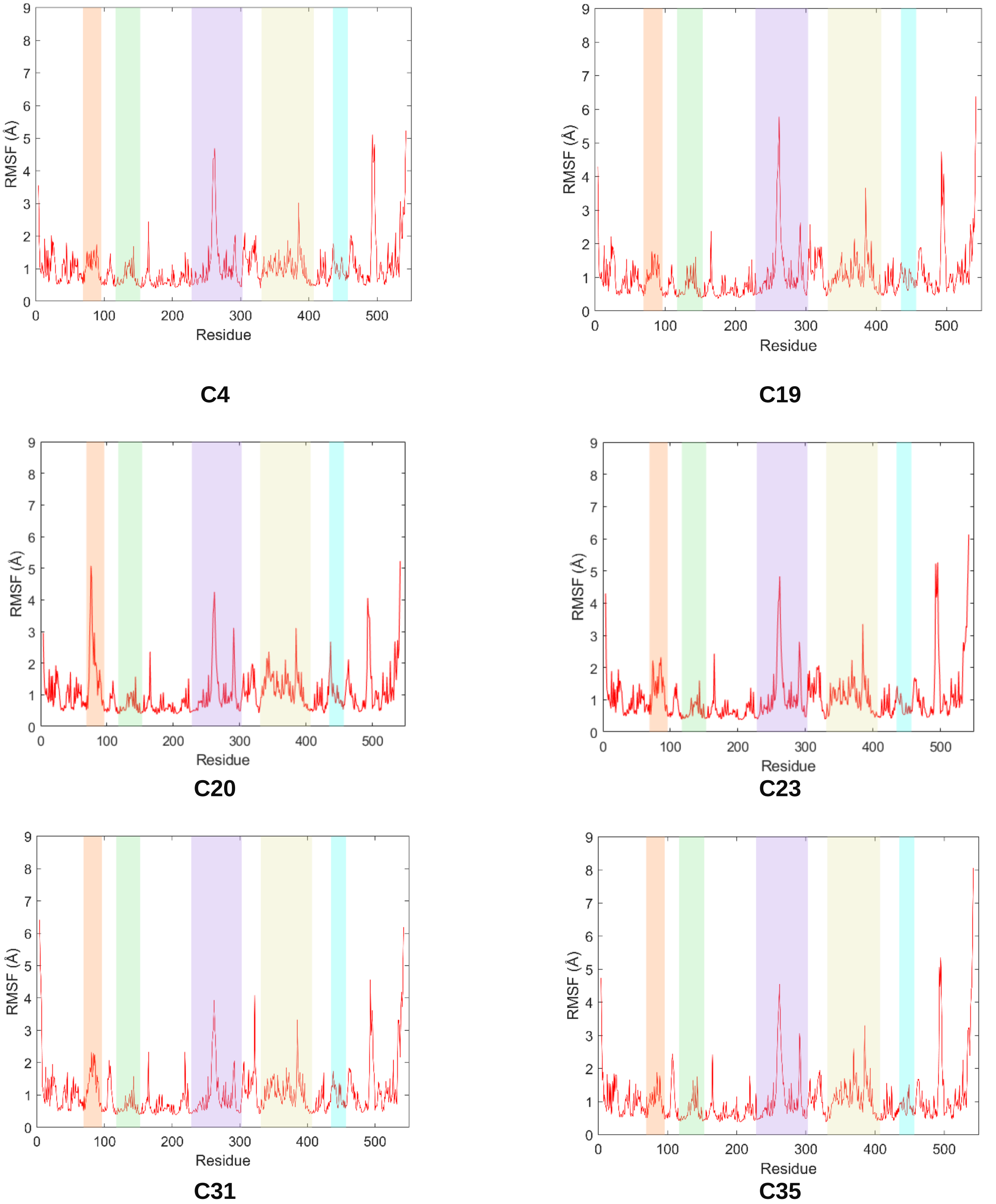

Figure S25. RMSF values for all heavy atoms (backbone and sidechain) of 500 ns MD simulations for C4, C19, C20, C23, C31 and C35. Green for S1 domain (117-153), purple for S2 domain (228-303), yellow for S3 domain (331-407), cyan for S4 domain (435-457), and red for Ω-loop (69-96).

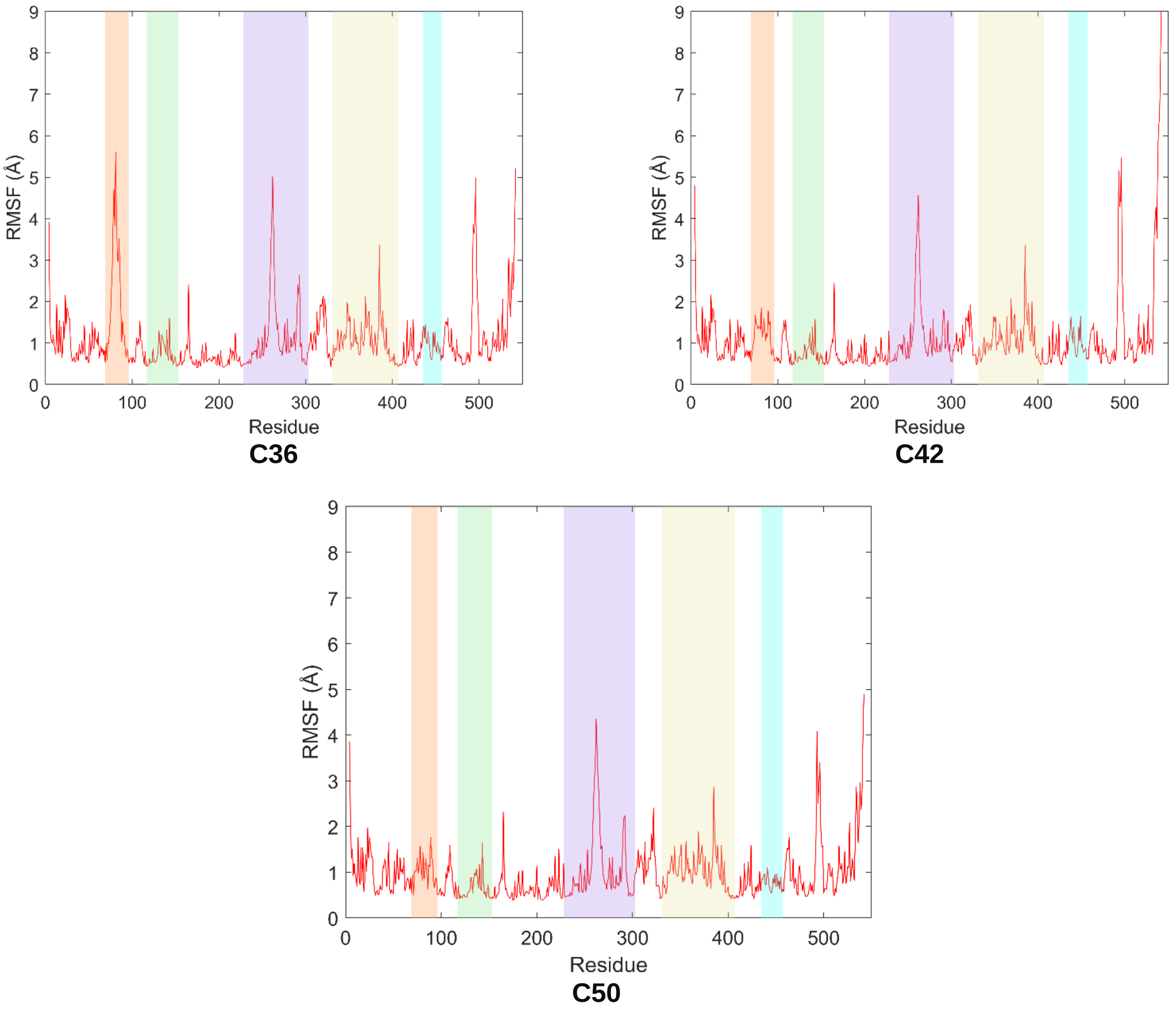

Figure S26. RMSF values for all heavy atoms (backbone and sidechain) of 500 ns MD simulations for C36, C42 and C50. Green for S1 domain (117-153), purple for S2 domain (228-303), yellow for S3 domain (331-407), cyan for S4 domain (435-457), and red for Ω-loop (69-96).

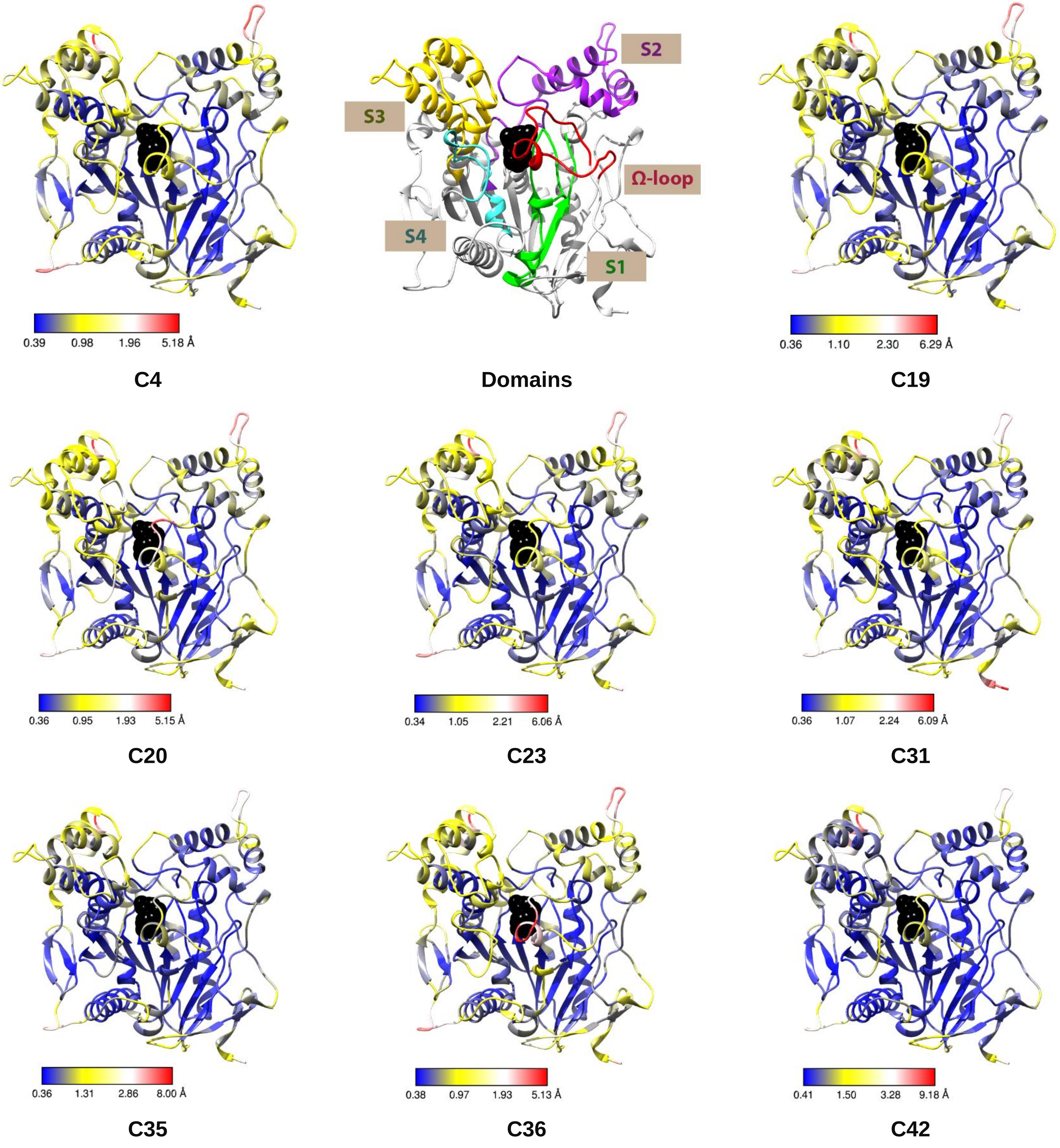

Figure S27. RMSF backbone values for 500 ns MD simulation applied on huAChE ribbons representation for C4, C19, C20, C23, C31, C35, C36, and C42 simulations. 4EY6 crystal.

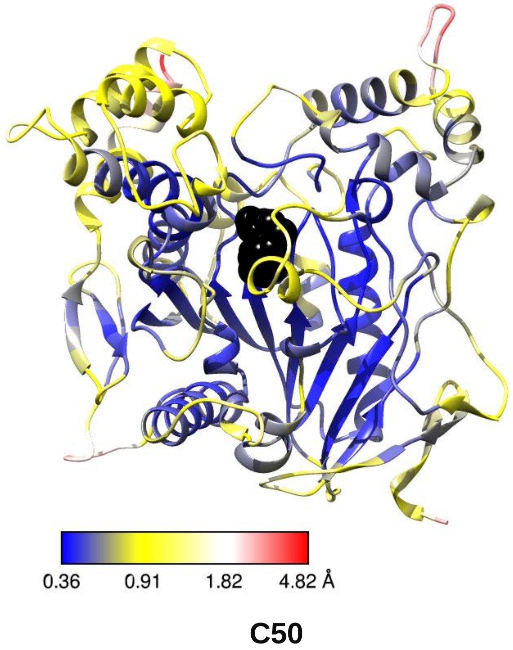

Figure S28. RMSF backbone values for 500 ns MD simulation applied on huAChE ribbons representation for C50 simulation. 4EY6 crystal.

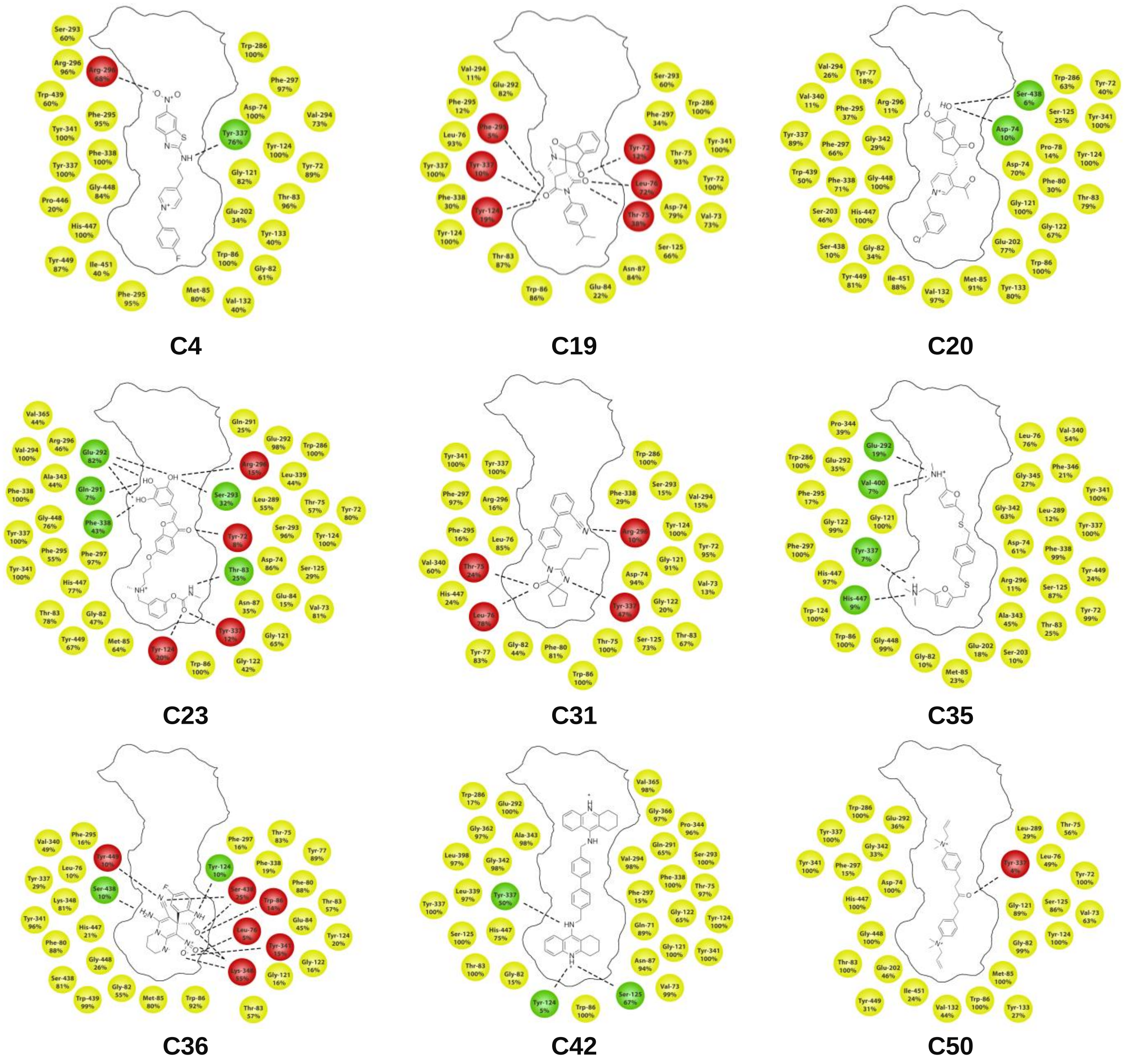

Figure S29. Protein-ligand interaction diagrams with the percentage frequency of interaction occurrences. Hydrophobic contacts are shown in yellow, H-bond donations in green, and H-bond acceptances in red.

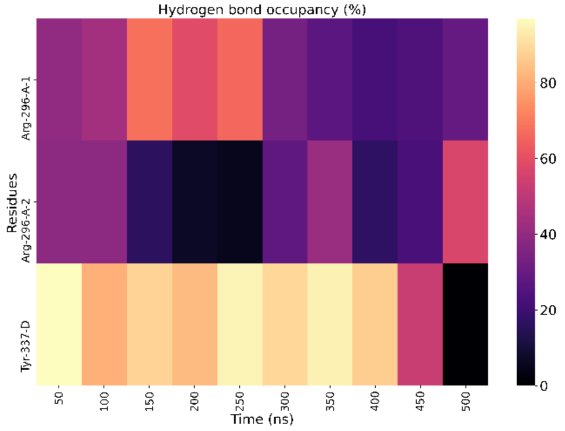

C4

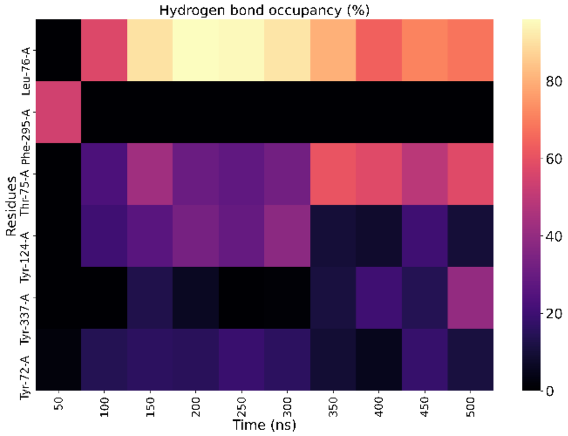

C19

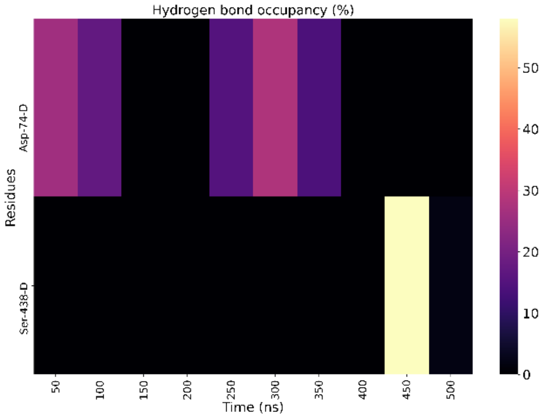

C20

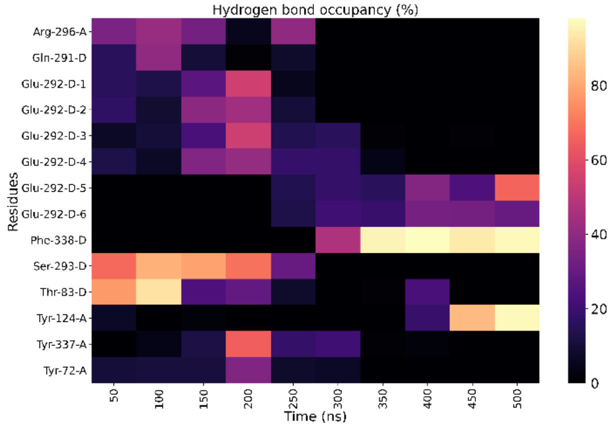

C23

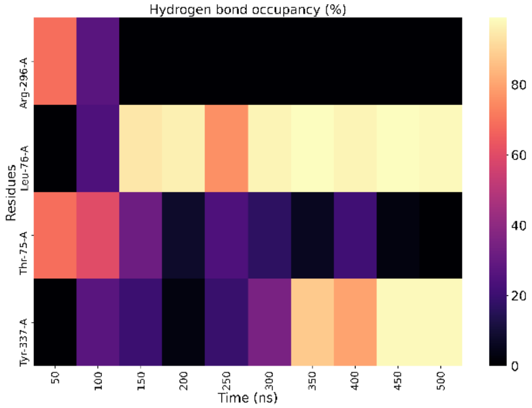

C31

Figure S30. Ligand Hydrogen bond occupancy (%) heatmap for 500 ns MD simulations of C4, C19, C20, C23, and C31.

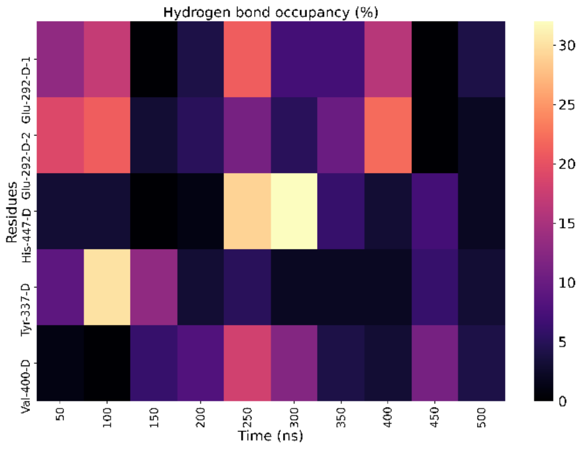

C35

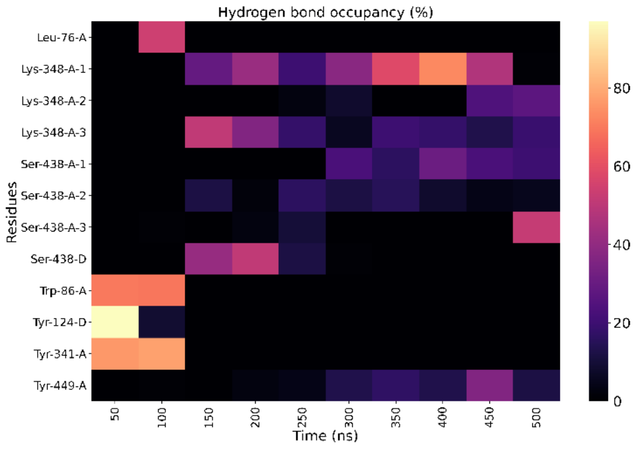

C36

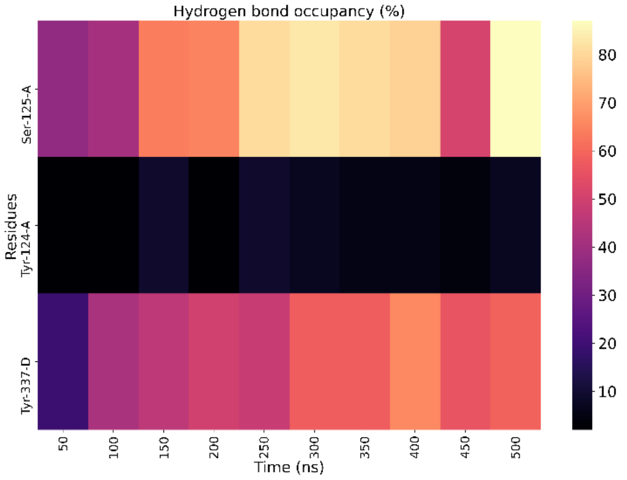

C42

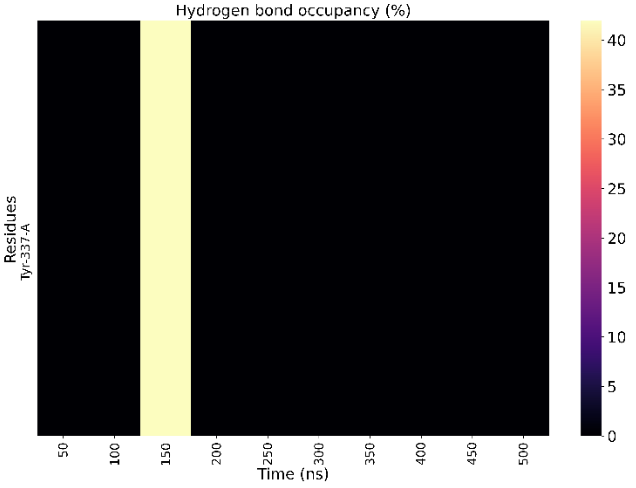

C50

Figure S31. Ligand Hydrogen bond occupancy (%) heatmap for 500 ns MD simulations of C35, C36, C42, and C50.

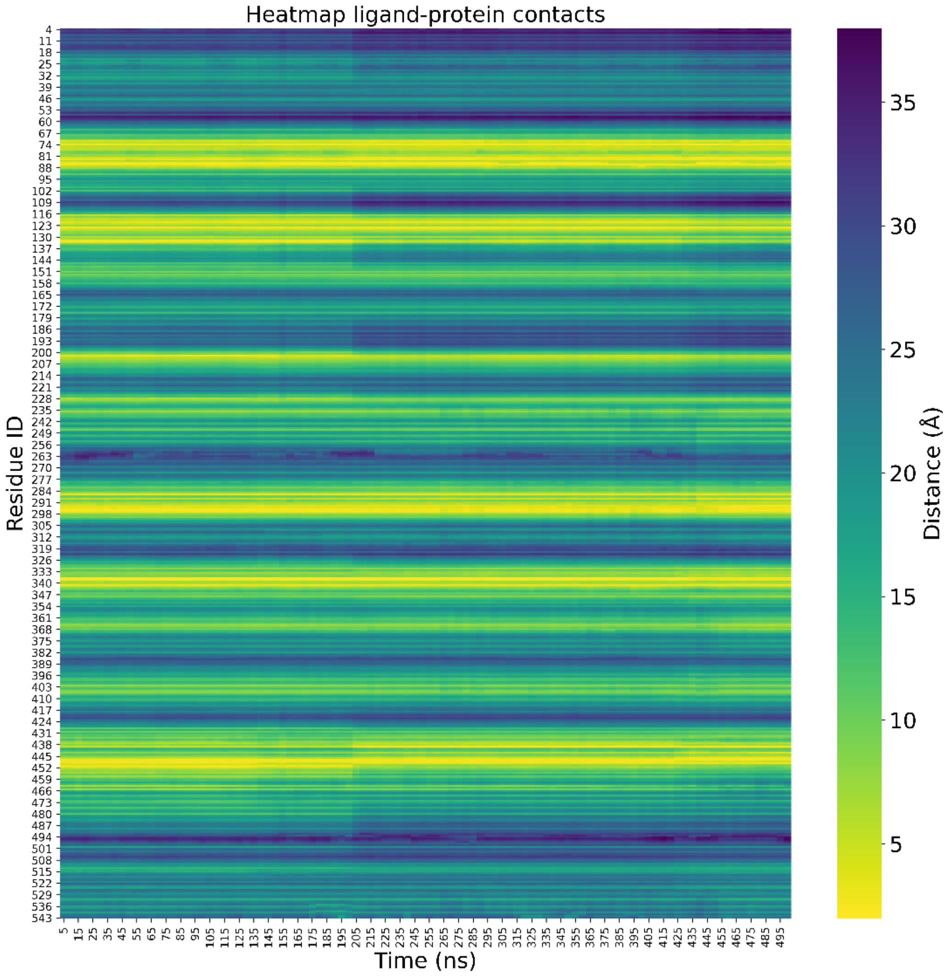

Figure S32. Protein-ligand contacts heatmap of all residues for 500 ns MD simulation of C4.

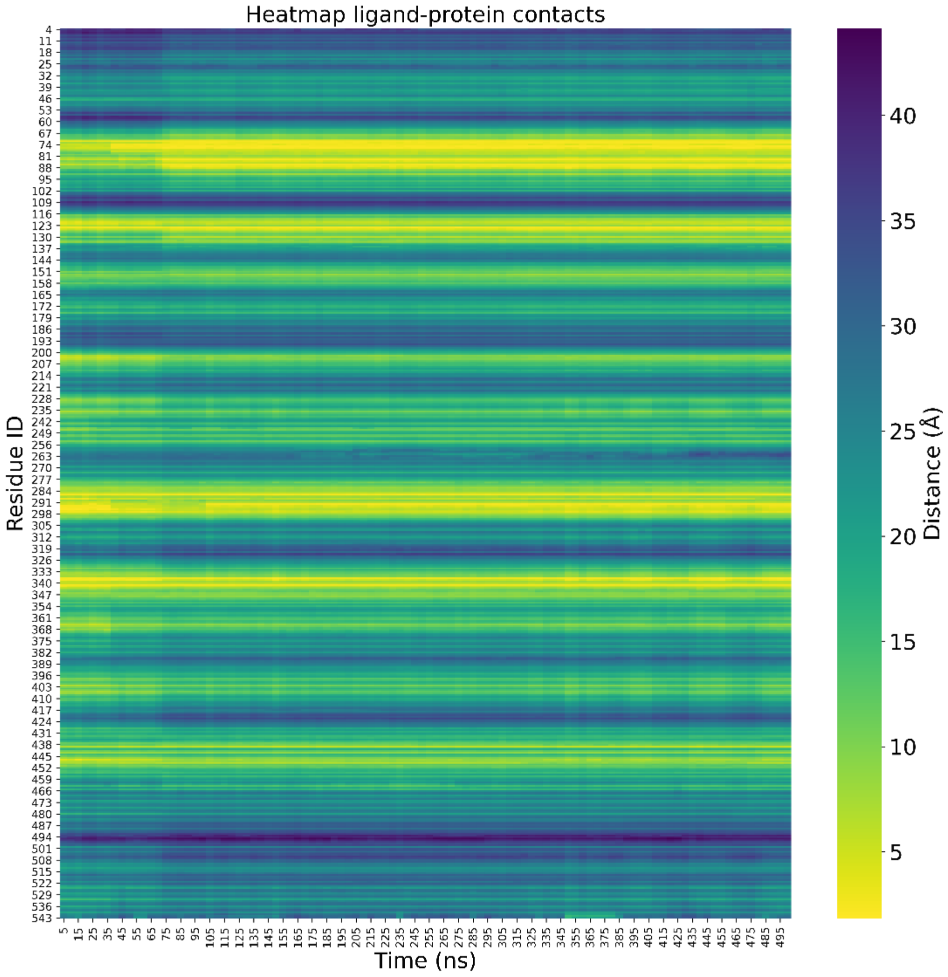

Figure S33. Protein-ligand contacts heatmap of all residues for 500 ns MD simulation of C19.

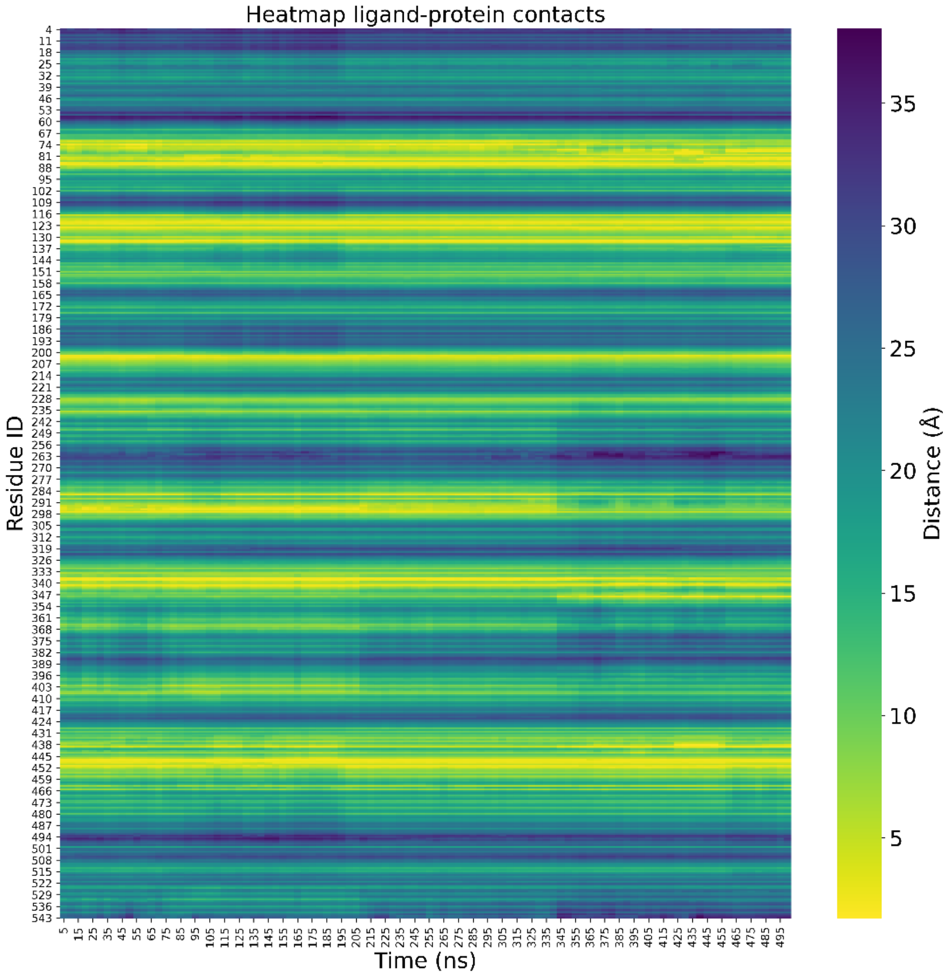

Figure S34. Protein-ligand contacts heatmap of all residues for 500 ns MD simulation of C20.

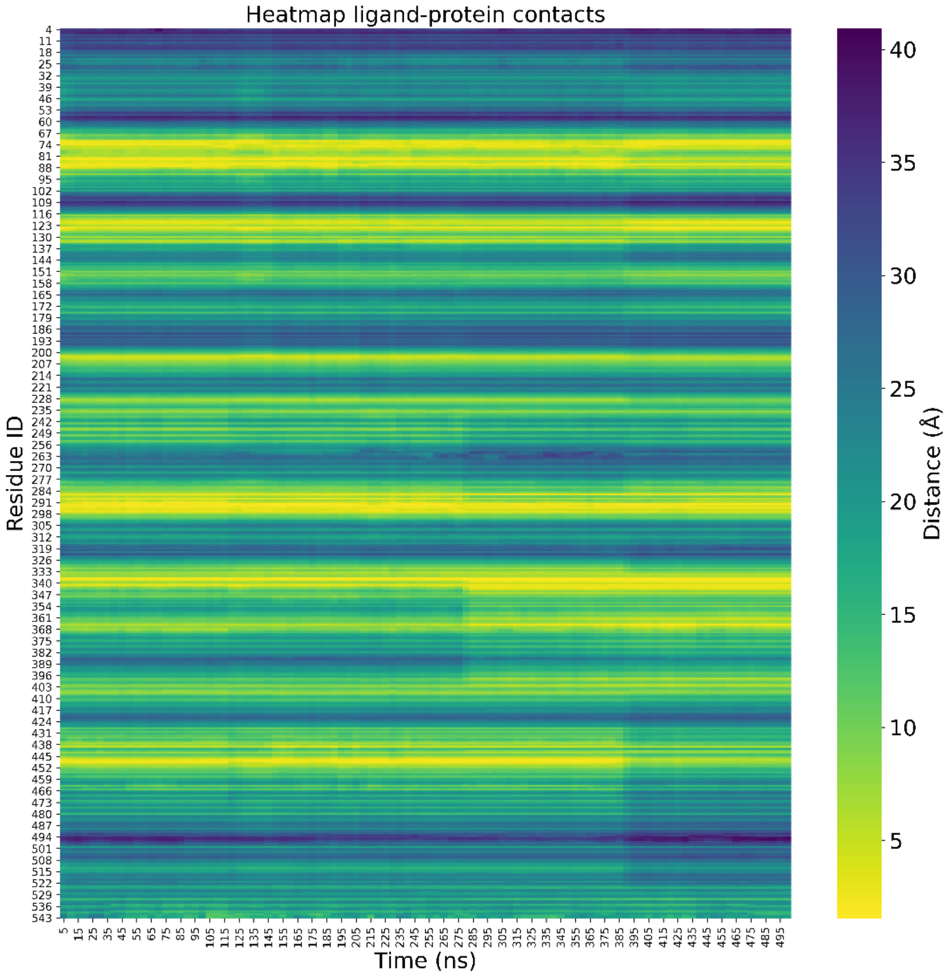

Figure S35. Protein-ligand contacts heatmap of all residues for 500 ns MD simulation of C23.

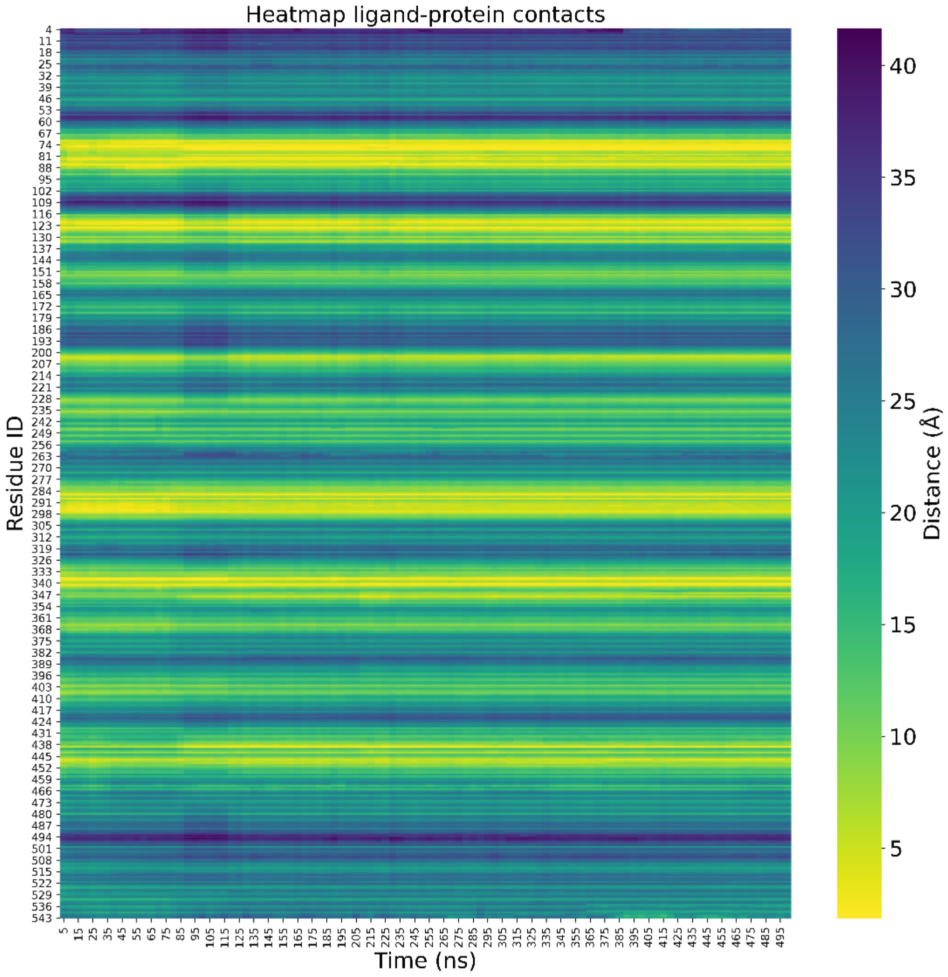

Figure S36. Protein-ligand contacts heatmap of all residues for 500 ns MD simulation of C31.

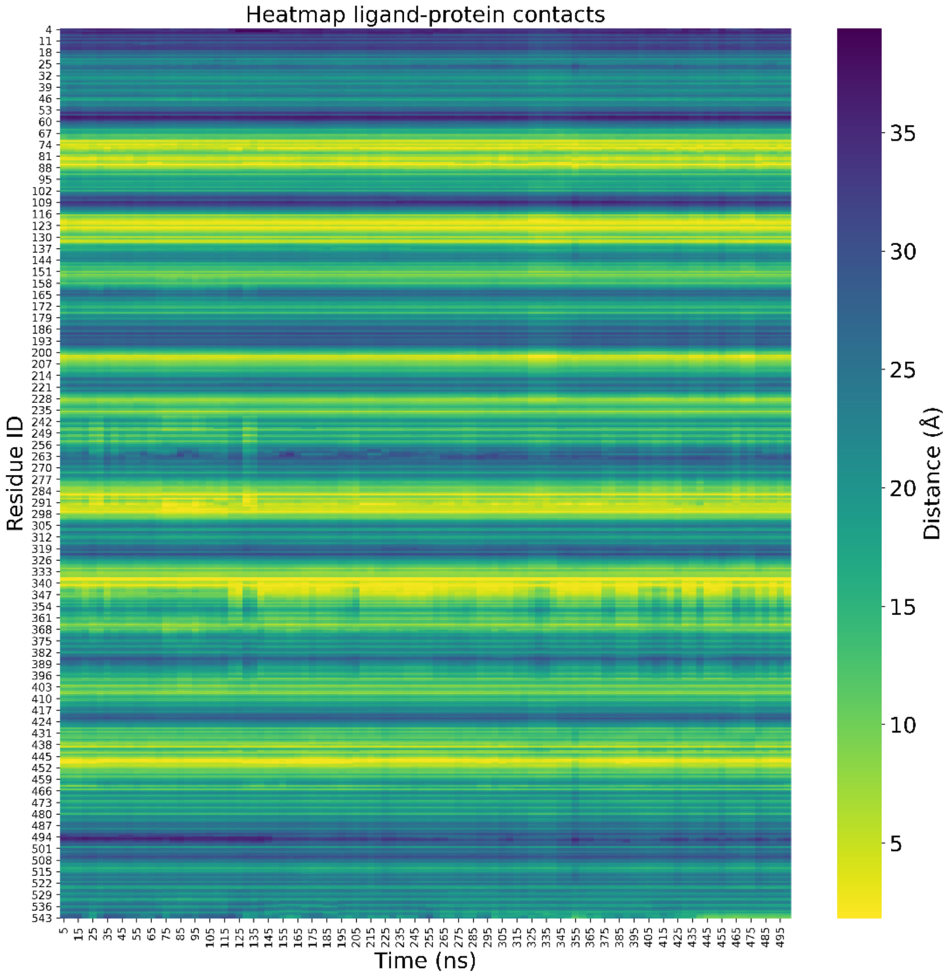

Figure S37. Protein-ligand contacts heatmap of all residues for 500 ns MD simulation of C35.

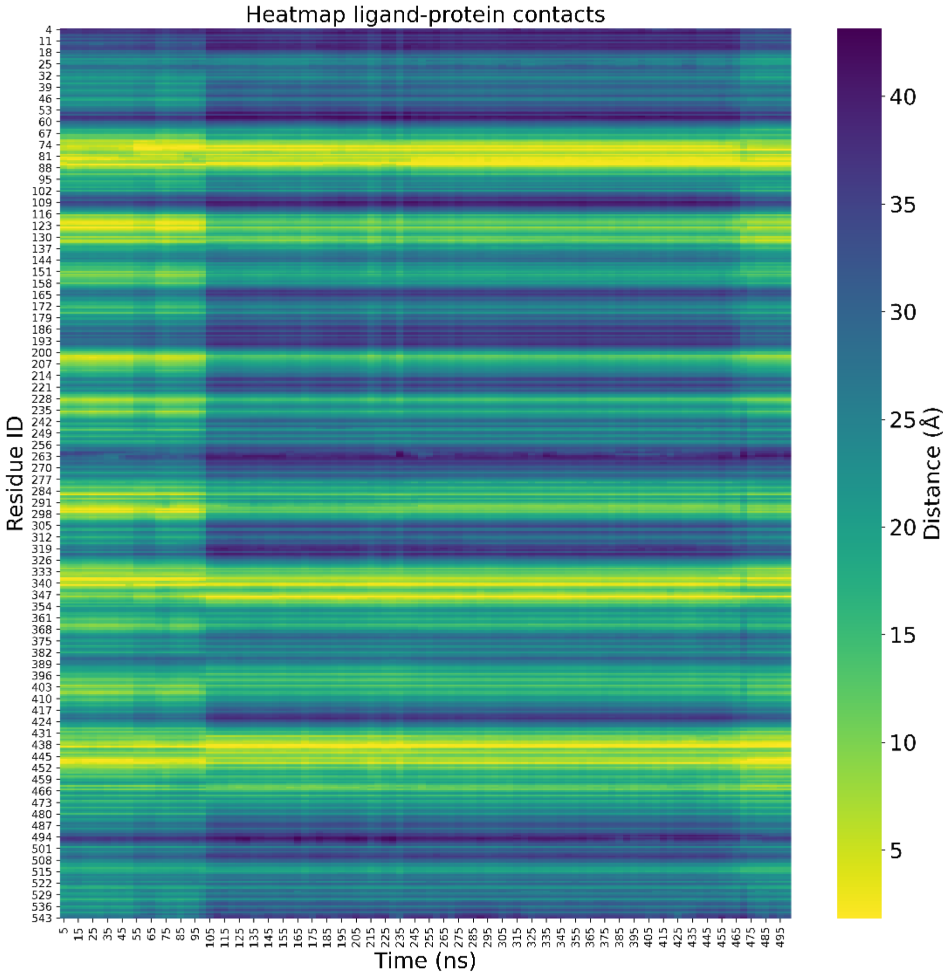

Figure S38. Protein-ligand contacts heatmap of all residues for 500 ns MD simulation of C36.

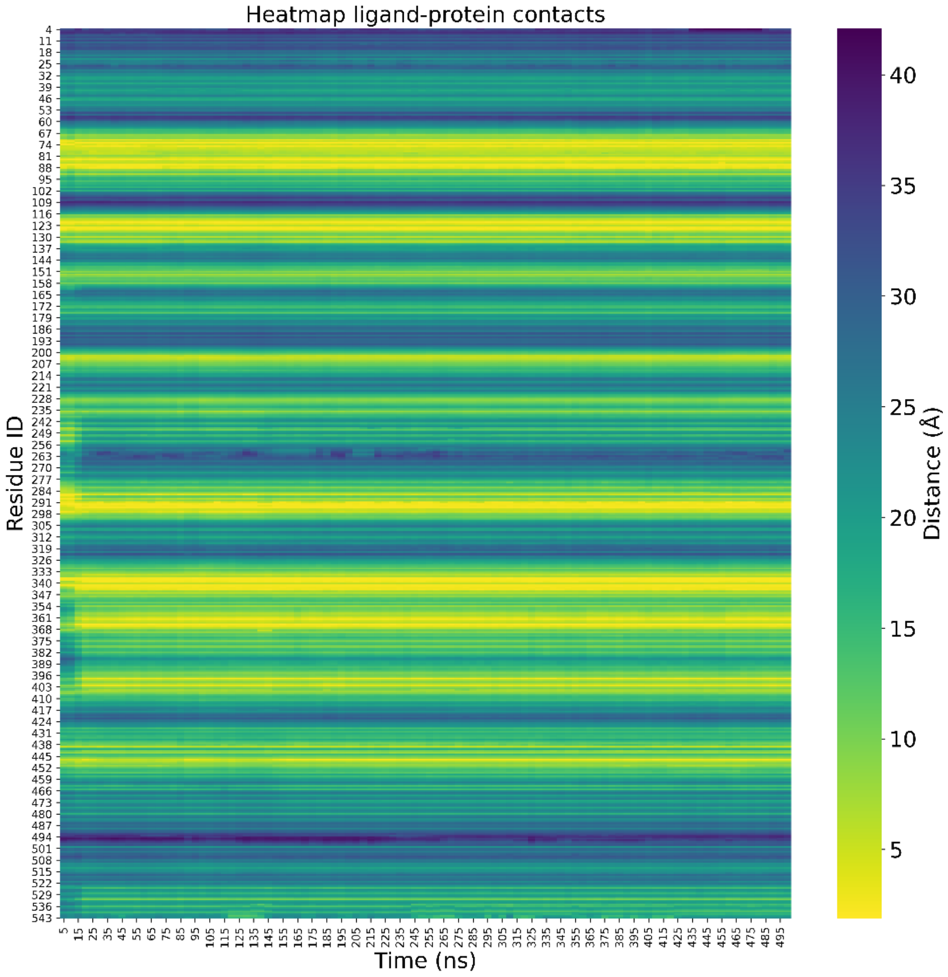

Figure S39. Protein-ligand contacts heatmap of all residues for 500 ns MD simulation of C42.

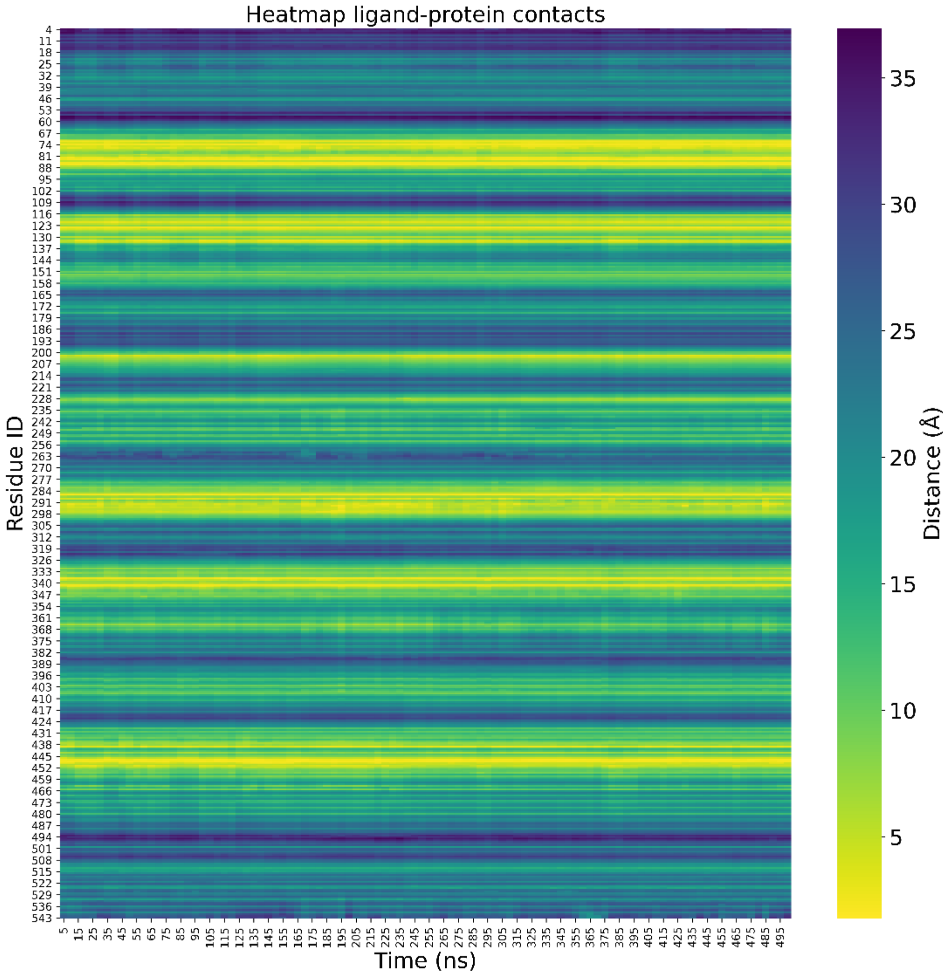

Figure S40. Protein-ligand contacts heatmap of all residues for 500 ns MD simulation of C50.

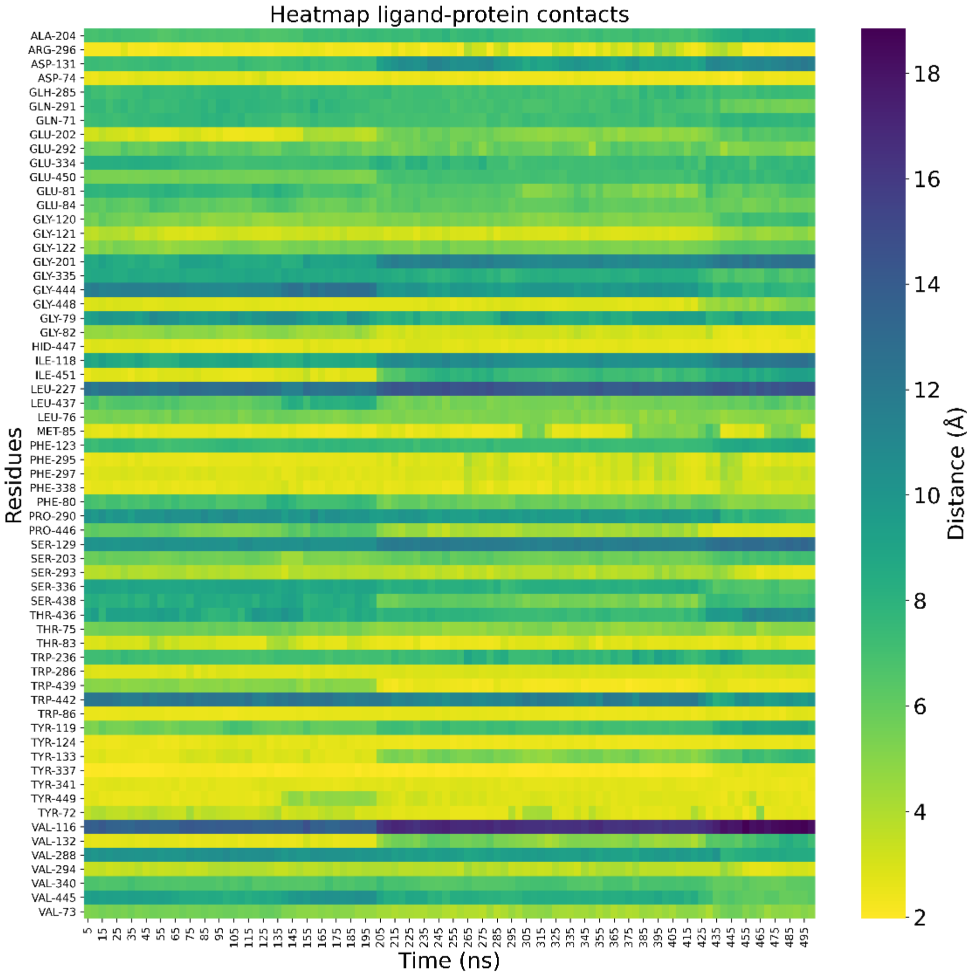

Figure S41. Protein-ligand contacts heatmap for residues that were within 5 angstroms of the ligand at some point during the simulation of 500 ns MD simulation of C4.

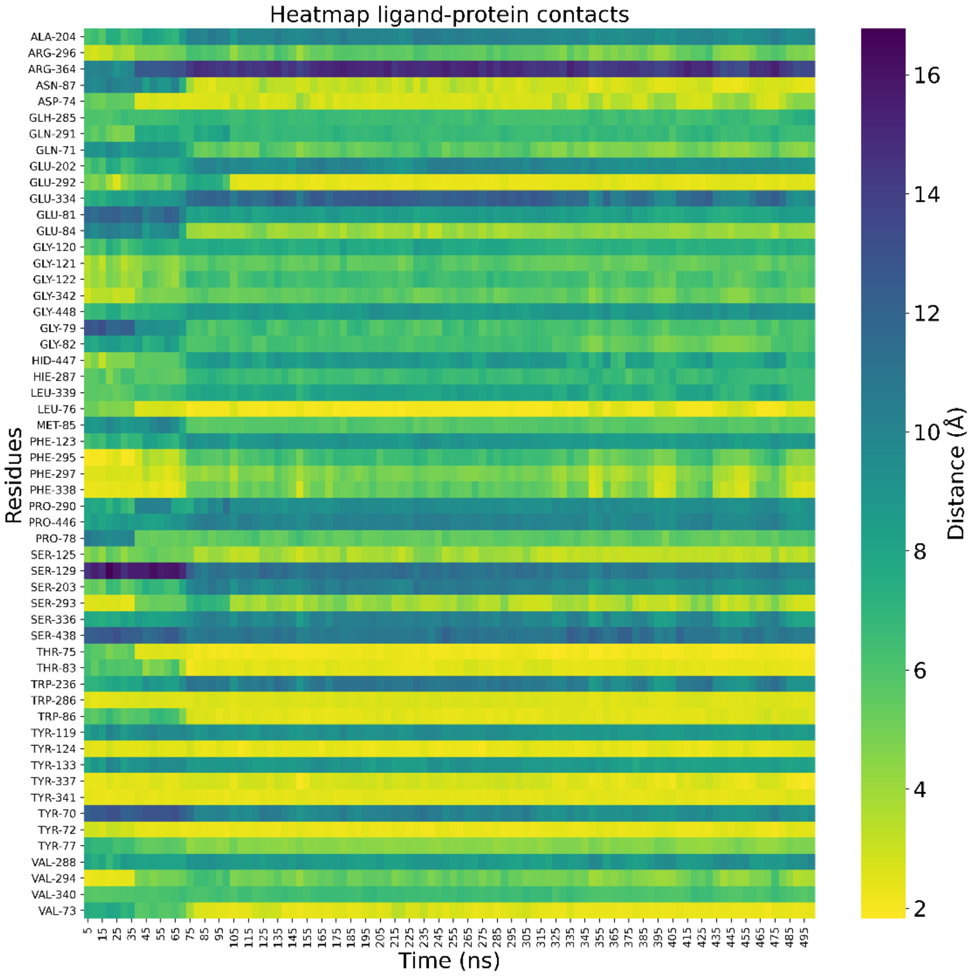

Figure S42. Protein-ligand contacts heatmap for residues that were within 5 angstroms of the ligand at some point during the simulation of 500 ns MD simulation of C19.

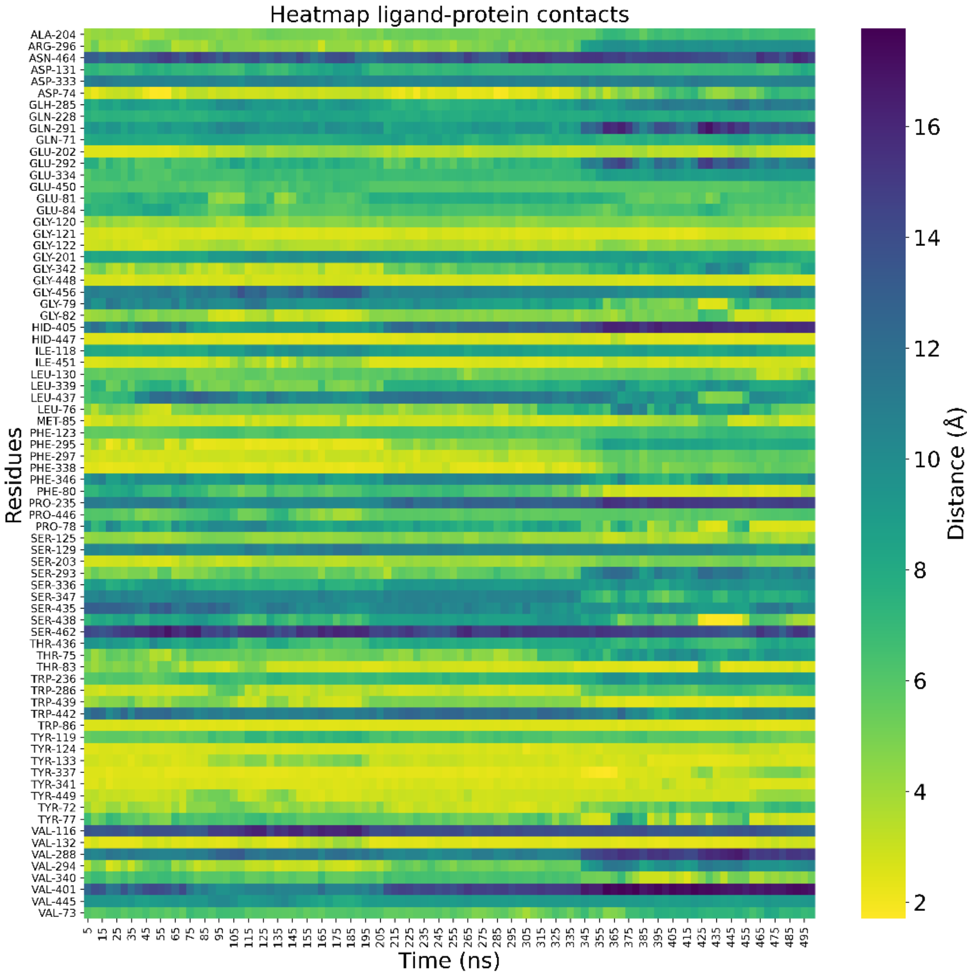

Figure S43. Protein-ligand contacts heatmap for residues that were within 5 angstroms of the ligand at some point during the simulation of 500 ns MD simulation of C20.

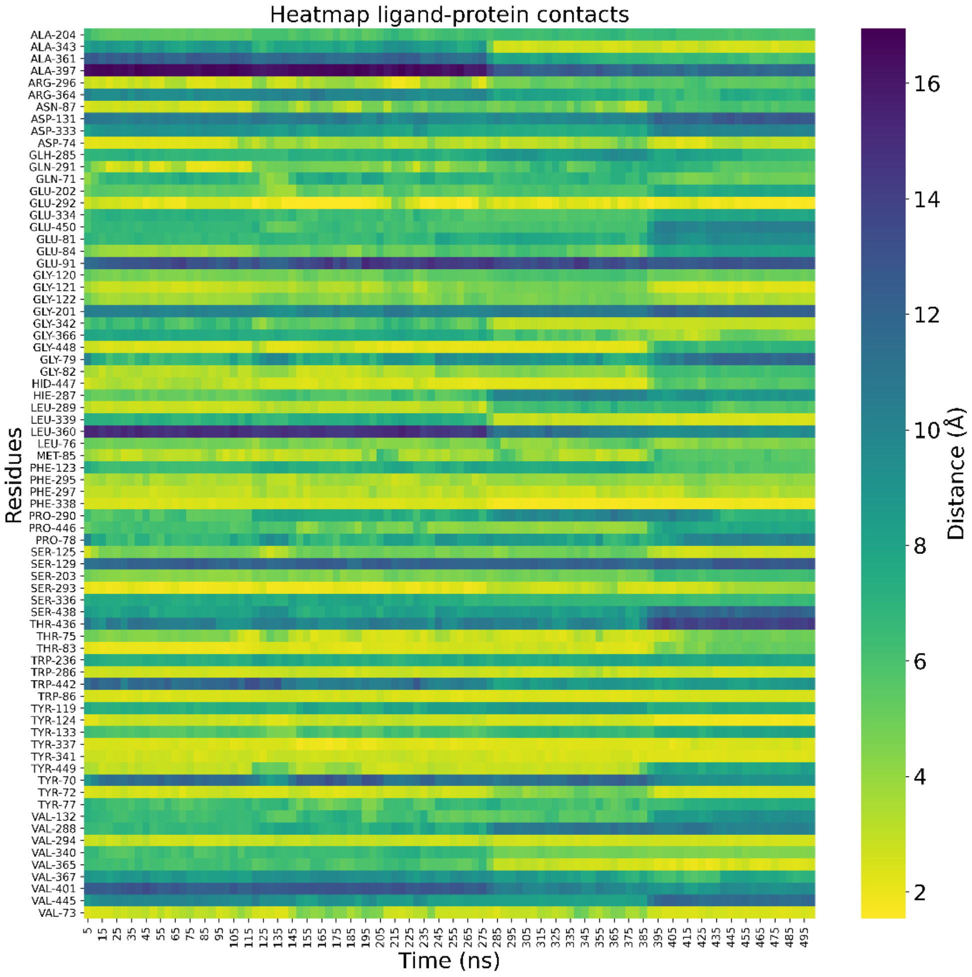

Figure S44. Protein-ligand contacts heatmap for residues that were within 5 angstroms of the ligand at some point during the simulation of 500 ns MD simulation of C23.

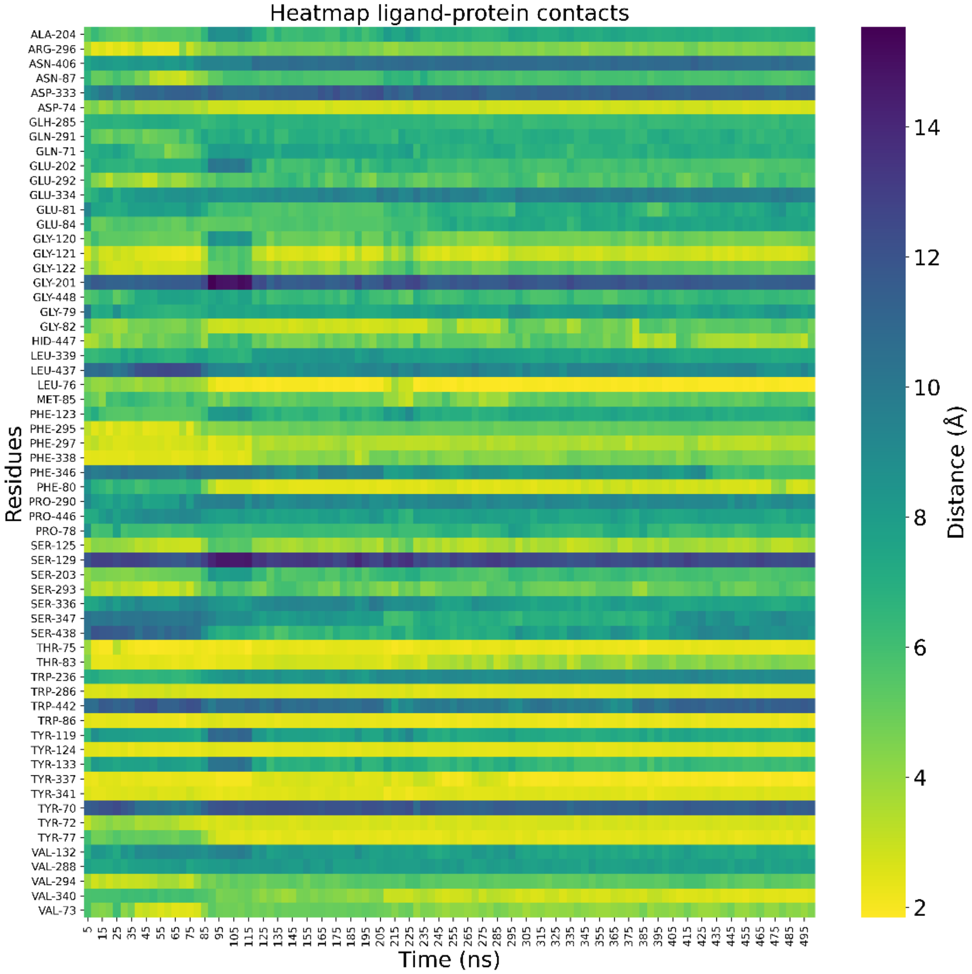

Figure S45. Protein-ligand contacts heatmap for residues that were within 5 angstroms of the ligand at some point during the simulation of 500 ns MD simulation of C31.

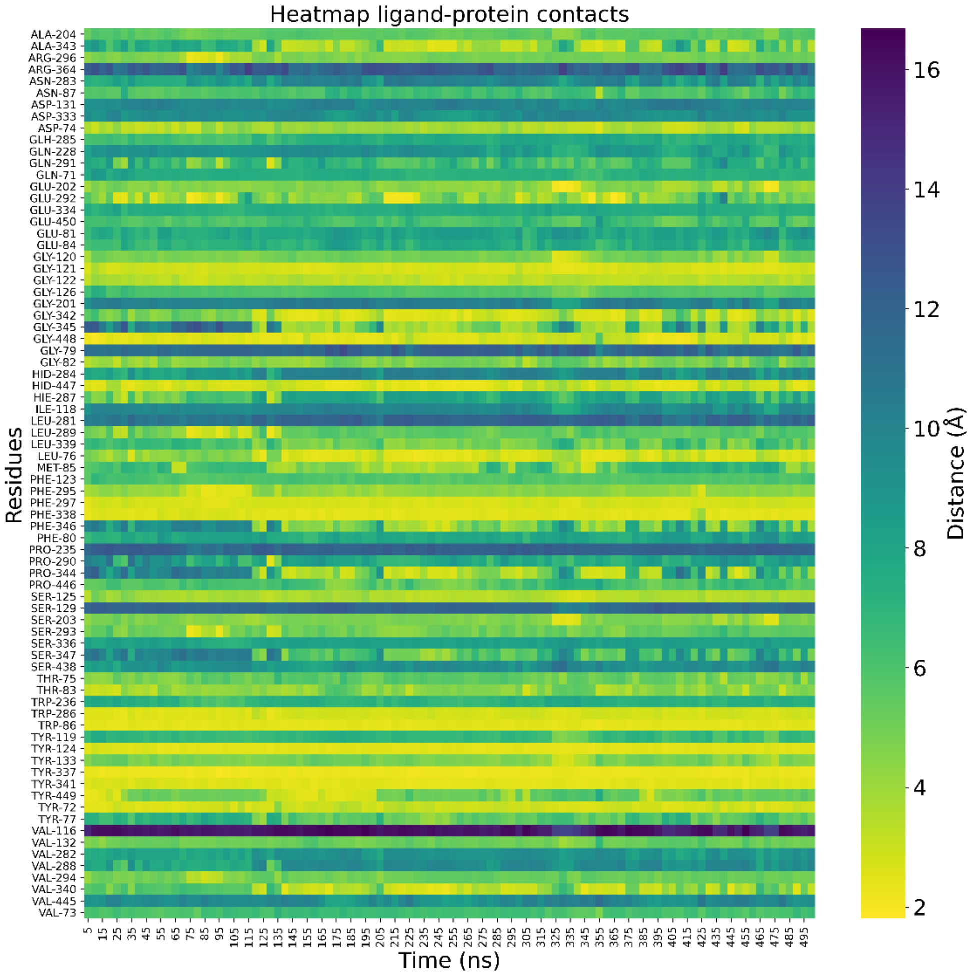

Figure S46. Protein-ligand contacts heatmap for residues that were within 5 angstroms of the ligand at some point during the simulation of 500 ns MD simulation of C35.

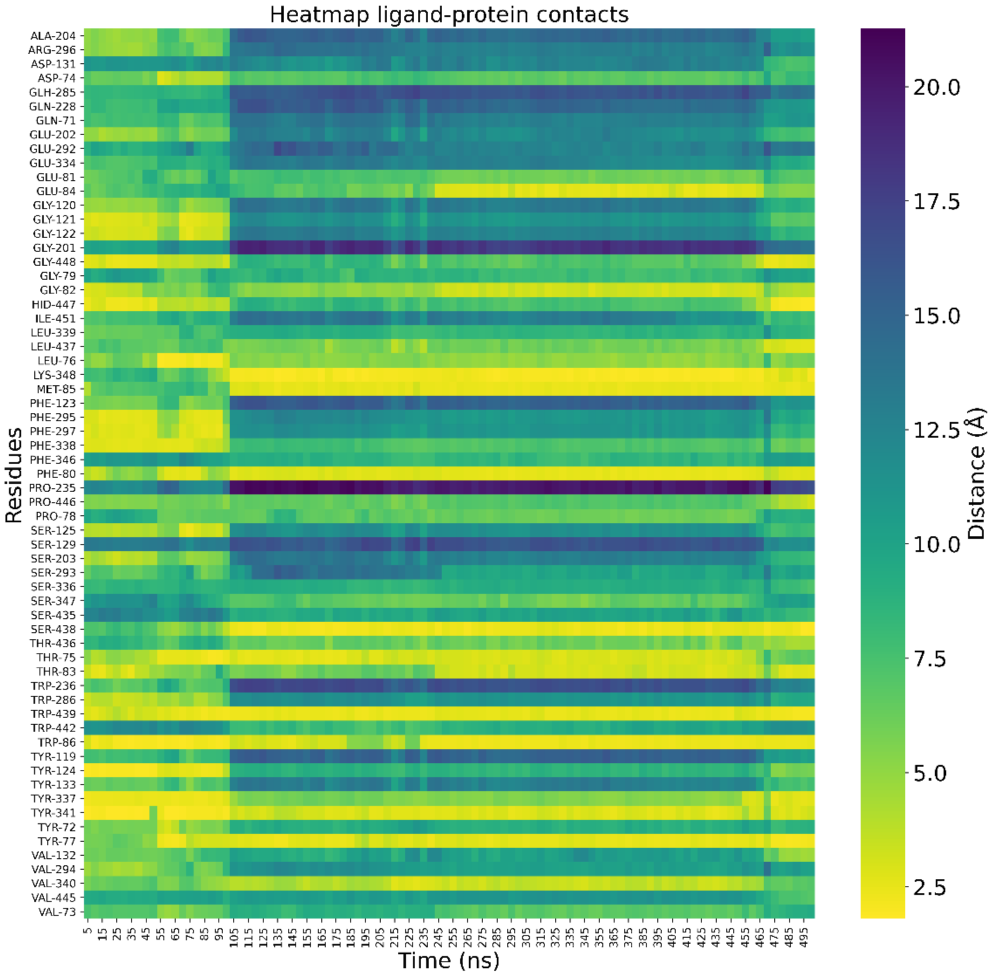

Figure S47. Protein-ligand contacts heatmap for residues that were within 5 angstroms of the ligand at some point during the simulation of 500 ns MD simulation of C36.

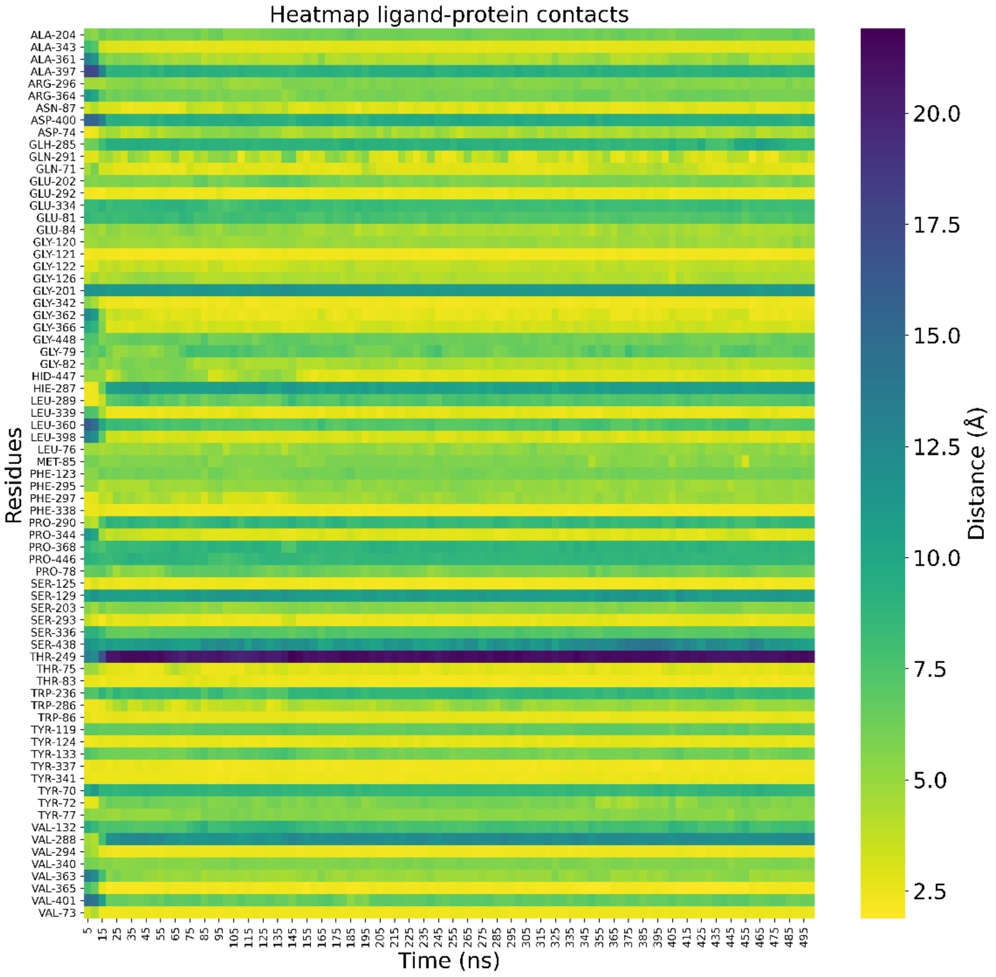

Figure S48. Protein-ligand contacts heatmap for residues that were within 5 angstroms of the ligand at some point during the simulation of 500 ns MD simulation of C42.

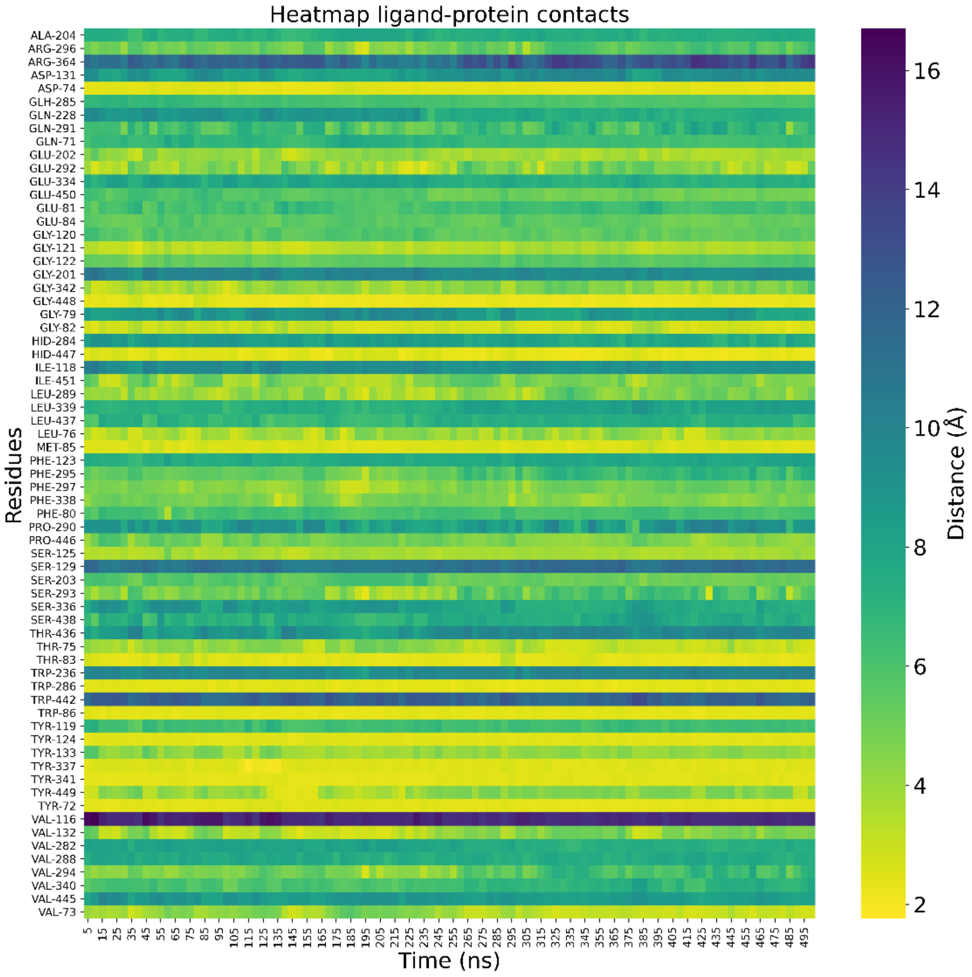

Figure S49. Protein-ligand contacts heatmap for residues that were within 5 angstroms of the ligand at some point during the simulation of 500 ns MD simulation of C50.

Table S17. Heavy atoms RMSF values of the 6 active site residues with highest flexibility.

C4	RMSF (Å)
ASP-74	1.10
HID-447	1.14
TRP-86	1.24
TYR-341	1.24
GLY-448	1.36
GLU-334	1.38

C19	RMSF (Å)
PHE-338	0.98
PHE-295	1.10
GLY-448	1.10
TRP-86	1.23
ASP-74	1.23
HID-447	1.26

C20	RMSF (Å)
GLU-334	1.29
HID-447	1.30
TYR-337	1.44
PHE-338	1.58
TYR-341	2.16
ASP-74	2.86

C23	RMSF (Å)
TYR-72	0.83
PHE-338	0.99
TYR-341	1.39
TYR-337	1.53
ASP-74	2.21
TRP-86	2.32

C31	RMSF (Å)
GLY-448	0.99
ASP-74	1.18
TYR-337	1.28
HID-447	1.38
TYR-341	1.51
TRP-86	2.24

C35	RMSF (Å)
PHE-338	0.95
ASP-74	1.06
GLY-448	1.10
TRP-86	1.20
TYR-341	1.25
HID-447	1.30

C36	RMSF (Å)
PHE-338	1.15
HID-447	1.27
TYR-341	1.30
TYR-337	1.33
ASP-74	1.37
TRP-86	2.67

C42	RMSF (Å)
TYR-72	1.01
GLY-448	1.04
TRP-86	1.05
PHE-338	1.14
HID-447	1.39
ASP-74	1.67

C50	RMSF (Å)
HID-447	0.86
PHE-338	0.98
TYR-72	0.99
ASP-74	1.03
TYR-337	1.04
TYR-341	1.37

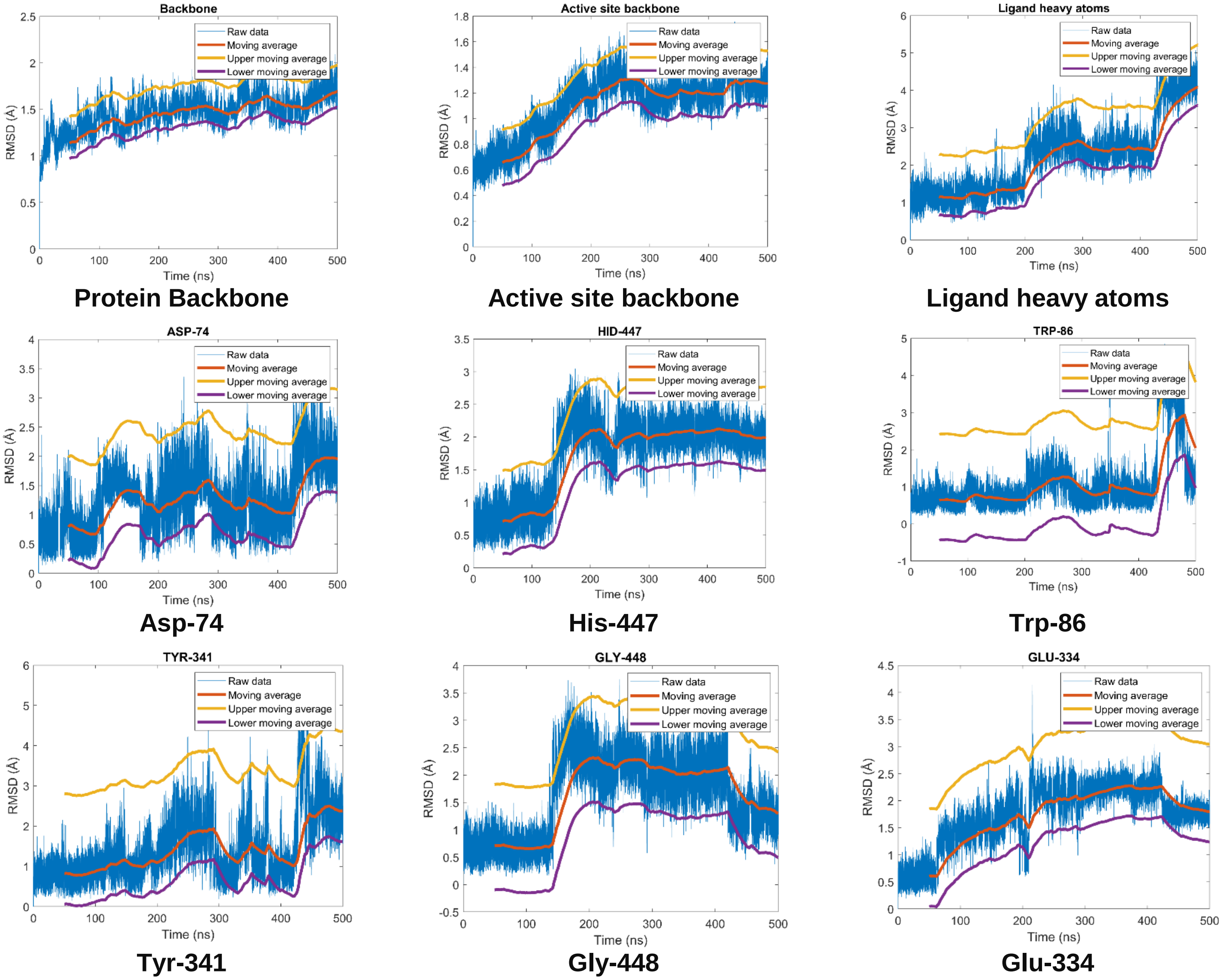

Figure S50. Heavy atoms RMSD for the 6 active site residues, backbone RMSD for the whole protein, backbone RMSD for the 17 active site residues and RMSD of ligand heavy atoms for C4.

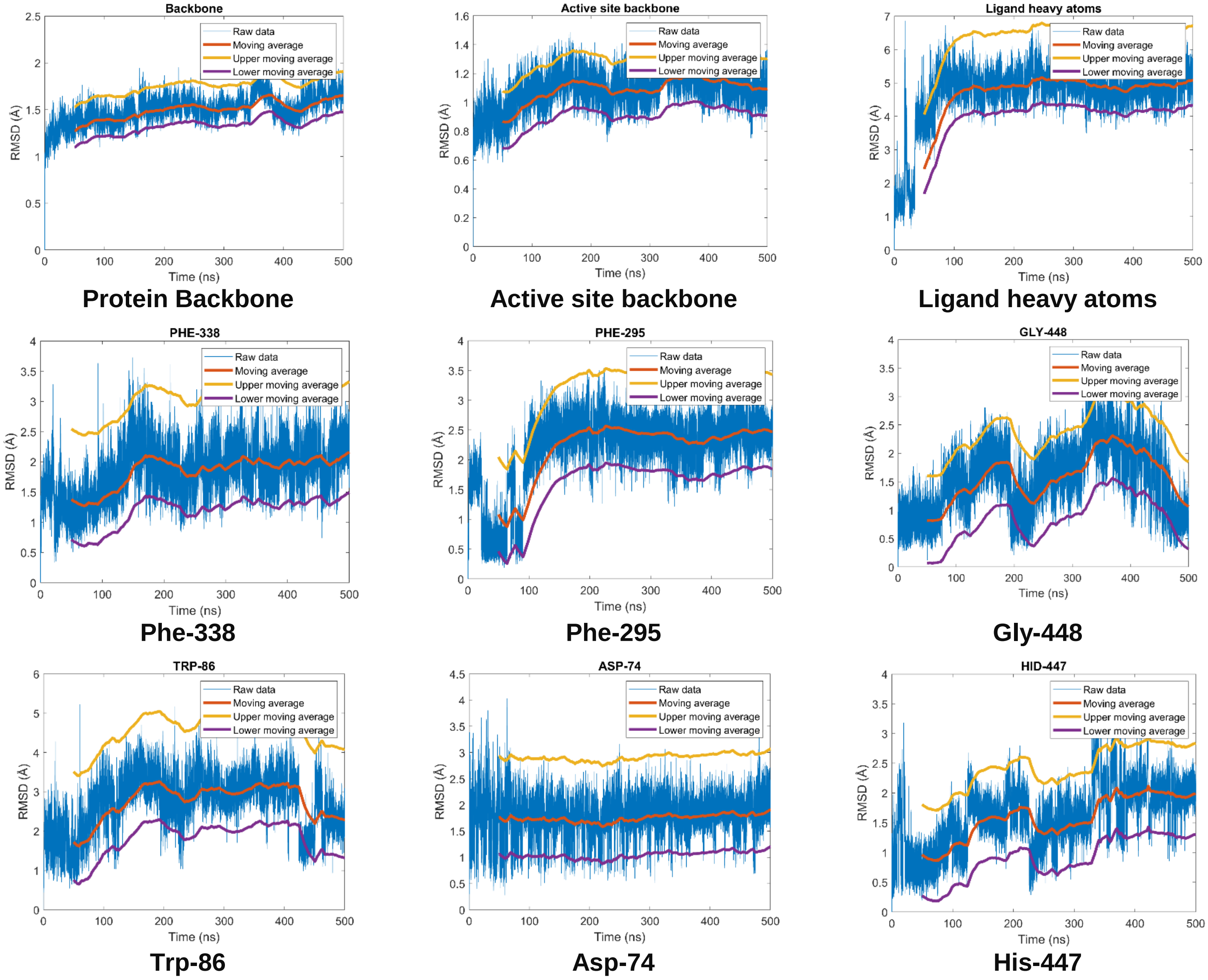

Figure S51. Heavy atoms RMSD for the 6 active site residues, backbone RMSD for the whole protein, backbone RMSD for the 17 active site residues and RMSD of ligand heavy atoms for C19.

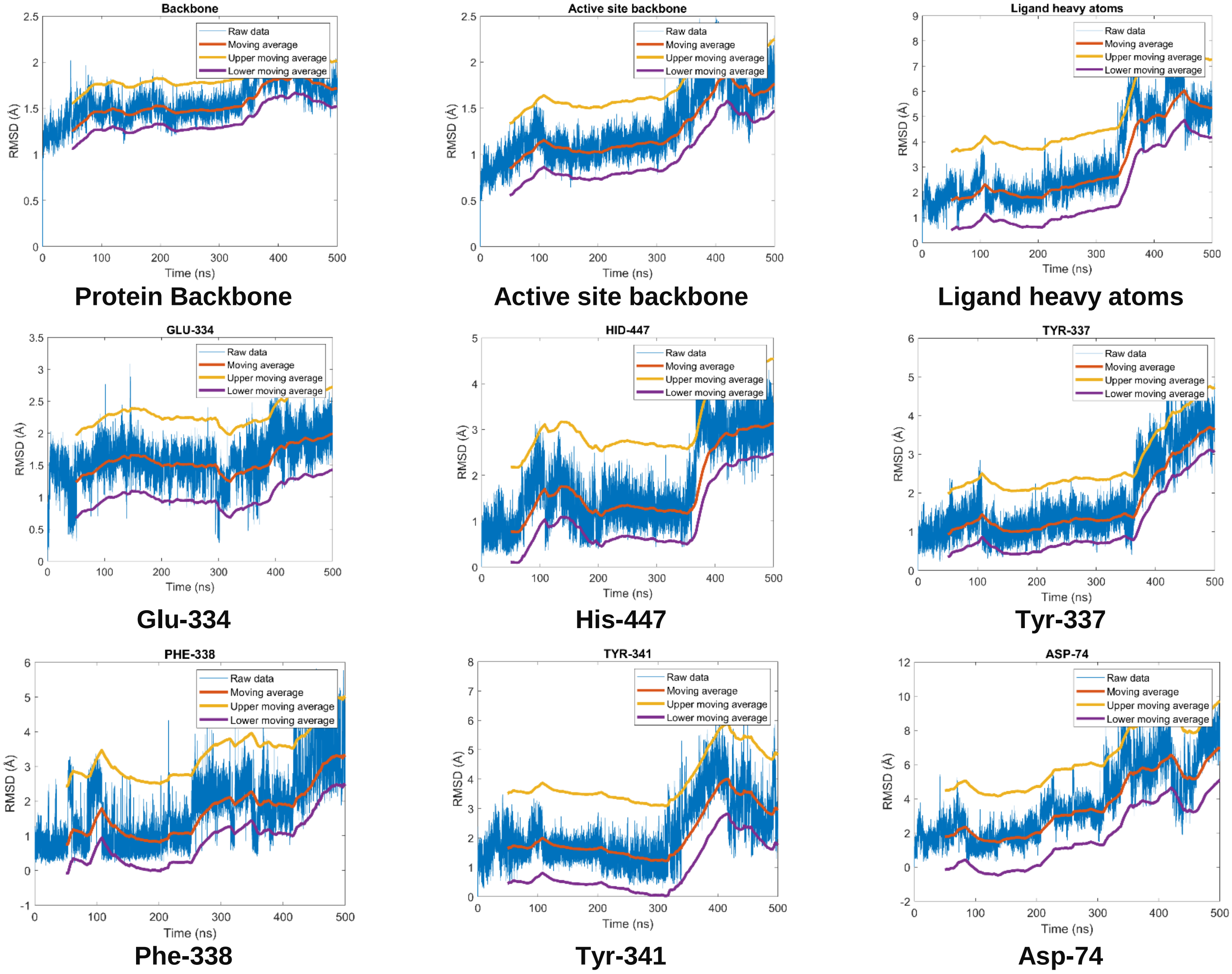

Figure S52. Heavy atoms RMSD for the 6 active site residues, backbone RMSD for the whole protein, backbone RMSD for the 17 active site residues and RMSD of ligand heavy atoms for C20.

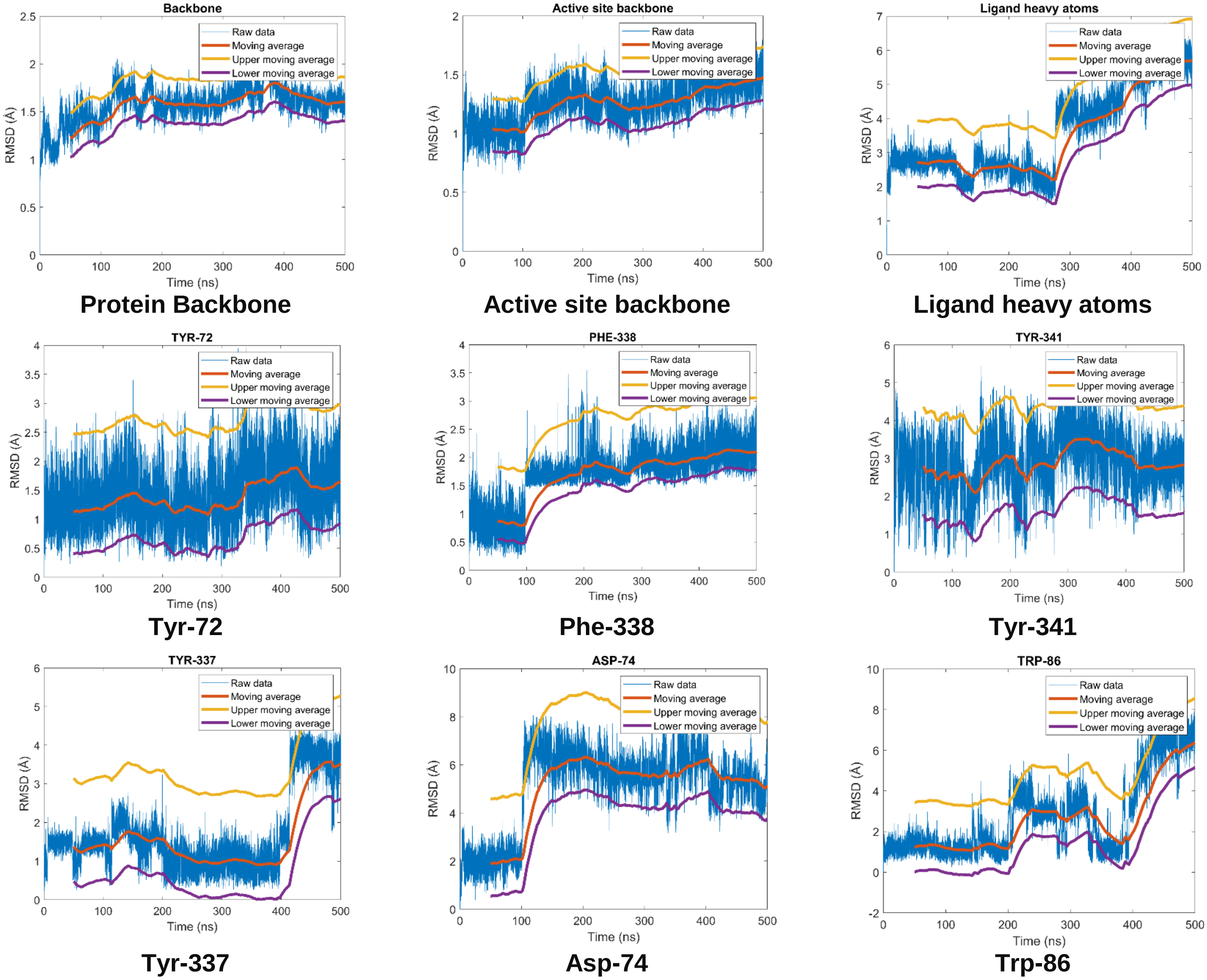

Figure S53. Heavy atoms RMSD for the 6 active site residues, backbone RMSD for the whole protein, backbone RMSD for the 17 active site residues and RMSD of ligand heavy atoms for C23.

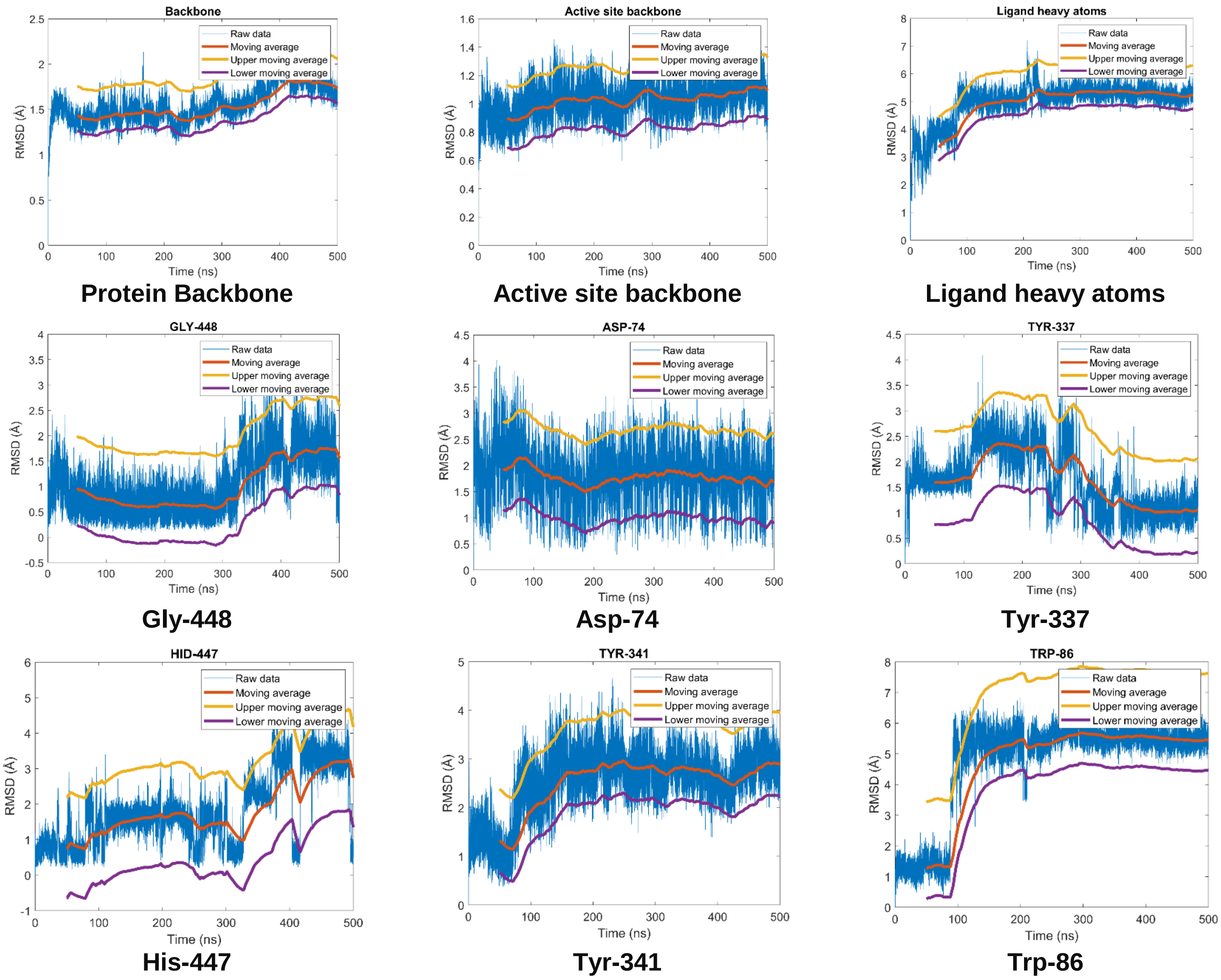

Figure S54. Heavy atoms RMSD for the 6 active site residues, backbone RMSD for the whole protein, backbone RMSD for the 17 active site residues and RMSD of ligand heavy atoms for C31.

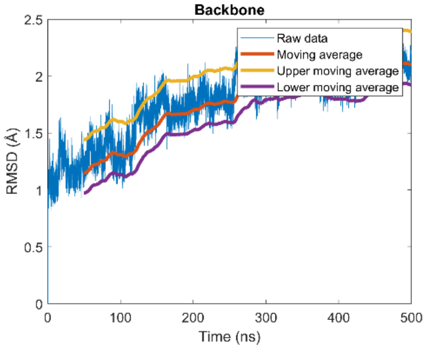

Protein Backbone

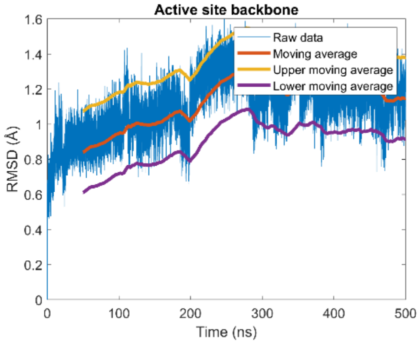

Active site backbone

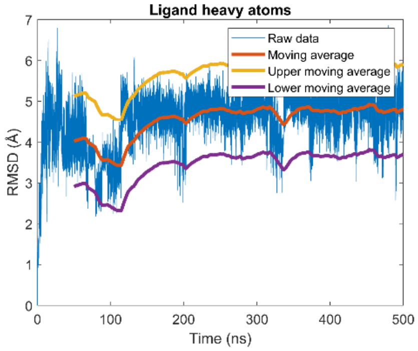

Ligand heavy atoms

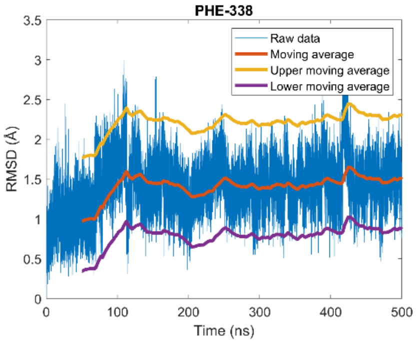

Phe-338

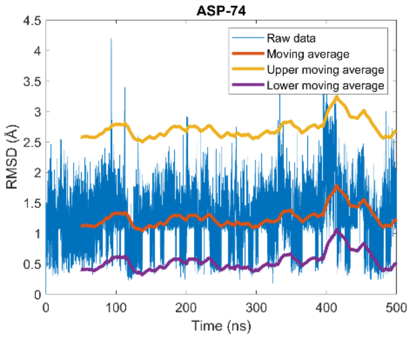

Asp-74

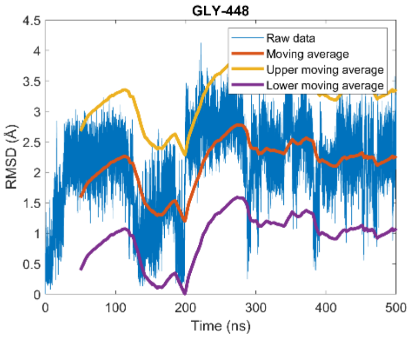

Gly-448

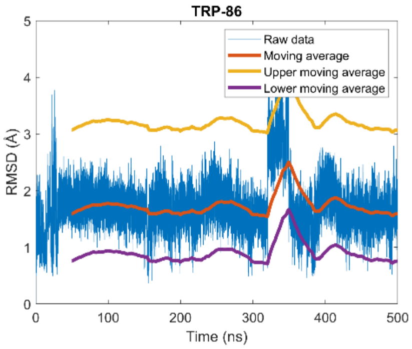

Trp-86

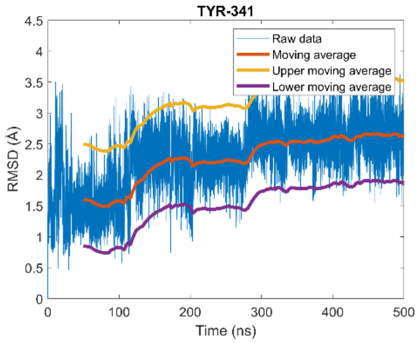

Tyr-341

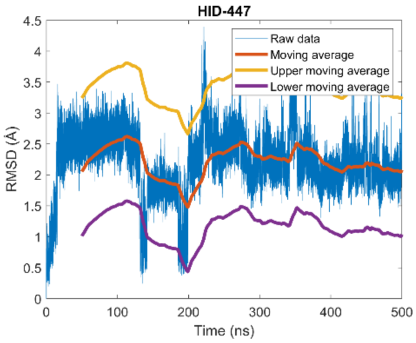

His-447

Figure S55. Heavy atoms RMSD for the 6 active site residues, backbone RMSD for the whole protein, backbone RMSD for the 17 active site residues and RMSD of ligand heavy atoms for C35.

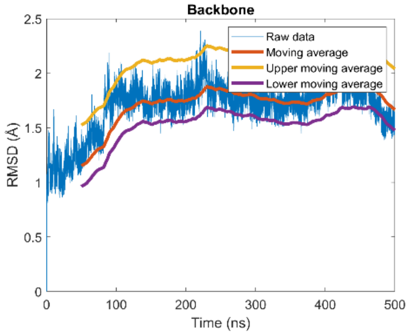

Protein Backbone

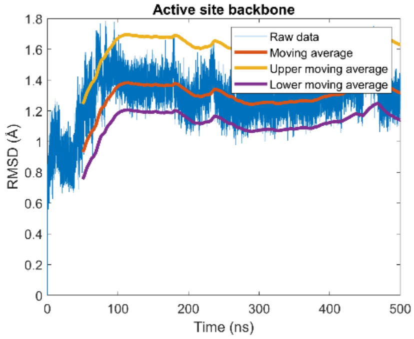

Active site backbone

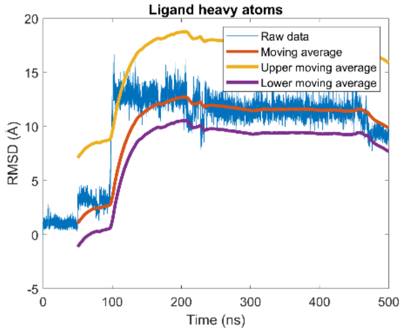

Ligand heavy atoms

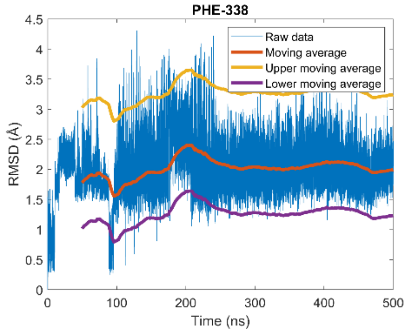

Phe-338

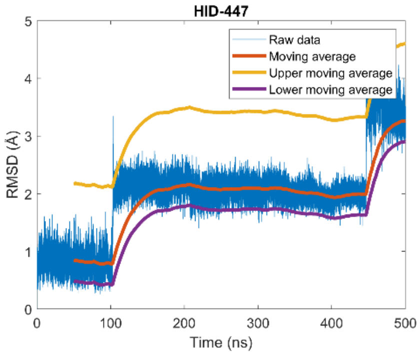

His-447

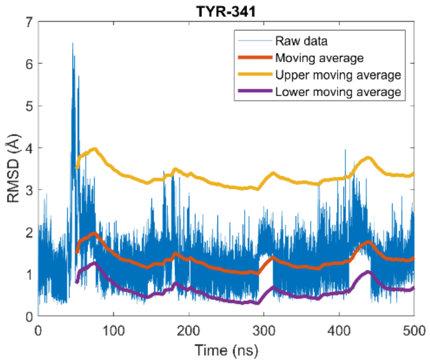

Tyr-341

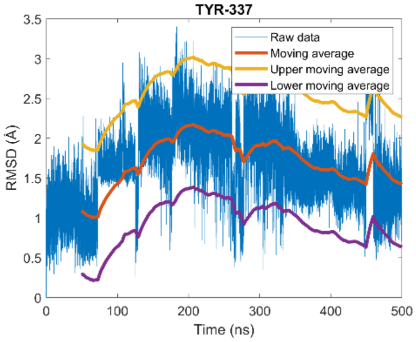

Tyr-337

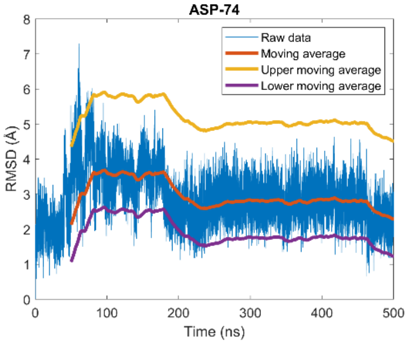

Asp-74

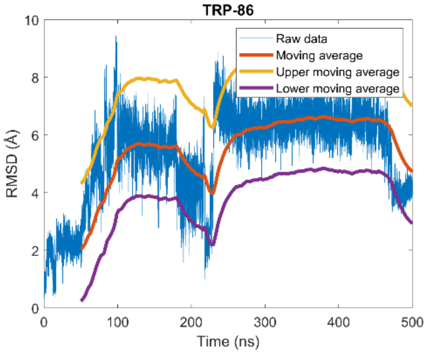

Trp-86

Figure S56. Heavy atoms RMSD for the 6 active site residues, backbone RMSD for the whole protein, backbone RMSD for the 17 active site residues and RMSD of ligand heavy atoms for C36.

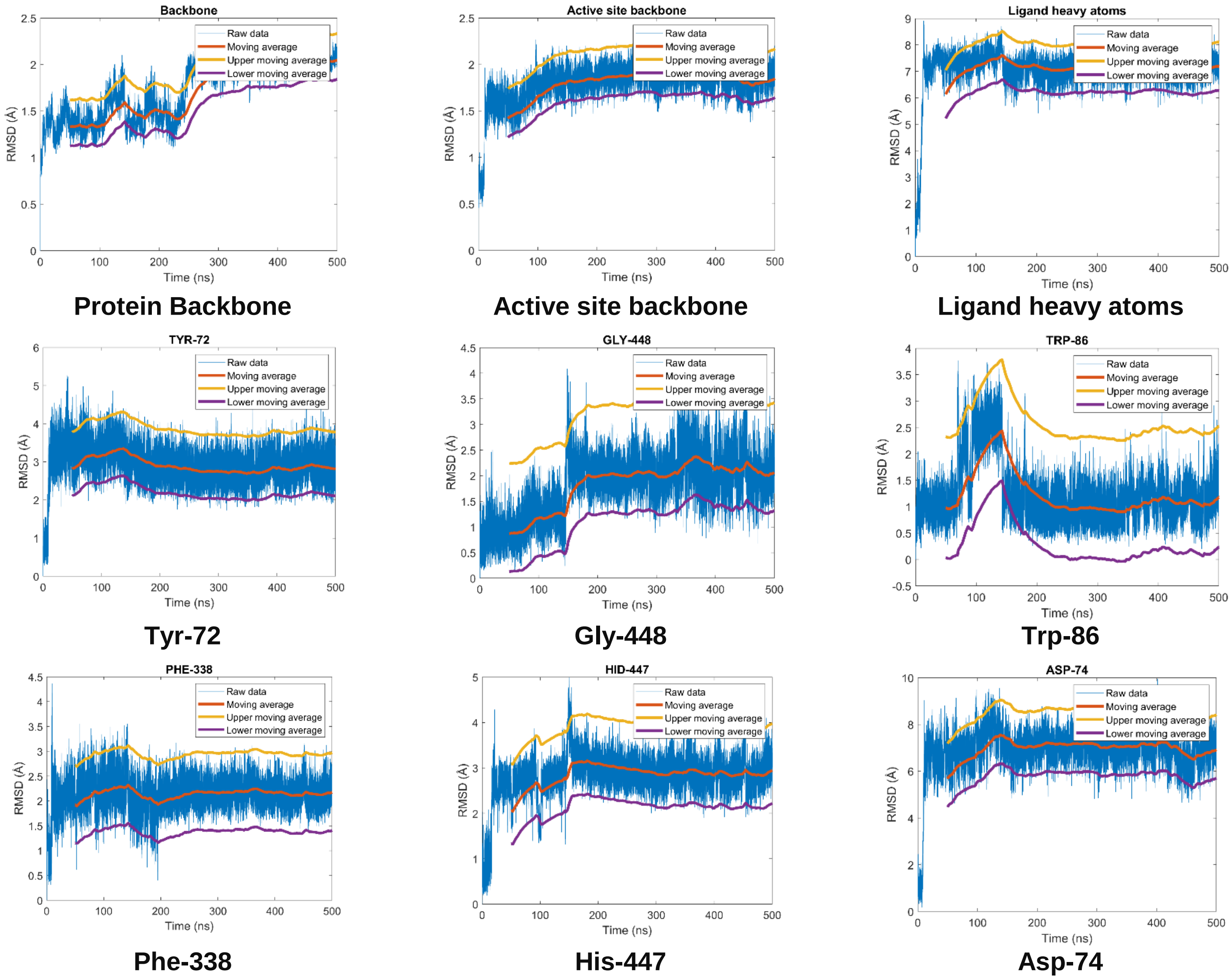

Figure S57. Heavy atoms RMSD for the 6 active site residues, backbone RMSD for the whole protein, backbone RMSD for the 17 active site residues and RMSD of ligand heavy atoms for C42.

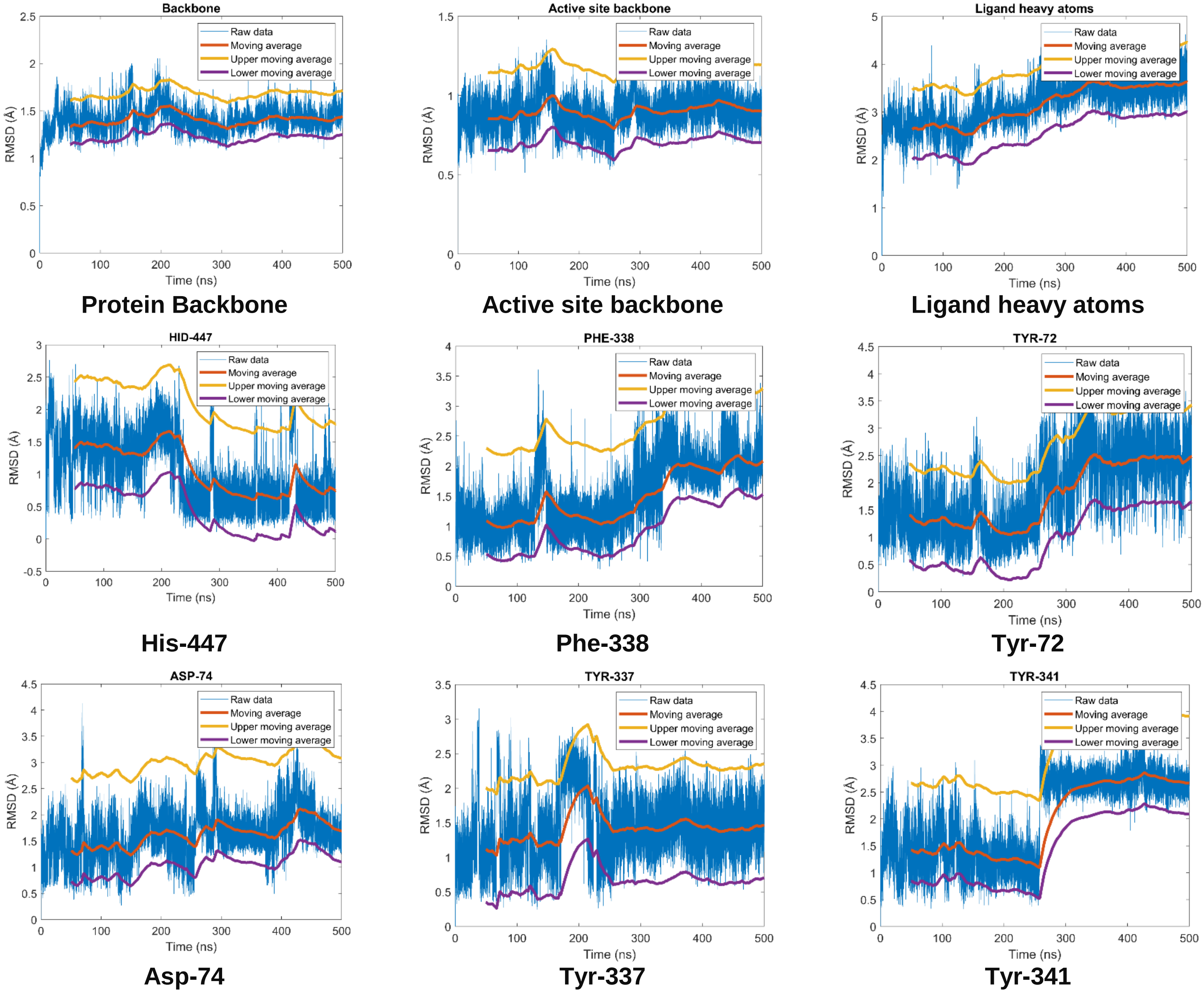

Figure S58. Heavy atoms RMSD for the 6 active site residues, backbone RMSD for the whole protein, backbone RMSD for the 17 active site residues and RMSD of ligand heavy atoms for C50.

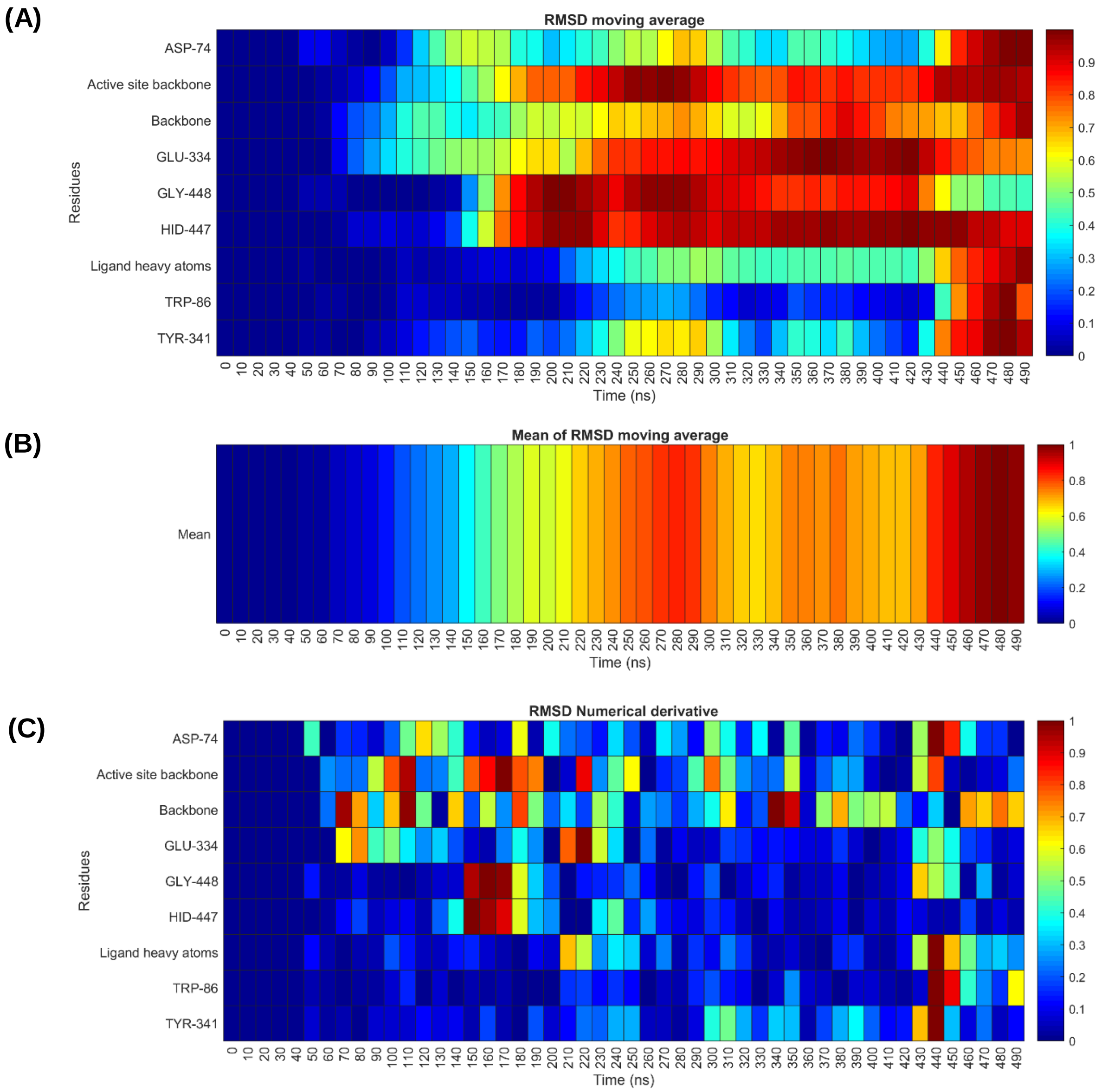

Figure S59. (A) RMSD Normalized moving average for the 9 data sets (Heavy atoms RMSD for the 6 active site residues, backbone RMSD for the whole protein, backbone RMSD for the 17 active site residues, and RMSD of ligand heavy atoms. (B) Mean of RMSD Normalized moving average for the 9 data sets. (C) Numerical derivative of RMSD Normalized moving average. The plots belong to C4.

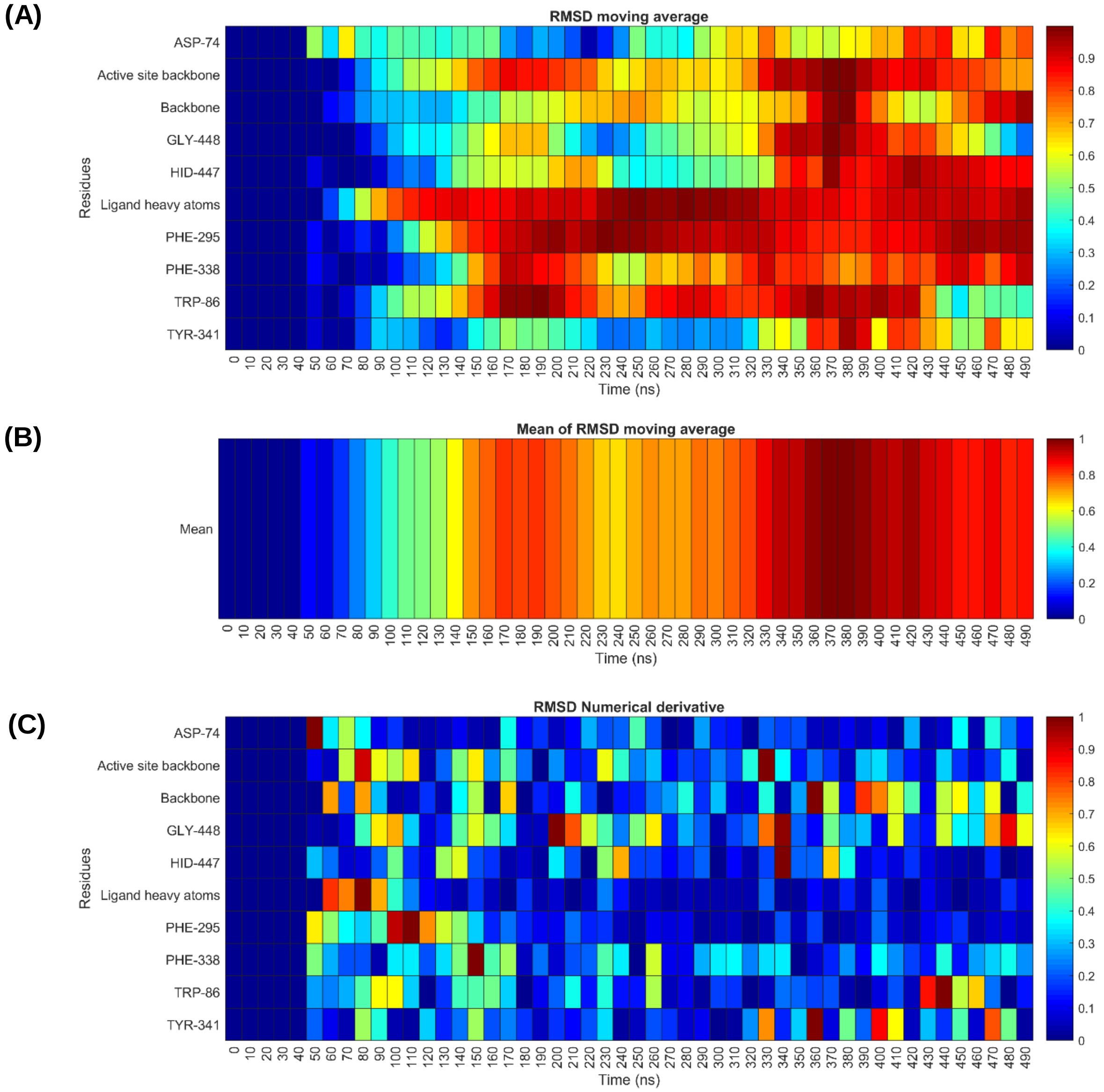

Figure S60. (A) RMSD Normalized moving average for the 9 data sets (Heavy atoms RMSD for the 6 active site residues, backbone RMSD for the whole protein, backbone RMSD for the 17 active site residues, and RMSD of ligand heavy atoms. (B) Mean of RMSD Normalized moving average for the 9 data sets. (C) Numerical derivative of RMSD Normalized moving average. The plots belong to C19.

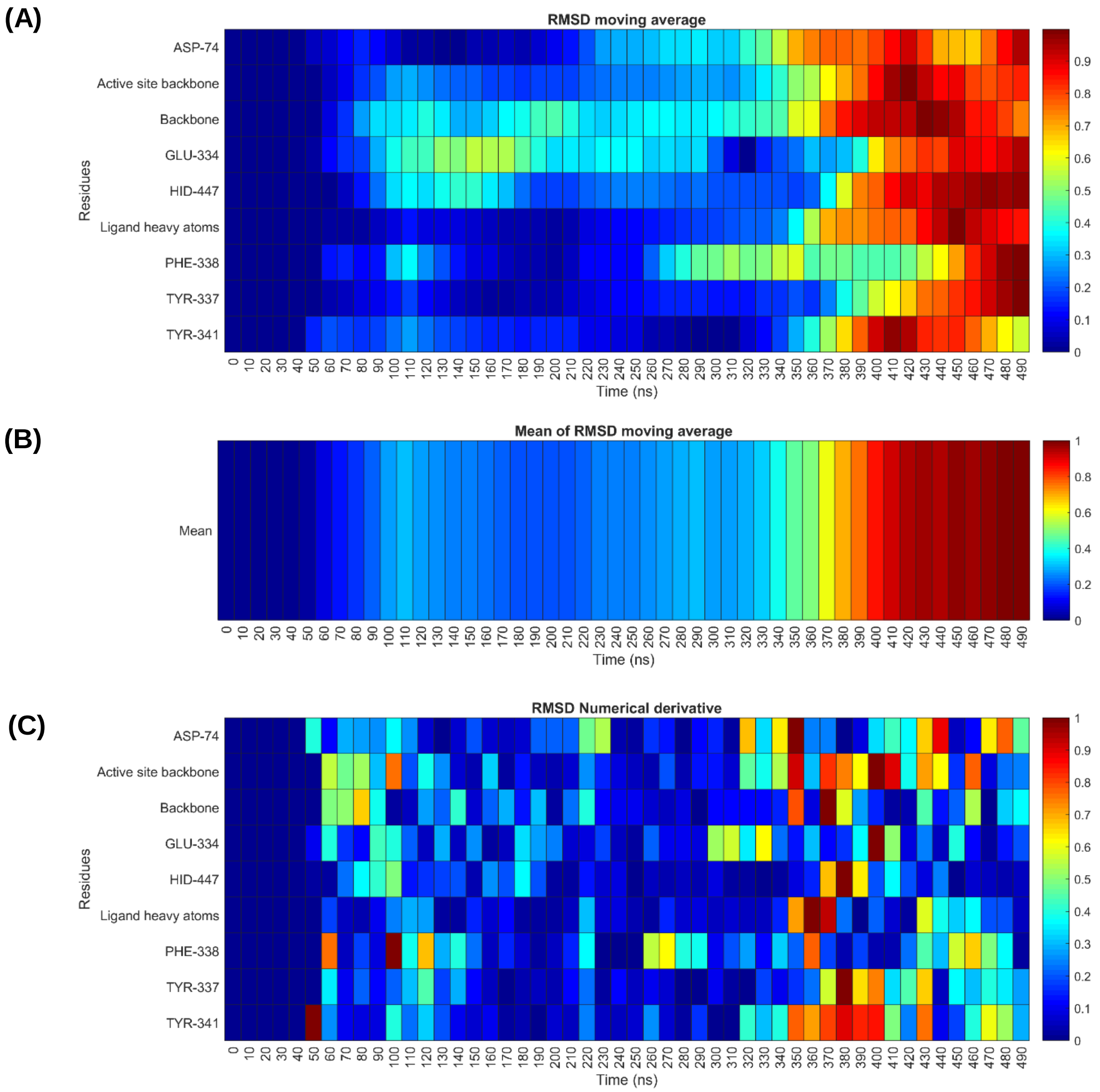

Figure S61. (A) RMSD Normalized moving average for the 9 data sets (Heavy atoms RMSD for the 6 active site residues, backbone RMSD for the whole protein, backbone RMSD for the 17 active site residues, and RMSD of ligand heavy atoms. (B) Mean of RMSD Normalized moving average for the 9 data sets. (C) Numerical derivative of RMSD Normalized moving average. The plots belong to C20.

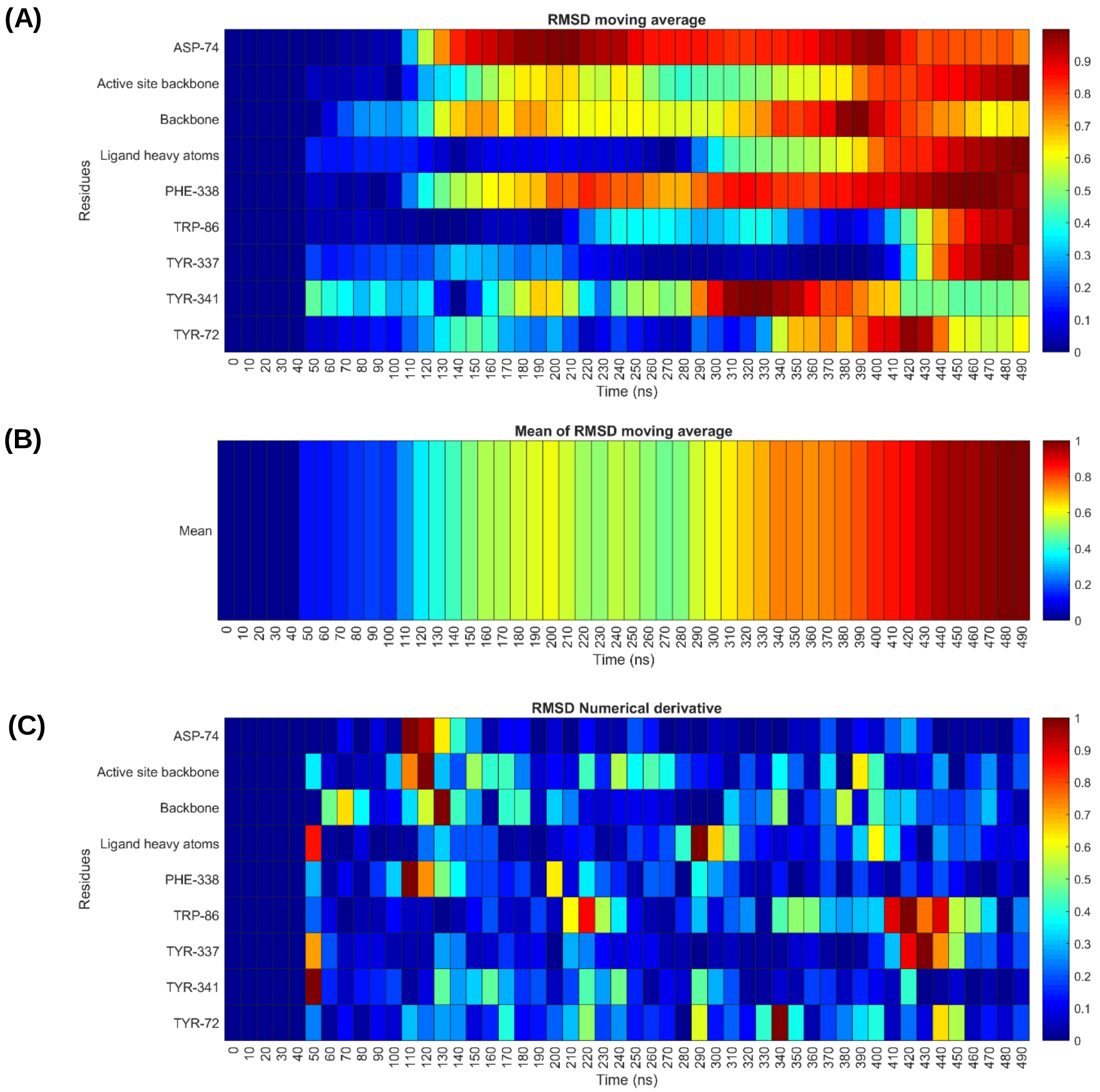

Figure S62. (A) RMSD Normalized moving average for the 9 data sets (Heavy atoms RMSD for the 6 active site residues, backbone RMSD for the whole protein, backbone RMSD for the 17 active site residues, and RMSD of ligand heavy atoms. (B) Mean of RMSD Normalized moving average for the 9 data sets. (C) Numerical derivative of RMSD Normalized moving average. The plots belong to C23.

(A)

(B)

(C)

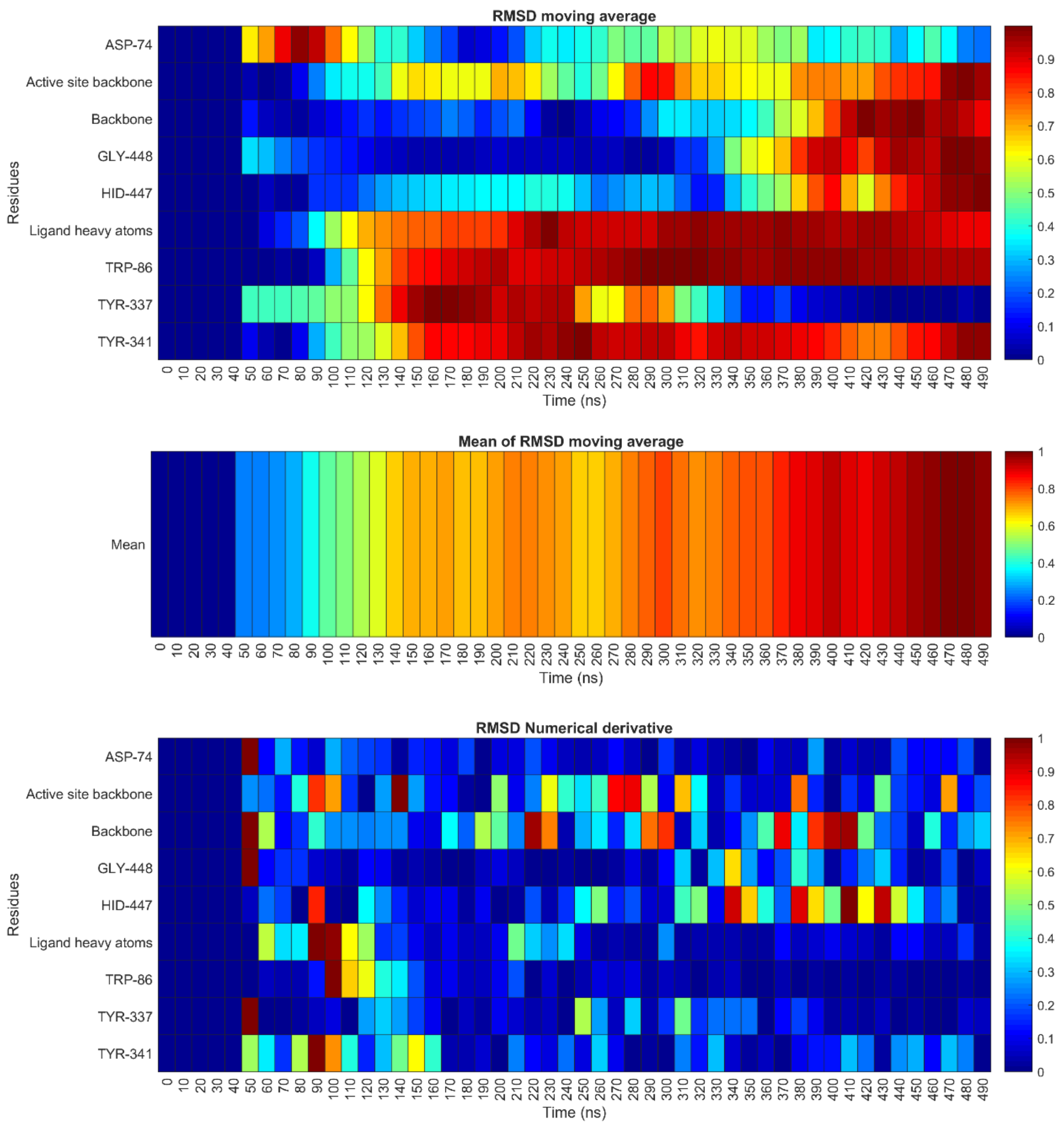

Figure S63. (A) RMSD Normalized moving average for the 9 data sets (Heavy atoms RMSD for the 6 active site residues, backbone RMSD for the whole protein, backbone RMSD for the 17 active site residues, and RMSD of ligand heavy atoms. (B) Mean of RMSD Normalized moving average for the 9 data sets. (C) Numerical derivative of RMSD Normalized moving average. The plots belong to C31.

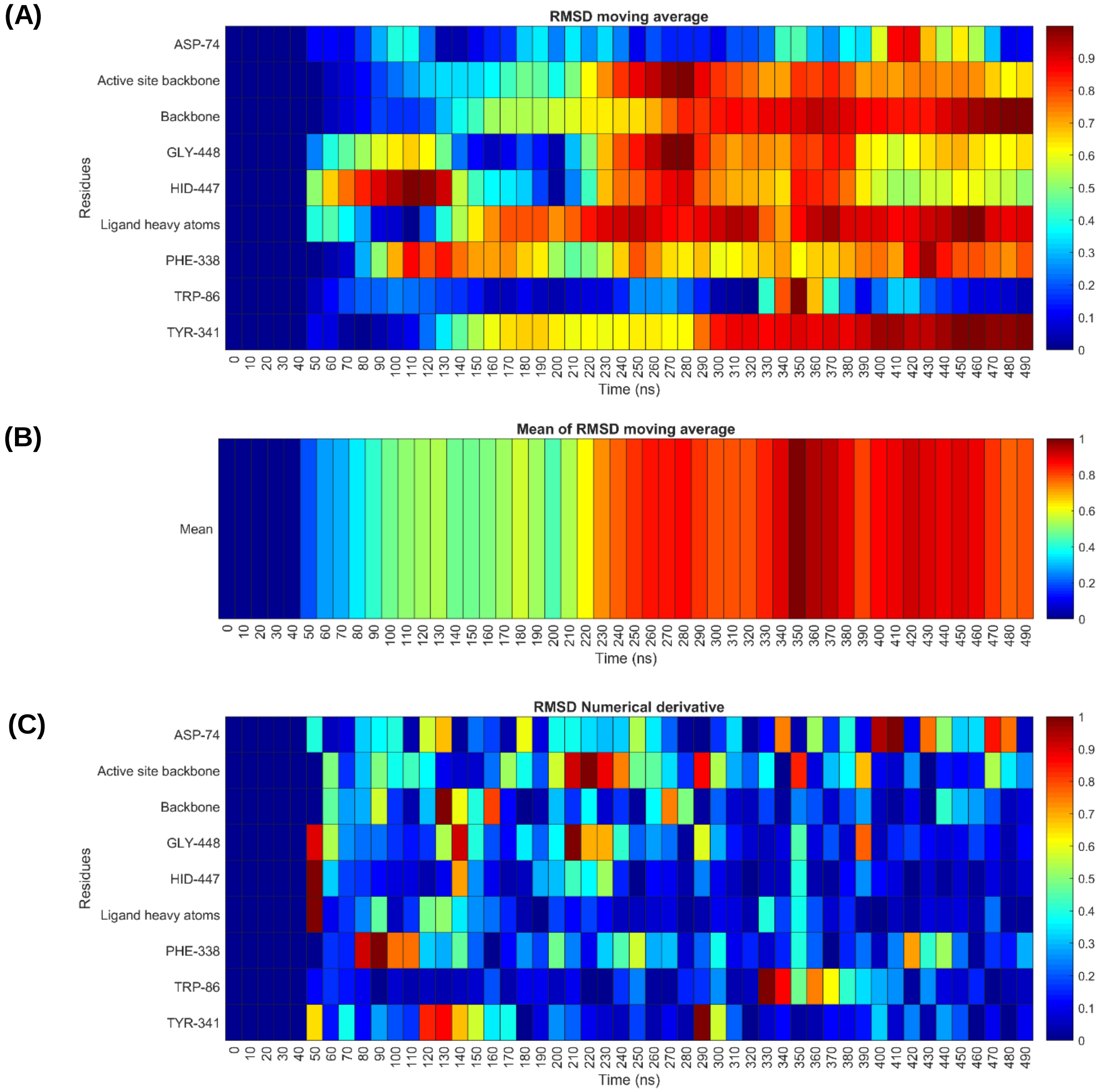

Figure S64. (A) RMSD Normalized moving average for the 9 data sets (Heavy atoms RMSD for the 6 active site residues, backbone RMSD for the whole protein, backbone RMSD for the 17 active site residues, and RMSD of ligand heavy atoms. (B) Mean of RMSD Normalized moving average for the 9 data sets. (C) Numerical derivative of RMSD Normalized moving average. The plots belong to C35.

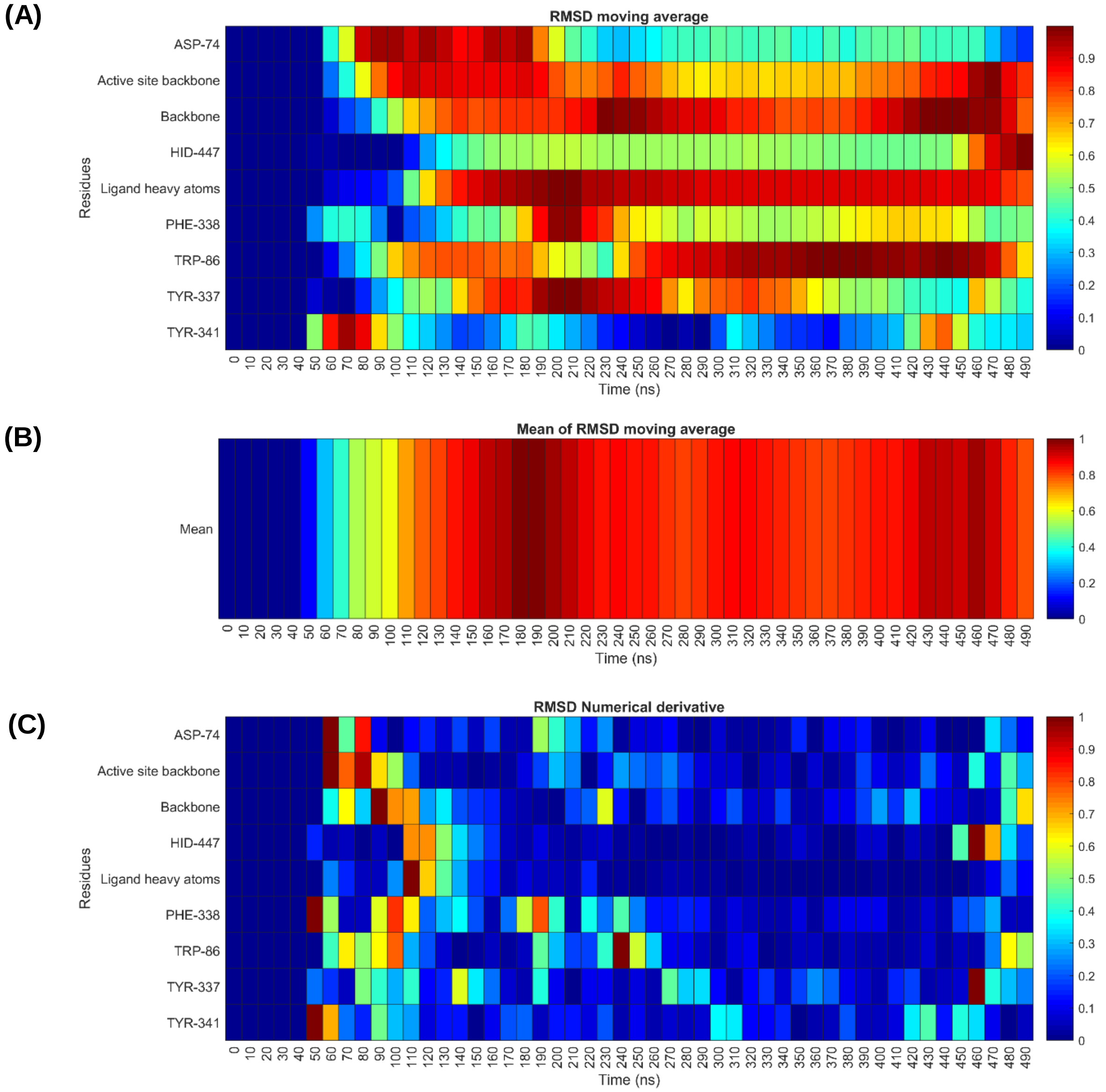

Figure S65. (A) RMSD Normalized moving average for the 9 data sets (Heavy atoms RMSD for the 6 active site residues, backbone RMSD for the whole protein, backbone RMSD for the 17 active site residues, and RMSD of ligand heavy atoms. (B) Mean of RMSD Normalized moving average for the 9 data sets. (C) Numerical derivative of RMSD Normalized moving average. The plots belong to C36.

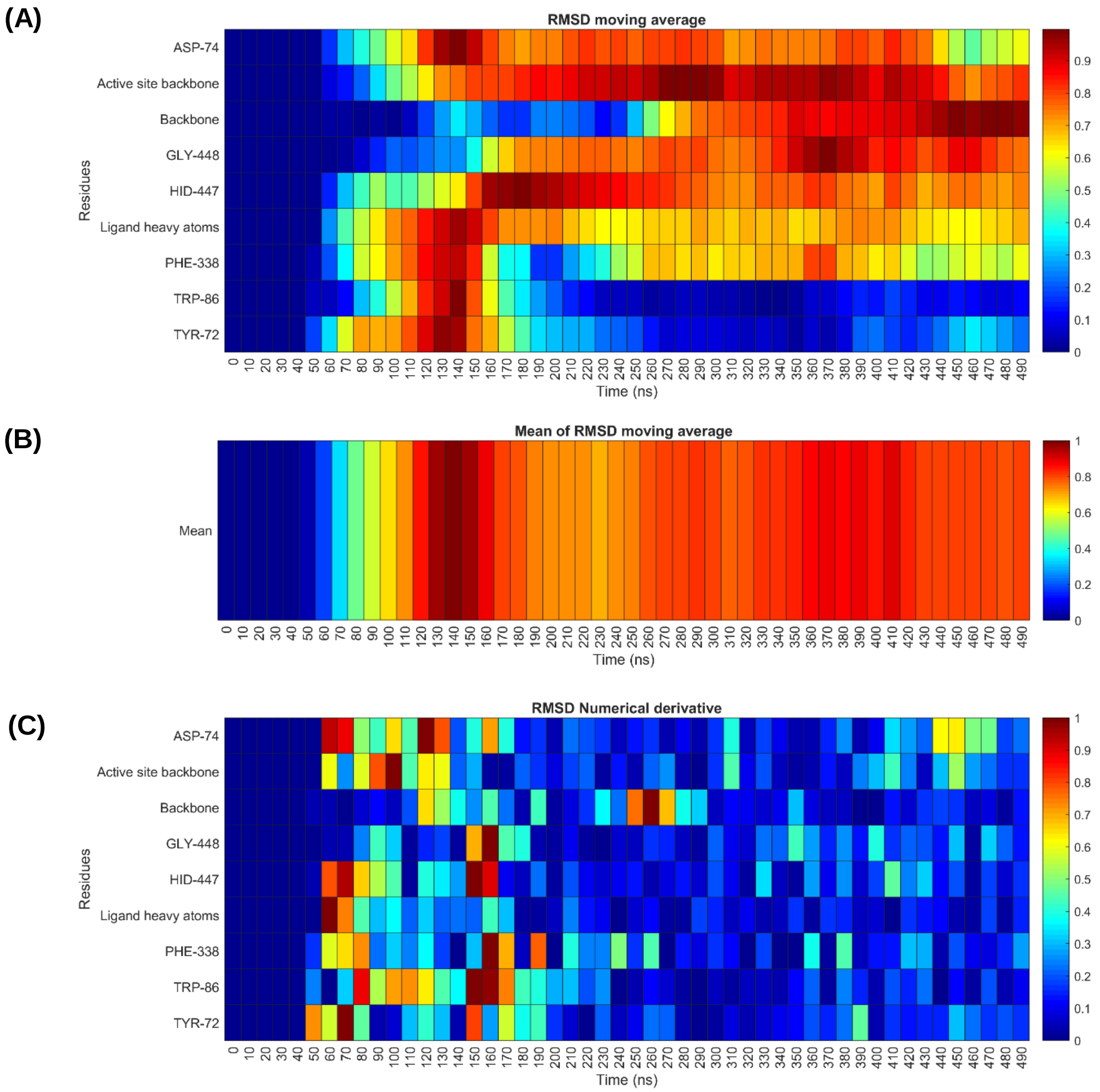

Figure S66. (A) RMSD Normalized moving average for the 9 data sets (Heavy atoms RMSD for the 6 active site residues, backbone RMSD for the whole protein, backbone RMSD for the 17 active site residues, and RMSD of ligand heavy atoms. (B) Mean of RMSD Normalized moving average for the 9 data sets. (C) Numerical derivative of RMSD Normalized moving average. The plots belong to C42.

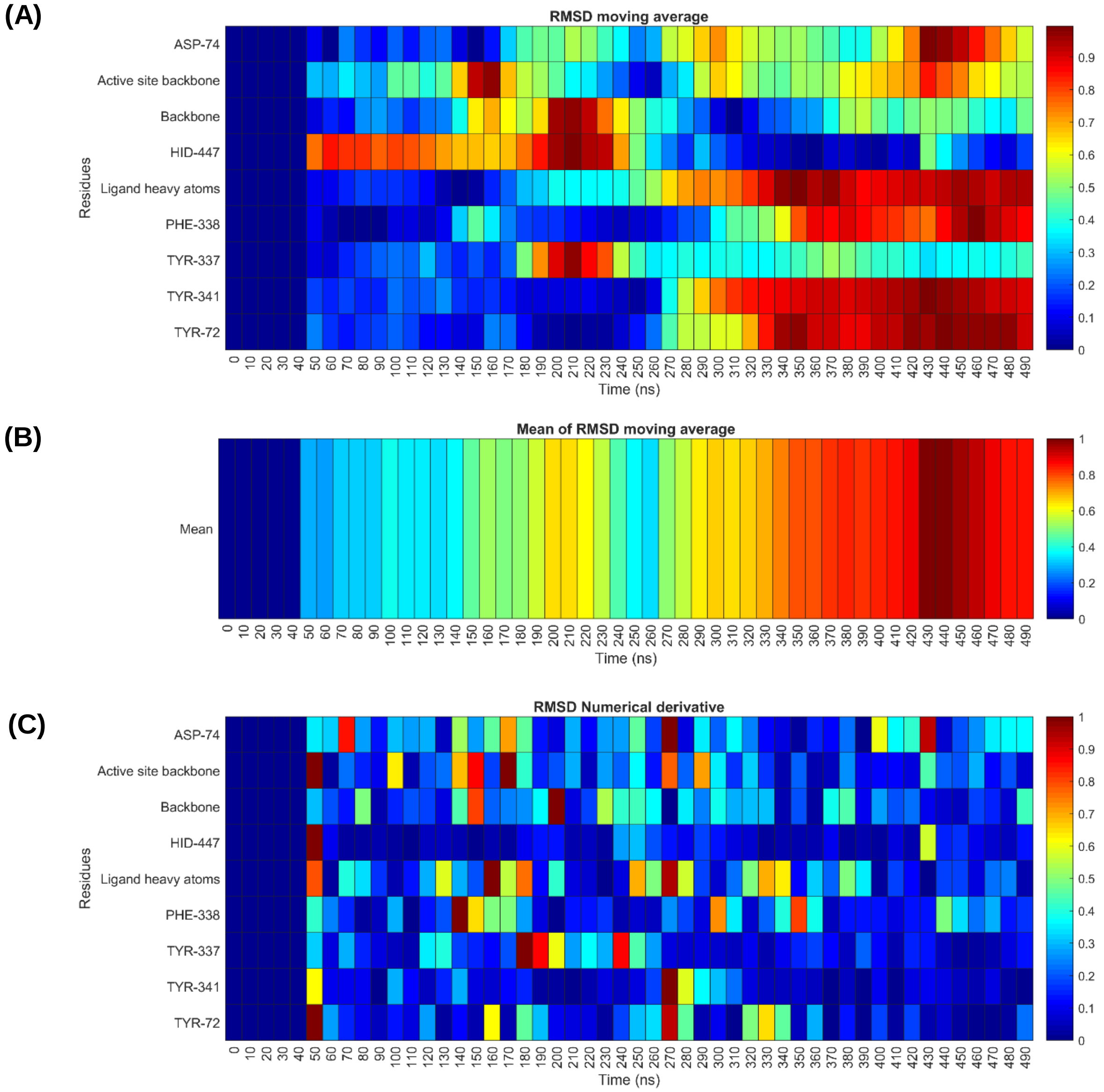

Figure S67. (A) RMSD Normalized moving average for the 9 data sets (Heavy atoms RMSD for the 6 active site residues, backbone RMSD for the whole protein, backbone RMSD for the 17 active site residues, and RMSD of ligand heavy atoms. (B) Mean of RMSD Normalized moving average for the 9 data sets. (C) Numerical derivative of RMSD Normalized moving average. The plots belong to C50.

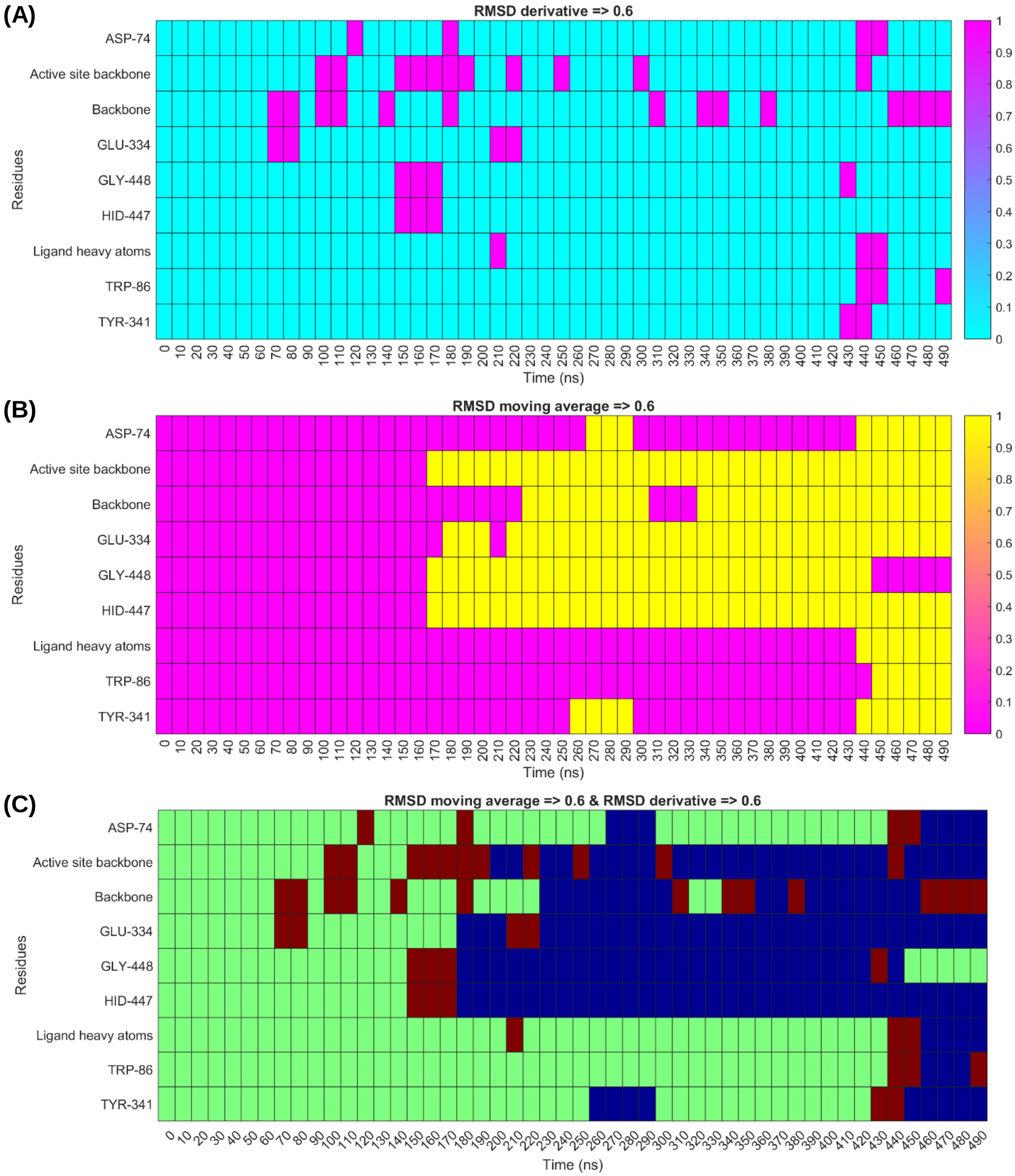

Figure S68. (A) Simulation times where the value of the numerical derivative is higher than 0.6. Pink (RMSD derivative ≥ 0.6), cyan (RMSD derivative < 0.6). **(B) Simulation times where the RMSD Normalized moving average is higher than 0.6.** Yellow (RMSD moving average ≥ 0.6), pink (RMSD moving average < 0.6)**. (C) Simulation times where the RMSD Normalized moving average and the numerical derivative are higher than 0.6.** Green (RMSD moving average < 0.6), blue (RMSD moving average => 0.6), and red (RMSD derivative => 0.6). The plots belong to C4.

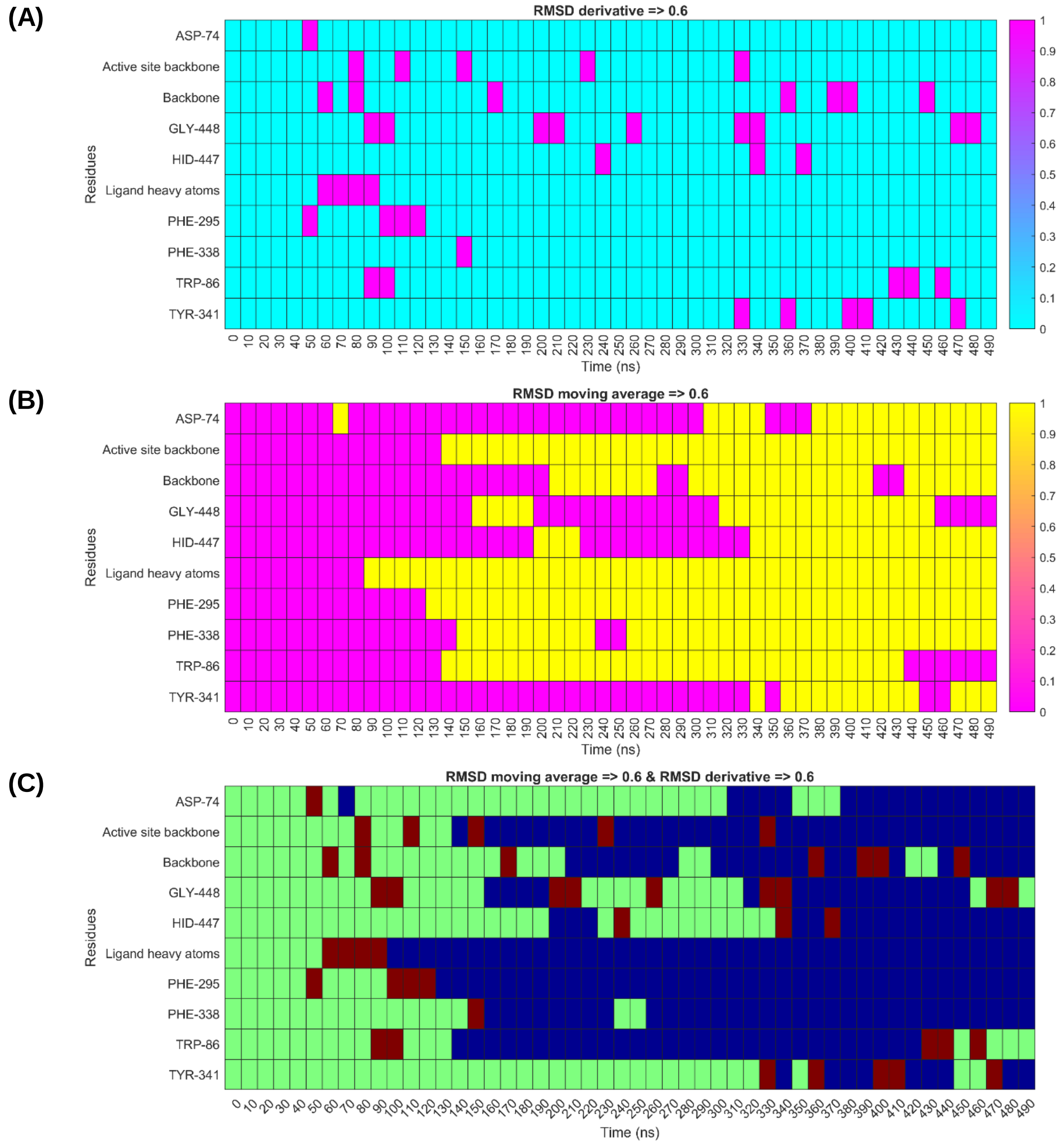

Figure S69. (A) Simulation times where the value of the numerical derivative is higher than 0.6. Pink (RMSD derivative ≥ 0.6), cyan (RMSD derivative < 0.6). **(B) Simulation times where the RMSD Normalized moving average is higher than 0.6.** Yellow (RMSD moving average ≥ 0.6), pink (RMSD moving average < 0.6)**. (C) Simulation times where the RMSD Normalized moving average and the numerical derivative are higher than 0.6.** Green (RMSD moving average < 0.6), blue (RMSD moving average => 0.6), and red (RMSD derivative => 0.6). The plots belong to C19.

(A)

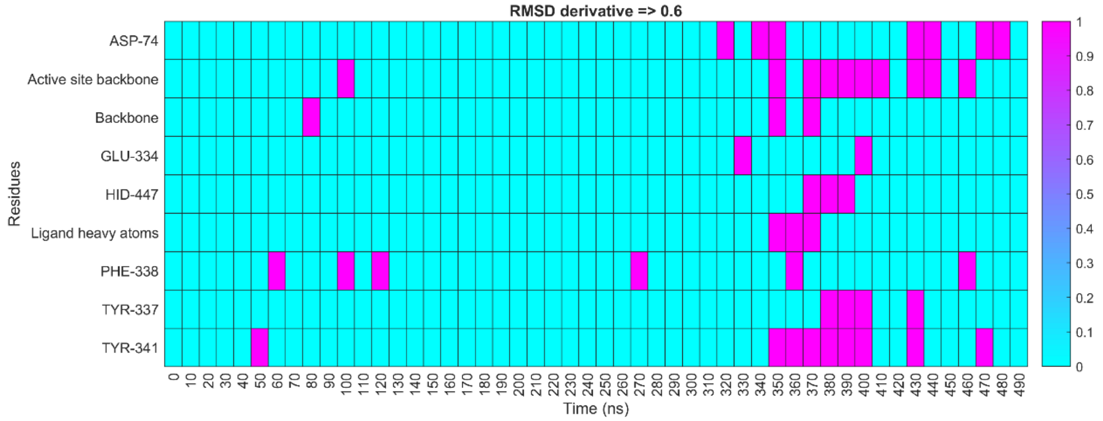

(B)

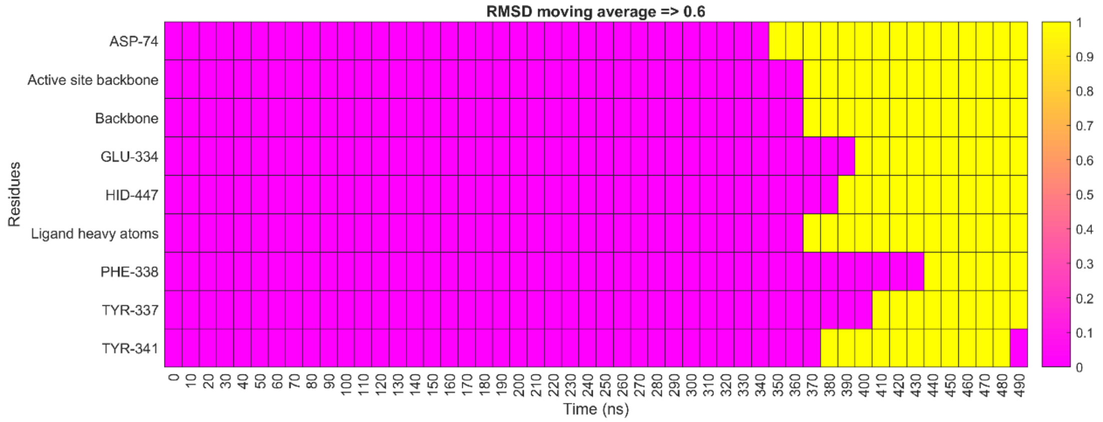

(C)

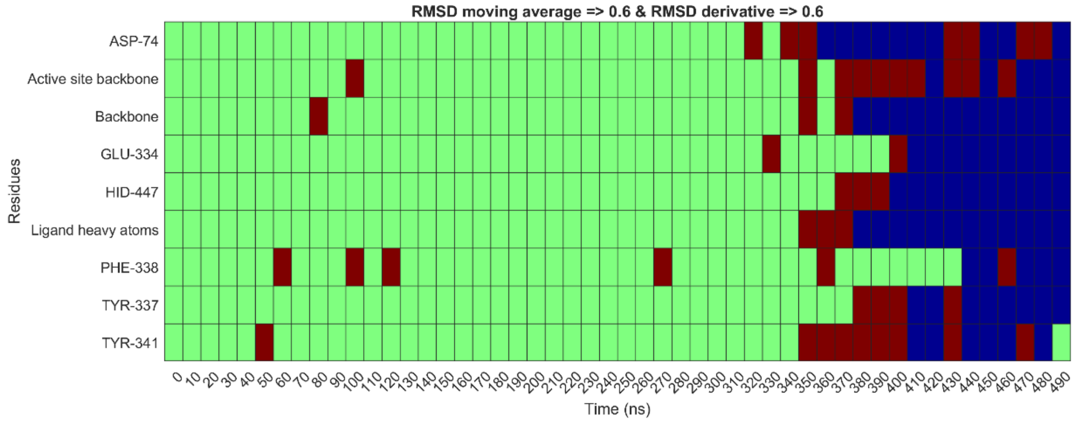

Figure S70. (A) Simulation times where the value of the numerical derivative is higher than 0.6. Pink (RMSD derivative ≥ 0.6), cyan (RMSD derivative < 0.6). **(B) Simulation times where the RMSD Normalized moving average is higher than 0.6.** Yellow (RMSD moving average ≥ 0.6), pink (RMSD moving average < 0.6)**. (C) Simulation times where the RMSD Normalized moving average and the numerical derivative are higher than 0.6.** Green (RMSD moving average < 0.6), blue (RMSD moving average => 0.6), and red (RMSD derivative => 0.6). The plots belong to C20.

(A) (B) (C)

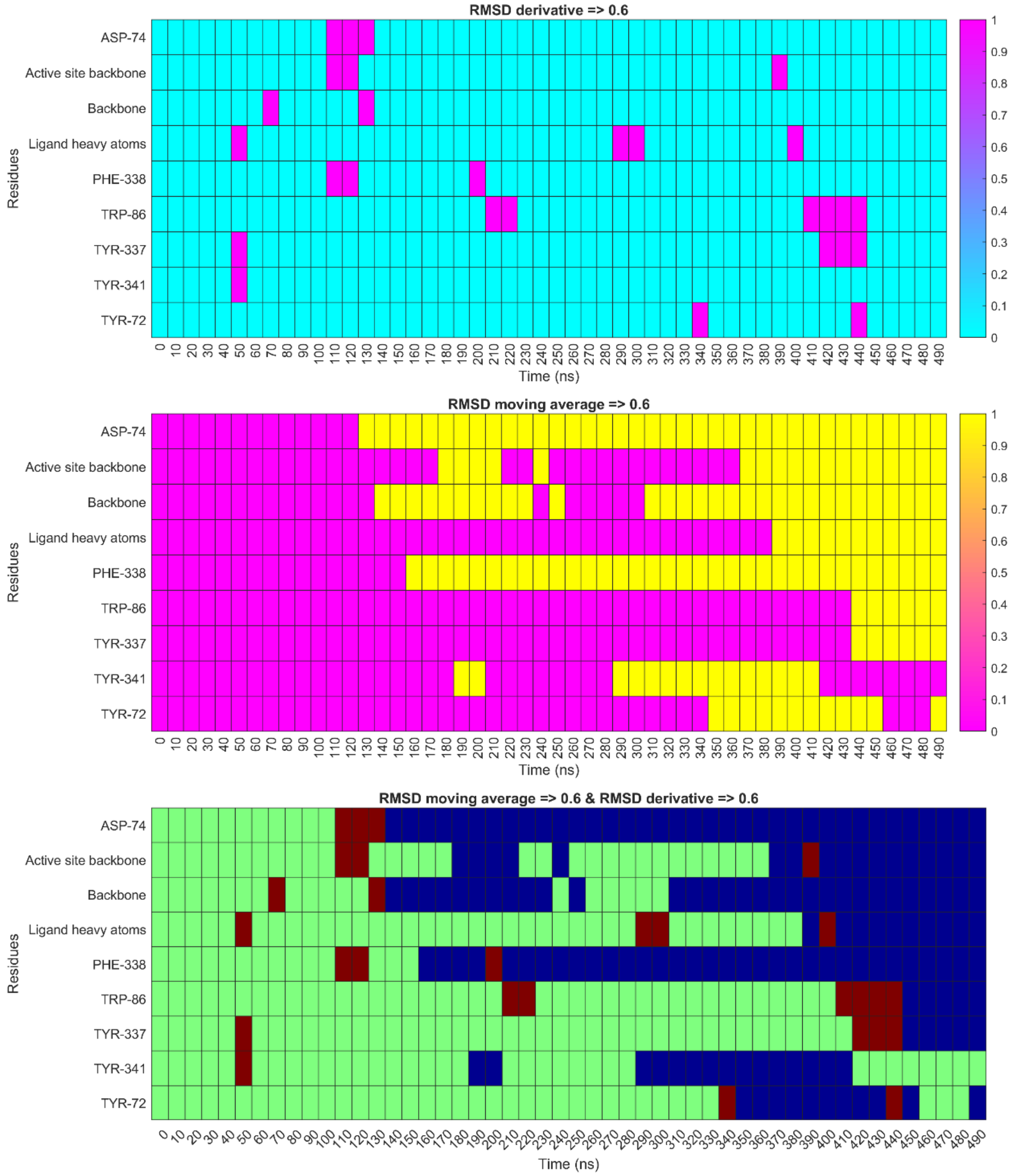

Figure S71. (A) Simulation times where the value of the numerical derivative is higher than 0.6. Pink (RMSD derivative ≥ 0.6), cyan (RMSD derivative < 0.6). **(B) Simulation times where the RMSD Normalized moving average is higher than 0.6.** Yellow (RMSD moving average ≥ 0.6), pink (RMSD moving average < 0.6)**. (C) Simulation times where the RMSD Normalized moving average and the numerical derivative are higher than 0.6.** Green (RMSD moving average < 0.6), blue (RMSD moving average => 0.6), and red (RMSD derivative => 0.6). The plots belong to C23.

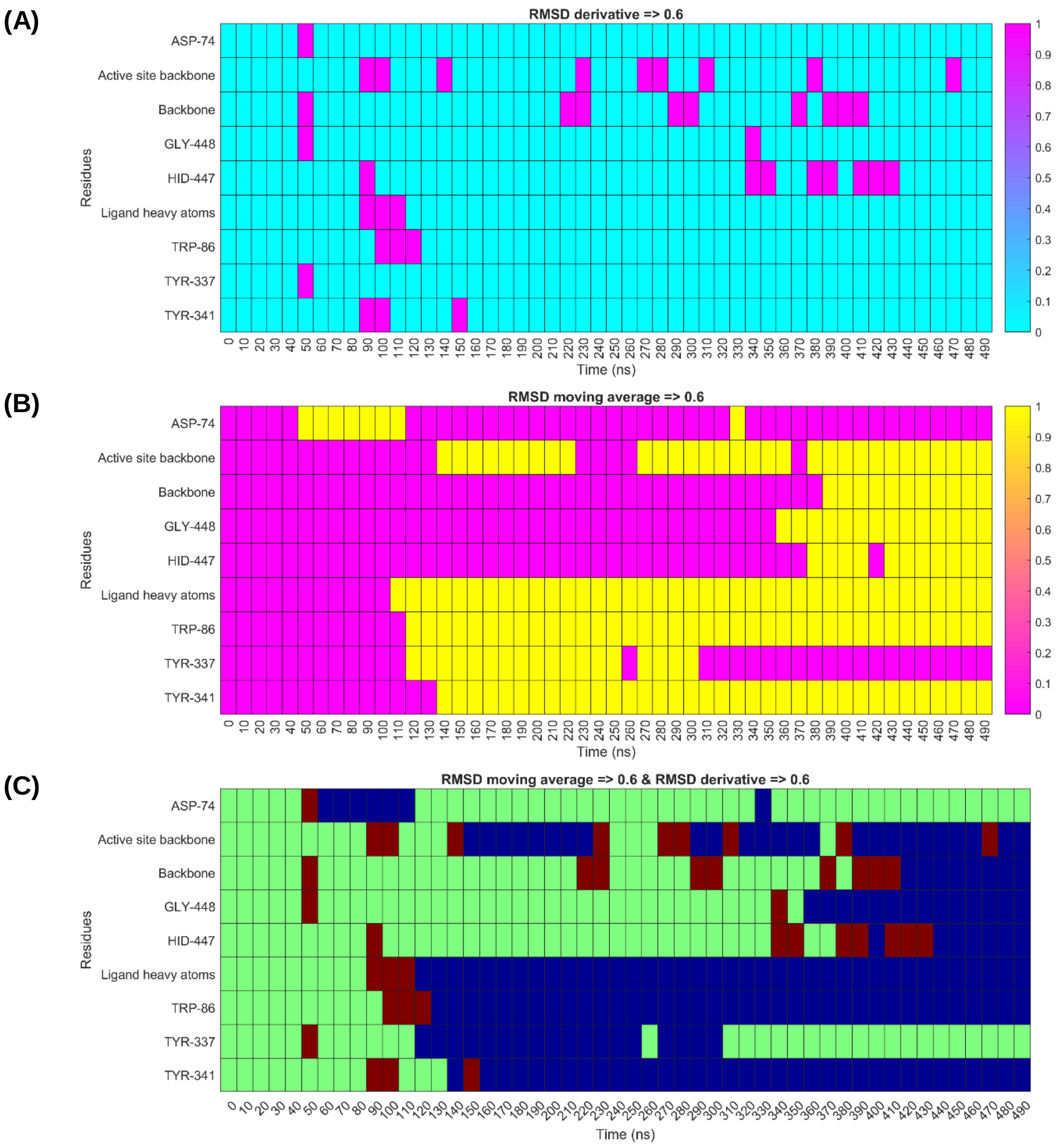

Figure S72. (A) Simulation times where the value of the numerical derivative is higher than 0.6. Pink (RMSD derivative ≥ 0.6), cyan (RMSD derivative < 0.6). **(B) Simulation times where the RMSD Normalized moving average is higher than 0.6.** Yellow (RMSD moving average ≥ 0.6), pink (RMSD moving average < 0.6)**. (C) Simulation times where the RMSD Normalized moving average and the numerical derivative are higher than 0.6.** Green (RMSD moving average < 0.6), blue (RMSD moving average => 0.6), and red (RMSD derivative => 0.6). The plots belong to C31.

(A)

(B)

(C)

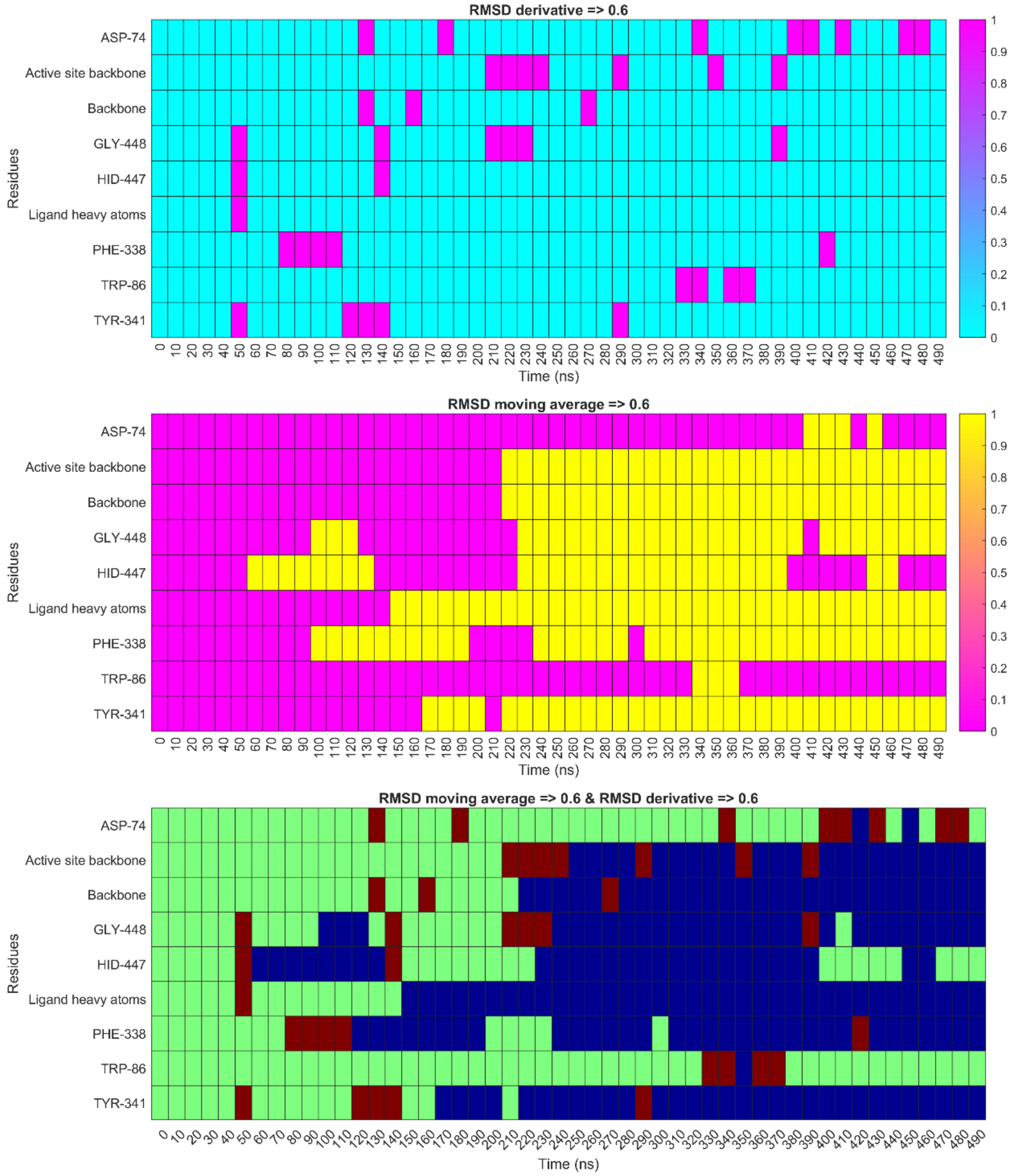

Figure S73. (A) Simulation times where the value of the numerical derivative is higher than 0.6. Pink (RMSD derivative ≥ 0.6), cyan (RMSD derivative < 0.6). **(B) Simulation times where the RMSD Normalized moving average is higher than 0.6.** Yellow (RMSD moving average ≥ 0.6), pink (RMSD moving average < 0.6)**. (C) Simulation times where the RMSD Normalized moving average and the numerical derivative are higher than 0.6.** Green (RMSD moving average < 0.6), blue (RMSD moving average => 0.6), and red (RMSD derivative => 0.6). The plots belong to C35

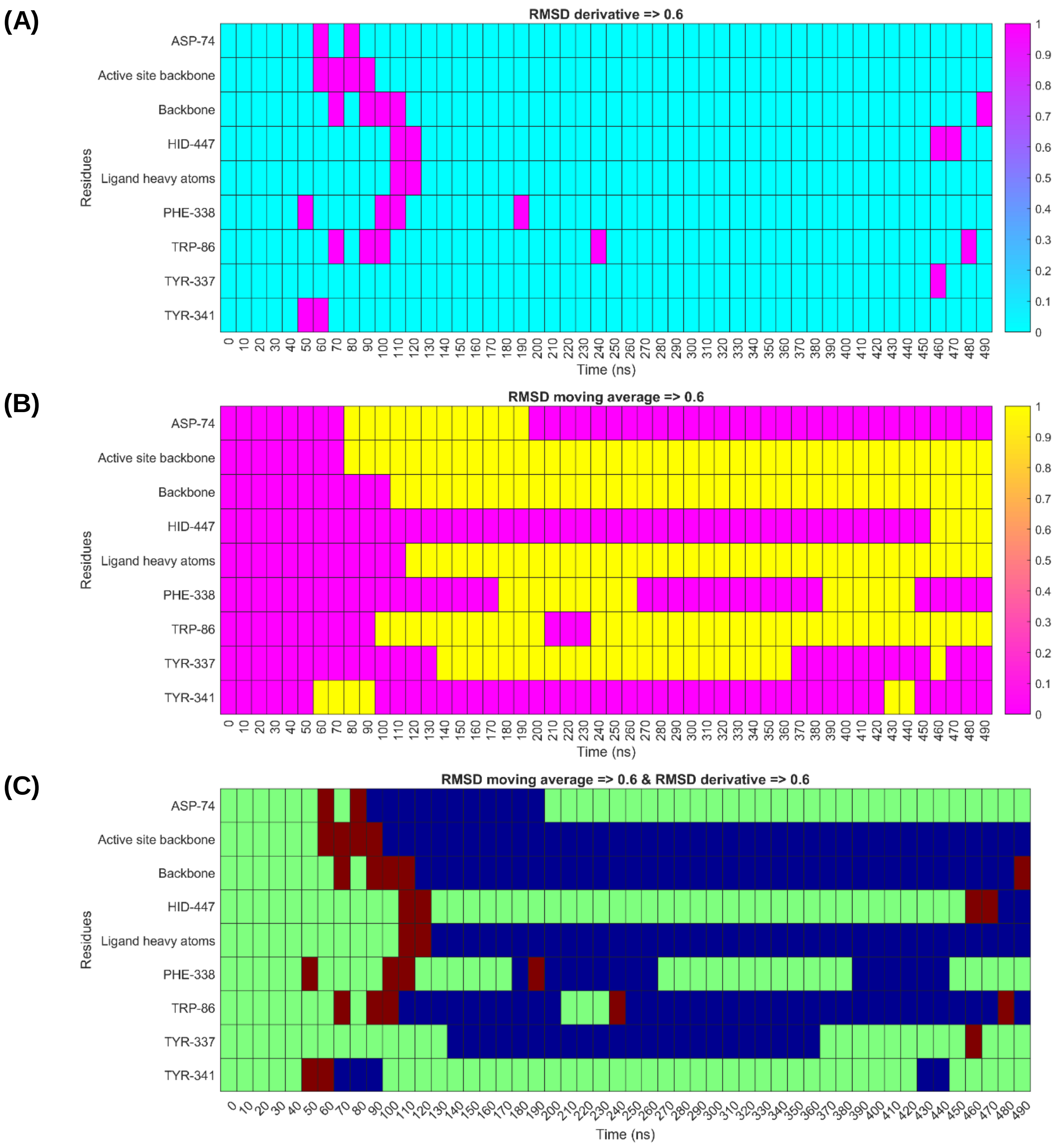

Figure S74. (A) Simulation times where the value of the numerical derivative is higher than 0.6. Pink (RMSD derivative ≥ 0.6), cyan (RMSD derivative < 0.6). **(B) Simulation times where the RMSD Normalized moving average is higher than 0.6.** Yellow (RMSD moving average ≥ 0.6), pink (RMSD moving average < 0.6)**. (C) Simulation times where the RMSD Normalized moving average and the numerical derivative are higher than 0.6.** Green (RMSD moving average < 0.6), blue (RMSD moving average => 0.6), and red (RMSD derivative => 0.6). The plots belong to C36.

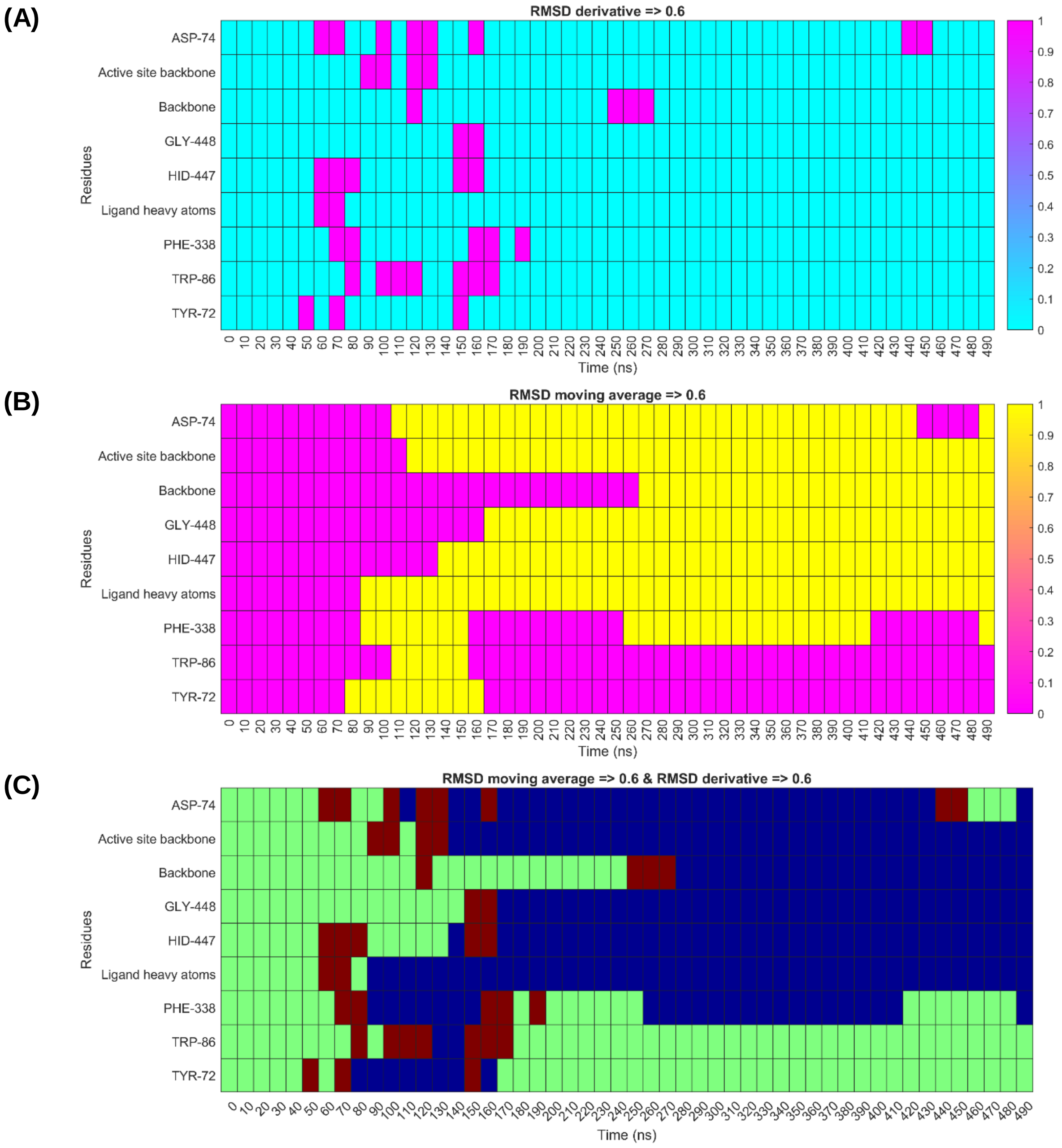

Figure S75. (A) Simulation times where the value of the numerical derivative is higher than 0.6. Pink (RMSD derivative ≥ 0.6), cyan (RMSD derivative < 0.6). **(B) Simulation times where the RMSD Normalized moving average is higher than 0.6.** Yellow (RMSD moving average ≥ 0.6), pink (RMSD moving average < 0.6)**. (C) Simulation times where the RMSD Normalized moving average and the numerical derivative are higher than 0.6.** Green (RMSD moving average < 0.6), blue (RMSD moving average => 0.6), and red (RMSD derivative => 0.6). The plots belong to C42.

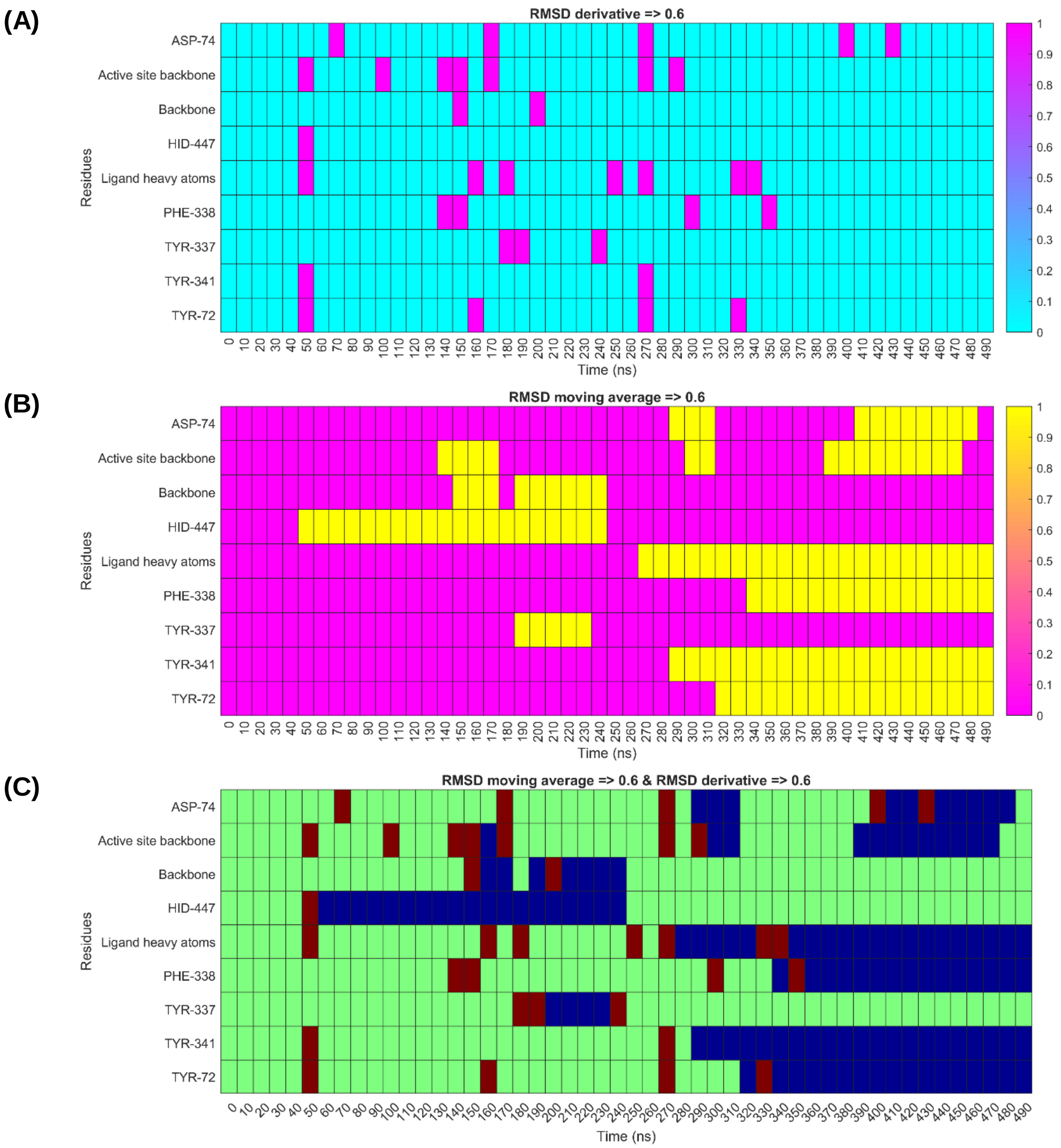

Figure S76. (A) Simulation times where the value of the numerical derivative is higher than 0.6. Pink (RMSD derivative ≥ 0.6), cyan (RMSD derivative < 0.6). **(B) Simulation times where the RMSD Normalized moving average is higher than 0.6.** Yellow (RMSD moving average ≥ 0.6), pink (RMSD moving average < 0.6)**. (C) Simulation times where the RMSD Normalized moving average and the numerical derivative are higher than 0.6.** Green (RMSD moving average < 0.6), blue (RMSD moving average => 0.6), and red (RMSD derivative => 0.6). The plots belong to C50.

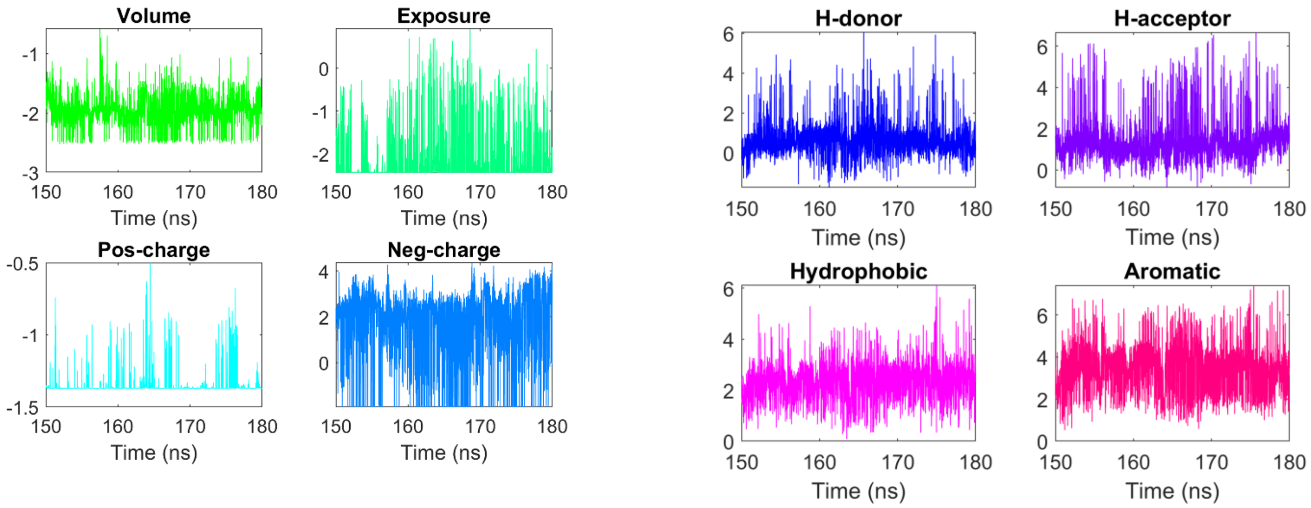

Figure S77. TRAPP-pocket results for the unstable zone of 150-180 ns of C4.

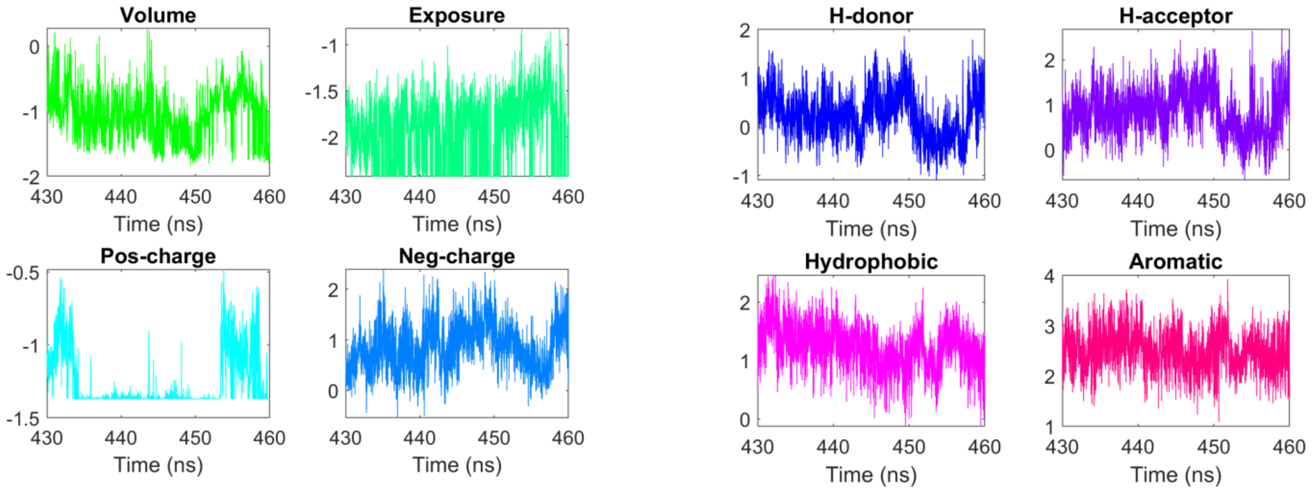

Figure S78. TRAPP-pocket results for the unstable zone of 430-460 ns for C4.

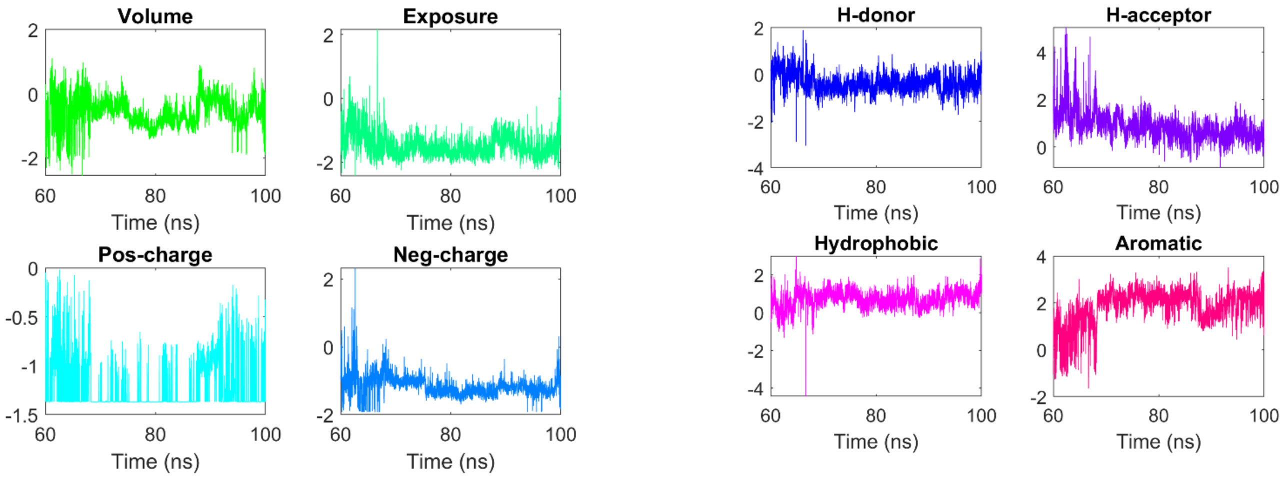

Figure S79. TRAPP-pocket results for the unstable zone of 60-100 ns for C19.

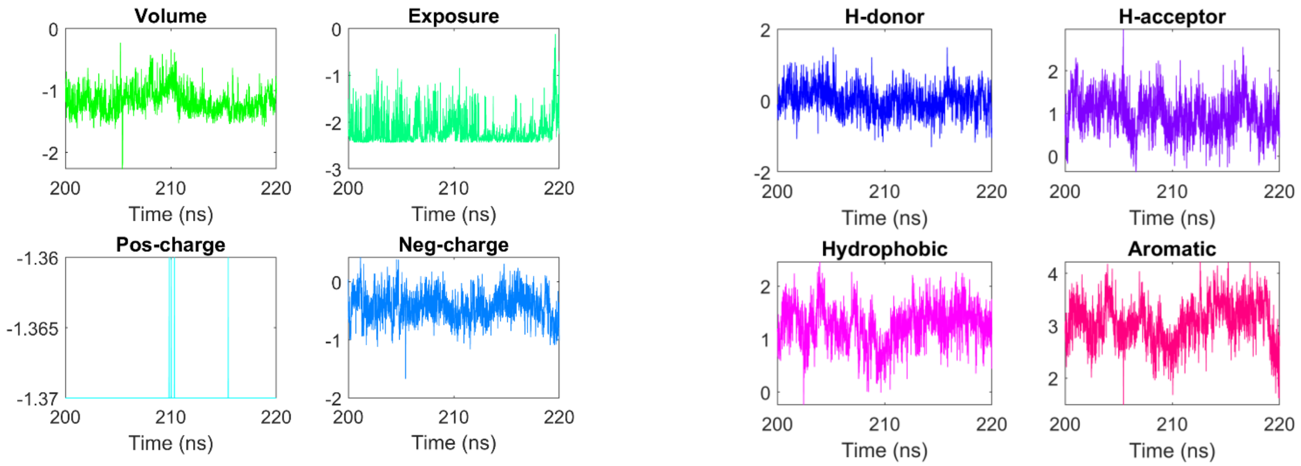

Figure S80. TRAPP-pocket results for the unstable zone of 200-220 ns for C19.

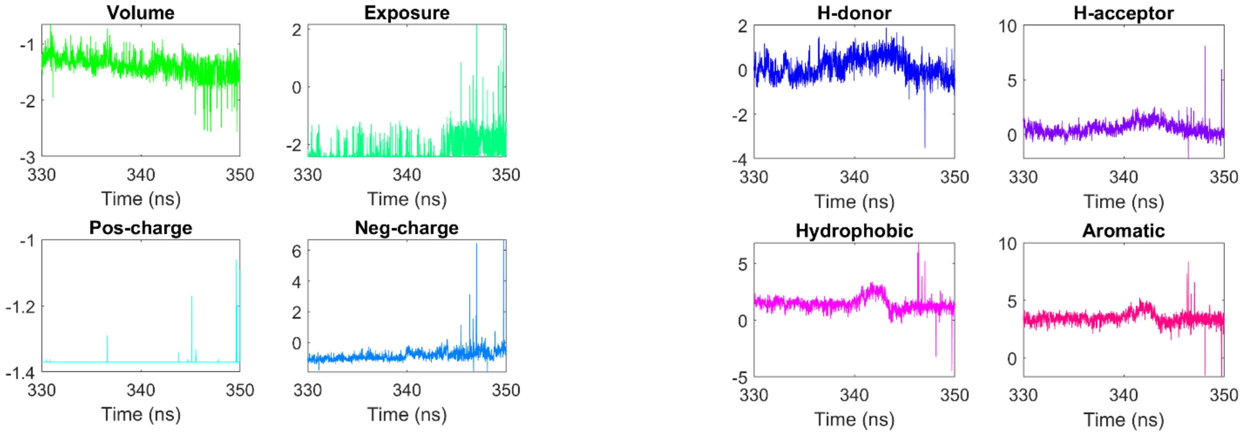

Figure S81. TRAPP-pocket results for the unstable zone of 330-350 ns for C19.

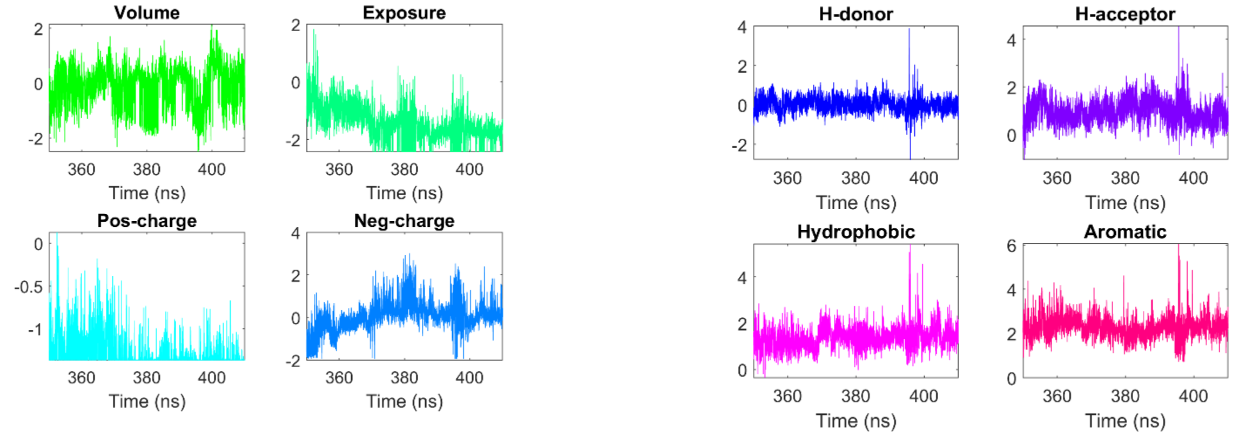

Figure S82. TRAPP-pocket results for the unstable zone of 350-410 ns for C20.

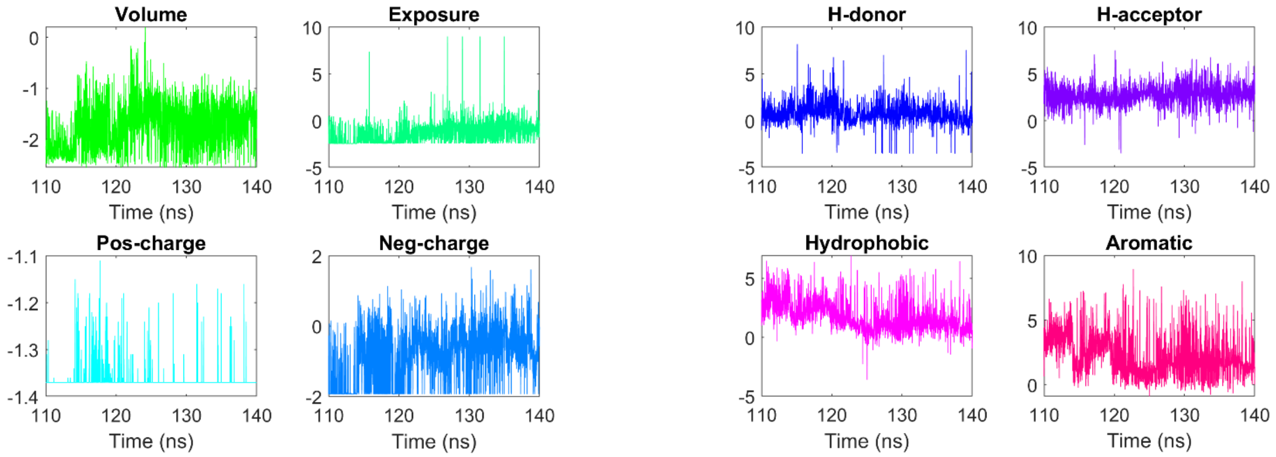

Figure S83. TRAPP-pocket results for the unstable zone of 110-140 ns for C23.

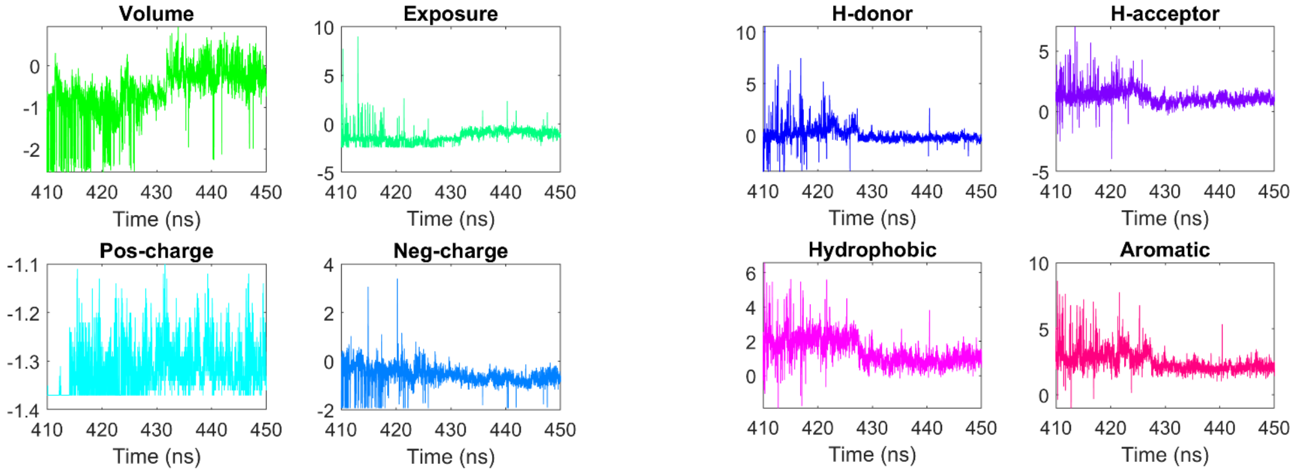

Figure S84. TRAPP-pocket results for the unstable zone of 410-450 ns for C23.

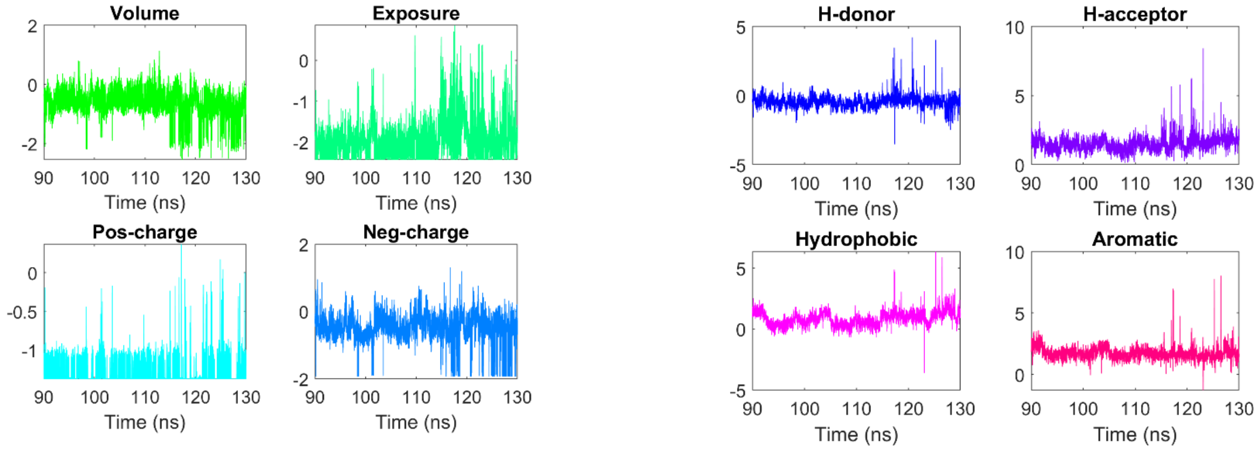

Figure S85. TRAPP-pocket results for the unstable zone of 90-130 ns for C31.

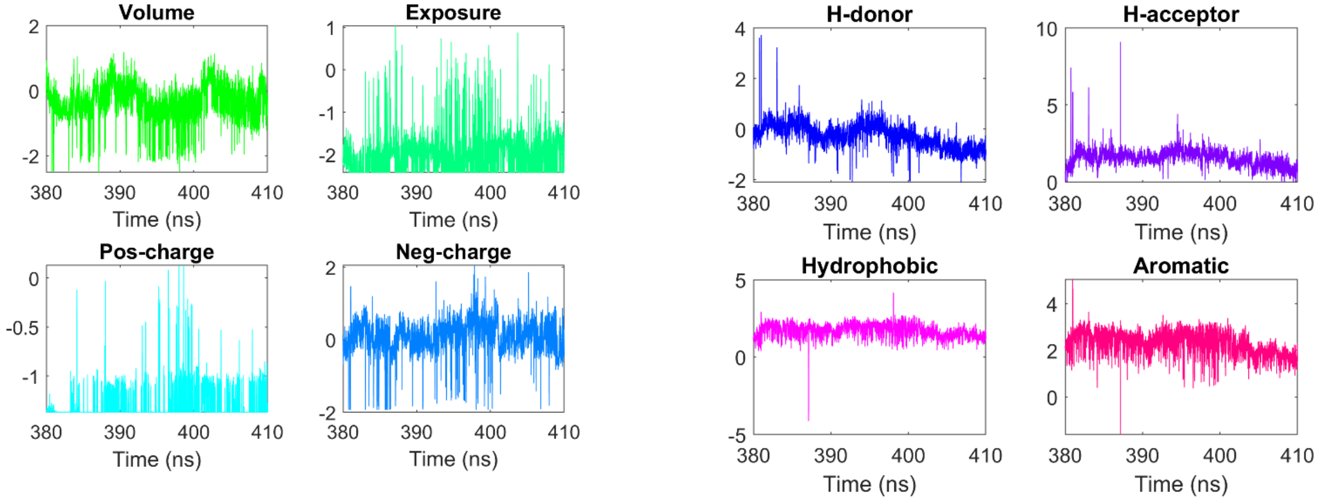

Figure S86. TRAPP-pocket results for the unstable zone of 380-410 ns for C31.

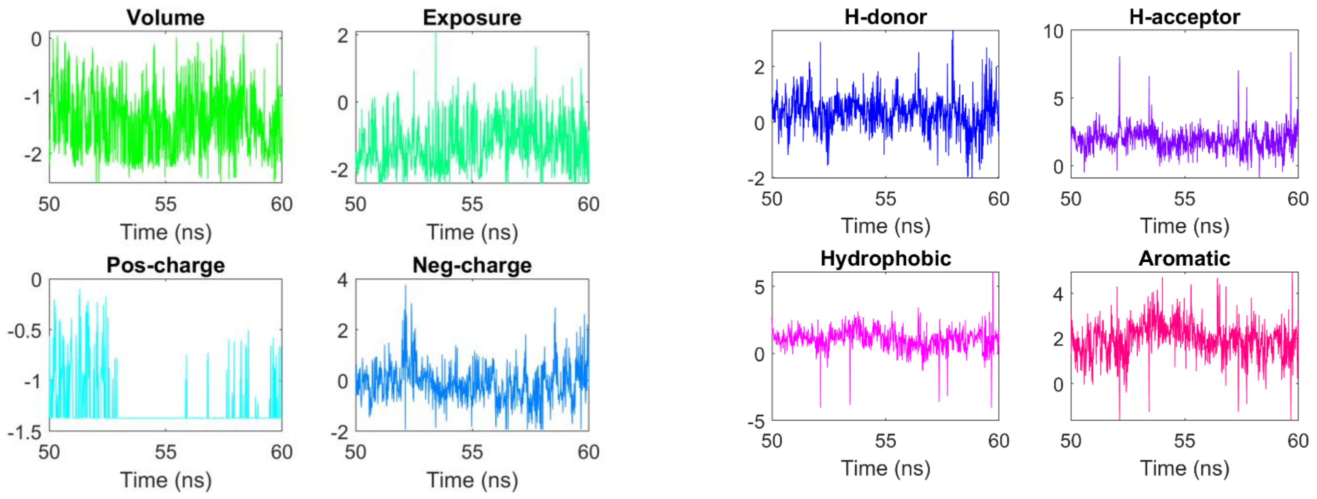

Figure S87. TRAPP-pocket results for the unstable zone of 50-60 ns for C35.

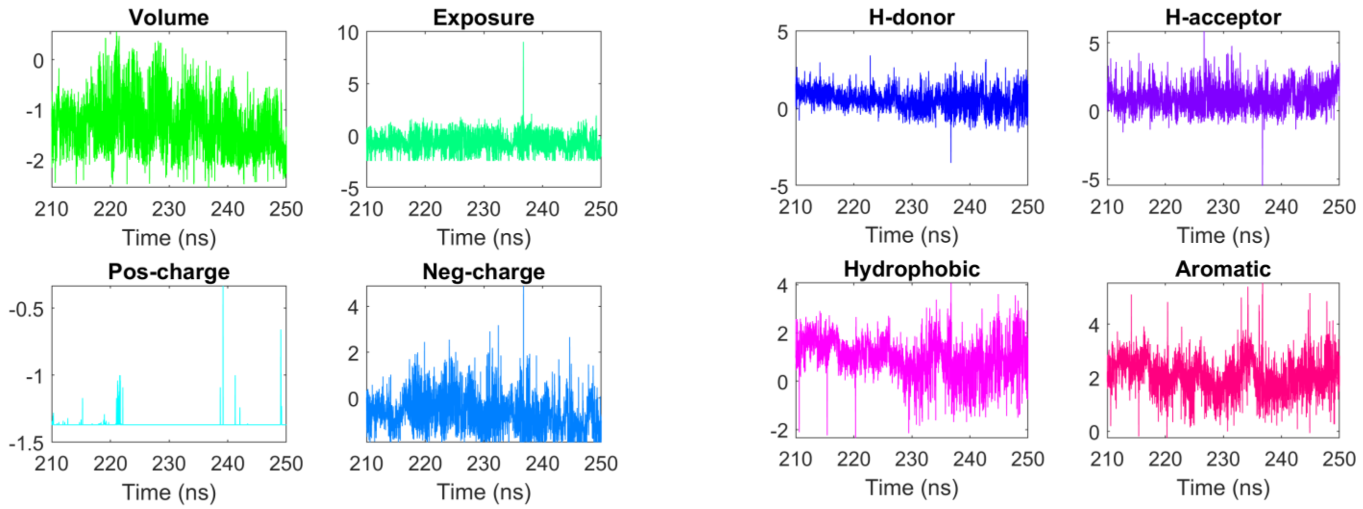

Figure S88. TRAPP-pocket results for the unstable zone of 210-250 ns for C35.

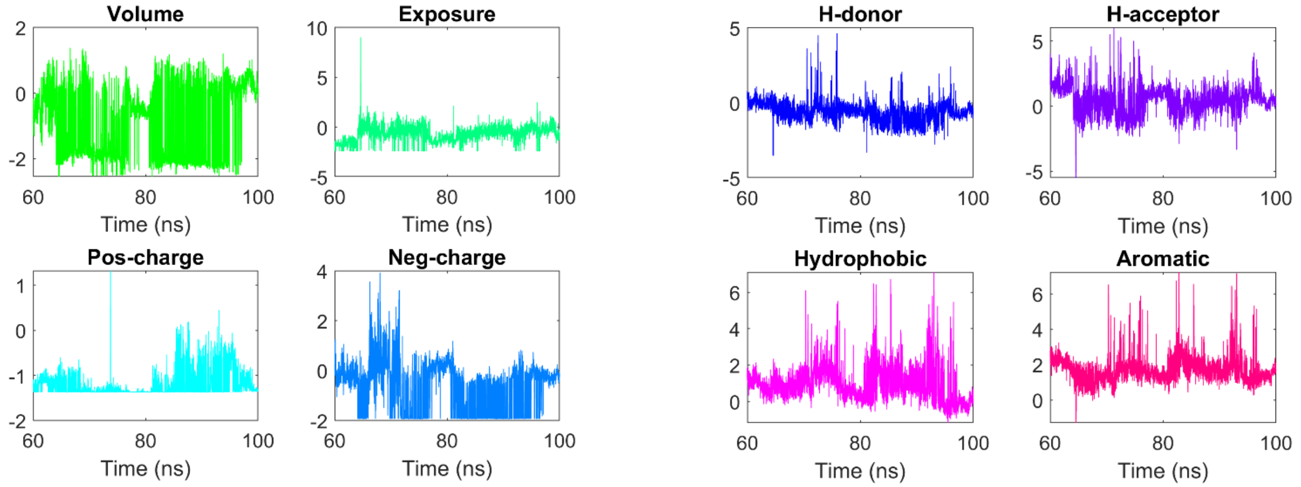

Figure S89. TRAPP-pocket results for the unstable zone of 60-100 ns for C36.

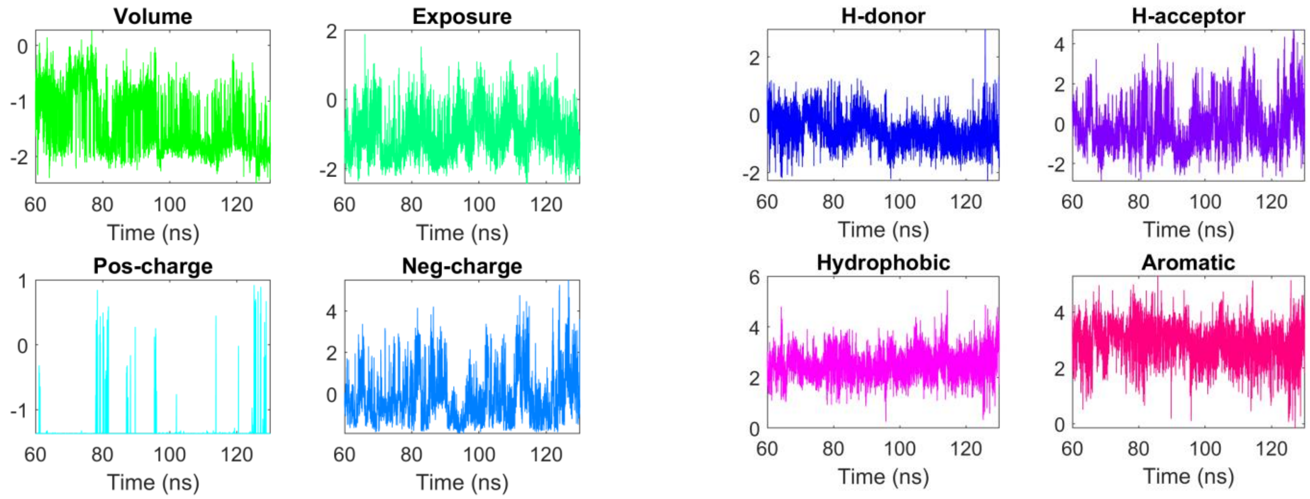

Figure S90. TRAPP-pocket results for the unstable zone of 60-100 ns for C42.

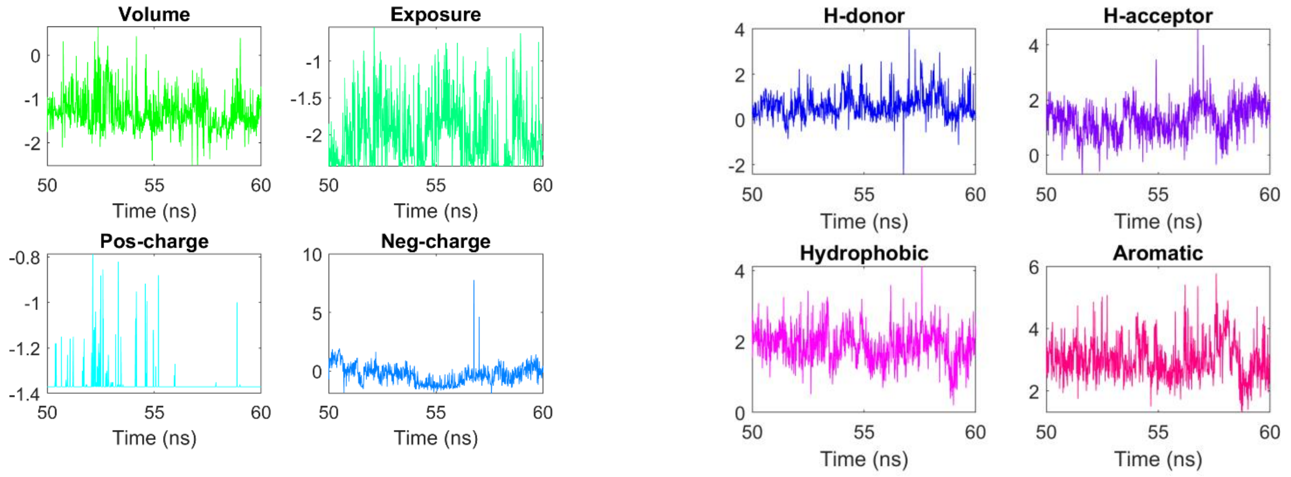

Figure S91. TRAPP-pocket results for the unstable zone of 50-60 ns for C50.

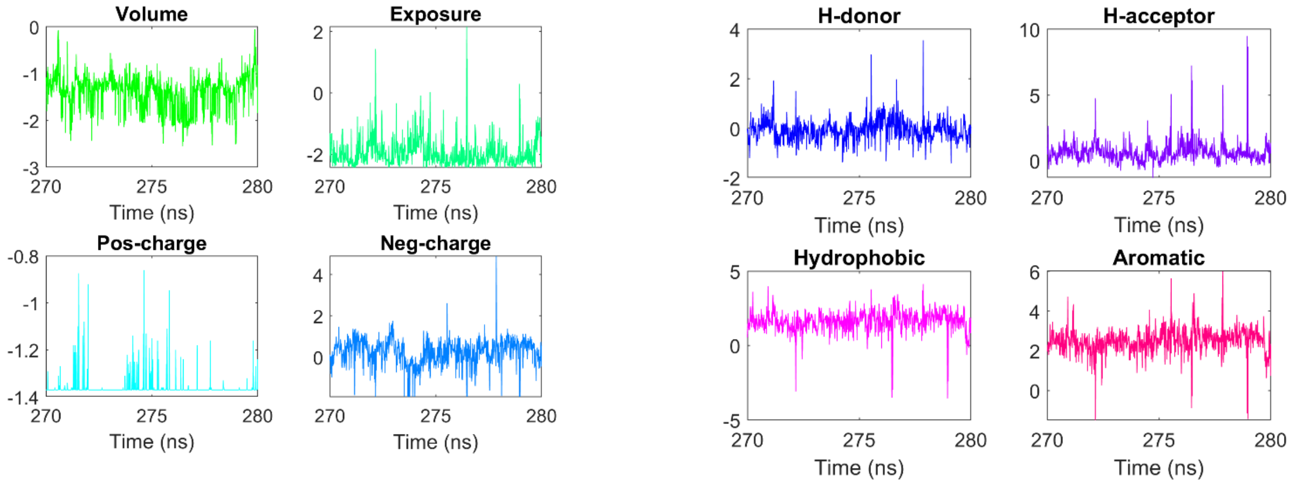

Figure S92. TRAPP-pocket results for the unstable zone of 270-280 ns for C50.

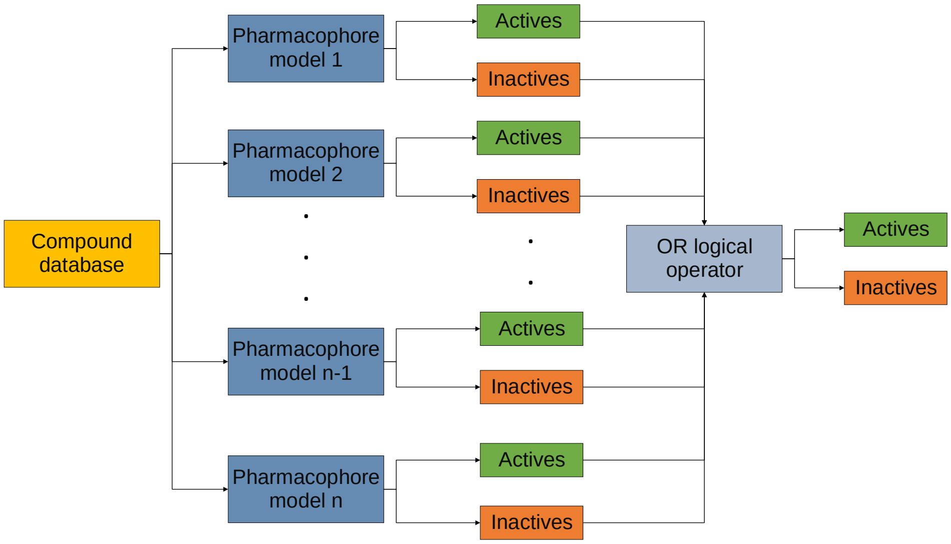

Figure S93. Scheme of a pharmacophore model ensemble.

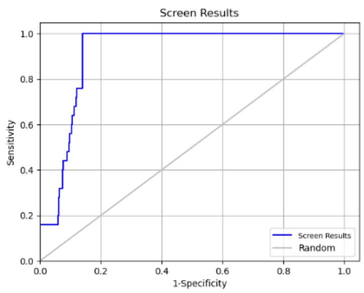

C-4 (AUC:0.91)

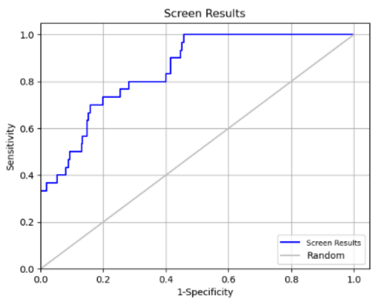

C-19 (AUC:0.85)

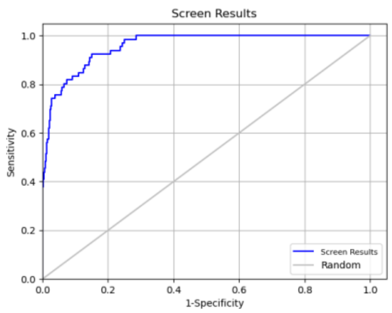

C-20 (AUC:0.96)

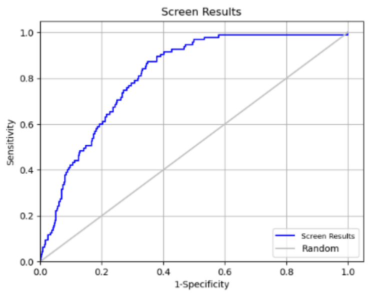

C-23 (AUC:0.81)

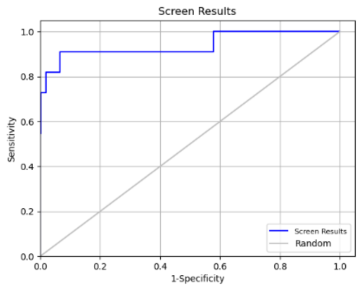

C-31 (AUC:0.94)

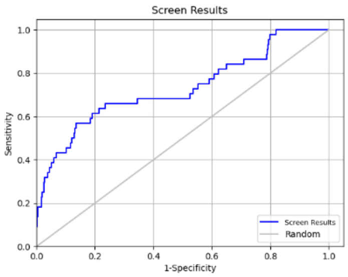

C-35 (AUC:0.73)

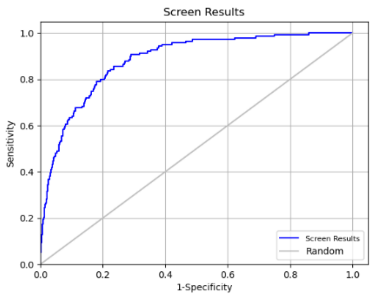

C-42 (AUC:0.88)

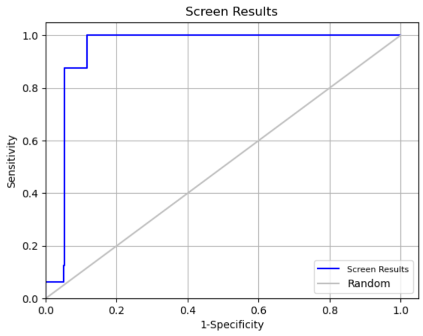

C-50 (AUC:0.94)

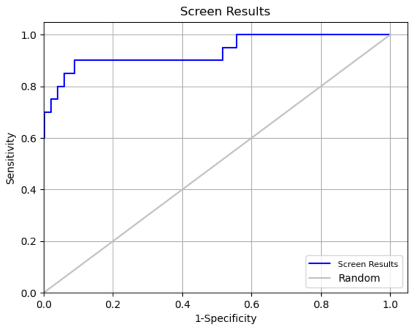

C-36 (AUC:0.94)

Figure S94. ROC curve for the validation of the ensemble pharmacophore models without YN2 application.

Table S18. Results of validation of pharmacophore model ensembles without YN2 application. TP: true positive, TN: true negative, FP: false positive, FN: false negative, RC: recall, SP: specificity, ACC: accuracy, BACC: balanced accuracy, ROC-AUC: area under ROC curve.

	**Cluster**								
	**C4**	**C19**	**C20**	**C23**	**C31**	**C35**	**C36**	**C42**	**C50**
**Actives**	25	30	66	95	11	44	20	139	16
**Decoys**	1274	1517	3475	5643	546	3467	2079	8218	820
**Total**	1299	1547	3541	5738	557	3511	2099	8357	836
**TP**	25	30	66	94	11	44	20	139	16
**TN**	1063	79	475	1244	22	621	1	767	443
**FP**	211	1438	3000	4399	524	2846	2078	7451	377
**FN**	0	0	0	1	0	0	0	0	0
**RC**	1.000	1.000	1.000	0.989	1.000	1.000	1.000	1.000	1.000
**SP**	0.834	0.052	0.137	0.220	0.040	0.179	0.000	0.093	0.540
**ACC**	0.838	0.070	0.153	0.233	0.059	0.189	0.010	0.108	0.549
**BACC**	0.917	0.526	0.568	0.605	0.520	0.590	0.500	0.547	0.770
**ROC AUC**	0.910	0.850	0.960	0.810	0.940	0.730	0.940	0.880	0.940

Table S19. Results of optimization for YN2 threshold for C4. TP: true positive, TN: true negative, FP: false positive, FN: false negative, Objective function is sum of recall and specificity. The values in red correspond optimal YN2 value that maximizes objective function.

YN2 threshold	TP	TN	FP	FN	Recall	Specificity	Objective Function
0.0898	25	1100	174	0	1.0000	0.8634	1.8634
0.1403	25	1111	163	0	1.0000	0.8721	1.8721
0.1909	25	1118	156	0	1.0000	0.8776	1.8776
0.2415	25	1134	140	0	1.0000	0.8901	1.8901
0.2920	25	1153	121	0	1.0000	0.9050	1.9050
0.3426	25	1183	91	0	1.0000	0.9286	1.9286
0.3932	21	1186	88	4	0.8400	0.9309	1.7709
0.4437	21	1193	81	4	0.8400	0.9364	1.7764
0.4943	19	1200	74	6	0.7600	0.9419	1.7019
0.5449	18	1215	59	7	0.7200	0.9537	1.6737
0.5954	18	1224	50	7	0.7200	0.9608	1.6808
0.6460	12	1232	42	13	0.4800	0.9670	1.4470
0.6966	4	1241	33	21	0.1600	0.9741	1.1341
0.7472	4	1261	13	21	0.1600	0.9898	1.1498
0.7977	4	1272	2	21	0.1600	0.9984	1.1584
0.8483	4	1273	1	21	0.1600	0.9992	1.1592
0.8989	4	1274	0	21	0.1600	1.0000	1.1600
0.9494	3	1274	0	22	0.1200	1.0000	1.1200

Table S20. Results of optimization for YN2 threshold for C19. TP: true positive, TN: true negative, FP: false positive, FN: false negative, Objective function is sum of recall and specificity. The values in red correspond optimal YN2 value that maximizes Objective function.

YN2 threshold	TP	TN	FP	FN	Recall	Specificity	Objective Function
0.1777	29	824	693	1	0.9667	0.5432	1.5098
0.2234	28	824	693	2	0.9333	0.5432	1.4765
0.2691	28	827	690	2	0.9333	0.5452	1.4785
0.3147	28	829	688	2	0.9333	0.5465	1.4798
0.3604	28	830	687	2	0.9333	0.5471	1.4805
0.4061	28	831	686	2	0.9333	0.5478	1.4811
0.4518	28	837	680	2	0.9333	0.5517	1.4851
0.4975	28	844	673	2	0.9333	0.5564	1.4897
0.5432	28	864	653	2	0.9333	0.5695	1.5029
0.5888	24	895	622	6	0.8000	0.5900	1.3900
0.6345	22	968	549	8	0.7333	0.6381	1.3714
0.6802	17	1059	458	13	0.5667	0.6981	1.2648
0.7259	14	1196	321	16	0.4667	0.7884	1.2551
0.7716	11	1376	141	19	0.3667	0.9071	1.2737
0.8173	10	1490	27	20	0.3333	0.9822	1.3155
0.8629	9	1513	4	21	0.3000	0.9974	1.2974
0.9086	7	1517	0	23	0.2333	1.0000	1.2333
0.9543	7	1517	0	23	0.2333	1.0000	1.2333

Table S21. Results of optimization for YN2 threshold for C20. TP: true positive, TN: true negative, FP: false positive, FN: false negative, Objective function is sum of recall and specificity. The values in red correspond optimal YN2 value that maximizes Objective function.

YN2 threshold	TP	TN	FP	FN	Recall	Specificity	Objective Function
0.0952	64	2480	995	2	0.9697	0.7137	1.6834
0.1648	63	2484	991	3	0.9545	0.7148	1.6694
0.2344	63	2486	989	3	0.9545	0.7154	1.6699
0.3040	63	2527	948	3	0.9545	0.7272	1.6817
0.3737	58	2581	894	8	0.8788	0.7427	1.6215
0.4433	56	2717	758	10	0.8485	0.7819	1.6304
0.5129	52	2752	723	14	0.7879	0.7919	1.5798
0.5826	49	2809	666	17	0.7424	0.8083	1.5508
0.6522	47	2949	526	19	0.7121	0.8486	1.5608
0.7218	38	3379	96	28	0.5758	0.9724	1.5481
0.7915	26	3464	11	40	0.3939	0.9968	1.3908
0.8611	14	3473	2	52	0.2121	0.9994	1.2115
0.9307	3	3473	2	63	0.0455	0.9994	1.0449
1.0004	0	3473	2	66	0.0000	0.9994	0.9994
1.0700	0	3473	2	66	0.0000	0.9994	0.9994
1.1396	0	3473	2	66	0.0000	0.9994	0.9994
1.2092	0	3473	2	66	0.0000	0.9994	0.9994
1.2789	0	3473	2	66	0.0000	0.9994	0.9994

Table S22. Results of optimization for YN2 threshold for C23. TP: true positive, TN: true negative, FP: false positive, FN: false negative, Objective function is sum of recall and specificity. The values in red correspond optimal YN2 value that maximizes Objective function.

YN2 threshold	TP	TN	FP	FN	Recall	Specificity	Objective Function
0.0775	94	2454	3189	1	0.9895	0.4349	1.4243
0.1353	90	2642	3001	5	0.9474	0.4682	1.4156
0.1931	88	2832	2811	7	0.9263	0.5019	1.4282
0.2508	88	3052	2591	7	0.9263	0.5408	1.4672
0.3086	88	3291	2352	7	0.9263	0.5832	1.5095
0.3664	86	3510	2133	9	0.9053	0.6220	1.5273
0.4242	85	3747	1896	10	0.8947	0.6640	1.5587
0.4820	77	3993	1650	18	0.8105	0.7076	1.5181
0.5398	72	4298	1345	23	0.7579	0.7617	1.5195
0.5976	60	4565	1078	35	0.6316	0.8090	1.4405
0.6554	55	4749	894	40	0.5789	0.8416	1.4205
0.7131	49	4918	725	46	0.5158	0.8715	1.3873
0.7709	46	5155	488	49	0.4842	0.9135	1.3977
0.8287	28	5385	258	67	0.2947	0.9543	1.2490
0.8865	10	5533	110	85	0.1053	0.9805	1.0858
0.9443	4	5607	36	91	0.0421	0.9936	1.0357
1.0021	0	5632	11	95	0.0000	0.9981	0.9981
1.0599	0	5636	7	95	0.0000	0.9988	0.9988

Table S23. Results of optimization for YN2 threshold for C31. TP: true positive, TN: true negative, FP: false positive, FN: false negative, Objective function is sum of recall and specificity. The values in red correspond optimal YN2 value that maximizes Objective function.

YN2 threshold	TP	TN	FP	FN	Recall	Specificity	Objective Function
0.4395	10	232	314	1	0.9091	0.4249	1.3340
0.4706	10	233	313	1	0.9091	0.4267	1.3358
0.5018	10	234	312	1	0.9091	0.4286	1.3377
0.5329	10	234	312	1	0.9091	0.4286	1.3377
0.5640	10	234	312	1	0.9091	0.4286	1.3377
0.5952	10	235	311	1	0.9091	0.4304	1.3395
0.6263	10	238	308	1	0.9091	0.4359	1.3450
0.6575	10	254	292	1	0.9091	0.4652	1.3743
0.6886	10	333	213	1	0.9091	0.6099	1.5190
0.7197	9	440	106	2	0.8182	0.8059	1.6240
0.7509	9	505	41	2	0.8182	0.9249	1.7431
0.7820	9	531	15	2	0.8182	0.9725	1.7907
0.8132	7	542	4	4	0.6364	0.9927	1.6290
0.8443	5	545	1	6	0.4545	0.9982	1.4527
0.8754	5	545	1	6	0.4545	0.9982	1.4527
0.9066	4	546	0	7	0.3636	1.0000	1.3636
0.9377	3	546	0	8	0.2727	1.0000	1.2727
0.9689	3	546	0	8	0.2727	1.0000	1.2727

Table S24. Results of optimization for YN2 threshold for C35. TP: true positive, TN: true negative, FP: false positive, FN: false negative, Objective function is sum of recall and specificity. The values in red correspond optimal YN2 value that maximizes Objective function.

YN2 threshold	TP	TN	FP	FN	Recall	Specificity	Objective Function
0.0632	43	474	2993	1	0.9773	0.1367	1.1140
0.1152	43	489	2978	1	0.9773	0.1410	1.1183
0.1673	41	508	2959	3	0.9318	0.1465	1.0783
0.2193	38	533	2934	6	0.8636	0.1537	1.0174
0.2713	38	611	2856	6	0.8636	0.1762	1.0399
0.3234	38	697	2770	6	0.8636	0.2010	1.0647
0.3754	38	740	2727	6	0.8636	0.2134	1.0771
0.4275	38	774	2693	6	0.8636	0.2232	1.0869
0.4795	38	812	2655	6	0.8636	0.2342	1.0978
0.5316	34	843	2624	10	0.7727	0.2431	1.0159
0.5836	33	885	2582	11	0.7500	0.2553	1.0053
0.6357	32	931	2536	12	0.7273	0.2685	0.9958
0.6877	32	1010	2457	12	0.7273	0.2913	1.0186
0.7398	30	1143	2324	14	0.6818	0.3297	1.0115
0.7918	30	1243	2224	14	0.6818	0.3585	1.0403
0.8439	30	1391	2076	14	0.6818	0.4012	1.0830
0.8959	30	1675	1792	14	0.6818	0.4831	1.1649
0.9480	19	3223	244	25	0.4318	0.9296	1.3614

Table S25. Results of optimization for YN2 threshold for C36. TP: true positive, TN: true negative, FP: false positive, FN: false negative, Objective function is sum of recall and specificity. The values in red correspond optimal YN2 value that maximizes Objective function.

YN2 threshold	TP	TN	FP	FN	Recall	Specificity	Objective Function
0.2539	20	922	1157	0	1.0000	0.4435	1.4435
0.2953	20	923	1156	0	1.0000	0.4440	1.4440
0.3368	20	946	1133	0	1.0000	0.4550	1.4550
0.3782	20	974	1105	0	1.0000	0.4685	1.4685
0.4197	20	989	1090	0	1.0000	0.4757	1.4757
0.4611	19	1023	1056	1	0.9500	0.4921	1.4421
0.5026	18	1076	1003	2	0.9000	0.5176	1.4176
0.5440	18	1233	846	2	0.9000	0.5931	1.4931
0.5855	17	1423	656	3	0.8500	0.6845	1.5345
0.6269	17	1608	471	3	0.8500	0.7734	1.6234
0.6684	16	1749	330	4	0.8000	0.8413	1.6413
0.7098	16	1839	240	4	0.8000	0.8846	1.6846
0.7513	13	1937	142	7	0.6500	0.9317	1.5817
0.7927	12	2008	71	8	0.6000	0.9658	1.5658
0.8342	11	2048	31	9	0.5500	0.9851	1.5351
0.8756	11	2070	9	9	0.5500	0.9957	1.5457
0.9171	8	2077	2	12	0.4000	0.9990	1.3990
0.9585	6	2079	0	14	0.3000	1.0000	1.3000

Table S26. Results of optimization for YN2 threshold for C42. TP: true positive, TN: true negative, FP: false positive, FN: false negative, Objective function is sum of recall and specificity. The values in red correspond optimal YN2 value that maximizes Objective function.

YN2 threshold	TP	TN	FP	FN	Recall	Specificity	Objective Function
0.0995	139	1171	7047	0	1.0000	0.1425	1.1425
0.1776	139	1248	6970	0	1.0000	0.1519	1.1519
0.2558	139	1333	6885	0	1.0000	0.1622	1.1622
0.3339	139	1467	6751	0	1.0000	0.1785	1.1785
0.4121	139	1797	6421	0	1.0000	0.2187	1.2187
0.4902	135	2151	6067	4	0.9712	0.2617	1.2330
0.5684	129	2498	5720	10	0.9281	0.3040	1.2320
0.6466	122	2962	5256	17	0.8777	0.3604	1.2381
0.7247	114	3875	4343	25	0.8201	0.4715	1.2917
0.8029	79	7032	1186	60	0.5683	0.8557	1.4240
0.8810	9	8196	22	130	0.0647	0.9973	1.0621
0.9592	1	8210	8	138	0.0072	0.9990	1.0062
1.0373	0	8210	8	139	0.0000	0.9990	0.9990
1.1155	0	8210	8	139	0.0000	0.9990	0.9990
1.1936	0	8211	7	139	0.0000	0.9991	0.9991
1.2718	0	8211	7	139	0.0000	0.9991	0.9991
1.3499	0	8212	6	139	0.0000	0.9993	0.9993
1.4281	0	8212	6	139	0.0000	0.9993	0.9993

Table S27. Results of optimization for YN2 threshold for C50. TP: true positive, TN: true negative, FP: false positive, FN: false negative, Objective function is sum of recall and specificity. The values in red correspond optimal YN2 value that maximizes Objective function.

YN2 threshold	TP	TN	FP	FN	Recall	Specificity	Objective Function
0.3567	16	725	95	0	1.0000	0.8841	1.8841
0.3924	16	726	94	0	1.0000	0.8854	1.8854
0.4282	16	726	94	0	1.0000	0.8854	1.8854
0.4639	16	727	93	0	1.0000	0.8866	1.8866
0.4996	16	727	93	0	1.0000	0.8866	1.8866
0.5354	16	728	92	0	1.0000	0.8878	1.8878
0.5711	16	729	91	0	1.0000	0.8890	1.8890
0.6069	16	733	87	0	1.0000	0.8939	1.8939
0.6426	14	738	82	2	0.8750	0.9000	1.7750
0.6783	1	791	29	15	0.0625	0.9646	1.0271
0.7141	1	791	29	15	0.0625	0.9646	1.0271
0.7498	1	795	25	15	0.0625	0.9695	1.0320
0.7856	1	800	20	15	0.0625	0.9756	1.0381
0.8213	1	808	12	15	0.0625	0.9854	1.0479
0.8570	1	818	2	15	0.0625	0.9976	1.0601
0.8928	1	820	0	15	0.0625	1.0000	1.0625
0.9285	1	820	0	15	0.0625	1.0000	1.0625
0.9643	1	820	0	15	0.0625	1.0000	1.0625

Table S28. Parameter values of pharmacophore model ensembles with YN2 application

Cluster	Number of stable conformations	Number of unstable conformations	PhaseScoreMin	PhaseScoreMax	YN2 MIN
C4	8	24	1.166	2.646	0.343
C19	9	23	2.047	2.880	0.178
C20	4	15	1.768	2.557	0.095
C23	7	24	1.389	1.931	0.424
C31	8	25	1.593	2.461	0.782
C35	8	30	0.161	2.008	0.948
C36	5	12	1.667	2.837	0.710
C42	4	17	1.240	2.206	0.803
C50	9	24	0.383	2.525	0.607

Table S29. Results of validation of pharmacophore model ensembles with YN2 application. TP: true positive, TN: true negative, FP: false positive, FN: false negative, RC: recall, SP: specificity, ACC: accuracy, BACC: balanced accuracy, ROC-AUC: area under ROC curve.

	**Cluster**								
	**C4**	**C19**	**C20**	**C23**	**C31**	**C35**	**C36**	**C42**	**C50**
**Actives**	25	30	66	95	11	44	20	139	16
**Decoys**	1274	1517	3475	5643	546	3467	2079	8218	820
**Total**	1299	1547	3541	5738	557	3511	2099	8357	836
**TP**	25	29	64	85	9	19	16	79	16
**TN**	1183	824	2480	3747	531	3223	1839	7032	733
**FP**	91	693	995	1896	15	244	240	1186	87
**FN**	0	1	2	10	2	25	4	60	0
**RC**	1.000	0.967	0.970	0.895	0.818	0.432	0.800	0.568	1.000
**SP**	0.929	0.543	0.714	0.664	0.973	0.930	0.885	0.856	0.894
**ACC**	0.930	0.551	0.718	0.668	0.969	0.923	0.884	0.851	0.896
**BACC**	0.964	0.755	0.842	0.779	0.895	0.681	0.842	0.712	0.947
**ROC AUC**	0.96	0.79	0.92	0.84	0.92	0.69	0.91	0.8	0.94

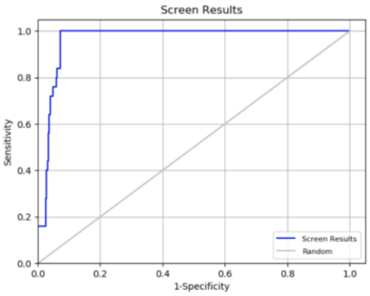

C-4 (AUC:0.96)

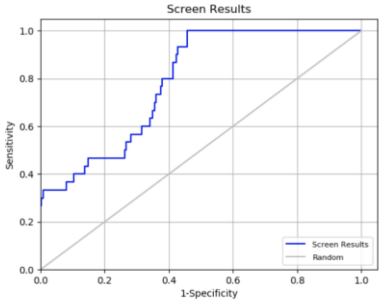

C-19 (AUC:0.79)

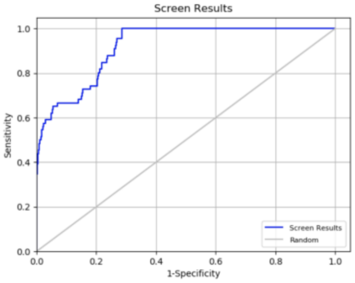

C-20 (AUC:0.92)

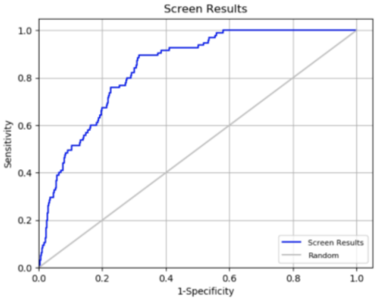

C-23 (AUC:0.84)

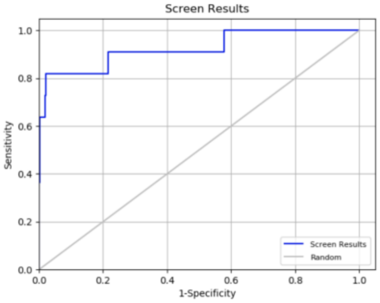

C-31 (AUC:0.92)

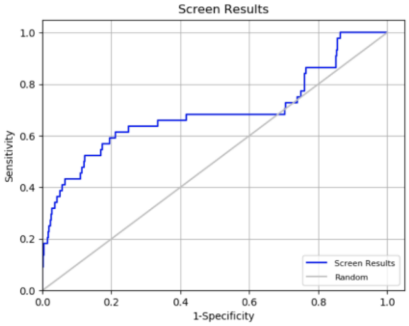

C-35 (AUC:0.69)

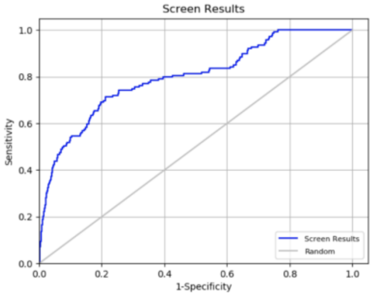

C-42 (AUC:0.80)

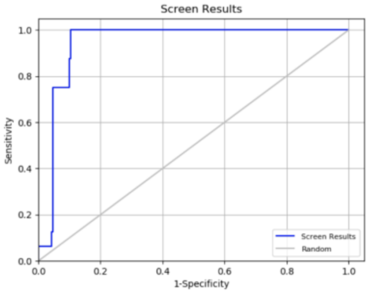

C-50 (AUC:0.94)

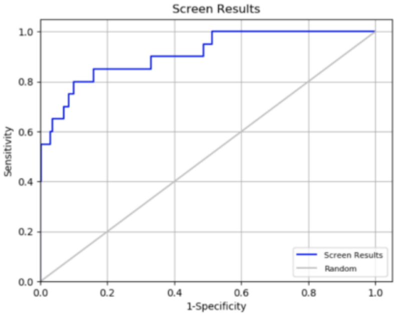

C-36 (AUC:0.91)

Figure S95. ROC curve for the validation of the ensemble pharmacophore models with YN2 application.

Table S30. Percentage improvement of recall and specificity for the ensembles using and not using YN2. A positive percentage indicates improvement, while a negative percentage indicates worsening.

**Recall**	0.0%	-3.3%	-3.0%	-9.6%	-18.2%	-56.8%	-20.0%	-43.2%	0.0%
**Specificity**	11.3%	943.0%	422.1%	201.2%	2313.6%	419.0%	183800.0%	816.8%	65.5%

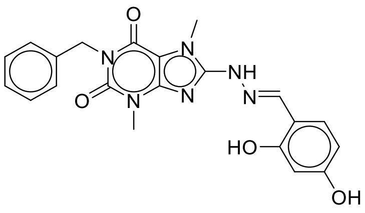	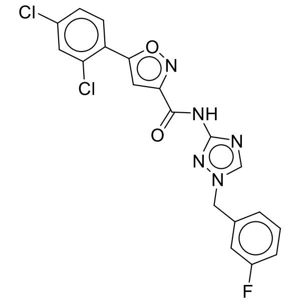	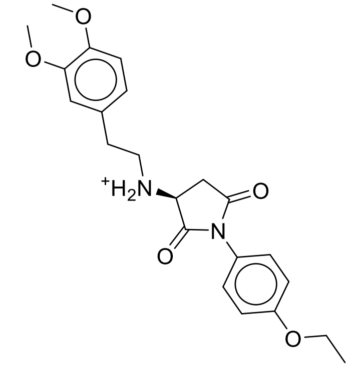
**C4-ZINC10274013 (-75.84 kJ/mol)**	**C4-ZINC2799745 (-74.43 kJ/mol)**	**C19-ZINC852356 (-68.65 kJ/mol)**
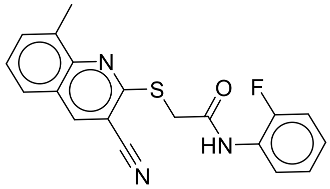	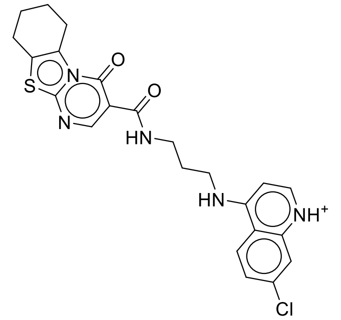	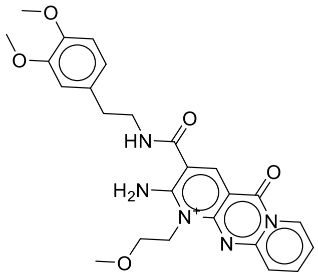
**C19-ZINC872638 (-67.62 kJ/mol)**	**C20-ZINC223043037 (-96.11 kJ/mol)**	**C20-ZINC100759000 (-89.09 kJ/mol)**
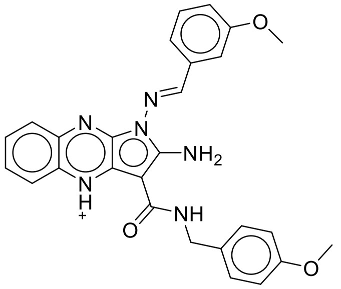	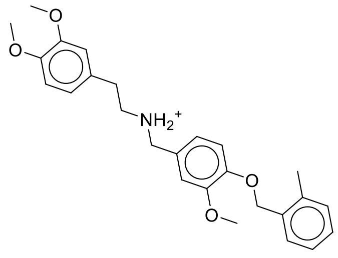	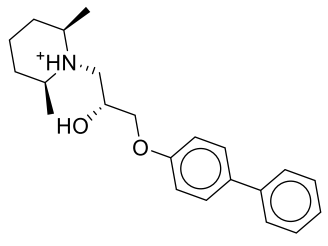
**C23-ZINC2256546 (-95.31 kJ/mol)**	**C23-ZINC16362780 (-83.7 kJ/mol)**	**C31-ZINC17245175 (-63.42 kJ/mol)**

Figure S96. Molecular structures of the two compounds with the lowest MMGBSA binding energy for C4, C19, C20, C23, C31 families from the virtual screening of the ZINC library.

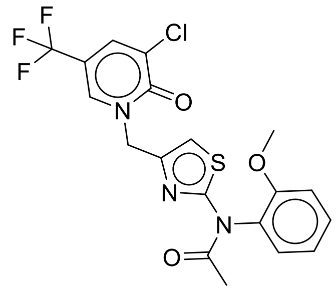	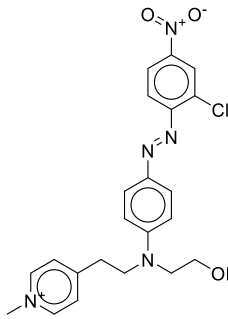	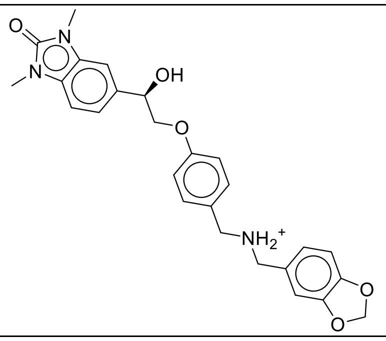
**C31-ZINC22983357 (-62.77 kJ/mol)**	**C35-ZINC18206771 (-115.69 kJ/mol)**	**C35-ZINC16363937 (-87.28 kJ/mol)**
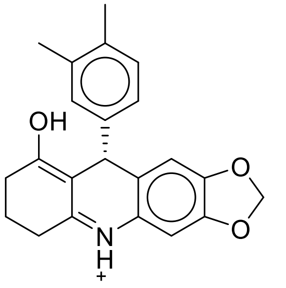	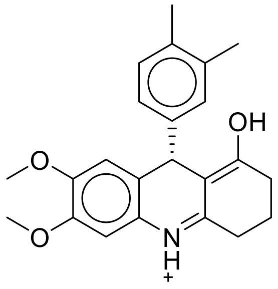	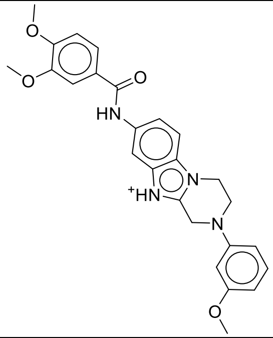
**C36-ZINC18196844 (-77.33 kJ/mol)**	**C36-ZINC18197226 (-76.77 kJ/mol)**	**C42-ZINC13691092 (-92.37 kJ/mol)**
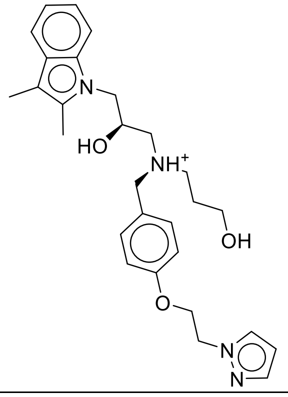	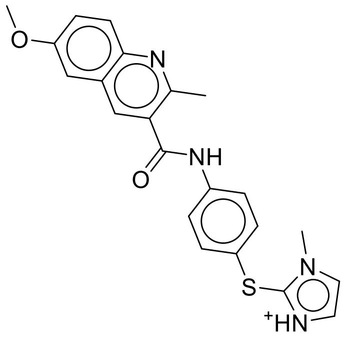	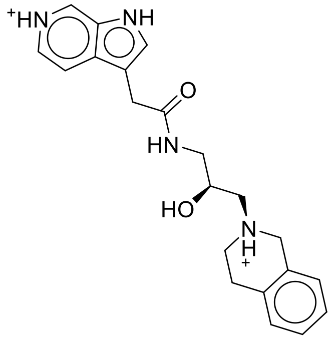
**C42-ZINC585270119 (-85.89 kJ/mol)**	**C50-ZINC58382287 (-91.71 kJ/mol)**	**C50-ZINC2325711293 (-88.87 kJ/mol)**

Figure S97. Molecular structures of the two compounds with the lowest MMGBSA binding energy for C31, C35, C36, C42, C50 families from the virtual screening of the ZINC library.

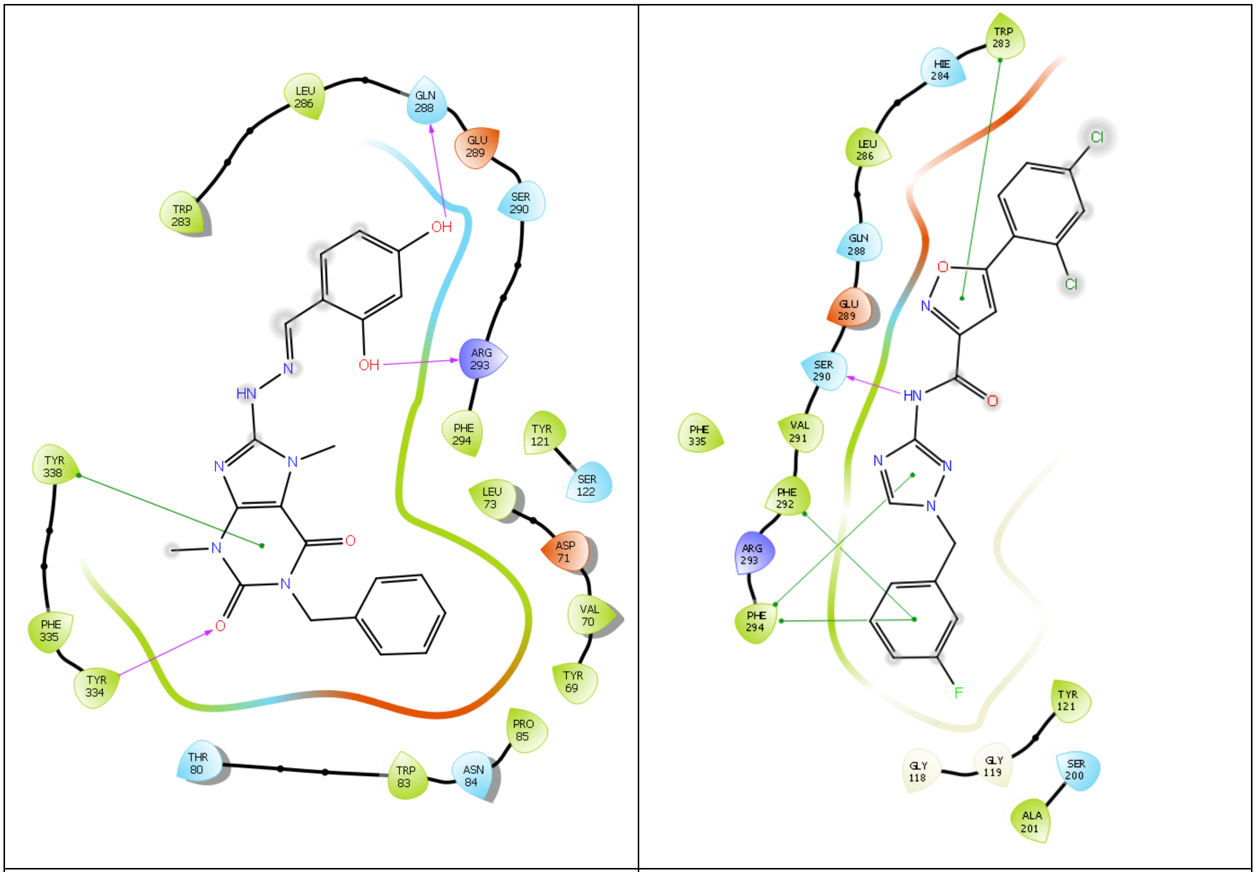

C4-ZINC10274013 (-75.84 kJ/mol)	C4-ZINC2799745 (-74.43 kJ/mol)

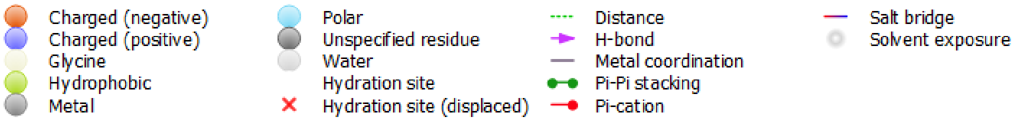

Figure S98. 2D Interaction diagrams of the two compounds with the lowest MMGBSA binding energy for C4 family from the virtual screening of the ZINC library.

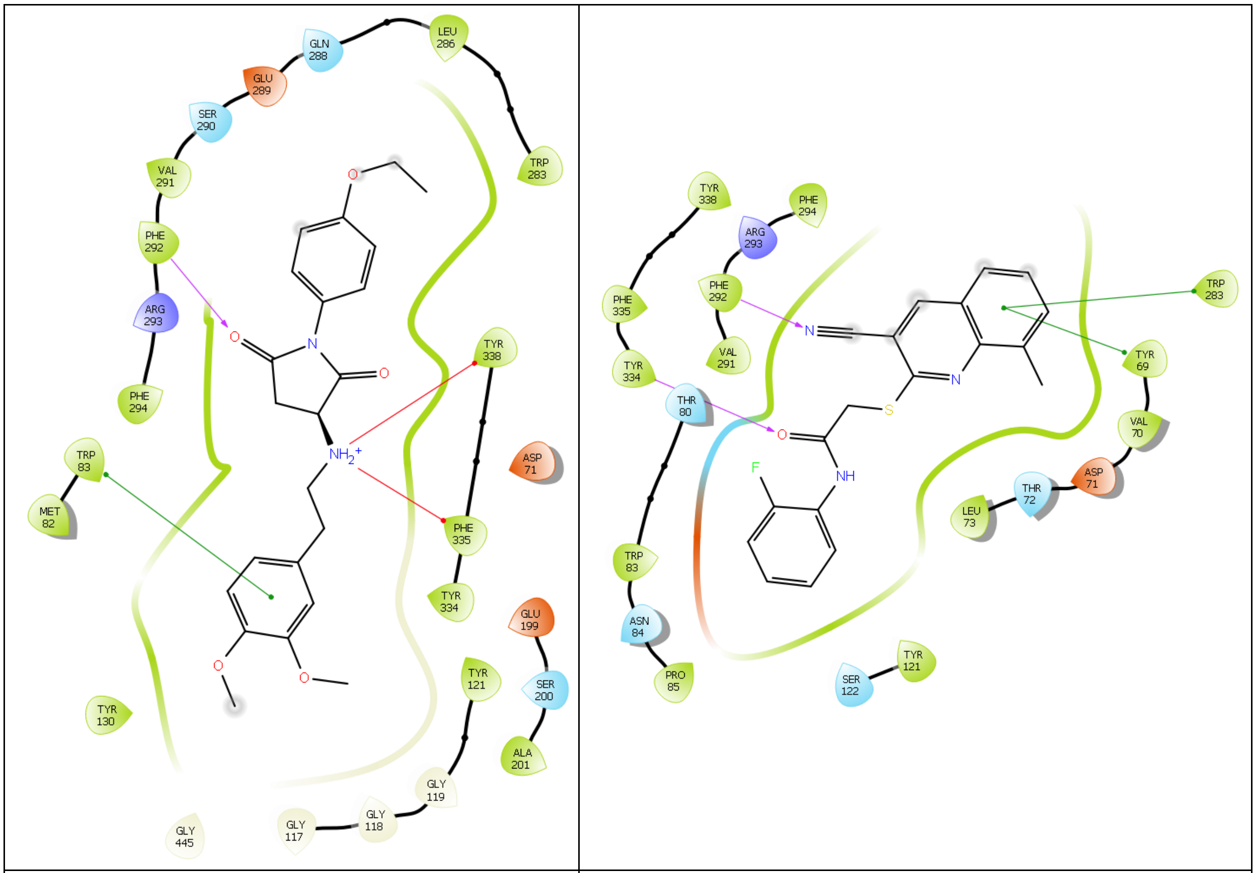

C19-ZINC852356 (-68.65 kJ/mol)	C19-ZINC872638 (-67.62 kJ/mol)

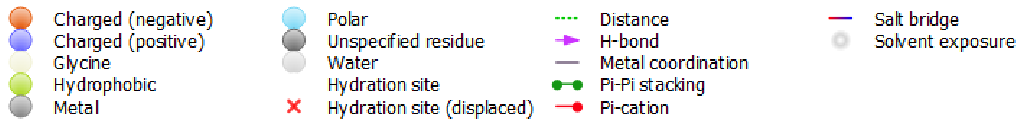

Figure S99. 2D Interaction diagrams of the two compounds with the lowest MMGBSA binding energy for C19 family from the virtual screening of the ZINC library.

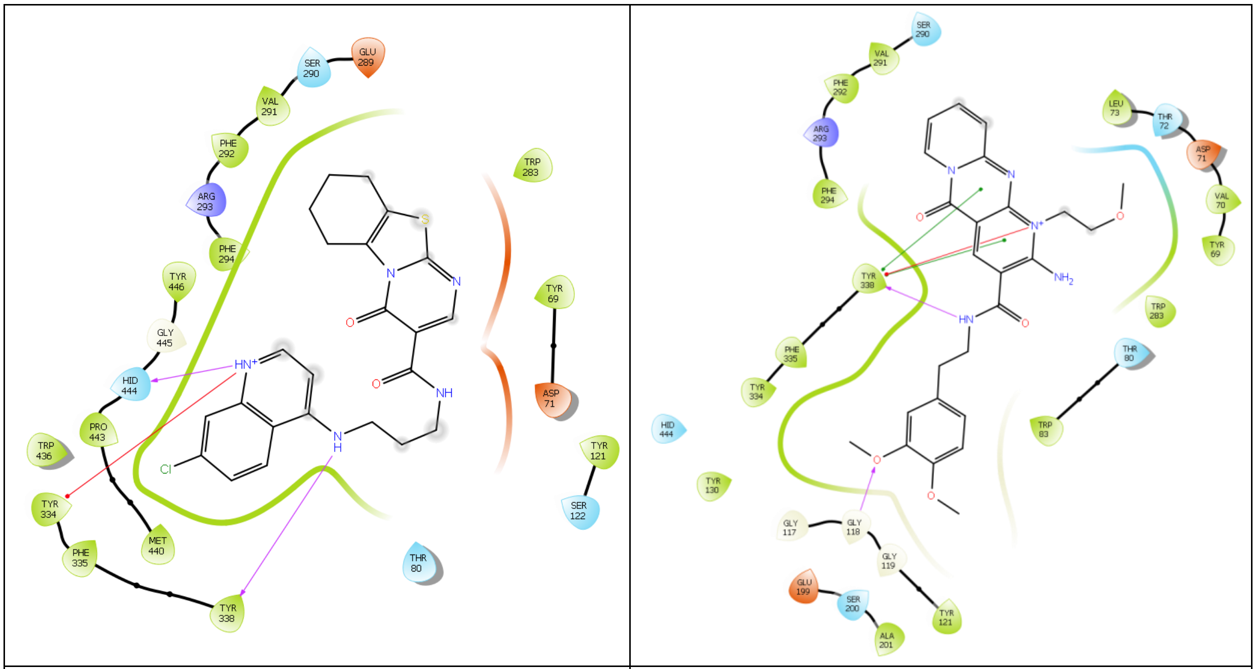

C20-ZINC223043037 (-96.11 kJ/mol)	C20-ZINC100759000 (-89.09 kJ/mol)

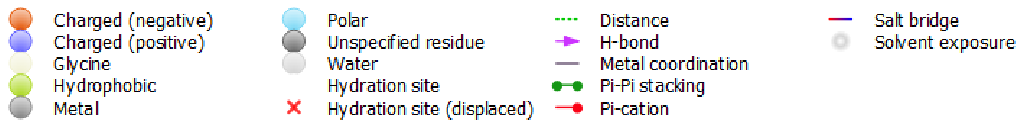

Figure S100. 2D Interaction diagrams of the two compounds with the lowest MMGBSA binding energy for C20 family from the virtual screening of the ZINC library.

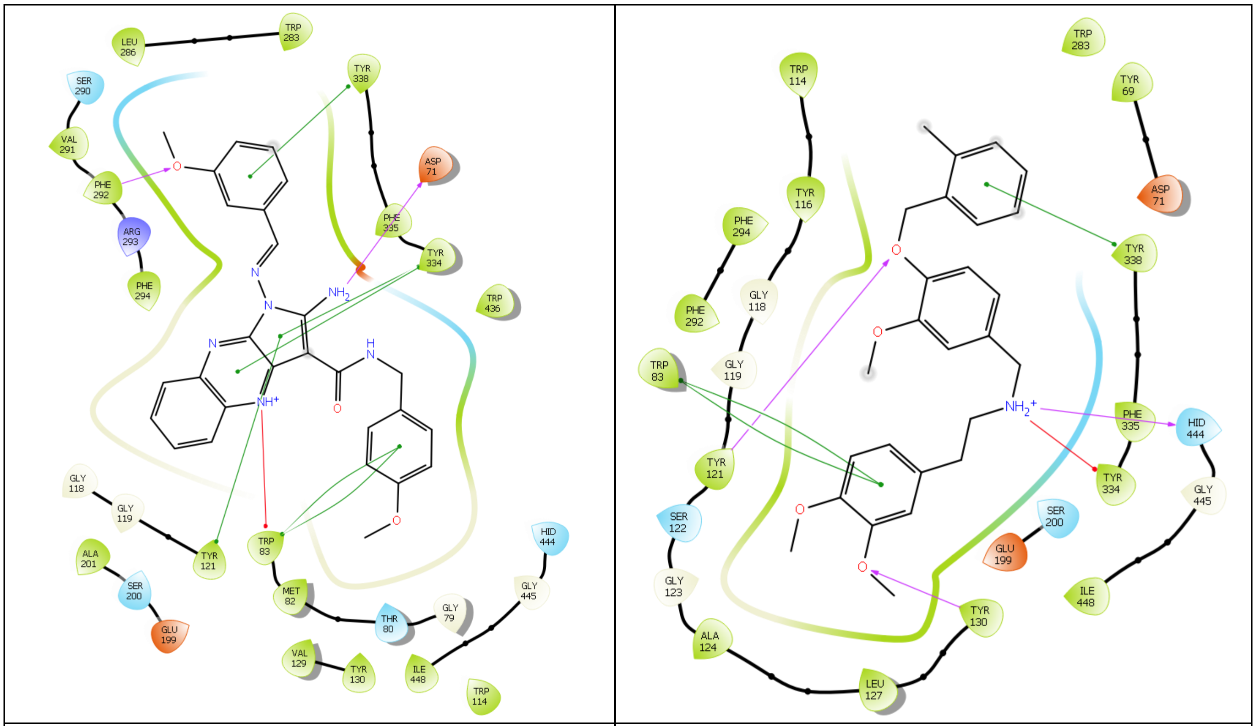

C23-ZINC2256546 (-95.31 kJ/mol)	C23-ZINC16362780 (-83.7 kJ/mol)

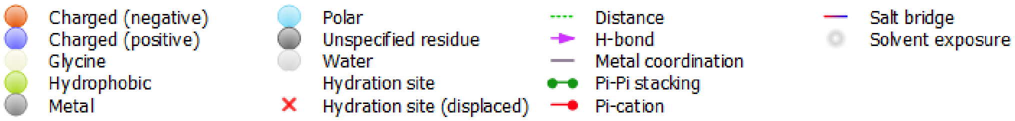

Figure S101. 2D Interaction diagrams of the two compounds with the lowest MMGBSA binding energy for C23 family from the virtual screening of the ZINC library.

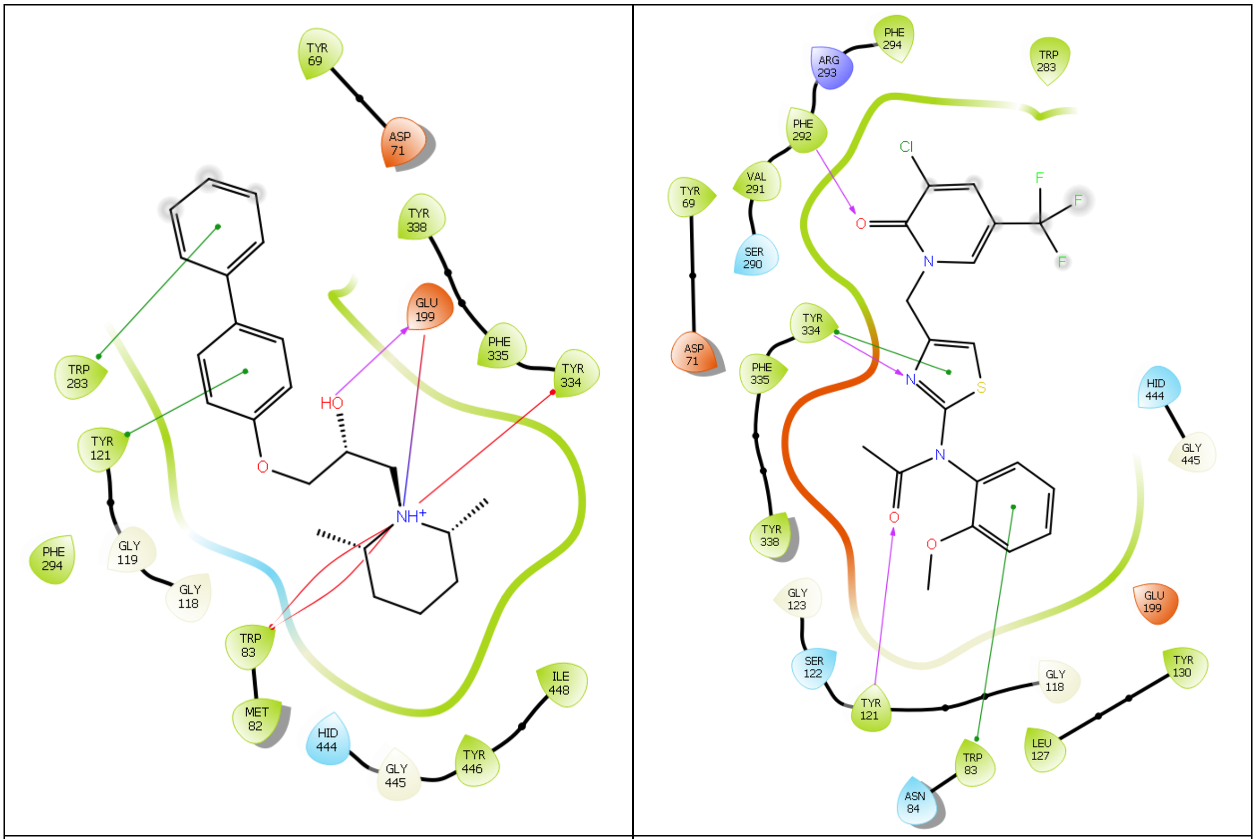

C31-ZINC17245175 (-63.42 kJ/mol)	C31-ZINC22983357 (-62.77 kJ/mol)

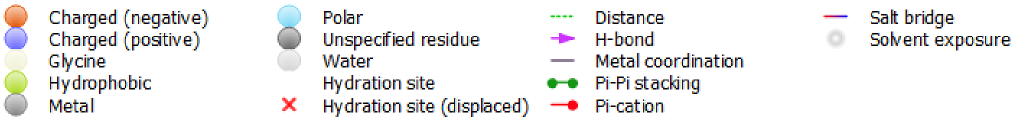

Figure S102. 2D Interaction diagrams of the two compounds with the lowest MMGBSA binding energy for C31 family from the virtual screening of the ZINC library.

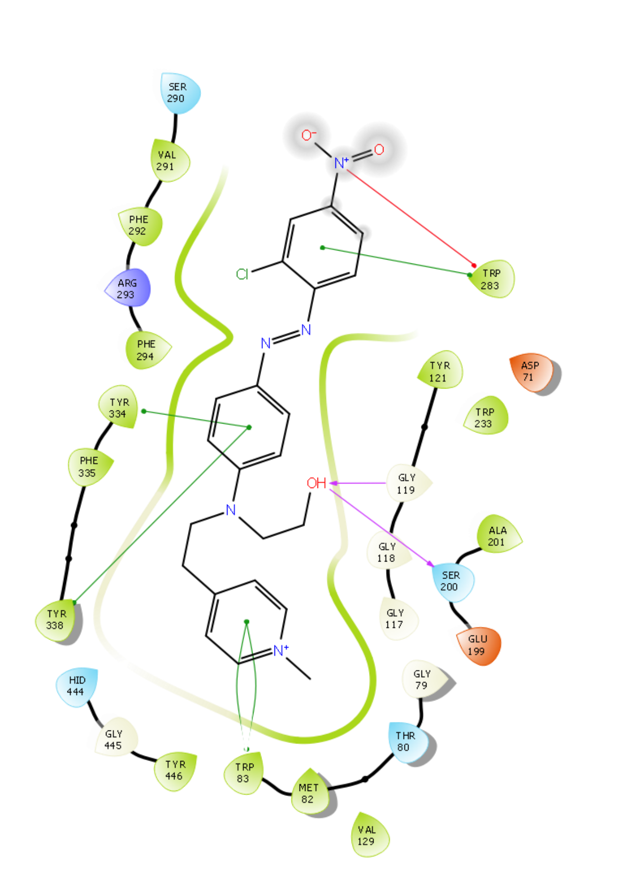

C35-ZINC18206771 (-115.69 kJ/mol)

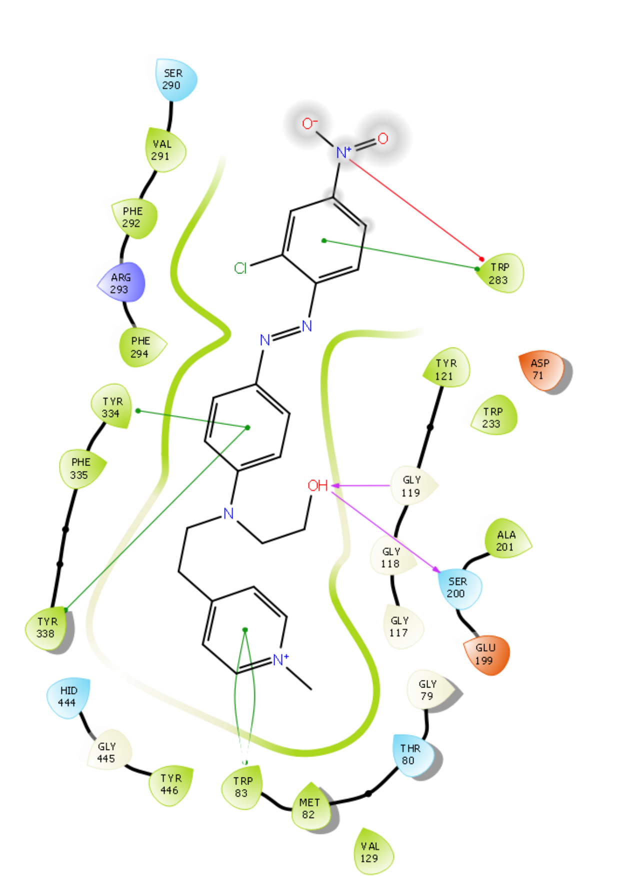

C35-ZINC16363937 (-87.28 kJ/mol)

- Charged (negative)
- Charged (positive)
- Glycine
- Hydrophobic
- Metal
- Polar
- Unspecified residue
- Water
- Hydration site
- Hydration site (displaced)
- Distance
- H-bond
- Metal coordination
- Pi-Pi stacking
- Pi-cation
- Salt bridge
- Solvent exposure

Figure S103. 2D Interaction diagrams of the two compounds with the lowest MMGBSA binding energy for C35 family from the virtual screening of the ZINC library.

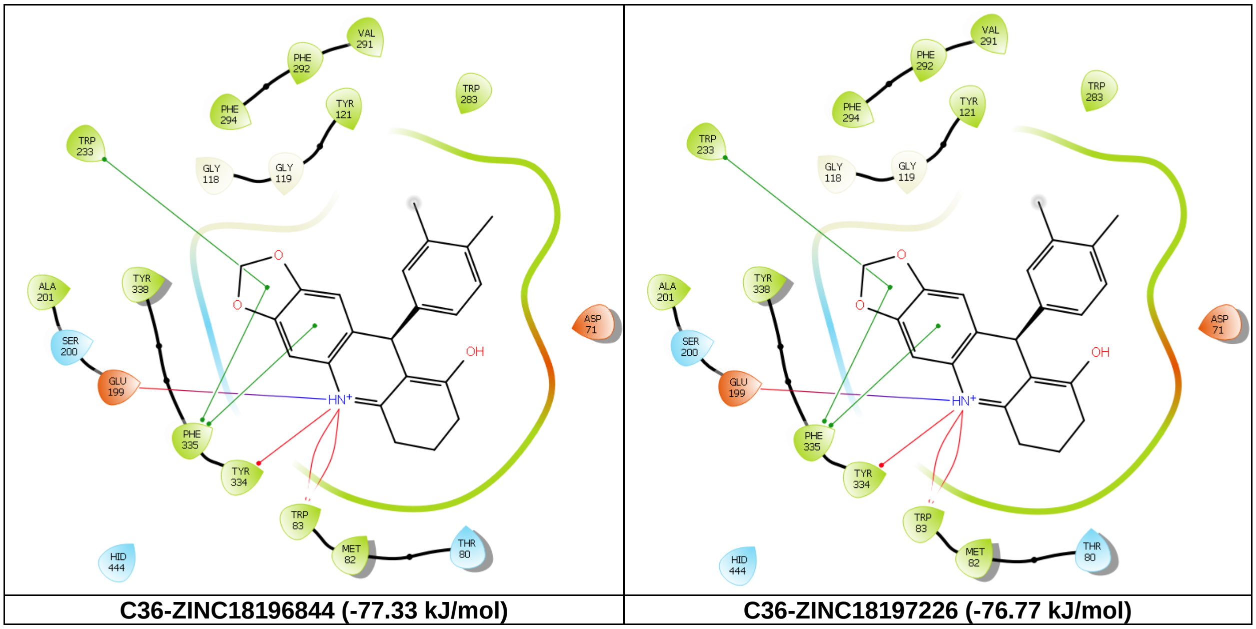

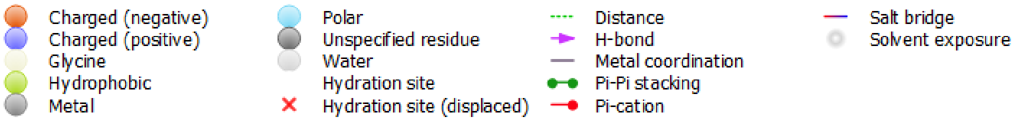

Figure S104. 2D Interaction diagrams of the two compounds with the lowest MMGBSA binding energy for C36 family from the virtual screening of the ZINC library.

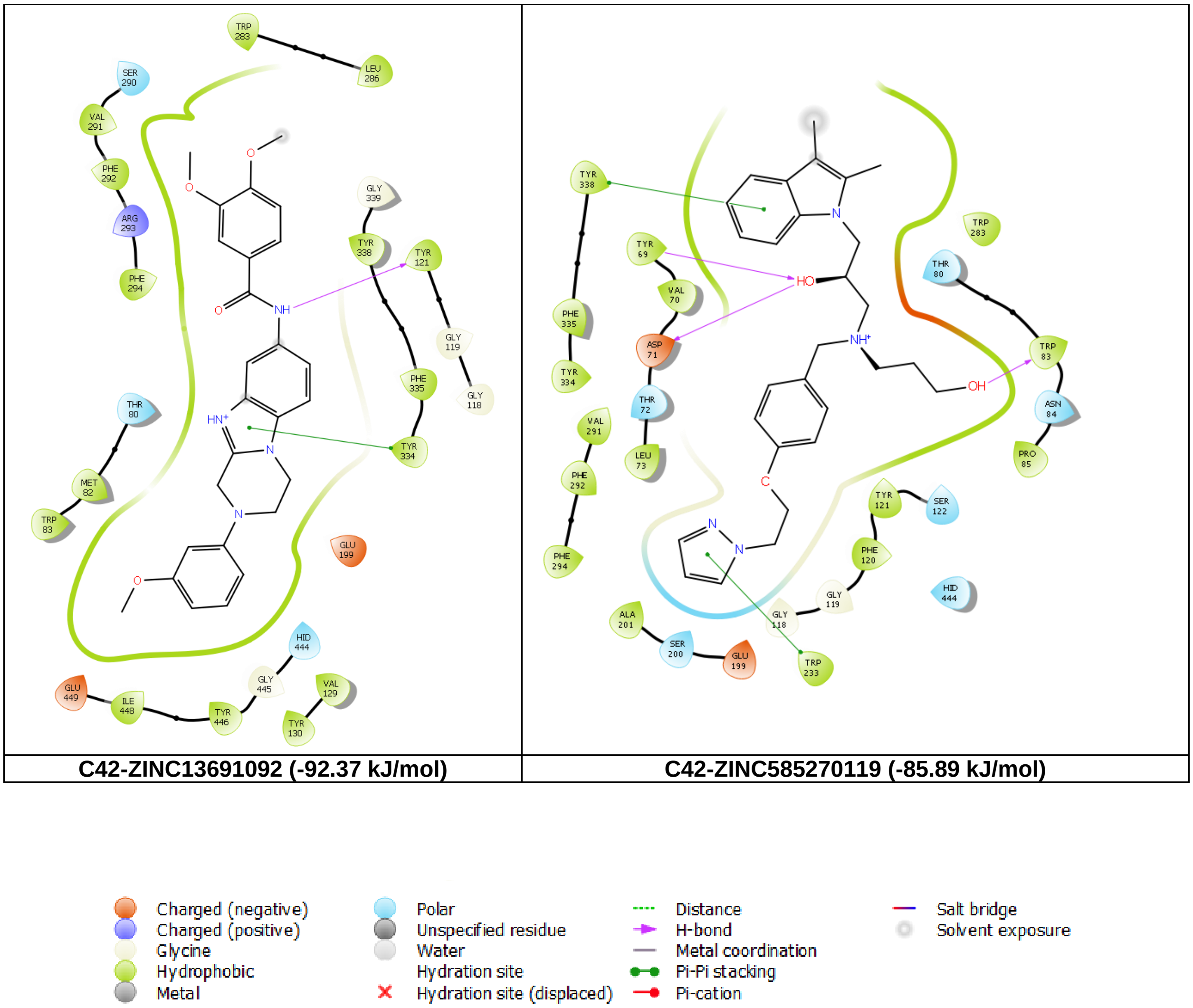

Figure S105. 2D Interaction diagrams of the two compounds with the lowest MMGBSA binding energy for C42 family from the virtual screening of the ZINC library.

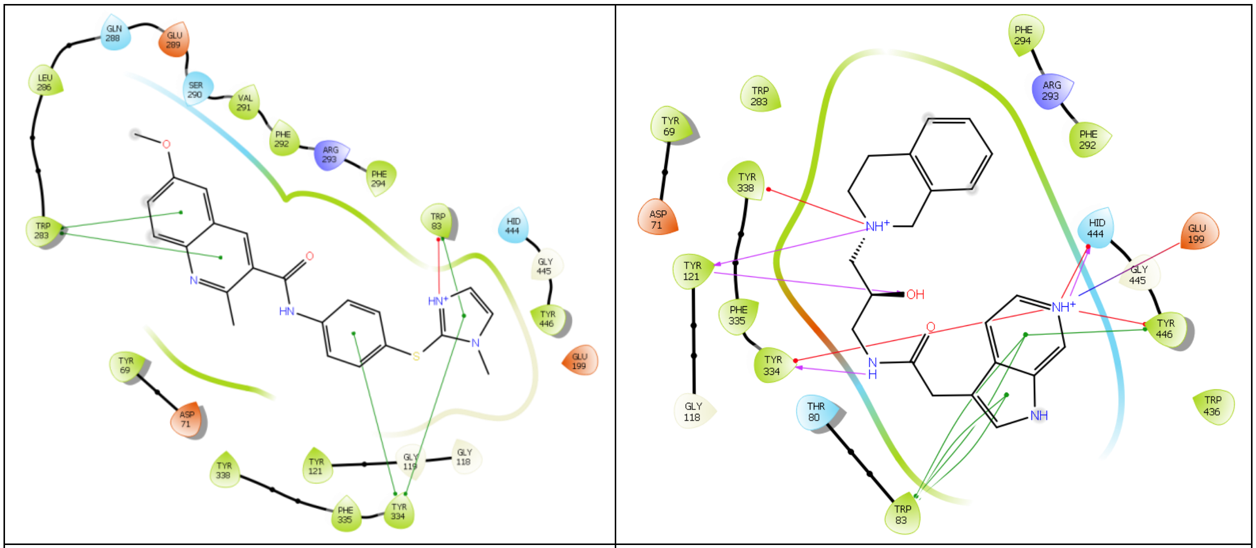

C50-ZINC58382287 (-91.71 kJ/mol)	C50-ZINC2325711293 (-88.87 kJ/mol)

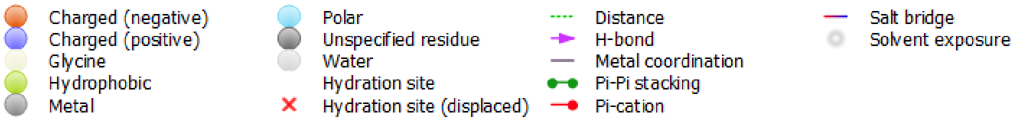

Figure S106. 2D Interaction diagrams of the two compounds with the lowest MMGBSA binding energy for C50 family from the virtual screening of the ZINC library.

References

(1) Wang, J.; Wolf, R. M.; Caldwell, J. W.; Kollman, P. A.; Case, D. A. Development and Testing of a General Amber Force Field. *J. Comput. Chem.* **2004**, *25* (9), 1157–1174. https://doi.org/10.1002/JCC.20035.

(2) Maier, J. A.; Martinez, C.; Kasavajhala, K.; Wickstrom, L.; Hauser, K. E.; Simmerling, C. Ff14SB: Improving the Accuracy of Protein Side Chain and Backbone Parameters from Ff99SB. *J. Chem. Theory Comput.* **2015**, *11* (8), 3696–3713. https://doi.org/10.1021/ACS.JCTC.5B00255/SUPPL_FILE/CT5B00255_SI_001.PDF.

(3) Sousa Da Silva, A. W.; Vranken, W. F. ACPYPE - AnteChamber PYthon Parser InterfacE. *BMC Res. Notes* **2012**, *5* (1), 1–8. https://doi.org/10.1186/1756-0500-5-367/FIGURES/3.

(4) *RmsdByResidue - PyMOLWiki*. https://pymolwiki.org/index.php/RmsdByResidue (accessed 2023-04-30).
